# Hyperglycemia impairs cognitive function by inducing mitochondrial damage through lactylation of LRPPRC at K223

**DOI:** 10.1038/s44321-026-00422-8

**Published:** 2026-04-06

**Authors:** Jingxi Xu, Yuqi Hao, Jingxue Cao, Xing Yang, Yun Gao, Xiaodong Sun, Rongrong Nie, Qiongsui Zhong, Yuanmei Zhong, Junjia Zhong, Tianpeng Zheng

**Affiliations:** 1 Department of Endocrinology and Metabolism, The Second Affiliated Hospital of Guilin Medical University, Guilin, Guangxi 541199 P. R. China; 2 Guangxi Key Laboratory of Diabetic Systems Medicine, Guilin Medical University, Guilin, Guangxi 541199 P. R. China; 3 Guangxi Key Laboratory of Brain and Cognitive Neuroscience, Guilin Medical University, Guilin, Guangxi 541199 P. R. China; 4 Department of Endocrinology and Metabolism, West China Hospital of Sichuan University, Chengdu, Sichuan 610041 P. R. China; 5 Department of Endocrinology and Metabolism, Affiliated Hospital of Shandong Second Medical University, Weifang, 261031 P. R. China; 6 The Department of Integrated Traditional Chinese and Western Medicine, The First Affiliated Hospital of Guilin Medical University, Guilin, Guangxi 541001 P. R. China; 7 The Second Clinical College of Guilin Medical University, Guilin, Guangxi 541199 P. R. China

**Keywords:** Metabolism, Neuroscience

## Abstract

High glucose impairs cognitive function in type 2 diabetes, but the underlying mechanism is unclear. In this study, guided by lactylome analysis, we reveal that high glucose induces LRPPRC K223 lactylation in hippocampal neurons by upregulating lactyltransferase AARS2, which weakens LRPPRC-SLIRP binding, reduces mitochondrial mRNA stability, subsequently leads to mitochondrial dysfunction, and ultimately results in neuronal apoptosis and cognitive decline. Notably, a novel short peptide designed to competitively inhibit LRPPRC K223 lactylation remarkably ameliorates cognitive impairment in diabetic mice. Moreover, through a large prospective cohort study, elevated plasma LRPPRC K224 lactylation (the human homolog of mouse LRPPRC K223) was identified as an independent predictor of cognitive impairment in type 2 diabetes patients. This work uncovers a key mechanism linking high glucose-induced lactylation to mitochondrial dysfunction and neuronal apoptosis, offering new molecular targets for prevention and treatment of diabetes-related cognitive impairment.

## Synopsis

The mechanism of diabetic cognitive impairment remains unclear. This study demonstrates that high glucose-induced LRPPRC K223 lactylation leads to a decline in mitochondrial mRNA stability and mitochondrial dysfunction in hippocampal neurons, resulting in neuronal damage and cognitive impairment.

- Under high glucose conditions, upregulated AARS2 leads to LRPPRC lactylation at K223, disrupting its interaction with SLIRP, destabilizing mitochondrial mRNA, ultimately causing hippocampal neuronal apoptosis and cognitive impairment.
- A newly developed short peptide, K223-pe, enhances mitochondrial function by regulating the LRPPRC/SLIRP/mitochondrial mRNA signaling, thereby reducing neuronal apoptosis and improving cognitive function.
- Increased plasma levels of LRPPRC K224 lactylation (homologous to mouse LRPPRC K223) independently predict the risk of mild cognitive impairment in diabetic patients.

## Abstract (web summary)

The mechanism of diabetic cognitive impairment remains unclear. This study demonstrates that high glucose-induced LRPPRC K223 lactylation leads to a decline in mitochondrial mRNA stability and mitochondrial dysfunction in hippocampal neurons, resulting in neuronal damage and cognitive impairment.

## Graphical abstract

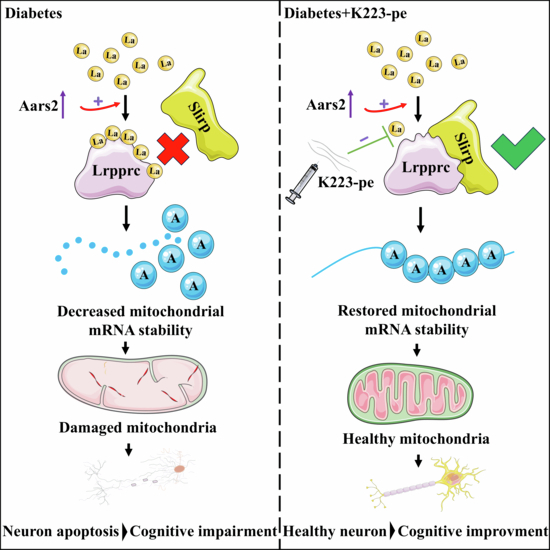

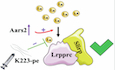

The paper explained **Problem**

Diabetes and cognitive impairment are interconnected in a vicious cycle: poor diabetes management increases the risk of cognitive decline, and impaired cognition subsequently hinders diabetes self-management, further elevating the risks of hospitalization, disability, and mortality. Nonetheless, there remains a lack of effective methods to predict cognitive impairment in diabetic patients, and treatments capable of reversing cognitive decline are still unavailable. Therefore, in-depth investigation of the mechanisms underlying diabetes-related cognitive impairment is crucial for developing targeted screening and treatment strategies.\

**Results**

High glucose upregulates AARS2, which leads to LRPPRC K223 lactylation in hippocampal neurons, impairing LRPPRC-SLIRP binding, thereby destabilizing mitochondrial mRNA, triggering mitochondrial dysfunction and ultimately causing neuronal apoptosis and cognitive impairment. Both the AARS2 conditional knockout and the LRPPRC K223R mutation reversed these effects. To overcome clinical limitations of AAV-based approaches, we designed a short peptide that competitively inhibits LRPPRC K223 lactylation, thereby ameliorating cognitive dysfunction by regulating the LRPPRC/SLIRP/mitochondrial mRNA pathway. A prospective cohort study further confirmed elevated plasma LRPPRC K224 lactylation (homologous to mouse LRPPRC K223) as an independent risk factor for cognitive impairment in elderly type 2 diabetic patients.

**Impact**

This study offers promising molecular targets and therapeutic avenues for the future prevention and treatment of diabetes-related cognitive impairment.

## Introduction

The global prevalence of diabetes is escalating rapidly, with age-standardized prevalence rates reaching 13.9% in women and 14.3% in men, resulting in an estimated 828 million adults having diabetes in 2022 (NCD Risk Factor Collaboration, 2024). Diabetic patients face a significantly increased risk of cognitive decline, with a 1.25 to 1.91 times higher risk of developing cognitive impairment compared to individuals with normal glucose tolerance (Xue et al, 2019). Even more concerning is the alarmingly high incidence of mild cognitive impairment (MCI) among elderly patients with type 2 diabetes, which ranges from 30 to 60% (Battini et al, 2024). Diabetes and cognitive impairment are interconnected in a vicious cycle: poor diabetes management increases the risk of cognitive decline, and impaired cognition subsequently hinders diabetes self-management, further elevating the risks of hospitalization, disability, and mortality (Bandosz et al, 2020; Srikanth et al, 2020). Nonetheless, there remains a lack of effective methods to predict cognitive impairment in diabetic patients, and treatments capable of reversing cognitive decline are still unavailable (Chu et al, 2025; Harwood et al, 2023). Therefore, in-depth investigation of the mechanisms underlying diabetes-related cognitive impairment is crucial for developing targeted screening and treatment strategies.

As a novel post-translational modification of proteins, lactylation has been shown to play a significant role in various pathophysiological processes (Hu et al, 2024). Recent studies have confirmed that lactylation also plays a key role in regulating cognitive function (Pan et al, 2022; Tian et al, 2025). Importantly, high glucose levels can significantly increase protein lactylation (Chen et al, 2024; Lin et al, 2024). However, whether and how high glucose impairs cognitive function through lactylation remains unclear.

Mitochondria are abundant organelles in neurons, serving as the primary energy source for neurons and playing a vital role in maintaining neuronal activity and cognitive function (Duarte et al, 2023; Song et al, 2024). Mitochondrial dysfunction has been established to drive the development and progression of various cognitive disorders, including Alzheimer’s disease, epilepsy, and autism spectrum disorder (D’Alessandro et al, 2025; Khaliulin et al, 2025; Zsurka and Kunz, 2015). Previous research indicates that lactylation of mitochondrial proteins can lead to mitochondrial dysfunction (Hong et al, 2024). However, it remains unclear whether and how high glucose can cause mitochondrial damage and cognitive impairment through lactylation of mitochondrial proteins.

In this study, we conducted a comprehensive lactylation proteomic analysis and found that under high-glucose conditions, the expression of the lactyltransferase AARS2 was upregulated, which led to the lactylation of LRPPRC—a key protein that stabilizes mitochondrial mRNA—at the K223 site. Subsequently, LRPPRC K223 lactylation reduced the binding affinity between LRPPRC and SLIRP, causing decreased mitochondrial mRNA stability in hippocampal neurons. This cascade of events led to mitochondrial dysfunction, ultimately resulting in neuronal apoptosis and cognitive impairment. Notably, conditional knockout of AARS2 and the K223R point mutation (lysine to arginine) in LRPPRC both reversed the above effects. Given the limitations of AAV-mediated gene knockout and point mutation approaches in clinical applications, we designed a short peptide specifically targeting LRPPRC K223 lactylation. This peptide competitively inhibited LRPPRC K223 lactylation, thereby ameliorating high glucose-induced cognitive dysfunction by modulating the LRPPRC/SLIRP/mitochondrial mRNA signaling pathway. Moreover, through a large prospective cohort study (*n* = 1870), we confirmed that elevated plasma LRPPRC K224 lactylation (homologous to mouse LRPPRC K223) is an independent risk factor for predicting cognitive impairment in elderly patients with type 2 diabetes. These findings demonstrate that high glucose-induced LRPPRC K223 lactylation leads to a decline in mitochondrial mRNA stability and mitochondrial dysfunction in hippocampal neurons, resulting in neuronal damage and cognitive impairment. Consequently, this study offers promising molecular targets and therapeutic avenues for the future prevention and treatment of such diseases.

## Results

### The mitochondrial protein LRPPRC K223 is lactylated under high glucose conditions

**Figure 1:**
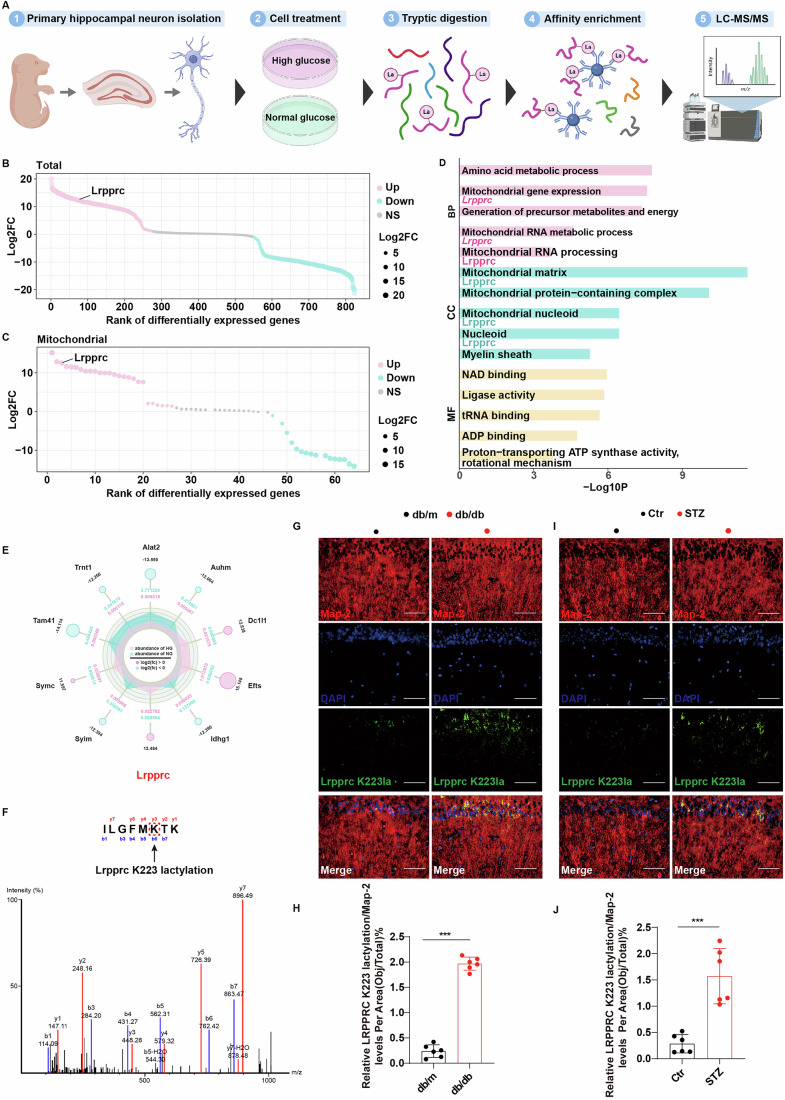
The mitochondrial protein LRPPRC K223 is lactylated under high glucose conditions. (**A**) Schematic representation of experimental workflow for quantification of lactylation in primary hippocampal neurons treated with normal glucose (NG, 5.5 mmol/L D-glucose) or high glucose (HG, 25 mmol/L D-glucose). (**B**) Scatter plot of differentially lactylated sites in all proteins in high glucose versus normal glucose-treated hippocampal neurons. (**C**) Scatter plot of differentially lactylated sites in mitochondrial proteins in high glucose versus normal glucose-treated hippocampal neurons. (**D**) GO analysis of differentially lactylated mitochondrial proteins. BP biological process, CC cellular component, MF molecular function. (**E**) Radar diagram showing the top ten differentially lactylated mitochondrial proteins in high glucose-treated hippocampal neurons. (**F**) Illustration of LRPPRC K223 lactylation identified by MS. (**G**,** H**) Representative images (**G**) and quantifications (**H**) showing IF staining of Lrpprc K223la (green) and MAP-2 (red) in the hippocampal CA1 neurons of db/m or db/db mice (*n* = 6 mice per group). Scale bar: 100 μm. *P* value <0.0001. (**I**,** J**) Representative images (**I**) and quantifications (**J**) showing IF staining of Lrpprc K223la (green) and MAP-2 (red) in the hippocampal CA1 neurons of Ctr or STZ mice (*n* = 6 mice per group). Scale bar: 100 μm. *P* value: 0.0002. Data were means ± SEM. *P < 0.05, **P < 0.01, ***P < 0.001. Hypergeometric test (**D**), Two-tailed Student’s unpaired *t*-test analysis (**H**, **J**). Source data are available online for this figure.

To elucidate the effect of high glucose on protein lactylation in hippocampal neurons, we performed a comprehensive characterization of the hippocampal neuronal lactylome under high glucose conditions (Fig. 1A). Quantitative lactylome profiling revealed that exposure of hippocampal neurons to high glucose resulted in altered lactylation of 825 lysine sites (Kla) distributed across 430 proteins (Fig. 1B). We observed substantial lactylation occurring on mitochondrial proteins (Fig. 1C). Normal mitochondrial function has been confirmed to play a crucial role in maintaining neuronal survival and cognitive function (Duarte et al, 2023; Song et al, 2024). Therefore, we focused our subsequent research on mitochondrial proteins. The differentially lactylated mitochondrial proteins (DLMPs) were mainly enriched in mitochondrial RNA processing and RNA degradation pathways, according to Gene Ontology (GO) and Kyoto Encyclopedia of Genes and Genomes (KEGG) analysis (Fig. 1D; Appendix Fig. S1A). The top ten most differentially lactylated mitochondrial proteins are presented in Fig. 1E.

Considering the above results, we focused our research on LRPPRC, a mitochondrial protein exhibiting the top three upregulated lactylation and known to play a critical role in regulating mitochondrial mRNA stability. Quantitative lactylome profiling revealed a significant increase in lactylation at the K223 site of LRPPRC following high glucose treatment (Fig. 1F).

Previous studies have confirmed that the hippocampal CA1 region plays a critical role in learning and memory (Ramsaran et al, 2023; Sun et al, 2019). Therefore, we focused our subsequent in vivo studies on the hippocampal CA1 region. To investigate whether high glucose induces elevated LRPPRC K223 lactylation in vivo, we employed a specific LRPPRC K223la antibody (Appendix Fig. S1B) to detect LRPPRC K223 lactylation levels in the hippocampal CA1 region of diabetic mice. Immunofluorescence analysis confirmed that LRPPRC K223 lactylation levels were significantly elevated in the hippocampal CA1 region of both type 1 (STZ-induced) and type 2 (db/db) diabetic mice compared with their respective control groups (Fig. 1G–J). Meanwhile, the lysine 223 residue of LRPPRC and its surrounding residues are highly conserved across diverse species, including *Homo sapiens*, *Mus musculus*, *Rattus norvegicus*, *Pongo abelii*, *Canis lupus familiaris*, *Panthera leo*, *Mesocricetus auratus*, *Pan troglodytes*, *Lynx canadensis*, *Vulpes vulpes*, and *Camelus ferus* (Appendix Fig. S1C).

### LRPPRC K223 lactylation results in hippocampal neuronal apoptosis and cognitive impairment

To further investigate the impact of LRPPRC K223 lactylation on neuronal survival, we mutated K223 to arginine (K223R) or threonine (K223T), which mimicked the delactylated or hyperlactylated state of the protein, and transfected wild-type (WT) LRPPRC, K223R, or K223T mutated LRPPRC in LRPPRC knockdown primary hippocampal neurons (Appendix Fig. S2A,B) under normal and high glucose conditions. As illustrated in Appendix Fig. S2C–F, high glucose (HG) treatment significantly increased apoptosis in primary hippocampal neurons compared to the normal glucose (NG) group. Under high glucose conditions, the K223R group showed markedly reduced apoptosis compared with the WT group. In contrast, the K223T group exhibited even higher apoptosis levels than the WT group, demonstrating that high glucose-induced LRPPRC K223 lactylation triggers hippocampal neuronal apoptosis. Previous studies have reported that high glucose may affect ubiquitination at specific sites, but these alterations appear to be relatively infrequent (Liang et al, 2024; Zhou et al, 2007). Consistent with this, our data indicate that LRPPRC exhibits minimal ubiquitination under both high- and normal-glucose conditions; the LRPPRC K223R and K223T mutations do not affect this PTM and subsequent regulation (Appendix Fig. S2G).

**Figure 2:**
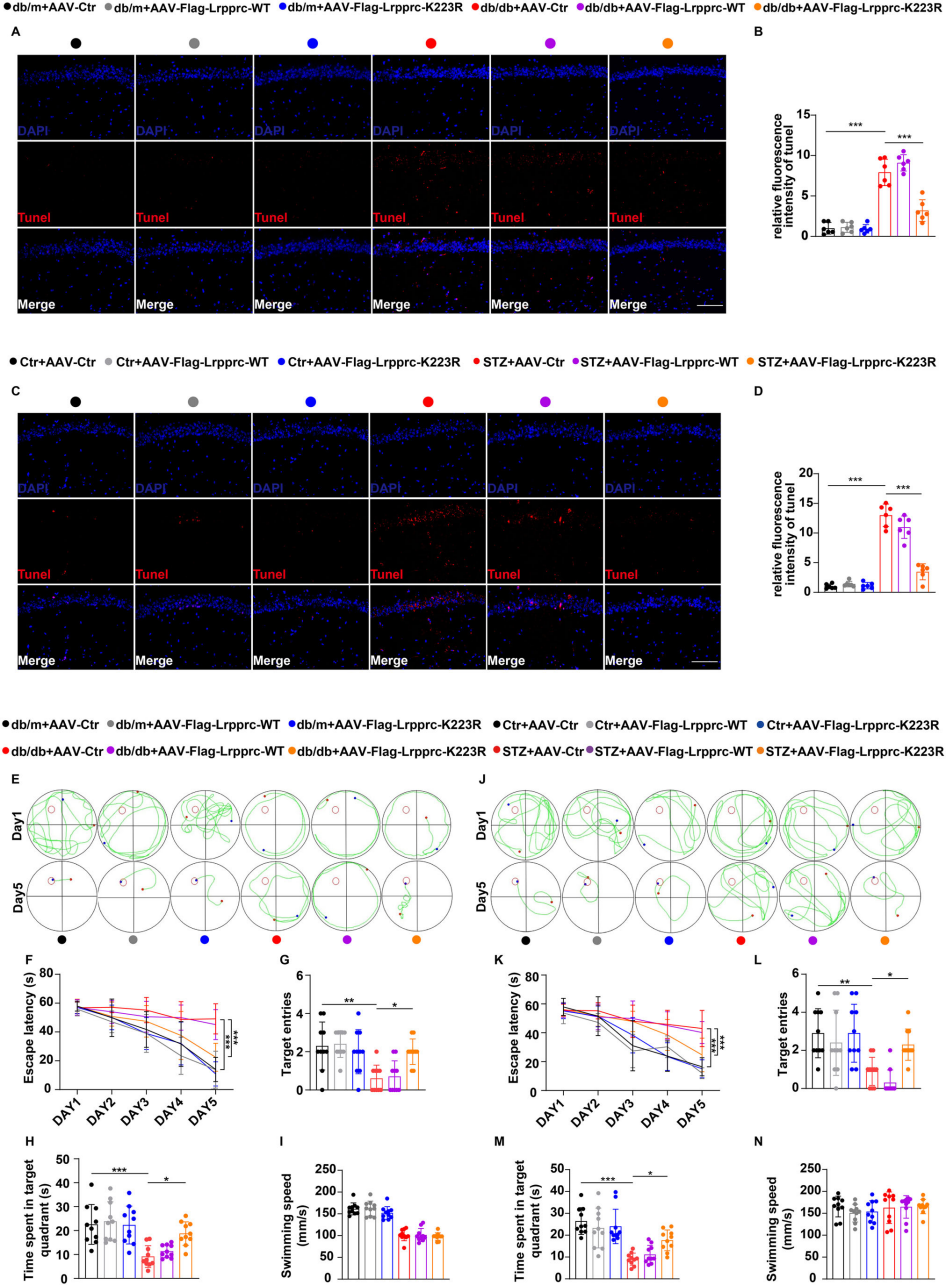
LRPPRC K223 lactylation results in hippocampal neuronal apoptosis and cognitive impairment. (**A**,** B**) Representative images (**A**) and quantifications (**B**) showing IF staining of Tunel (red) in the hippocampal CA1 neurons of db/m or db/db mice treated with AAV-Ctr, AAV-Flag-LRPPRC-WT, or AAV-Flag-LRPPRC-K223R (*n* = 6 mice per group). Scale bar: 100 μm. *P* values: <0.0001 (db/m + AAV-Ctr vs db/db+AAV-Ctr), <0.0001 (db/db + AAV-Ctr vs db/db + AAV-Flag-LRPPRC-K223R). (**C**, **D**) Representative images (**C**) and quantifications (**D**) showing IF staining of Tunel (red) in the hippocampal CA1 neurons of Ctr or STZ mice treated with AAV-Ctr, AAV-Flag-LRPPRC-WT, or AAV-Flag-LRPPRC-K223R (*n* = 6 mice per group). Scale bar: 100 μm. *P* values: <0.0001 (Ctr + AAV-Ctr vs STZ + AAV-Ctr), <0.0001 (STZ + AAV-Ctr vs STZ + AAV-Flag-LRPPRC-K223R). (**E**–**I**) Representative track images (**E**), escape latency to the platform (**F**), and swimming speed (**I**) during the training trials, target entries (**G**) and time spent in target quadrant (**H**) in the probe trial of Morris water maze of db/m or db/db mice injected with AAV-Ctr, AAV-Flag-LRPPRC-WT, or AAV-Flag-LRPPRC-K223R (*n* = 10 mice per group). *P* values: <0.0001 (**F**, escape latency, db/m + AAV-Ctr vs db/db + AAV-Ctr), <0.0001 (F, escape latency, db/db + AAV-Ctr vs db/db + AAV-Flag-LRPPRC-K223R), 0.0015 (**G**, target entries, db/m + AAV-Ctr vs db/db+AAV-Ctr), 0.0141 (**G**, target entries, db/db + AAV-Ctr vs db/db + AAV-Flag-LRPPRC-K223R), 0.0003 (**H**, time spent in target quadrant, db/m + AAV-Ctr vs db/db + AAV-Ctr), 0.0188 (**H**, time spent in target quadrant, db/db + AAV-Ctr vs db/db + AAV-Flag-LRPPRC-K223R). (**J**–**N**) Representative track images (**J**), escape latency to the platform (**K**), and swimming speed (**N**) during the training trials, target entries (**L**) and time spent in the target quadrant (**M**) in the probe trial of Morris water maze of Ctr or STZ mice injected with AAV-Ctr, AAV-Flag-LRPPRC-WT, or AAV-Flag-LRPPRC-K223R (*n* = 10 mice per group). *P* values: <0.0001 (**K**, escape latency, Ctr + AAV-Ctr vs STZ + AAV-Ctr), 0.0002 (**K**, escape latency, STZ + AAV-Ctr vs STZ + AAV-Flag-LRPPRC-K223R), 0.0017 (**L**, target entries, Ctr + AAV-Ctr vs STZ + AAV-Ctr), 0.0439 (**L**, target entries, STZ + AAV-Ctr vs STZ + AAV-Flag-LRPPRC-K223R), <0.0001 (**M**, time spent in target quadrant, Ctr + AAV-Ctr vs STZ + AAV-Ctr), 0.0340 (**M**, time spent in target quadrant, STZ + AAV-Ctr vs STZ + AAV-Flag-LRPPRC-K223R). Data were means ± SEM. *p < 0.05, **P < 0.01, ***p < 0.001. Two-way ANOVA followed by Tukey’s test (**B**, **D**, **F**–**I**, **K**, **L**). Source data are available online for this figure.

To explore whether LRPPRC K223 lactylation causes cognitive dysfunction in vivo, we specifically expressed LRPPRC WT or LRPPRC K223R mutants in CA1 neurons of diabetic mice and subsequently assessed neuronal apoptosis and cognitive function. The purity of isolated hippocampal CA1 neurons was validated via flow cytometry (Appendix Fig. S2H). As shown in Fig. 2A–N, expression of LRPPRC K223R, but not LRPPRC WT, in CA1 neurons of type 1 and type 2 diabetic mice significantly reduced neuronal apoptosis and improved cognitive function. These results demonstrate that high-glucose-induced LRPPRC K223 lactylation leads to hippocampal neuronal apoptosis and cognitive impairment.

### LRPPRC K223 lactylation reduces its binding to SLIRP, weakens mitochondrial mRNA stability, and triggers subsequent mitochondrial dysfunction

**Figure 3:**
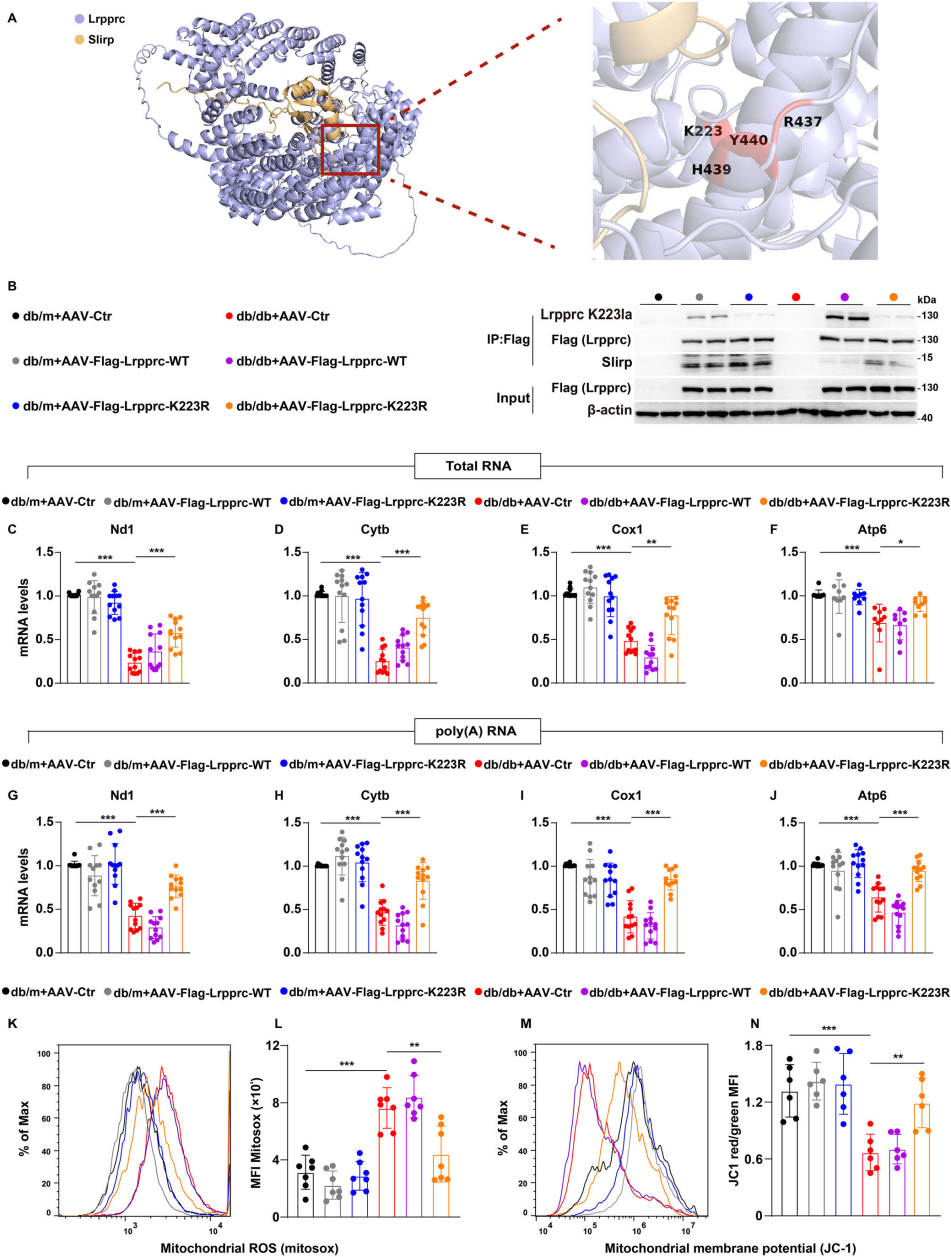
LRPPRC K223 lactylation reduces its binding to SLIRP, weakens mitochondrial mRNA stability, and triggers subsequent mitochondrial dysfunction. (**A**) Detailed position of the LRPPRC lactylation site K223 in relation to the LRPPRC-SLIRP binding sites R437, H439, and Y440. (**B**) Hippocampal CA1 neurons were isolated from db/m, db/db mice that received stereotactic injections of AAV-Ctr, AAV-Flag-LRPPRC-WT, or AAV-Flag-LRPPRC-K223R to specifically express either wild-type LRPPRC or the K223R mutant LRPPRC, and cell lysates were immunoprecipitated with anti-Flag antibodies and western blotted with the indicated antibodies (*n* = 6 mice per group). (**C**–**J**) Total RNA (**C**–**F**) or poly(A)-tailed RNA (**G**–**J**) was isolated from the hippocampal CA1 neurons of db/m, db/db mice that received stereotactic injections of AAV-Ctr, AAV-Flag-LRPPRC-WT, or AAV-Flag-LRPPRC-K223R to specifically express either wild-type LRPPRC or the K223R mutant LRPPRC. The levels of each mtRNA species were determined by PCR (*n* = 9–12 mice per group). *P* values: <0.0001 (**C**, Nd1, db/m + AAV-Ctr vs db/db+AAV-Ctr), <0.0001 (**C**, Nd1, db/db + AAV-Ctr vs db/db + AAV-Flag-LRPPRC-K223R), <0.0001 (**D**, Cytb, db/m + AAV-Ctr vs db/db + AAV-Ctr), <0.0001 (**D**, Cytb, db/db + AAV-Ctr vs db/db + AAV-Flag-LRPPRC-K223R), <0.0001 (**E**, Cox1, db/m + AAV-Ctr vs db/db + AAV-Ctr), 0.0011 (**E**, Cox1, db/db + AAV-Ctr vs db/db + AAV-Flag-LRPPRC-K223R), 0.0002 (**F**, Atp6, db/m + AAV-Ctr vs db/db + AAV-Ctr), 0.0309 (**F**, Atp6, db/db + AAV-Ctr vs db/db + AAV-Flag-LRPPRC-K223R), <0.0001 (**G**, Nd1, db/m + AAV-Ctr vs db/db+AAV-Ctr), <0.0001 (**G**, Nd1,db/db + AAV-Ctr vs db/db + AAV-Flag-LRPPRC-K223R), <0.0001 (**H**, Cytb, db/m + AAV-Ctr vs db/db + AAV-Ctr), <0.0001 (**H**, Cytb, db/db + AAV-Ctr vs db/db + AAV-Flag-LRPPRC-K223R), <0.0001 (**I**, Cox1, db/m + AAV-Ctr vs db/db + AAV-Ctr), <0.0001 (**I**, Cox1, db/db + AAV-Ctr vs db/db+AAV-Flag-LRPPRC-K223R), <0.0001 (**J**, Atp6, db/m + AAV-Ctr vs db/db+AAV-Ctr), <0.0001 (**J**, Atp6, db/db + AAV-Ctr vs db/db + AAV-Flag-LRPPRC-K223R). (**K, L**) Flow cytometry (**K**) and quantification analysis (**L**) of mtROS levels in the hippocampal CA1 neurons of db/m or db/db mice received stereotactic injections of AAV-Ctr, AAV-Flag-LRPPRC-WT, or AAV-Flag-LRPPRC-K223R to specifically express either wild-type LRPPRC or the K223R mutant LRPPRC (*n* = 7 mice per group). *P* values: <0.0001 (db/m + AAV-Ctr vs db/db + AAV-Ctr), 0.0013 (db/db + AAV-Ctr vs db/db + AAV-Flag-LRPPRC-K223R). (**M**,** N**): Flow cytometry (**M**) and quantification analysis (**N**) of MMP levels in the hippocampal CA1 neurons of db/m or db/db mice received stereotactic injections of AAV-Ctr, AAV-Flag-LRPPRC-WT, or AAV-Flag-LRPPRC-K223R to specifically express either wild-type LRPPRC or the K223R mutant LRPPRC (*n* = 6 mice per group). *P* values: 0.0008 (db/m + AAV-Ctr vs db/db + AAV-Ctr), 0.0057 (db/db + AAV-Ctr vs db/db + AAV-Flag-LRPPRC-K223R). Data were means ± SEM. *p < 0.05, **P < 0.01, ***p < 0.001. Two-way ANOVA followed by Tukey’s test (**C**–**J**, **L**, **N**). Source data are available online for this figure.

Given that LRPPRC K223 lactylation leads to hippocampal neuron apoptosis and cognitive impairment, we further investigated the underlying molecular mechanisms involved. LRPPRC needs to bind to another important mitochondrial protein, SLIRP, to maintain the stability of mitochondrial mRNA (Moran et al, 2024; Ruzzenente et al, 2012; Singh et al, 2024). Previous studies have shown that post-translational modifications near the protein–protein binding site can inhibit interprotein binding events (Duan and Walther, 2015; Kocyła et al, 2021). To investigate whether the LRPPRC K223 lactylation inhibits the binding of LRPPRC to SLIRP, we first used AlphaFold to identify the binding sites between LRPPRC and SLIRP. Next, we analyzed the spatial relationship between K223 and the LRPPRC-SLIRP binding sites, and found that K223 is located close to the binding sites R437, H439, and Y440 (Fig. 3A). To examine which binding site is functionally critical for the LRPPRC-SLIRP interaction, we transfected WT LRPPRC or LRPPRC mutants (R437A, H439A, or Y440A) into primary hippocampal neurons. As shown in Appendix Fig. S3A,B, compared to WT LRPPRC, the Y440A mutant markedly reduced the binding between LRPPRC and SLIRP, whereas R437A and H439A had limited effects. These data identify Y440 as the functionally critical site within the LRPPRC-SLIRP binding interface. To investigate whether LRPPRC K223R affects this binding, we generated a double mutant (K223R/Y440A) LRPPRC. As shown in Appendix Fig. S3C,D, under high glucose conditions, the K223R mutation significantly enhanced LRPPRC–-SLIRP binding compared to the WT group. However, in the Y440A background, the K223R mutation failed to restore LRPPRC–SLIRP interaction, regardless of glucose conditions. Our further experimental results demonstrate that in the hippocampal CA1 neurons isolated from both type 1 and type 2 diabetic mice, the lactylation level of LRPPRC at K223 is elevated, and the binding between LRPPRC and SLIRP is reduced (Fig. 3B; Appendix Fig. S3E). The LRPPRC K223R point mutation in hippocampal CA1 neurons of diabetic mice reversed this effect (Fig. 3B; Appendix Fig. S3E). Similarly, in vitro experiments demonstrated that high glucose (HG) treatment significantly reduced the binding between LRPPRC and SLIRP compared to the normal glucose (NG) group (Appendix Fig. S3F–H). Under high glucose conditions, the K223R group showed restored binding between LRPPRC and SLIRP compared with the WT group; by contrast, the K223T group exhibited even lower binding between LRPPRC and SLIRP than the WT group (Appendix Fig. S3F–H). These results confirm that LRPPRC K223 lactylation inhibits the binding of LRPPRC and SLIRP.

Subsequently, we investigated the impact of LRPPRC K223 lactylation-induced reduction in LRPPRC-SLIRP interaction on the stability of mitochondrial mRNA in hippocampal neurons. A previous study by Singh et al identified that Nd1, Cytb, Cox1, and Atp6 represent the most significantly downregulated members among all mitochondrial mRNAs encoding subunits of mitochondrial Complexes I, III, IV, and ATP synthase, respectively, following LRPPRC knockout (Singh et al, 2024). Accordingly, we selected these RNAs as representative indicators for subsequent analyses. Both in vivo and in vitro experiments demonstrated that LRPPRC K223 lactylation-mediated reduction of LRPPRC-SLIRP binding leads to decreased protein and mRNA levels of total Nd1 (Complex I), Cytb (Complex III), Cox1 (Complex IV), and Atp6 (ATP synthase) (Fig. 3C–F; Appendix Fig. S4A–R). Since polyadenylation stabilizes mRNA (Biziaev et al, 2024; Passmore and Coller, 2022), poly(A) RNA levels can reflect mRNA stability (Choi et al, 2024; Gao et al, 2023; Sharma et al, 2016). Therefore, we further assessed mitochondrial poly(A) RNA levels following LRPPRC K223 lactylation. As shown in Fig. 3G–J; Appendix Fig. S4S–Z, disruption of LRPPRC-SLIRP binding through LRPPRC K223 lactylation leads to decreased levels of poly(A) Nd1, Cytb, Cox1, and Atp6, reflecting reduced mitochondrial mRNA stability. Previous studies have indicated that the stability of mitochondrial mRNA is critical for maintaining mitochondrial function (Duan et al, 2025). Accordingly, we assessed several indicators reflecting mitochondrial function and found that reduced mitochondrial mRNA stability results in mitochondrial dysfunction, as indicated by excessive reactive oxygen species (ROS) accumulation and a decline in mitochondrial membrane potential (Fig. 3K–N; Appendix Fig. S5A–J). Importantly, these effects were reversed by the K223R mutation (Fig. 3K–N; Appendix Fig. S5A–J). Additionally, in vitro experimental results demonstrated that under high glucose conditions, the K223T group showed even lower mitochondrial mRNA stability (Appendix Fig. S4O–R,W–Z) and more severe mitochondrial dysfunction compared with the WT group (as indicated by elevated mitochondrial ROS levels, decreased mitochondrial membrane potential and OCR) (Appendix Fig. S5E–J).

In summary, LRPPRC K223 lactylation reduces its binding to SLIRP, weakens mitochondrial mRNA stability, and triggers subsequent mitochondrial dysfunction.

### LRPPRC K223 is lactylated by AARS2 under high-glucose conditions

Lactylation is driven by a series of lactyltransferases (writers) (Zong et al, 2025). To identify the writer responsible for LRPPRC K223 lactylation, a lactyltransferase siRNA library was constructed, and the LRPPRC lactylation level was determined by immunoprecipitation and western blotting. Knocking down AARS2 expression diminished LRPPRC lactylation levels, but no other lactyltransferase exerted this effect (Appendix Fig. S6A,B).

**Figure 4:**
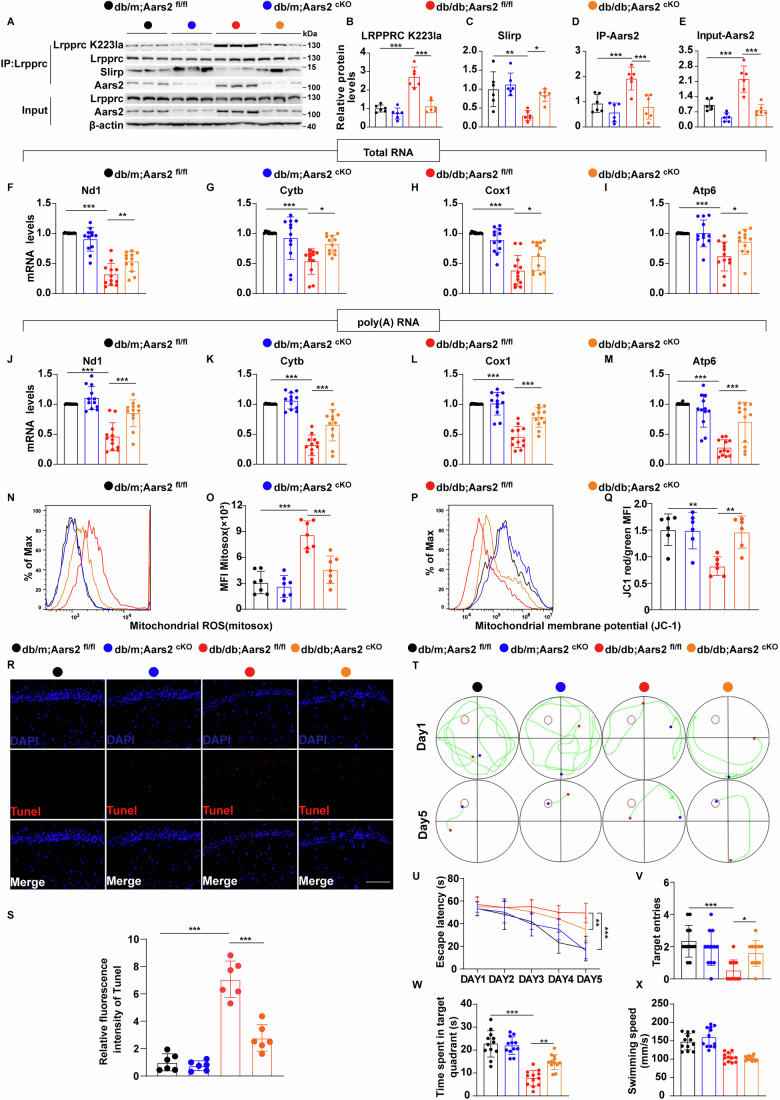
LRPPRC K223 is lactylated by Aars2 under high-glucose conditions. (**A**–**E**) Hippocampal CA1 neurons were isolated from db/m Aars2 ^fl/fl^, db/db Aars2 ^fl/fl^ mice injected with AAV-CAMKII (as a control vector) or AAV-CAMKII-Cre, and cell lysates were immunoprecipitated with LRPPRC antibodies and western blotted with the indicated antibodies (**A**). Quantification analysis of Lrpprc K223la (**B**), Slirp (**C**), IP-Aars2 (**D**), and Input-Aars2 (**E**) levels in the indicated groups (*n* = 6 mice per group). *P* values: <0.0001 (**B**, Lrpprc K223la, db/m;Aars2 ^fl/fl^ vs db/db;Aars2 ^fl/fl^), <0.0001 (**B**, Lrpprc K223la, db/db;Aars2 ^fl/fl^ vs db/db;Aars2 ^cKO^), 0.0029 (**C**, Slirp, db/m;Aars2 ^fl/fl^ vs db/db Aars2 ^fl/fl^), 0.0231 (**C**, Slirp, db/db;Aars2 ^fl/fl^ vs db/db;Aars2 ^cKO^), 0.0006 (**D**, IP-Aars2, db/m;Aars2 ^fl/fl^ vs db/db Aars2 ^fl/fl^), 0.0002 (**D**, IP-Aars2, db/db;Aars2 ^fl/fl^ vs db/db;Aars2 ^cKO^), <0.0001 (**E**, Input-Aars2, db/m;Aars2 ^fl/fl^ vs db/db Aars2 ^fl/fl^), <0.0001 (**E**, Input-Aars2, db/db;Aars2 ^fl/fl^ vs db/db;Aars2 ^cKO^). (**F**–**M**): Total RNA (**F**–**I**) or poly(A)-tailed RNA (**J**–**M**) was isolated from the hippocampal CA1 neurons of db/m Aars2 ^fl/fl^, db/db Aars2 ^fl/fl^ mice injected with AAV-CAMKII (as a control vector), or AAV-CAMKII-Cre (*n* = 12 mice per group). The levels of each mtRNA species were determined by PCR. *P* values: <0.0001 (**F**, Nd1, db/m;Aars2 ^fl/fl^ vs db/db;Aars2 ^fl/fl^), 0.0095 (**F**, Nd1, db/db;Aars2 ^fl/fl^ vs db/db;Aars2 ^cKO^), <0.0001 (**G**, Cytb, db/m;Aars2 ^fl/fl^ vs db/db;Aars2 ^fl/fl^), 0.0149 (**G**, Cytb, db/db;Aars2 ^fl/fl^ vs db/db;Aars2 ^cKO^), <0.0001 (**H**, Cox1, db/m;Aars2 ^fl/fl^ vs db/db;Aars2 ^fl/fl^), 0.0258 (H, Cox1, db/db;Aars2 ^fl/fl^ vs db/db;Aars2 ^cKO^), <0.0001 (**I**, Atp6, db/m;Aars2 ^fl/fl^ vs db/db;Aars2 ^fl/fl^), 0.0205 (**I**, Atp6, db/db;Aars2 ^fl/fl^ vs db/db;Aars2 ^cKO^), <0.0001 (**J**, Nd1, db/m;Aars2 ^fl/fl^ vs db/db;Aars2 ^fl/fl^), <0.0001 (**J**, Nd1, db/db;Aars2 ^fl/fl^ vs db/db;Aars2 ^cKO^), <0.0001 (**K**, Cytb, db/m;Aars2 ^fl/fl^ vs db/db;Aars2 ^fl/fl^), <0.0001 (**K**, Cytb, db/db;Aars2 ^fl/fl^ vs db/db;Aars2 ^cKO^), <0.0001 (**L**, Cox1, db/m;Aars2 ^fl/fl^ vs db/db;Aars2 ^fl/fl^), <0.0001 (**L**, Cox1, db/db;Aars2 ^fl/fl^ vs db/db;Aars2 ^cKO^), <0.0001 (**M**, Atp6, db/m;Aars2 ^fl/fl^ vs db/db;Aars2 ^fl/fl^), 0.0002 (**M**, Atp6, db/db;Aars2 ^fl/fl^ vs db/db;Aars2 ^cKO^). (**N**,** O**) Flow cytometry (**N**) and quantification analysis (**O**) of mtROS levels in the hippocampal CA1 neurons of db/m Aars2 ^fl/fl^, db/db Aars2 ^fl/fl^ mice injected with AAV-CAMKII (as a control vector) or AAV-CAMKII-Cre (*n* = 7 mice per group). *P* values: <0.0001 (db/m;Aars2 ^fl/fl^ vs db/db;Aars2 ^fl/fl^), 0.0001 (db/db;Aars2 ^fl/fl^ vs db/db;Aars2 ^cKO^). (**P**,** Q**) Flow cytometry (**P**) and quantification analysis (**Q**) of MMP levels in the hippocampal CA1 neurons of db/m Aars2 ^fl/fl^, db/db Aars2 ^fl/fl^ mice injected with AAV-CAMKII (as a control vector) or AAV-CAMKII-Cre (*n* = 6 mice per group). *P* values: 0.0026 (db/m;Aars2 ^fl/fl^ vs db/db;Aars2 ^fl/fl^), 0.0049 (db/db;Aars2 ^fl/fl^ vs db/db;Aars2 ^cKO^). (**R**,** S**) Representative images (**R**) and quantifications (**S**) showing IF staining of Tunel (red) in the hippocampal CA1 neurons of db/m Aars2 ^fl/fl^, db/db Aars2 ^fl/fl^ mice injected with AAV-CAMKII or AAV-CAMKII-Cre (*n* = 6 mice per group). Scale bar: 100 μm. *P* values: <0.0001 (db/m;Aars2 ^fl/fl^ vs db/db;Aars2 ^fl/fl^), <0.0001 (db/db;Aars2 ^fl/fl^ vs db/db;Aars2 ^cKO^). (**T**–**X**) Representative track images (**T**), escape latency to the platform (**U**), and swimming speed (**X**) during the training trials, target entries (**V**) and time spent in the target quadrant (**W**) in the probe trial of the Morris water maze of db/m Aars2 ^fl/fl^, db/db Aars2 ^fl/fl^ mice injected with AAV-CAMKII or AAV-CAMKII-Cre (*n* = 12 mice per group). *P* values: <0.0001 (**U**, escape latency, db/m;Aars2 ^fl/fl^ vs db/db;Aars2 ^fl/fl^), 0.0023 (**U**, escape latency, db/db;Aars2 ^fl/fl^ vs db/db;Aars2 ^cKO^), <0.0001 (**V**, target entries, db/m;Aars2 ^fl/fl^ vs db/db;Aars2 ^fl/fl^), 0.0249 (**V**, target entries, db/db;Aars2 ^fl/fl^ vs db/db;Aars2 ^cKO^), <0.0001 (**W**, time spent in target quadrant, db/m;Aars2 ^fl/fl^ vs db/db;Aars2 ^fl/fl^), 0.0010 (**W**, time spent in target quadrant, db/db;Aars2 ^fl/fl^ vs db/db;Aars2 ^cKO^). Data were means ± SEM. *p < 0.05, **P < 0.01, ***p < 0.001. Two-way ANOVA followed by Tukey’s test (**B**–**M**, **O**, **Q**, **S**, **U**–**X**). Source data are available online for this figure.

To further elucidate the role of AARS2 in modulating LRPPRC K223 lactylation, we overexpressed AARS2 in primary hippocampal neurons and observed that AARS2 overexpression promoted its binding to LRPPRC, thereby elevating LRPPRC K223 lactylation levels, and reducing the binding between LRPPRC and SLIRP (Appendix Fig. S6C–G). To validate the above findings under high glucose conditions, primary hippocampal neurons were treated with high glucose, and AARS2 expression was knocked down using siRNA. As illustrated in Appendix Fig. S6H–J, high-glucose exposure led to an increase in AARS2 expression, enhanced the interaction between AARS2 and LRPPRC, and consequently raised LRPPRC K223 lactylation. The above effects were reversed following AARS2 knockdown. Moreover, these findings were validated in vivo: as shown in Fig. 4A–E; Appendix Fig. S6K–O, AARS2 expression was significantly upregulated in the hippocampal CA1 neurons of both type 1 and type 2 diabetic mice models. This upregulation intensified the binding between AARS2 and LRPPRC, resulting in elevated LRPPRC K223 lactylation. Notably, specific deletion of AARS2 in the hippocampal CA1 neurons of type 1 and type 2 diabetic mice (Appendix Fig. S6P,Q) reversed the above effect.

Next, we investigated whether AARS2-mediated lactylation of LRPPRC K223 under high glucose conditions affects the binding capacity of LRPPRC to SLIRP and subsequent mitochondrial mRNA stability. As shown in Fig. 4A–E; Appendix Fig. S6K–O, in hippocampal CA1 neurons of type 1 and type 2 diabetic mice, the binding affinity between LRPPRC and SLIRP was significantly reduced, leading to decreased mitochondrial mRNA stability and subsequent mitochondrial dysfunction (Fig. 4F–Q; Appendix Fig. S7A–L). Deletion of AARS2 specifically in hippocampal CA1 neurons of type 1 and type 2 diabetic mice reversed these effects, thereby resulting in reduced neuronal apoptosis and improved cognitive function (Fig. 4F–X; Appendix Fig. S7A–S). Similar results were observed in primary hippocampal neurons (Appendix Fig. S8A–P).

In summary, under high glucose conditions, the expression of the lactyltransferase AARS2 was upregulated, leading to increased binding between AARS2 and LRPPRC. This leads to increased levels of LRPPRC K223 lactylation, decreased binding of LRPPRC to SLIRP, and subsequent decreased mitochondrial mRNA stability and mitochondrial dysfunction. These changes ultimately lead to hippocampal neuronal apoptosis and cognitive impairment.

### K223-pe enhances mitochondrial function by regulating the LRPPRC/SLIRP/mitochondrial mRNA signaling pathway, thereby reducing neuronal apoptosis and improving cognitive function

**Figure 5:**
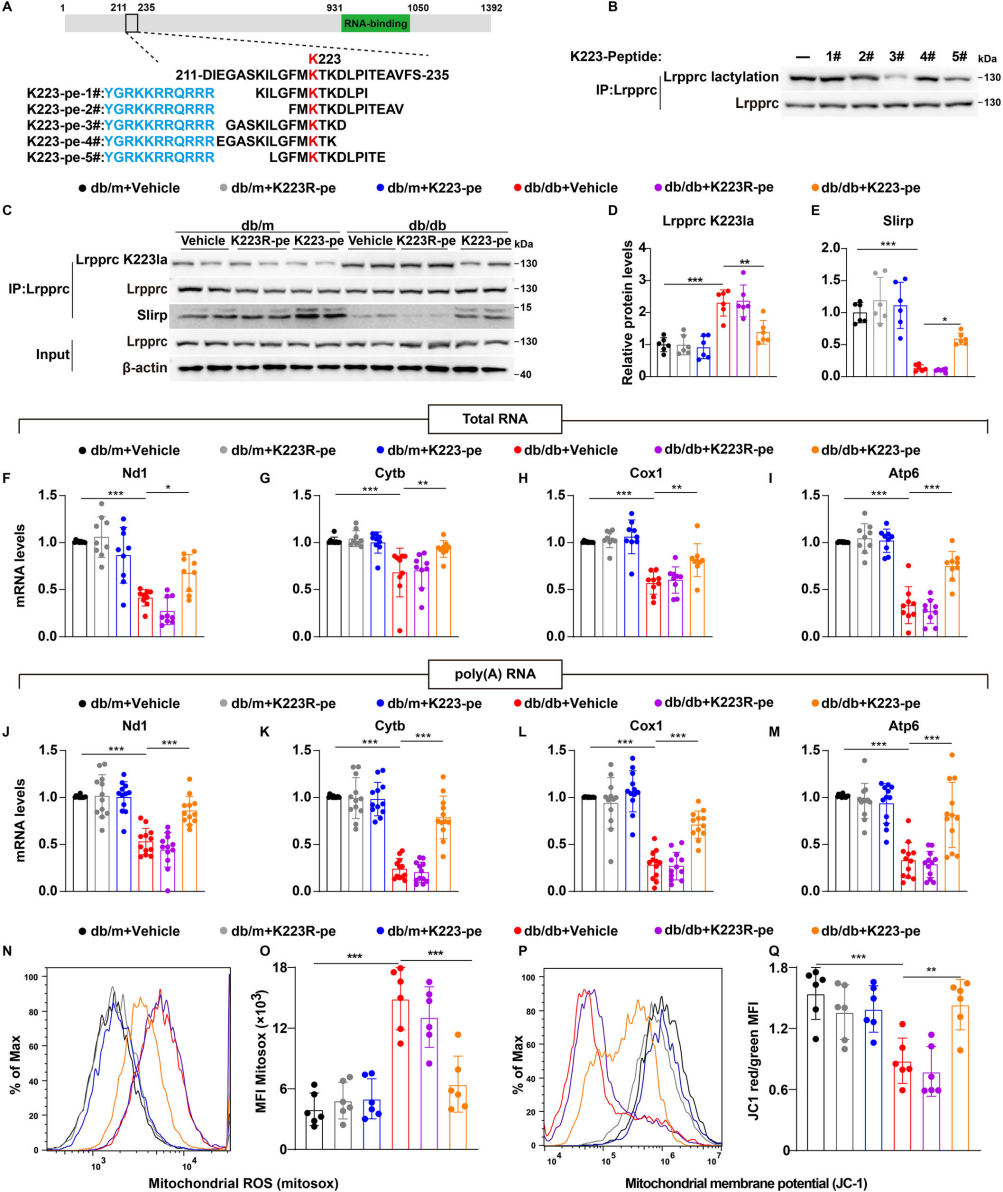
K223-pe enhances mitochondrial function by regulating the LRPPRC/SLIRP/mitochondrial mRNA signaling pathway. (**A**) Schematic illustration of designed peptides. Blue: cell-penetrating peptide (CPP). Red: site for LRPPRC lactylation. (**B**) Primary hippocampal neurons were treated with five short peptides (20 μM) in the presence of high glucose (HG, 25 mmol/L D-glucose), and LRPPRC lactylation was determined by immunoprecipitation and western blot using the indicated antibodies. Three independent experiments were performed. (**C**–**E**) Hippocampal CA1 neurons were isolated from db/m, db/db mice treated with vehicle, K223R-pe (scrambled peptide, 5 mg/kg) or K223-pe (5 mg/kg), and cell lysates were immunoprecipitated with LRPPRC antibodies and western blotted with the indicated antibodies (**C**). Quantification analysis of Lrpprc K223la (**D**) and Slirp (**E**) levels in the indicated groups (*n* = 6 mice per group). *P* values: <0.0001 (**D**, Lrpprc K223la, db/m + vehicle vs db/db + vehicle), 0.0020 (**D**, Lrpprc K223la, db/db + vehicle vs db/db + K223-pe), <0.0001 (**E**, Slirp, db/m + vehicle vs db/db + vehicle), 0.0153 (**E**, Slirp, db/db + vehicle vs db/db + K223-pe). (**F**–**M**) Total RNA (**F**–**I**) or poly(A)-tailed RNA (**J**–**M**) was isolated from the hippocampal CA1 neurons of db/m, db/db mice treated with vehicle, K223R-pe (scrambled peptide, 5 mg/kg) or K223-pe (5 mg/kg). The levels of each mtRNA species were determined by PCR (*n* = 9–12 mice per group). *P* values: <0.0001 (**F**, Nd1, db/m + vehicle vs db/db + vehicle), 0.0421 (**F**, Nd1, db/db + vehicle vs db/db + K223-pe), 0.0002 (**G**, Cytb, db/m + vehicle vs db/db + vehicle), 0.0087 (**G**, Cytb, db/db + vehicle vs db/db+K223-pe), <0.0001 (**H**, Cox1, db/m + vehicle vs db/db + vehicle), 0.0035 (**H**, Cox1, db/db + vehicle vs db/db + K223-pe), <0.0001 (**I**, Atp6, db/m + vehicle vs db/db + vehicle), <0.0001 (**I**, Atp6, db/db + vehicle vs db/db+K223-pe), <0.0001 (**J**, Nd1, db/m + vehicle vs db/db + vehicle), <0.0001 (**J**, Nd1, db/db + vehicle vs db/db + K223-pe), <0.0001 (**K**, Cytb, db/m + vehicle vs db/db + vehicle), <0.0001 (**K**, Cytb, db/db + vehicle vs db/db + K223-pe), <0.0001 (**L**, Cox1, db/m + vehicle vs db/db + vehicle), <0.0001 (**L**, Cox1, db/db + vehicle vs db/db + K223-pe), <0.0001 (**M**, Atp6, db/m + vehicle vs db/db + vehicle), <0.0001 (**M**, Atp6, db/db + vehicle vs db/db + K223-pe). (**N**,** O**) Flow cytometry (**N**) and quantification analysis (**O**) of mtROS levels in the hippocampal CA1 neurons of db/m, db/db mice treated with vehicle, K223R-pe (scrambled peptide, 5 mg/kg), or K223-pe (5 mg/kg) (*n* = 6 mice per group). *P* values: <0.0001 (db/m + vehicle vs db/db + vehicle), <0.0001 (db/db + vehicle vs db/db + K223-pe). (**P**,** Q**) Flow cytometry (**P**) and quantification analysis (**Q**) of MMP levels in the hippocampal CA1 neurons of db/m, db/db mice treated with vehicle, K223R-pe (scrambled peptide, 5 mg/kg) or K223-pe (5 mg/kg) (*n* = 6 mice per group). *P* values: 0.0008 (db/m + vehicle vs db/db + vehicle), 0.0061 (db/db + vehicle vs db/db + K223-pe). Data were means ± SEM. *p < 0.05, **P < 0.01, ***p < 0.001. Two-way ANOVA followed by Tukey’s test (**D**–**M**, **O**, **Q**). Source data are available online for this figure.

The above results demonstrate that LRPPRC K223 lactylation is a critical pathogenic factor contributing to cognitive impairment in type 2 and type 1 diabetic mice. Given this, targeting this modification offers a potential therapeutic strategy. However, the clinical application of both AAV-mediated LRPPRC K223 mutation and AARS2 knockout remains limited. To overcome these limitations, we designed five short peptides, named K223-peptide 1# through 5#, each containing a homologous sequence around the LRPPRC K223 site to competitively inhibit LRPPRC K223 lactylation, and fused with a cell-penetrating peptide to facilitate crossing the blood–brain barrier (Fig. 5A). Among these peptides, K223-peptide 3# (referred to as K223-pe) exhibited the most potent inhibitory effect on LRPPRC K223 lactylation (Fig. 5B). In addition, we created a scrambled control peptide, K223R-pe, in which the lysine (K) at position 223 was substituted with an arginine (R) residue.

Next, we assessed the blood–brain barrier (BBB) permeability of K223-pe. As shown in Appendix Fig. S9A, at 4 h after injection, FITC-labeled K223-pe crossed the BBB and was detected in the cytoplasm of neurons in the hippocampal CA1 region. The half-life of K223-pe was 59.27 min (Appendix Fig. S9B). To assess the specificity of K223-pe, we examined its effect in vivo on the lactylation of other PPR family members, which share similar sequences, structures, and functions with LRPPRC. The results demonstrated that K223-pe specifically reduced LRPPRC lactylation without affecting the lactylation levels of other PPR family members (Appendix Fig. S9C–N).

**Figure 6:**
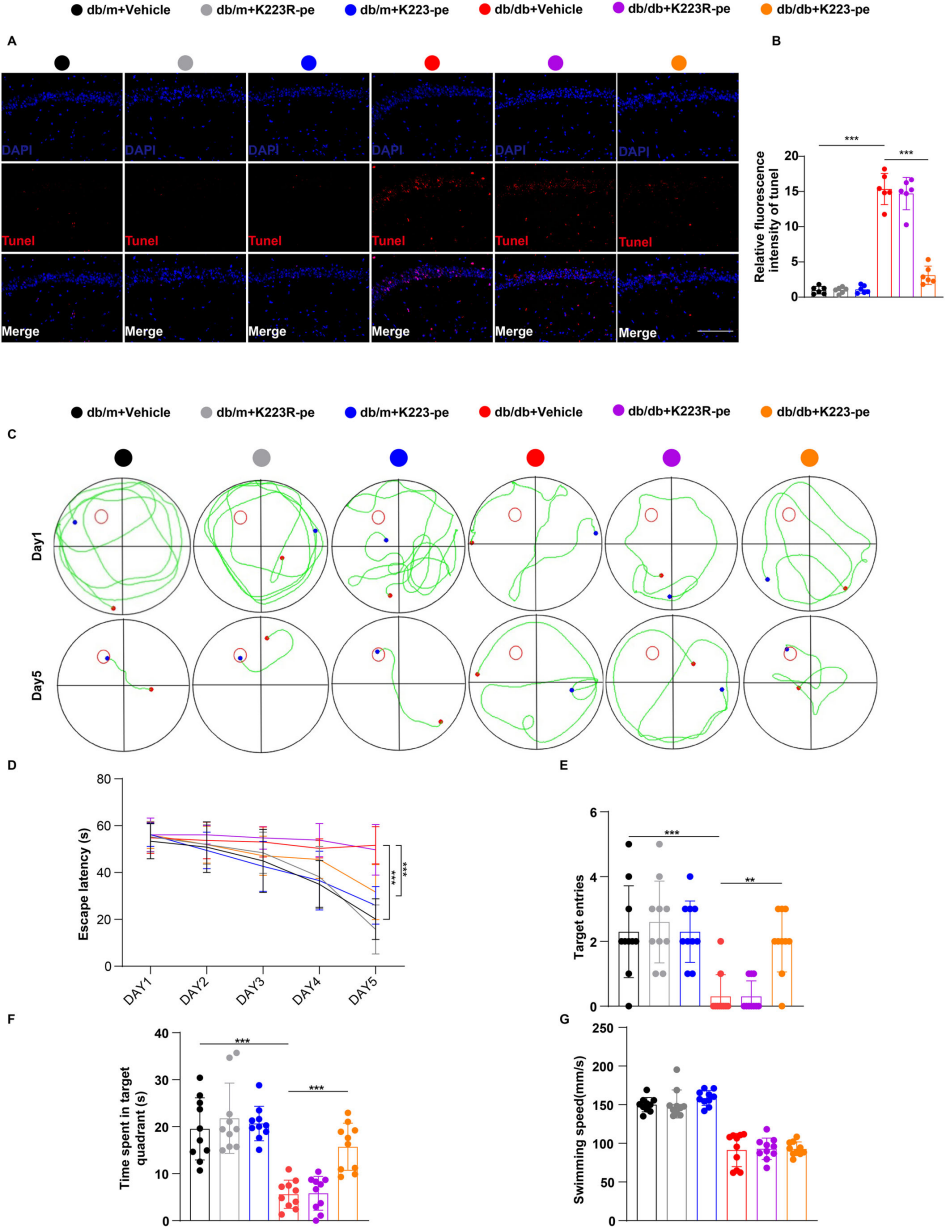
K223-pe reduces neuronal apoptosis and improves cognitive function by regulating the LRPPRC/SLIRP/mitochondrial mRNA signaling pathway. (**A**,** B**) Representative images (**A**) and quantifications (**B**) showing IF staining of Tunel (red) in the hippocampal CA1 neurons of db/m, db/db mice treated with vehicle, K223R-pe (scrambled peptide, 5 mg/kg) or K223-pe (5 mg/kg) (*n* = 6 mice per group). Scale bar: 100 μm. *P* values: <0.0001 (db/m + vehicle vs db/db + vehicle), <0.0001 (db/db + vehicle vs db/db + K223-pe). (**C**–**G**) Representative track images (**C**), escape latency to the platform (**D**), and swimming speed (**G**) during the training trials, target entries (**E**) and time spent in target quadrant (**F**) in the probe trial of Morris water maze of db/m, db/db mice treated with vehicle, K223R-pe (scrambled peptide, 5 mg/kg) or K223-pe (5 mg/kg) (*n* = 10 mice per group). *P* values: <0.0001 (**D**, escape latency, db/m + vehicle vs db/db + vehicle), 0.0004 (**D**, escape latency, db/db + vehicle vs db/db + K223-pe), 0.0006 (**E**, target entries, db/m + vehicle vs db/db+vehicle), 0.0051 (**E**, target entries, db/db + vehicle vs db/db+K223-pe), <0.0001 (**F**, time spent in target quadrant, db/m + vehicle vs db/db + vehicle), 0.0008 (**F**, time spent in target quadrant, db/db + vehicle vs db/db + K223-pe). Data were means ± SEM. *p < 0.05, **P < 0.01, ***p < 0.001. Two-way ANOVA followed by Tukey’s test (**B**, **D**–**G**). Source data are available online for this figure.

Next, we assessed the therapeutic potential of K223-pe. As shown in Fig. 5C–E; Appendix Fig. S10A–C, treatment with K223-pe, but not with K223R-pe, significantly reversed the high glucose-induced LRPPRC K223 lactylation in hippocampal CA1 neurons from both type 1 and type 2 diabetic mice, thereby restoring the LRPPRC-SLIRP interaction. This cascade of events enhanced mitochondrial mRNA stability, leading to improved mitochondrial function (as evidenced by decreased mitochondrial ROS levels and increased membrane potential) (Fig. 5F–Q; Appendix Fig. S10D–O), which subsequently reduced neuronal apoptosis and ameliorated cognitive dysfunction in type 2 and type 1 diabetic mice (Fig. 6A–G; Appendix Fig. S11A–G).

In conclusion, our results suggest that K223-pe enhances mitochondrial function by regulating the LRPPRC/SLIRP/mitochondrial mRNA signaling axis, which ultimately leads to reduced neuronal apoptosis and improved cognitive dysfunction in type 2 and type 1 diabetic mice.

### Elevated plasma LRPPRC K224la levels serve as an independent risk factor for predicting mild cognitive impairment in individuals with diabetes

The above findings highlight the important role of LRPPRC K223 lactylation in the development of cognitive impairment in diabetic mice. However, there are significant challenges in clinically detecting LRPPRC lactylation within hippocampal CA1 neurons of diabetic patients. To enhance the practical utility of clinical detection, we explored whether LRPPRC lactylation levels in peripheral blood could serve as a surrogate marker reflecting its status in hippocampal CA1 neurons. Our ELISA results revealed a strong positive correlation between LRPPRC K223 lactylation levels in plasma and hippocampal CA1 neurons of diabetic mice (Appendix Fig. S12A). Notably, mouse (*Mus musculus*) LRPPRC lysine 223 is homologous to human (*Homo sapiens*) LRPPRC lysine 224. Based on these findings, we further examined whether plasma LRPPRC K224 lactylation could be an independent predictor of mild cognitive impairment (MCI) in diabetic patients. Accordingly, we conducted a longitudinal prospective study with a 4.8-year follow-up period involving 1870 participants aged 60 or older with type 2 diabetes.

**Figure 7:**
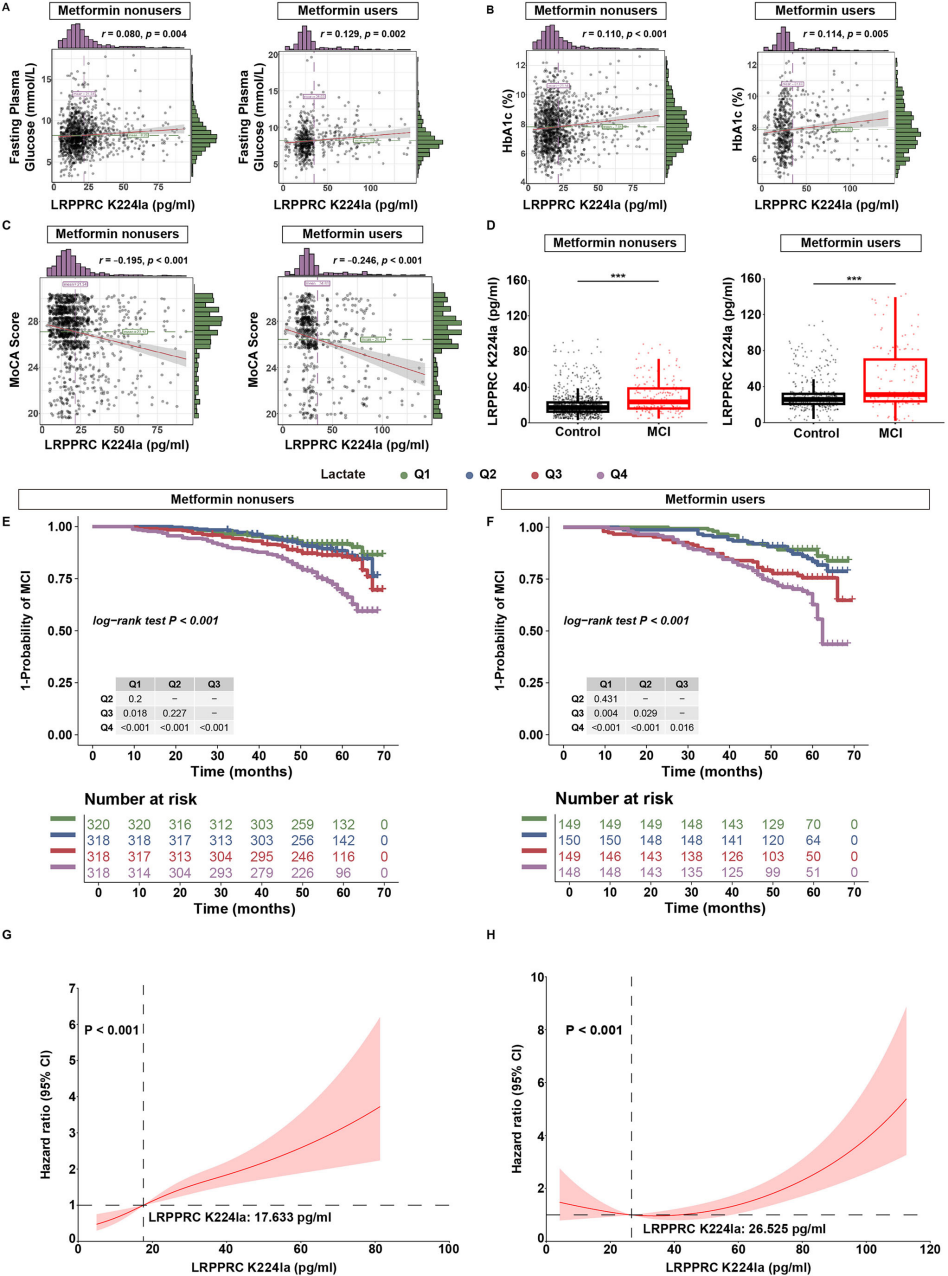
Elevated plasma LRPPRC K224la levels serve as an independent risk factor for predicting mild cognitive impairment in individuals with diabetes. (**A**–**C**) Association of LRPPRC K224la level with fasting plasma glucose (**A**), HbA1c (**B**), and MoCA score (**C**) for metformin nonusers and users. *P* values: 0.004 (**A**, Metformin nonusers), 0.002 (**A**, Metformin users), <0.001 (**B**, Metformin nonusers), 0.005 (**B**, Metformin users), <0.001 (**C**, Metformin nonusers), <0.001 (**C**, Metformin users). (**D**) LRPPRC K224la levels at baseline for metformin nonusers and users among diabetic patients with and without MCI. Boxes represent the first and third quartiles, with the medians indicated by the central lines. Whiskers extend to the lowest and highest values within 1.5 times the interquartile range. (Metformin nonusers: *n* = 1274, Metformin users *n* = 596). (**E**,** F**) Kaplan–Meier curve estimates of the probability of MCI according to four quartiles of LRPPRC K224la level at baseline for metformin nonusers (**E**) and users (**F**). *P* values: <0.001 (**E**, Metformin nonusers), <0.001 (**F**, Metformin users). (**G**,** H**) LRPPRC K224la levels on a continuous scale and risk of incident MCI for metformin nonusers (**G**) and users (**H**). Hazard ratios (HRs; solid line) and 95% confidence intervals (CIs; shading) were derived from multivariable Cox regression using a cubic natural spline. The model was adjusted for age, gender, body mass index (BMI), cigarette smoking, habitual alcohol consumption, leisure-time physical activity, education level, annual income, diabetes therapy, statin use, nonsteroidal anti-inflammatory drug (NSAID) use, duration of diabetes, diabetic nephropathy, cardiovascular disease, systolic blood pressure (SBP), triglycerides (TG), high-density lipoprotein cholesterol (HDL-C), and HbAlc. *P* values: <0.001 (**G**), < 0.001 (**H**). Data were means ± SEM. *p < 0.05, **P < 0.01, ***p < 0.001. Pearson correlation coefficient (**A**–**C**), Two-tailed Student’s unpaired *t*-test analysis (**D**), Wald test (**G**, **H**). Source data are available online for this figure.

Recognizing the potential influence of metformin on plasma lactate levels and subsequent protein lactylation, we stratified the study cohort into two subgroups based on metformin usage. Our analysis revealed a significant elevation in plasma LRPPRC K224la levels among metformin users compared to metformin nonusers (Appendix Fig. S12B). Correlation analyses further demonstrated a significant positive association between plasma LRPPRC K224la levels and both FPG (Fig. 7A) and HbA1c (Fig. 7B), and a significant negative association with MoCA scores (Fig. 7C), independent of metformin use. Diabetic patients who subsequently developed MCI exhibited significantly higher baseline LRPPRC K224la levels (all *P* < 0.001) (Fig. 7D).

The incidence of MCI among older adults with type 2 diabetes during the follow-up period is detailed in Appendix Table S1. To ascertain the independent association between plasma LRPPRC K224la levels and MCI risk, we employed Cox proportional hazards regression modeling. Two models were constructed: Model 1, an unadjusted model, and Model 2, which adjusted for potential confounding variables. Model 1 analysis revealed that participants in the highest quartile of plasma LRPPRC K224la exhibited a significantly elevated risk of developing MCI compared to those in the lowest quartile. This association was observed in both metformin nonusers (HR = 3.7, 95% CI 2.4-5.6) and metformin users (HR = 3.8, 95% CI 2.2–6.3) (Appendix Table S1). Model 2 incorporated adjustments for a range of demographic and clinical factors, including age, gender, BMI, cigarette smoking, habitual alcohol consumption, leisure-time physical activity, education level, annual income, diabetes therapy, statin use, NSAID use, duration of diabetes, diabetic nephropathy, cardiovascular disease, SBP, TG, HDL-C, and HbA1c. Even after adjusting for these potential confounders, the association between the highest quartile of plasma LRPPRC K224la and increased MCI risk persisted. The adjusted HR remained significant for both metformin nonusers (HR = 3.1, 95% CI 2.0–4.7) and metformin users (HR = 2.4, 95% CI 1.4–4.2) (Appendix Table S1). These findings suggest that elevated plasma LRPPRC K224la is a robust and independent predictor of MCI in older adults with type 2 diabetes.

Kaplan–Meier survival curves (Fig. 7E,F) demonstrated that MCI-free survival in individuals with type 2 diabetes decreased across quartiles (Q) of plasma LRPPRC K224la distribution (*P* < 0.001 by log-rank test). To further explore the association between plasma LRPPRC K224la and MCI risk, we employed Cox proportional hazards regression models incorporating restricted cubic splines. After adjusting for a range of potential confounders, the inflection points for MCI risk were identified as 17.633 pg/ml (Fig. 7G) and 26.525 pg/ml (Fig. 7H) in metformin nonusers and users, respectively (both *P* < 0.001). These results suggest that higher plasma LRPPRC K224 lactylation is a strong and independent predictor of MCI in older adults with type 2 diabetes.

## Discussion

**Figure 8:**
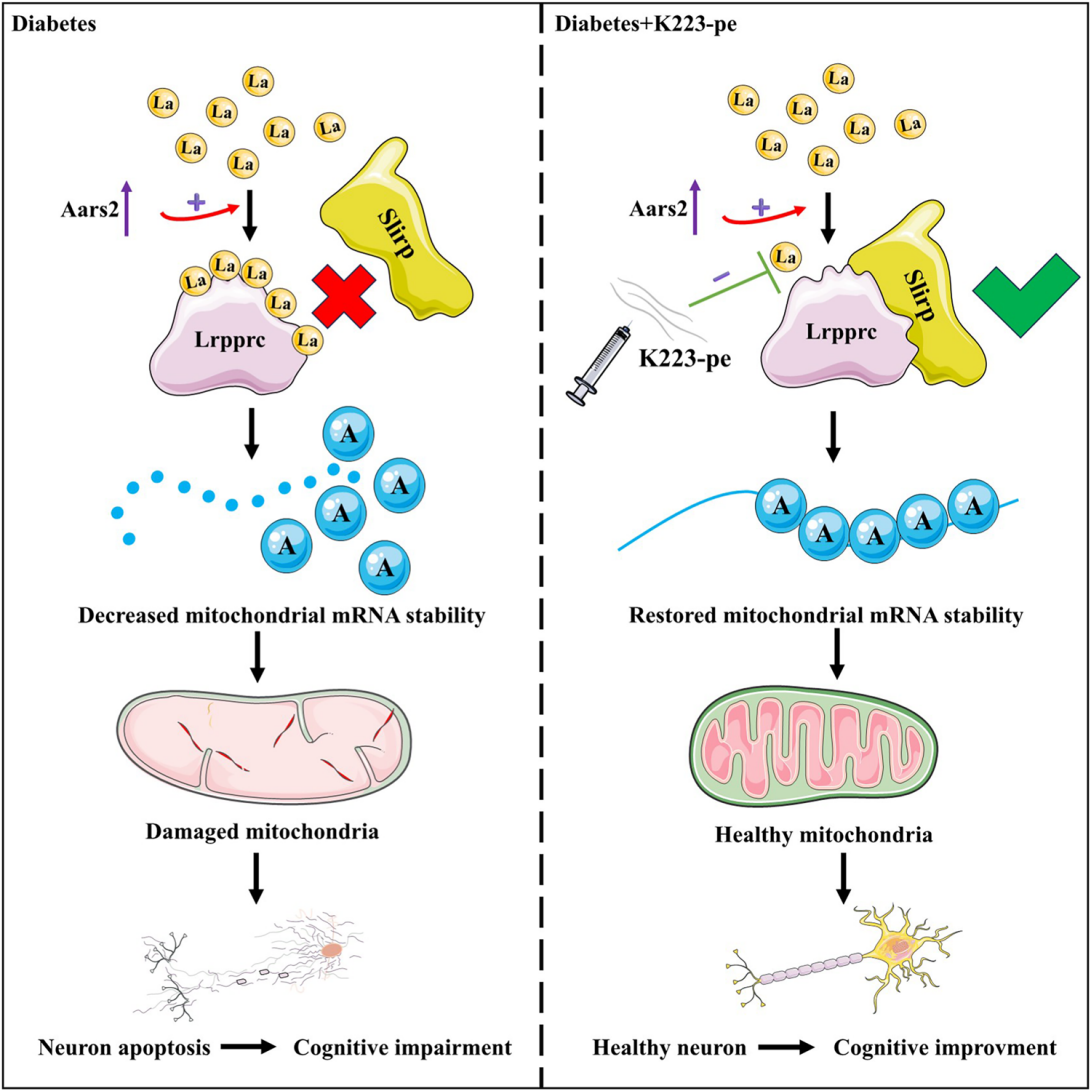
Hyperglycemia impairs cognitive function by inducing mitochondrial damage through lactylation of LRPPRC at K223. Hyperglycemia-induced LRPPRC/SLIRP/mitochondrial mRNA signaling drives neuronal apoptosis and cognitive impairment; K223-pe reverses these effects by blocking this signaling.

In this study, we uncover a new mechanism of high glucose-induced hippocampal neuronal apoptosis and cognitive impairment. The key findings are as follows: (1) Under high glucose conditions, the expression of the lactyltransferase AARS2 is upregulated, leading to the lactylation of LRPPRC, a key mitochondrial protein responsible for stabilizing mitochondrial mRNA, at K223. LRPPRC K223 lactylation reduces its binding affinity with SLIRP, resulting in decreased stability of mitochondrial mRNA in hippocampal neurons and consequently leading to mitochondrial dysfunction, which ultimately causes hippocampal neuron apoptosis and cognitive impairment. (2) A newly developed short peptide, K223-pe, acts as a competitive inhibitor of LRPPRC K223 lactylation and substantially enhances cognitive performance in diabetic mice by regulating the LRPPRC/SLIRP/mitochondrial mRNA signaling pathway. (3) Increased plasma levels of LRPPRC K224 lactylation (homologous to mouse LRPPRC K223) independently predict the risk of mild cognitive impairment in diabetic patients (Fig. 8).

Previous studies have demonstrated that mitochondrial mRNA stability is crucial for maintaining mitochondrial function in neurons (Schweingruber et al, 2025). LRPPRC is an important mitochondrial protein expressed in various cell types throughout the body, including neurons, cardiomyocytes, and skeletal muscle cells (Kim et al, 2012; Liao et al, 2016; Pham et al, 2024). LRPPRC forms a complex with SLIRP, which binds and stabilizes mitochondrial mRNA, promoting its polyadenylation and translation, thereby preserving normal mitochondrial function (Moran et al, 2024; Rubalcava-Gracia et al, 2024; Ruzzenente et al, 2012; Singh et al, 2024). Research by Kim et al showed that LRPPRC deficiency in dopaminergic neurons leads to mitochondrial dysfunction, resulting in neuronal damage and exacerbating the progression of Parkinson’s disease (Kim et al, 2012). Our study further reveals that under high-glucose conditions, lactylation at LRPPRC residue K223, located near the binding sites between LRPPRC and SLIRP, is significantly increased. LRPPRC K223 lactylation reduces the binding affinity between LRPPRC and SLIRP, causing a decrease in mitochondrial mRNA stability and, consequently, mitochondrial dysfunction, ultimately leading to neuronal apoptosis and cognitive impairment. Consistent with these findings, previous studies using indirect calorimetry have reported impaired energy metabolism in both db/db and STZ diabetic mice (Lafferty et al, 2022; López-Soldado et al, 2022). These observations suggest that mitochondrial dysfunction may lead to cognitive impairment through disruption of energy metabolism. Future studies should employ indirect calorimetry to directly validate this mechanism. These findings emphasize the important role of lactylation in regulating LRPPRC function, which in turn modulates mitochondrial health, neuronal survival, and cognitive function.

Lactylation is directly regulated by lactyltransferases (writers) (Zong et al, 2025). Our study demonstrates that, under high-glucose conditions, the expression of lactyltransferase AARS2 is significantly upregulated, resulting in lactylation of LRPPRC at lysine 223. The canonical function of AARS2 is to charge specific tRNAs with their cognate amino acid, alanine (Zhang et al, 2025a). Only recently has it been discovered that AARS2 also exhibits lactyltransferase activity(Li et al, 2024). AARS2 is widely expressed across various cell types throughout the body, including neurons, cardiomyocytes, and ovarian cells, and plays a critical role in the development and progression of leukodystrophy, ataxia, and spasticity (Podmanicky et al, 2024; Zhang et al, 2025a; Zhang et al, 2025b). Previous studies have shown that AARS2 interacts with LRPPRC (Go et al, 2021). Our research further confirms that AARS2 binds to LRPPRC and mediates its lactylation at lysine 223, and hippocampal neuron-specific knockout of AARS2 ameliorates neuronal damage and cognitive impairment in diabetic mice. These findings indicate that AARS2 represents a potential therapeutic target for diabetic cognitive dysfunction.

However, AAV-mediated point mutation of LRPPRC and conditional knockout of AARS2 still face limitations in clinical applications, as AAV therapy in humans may cause severe toxic reactions or even death (2020). To address these limitations, we devised a novel strategy by synthesizing a short peptide containing the LRPPRC K223 site (designated K223-pe), which was combined with a cell-penetrating peptide to facilitate crossing the blood–brain barrier (Qin et al, 2019). Previous studies have indicated that similar peptide-based strategies can alleviate Alzheimer’s disease and cancer by inhibiting acetylation and glycosylation of target proteins (Zhou et al, 2022; Zhu et al, 2023). Our peptide K223-pe competitively blocks lactylation at LRPPRC K223 without affecting lactylation of other PPR family proteins. By modulating the LRPPRC/SLIRP/mitochondrial mRNA signaling cascade, K223-pe effectively improves cognitive deficits in diabetic mice, providing a novel therapeutic avenue for diabetic cognitive impairment.

Cognitive impairment is often difficult to reverse once established, underscoring the critical need for prevention and early screening to control its progression (Blass et al, 2025; Frisoni et al, 2023; Sasannejad et al, 2019). Although elevated lactylation of LRPPRC in hippocampal CA1 neurons is a key contributor to cognitive decline, it remains a significant challenge in clinical settings to quickly and easily evaluate LRPPRC lactylation levels in hippocampal neurons of diabetic patients. Encouragingly, our findings demonstrate a significant positive correlation between the LRPPRC lactylation levels in peripheral blood and in hippocampal CA1 neurons. Furthermore, through a large-scale prospective cohort study, we confirmed that the lactylation level at LRPPRC K224 (the human counterpart of mouse LRPPRC K223) in peripheral blood can independently predict the onset and progression of cognitive impairment in patients with diabetes. This discovery offers a promising, accessible method for large-scale screening of individuals at high risk for diabetes-associated cognitive impairment. Notably, metformin users exhibited elevated plasma LRPPRC K224 lactylation. While metformin is highly effective in glycemic control and remains a first-line treatment for type 2 diabetes, several studies have reported cognitive side effects associated with metformin use (Moore et al, 2013; Porter et al, 2019). Our finding that metformin users exhibit elevated plasma LRPPRC K224 lactylation suggests a potential mechanism underlying these cognitive side effects.

In conclusion, we identify a mechanistic connection between high glucose-induced lactylation and the LRPPRC/SLIRP/mitochondrial mRNA signaling cascade in regulating mitochondrial function and apoptosis in hippocampal neurons. Our research also emphasizes the essential role of lactylation in impairing mitochondrial protein function under high glucose conditions, uncovers a novel neuroprotective mechanism of short peptides in type 2 and type 1 diabetic mice, and identifies a new peripheral blood biomarker for predicting cognitive impairment in diabetic patients.

### Limitation of the Study

First, LRPPRC is expressed across multiple organs (Lei et al, 2016; Ruzzenente et al, 2012; Sasarman et al, 2015), indicating that LRPPRC K223la detected in plasma may derive from diverse tissue sources. Notably, in mice experiments, we found a significant positive correlation between LRPPRC K223la in plasma and hippocampal CA1 neurons, suggesting that plasma LRPPRC K223la might be partially derived from hippocampal CA1 neurons. However, the inability to procure tissue specimens in patients precludes direct experimental verification of human plasma LRPPRC K224la sources, representing a limitation of this study that warrants future investigation. Second, because diet-induced obese (DIO) and non-obese diabetic (NOD) mice more closely recapitulate the human diabetic situation, the underlying mechanisms should be validated in DIO and NOD mouse models in future studies to improve translational relevance.

## Methods

**Table:** Reagents and tools table

Reagent/resource	Reference or source	Identifier or catalog number
**Experimental models**		
Primary murine hippocampal neurons	This Paper	N/A
Mouse: BKS.Cg-Dock7^m^ +/+ Lepr^db^/J	Jackson Laboratory	Cat# 000642
Mouse: Aars2 ^fl/fl^	GemPharmatech Co. Ltd	Cat# T008338
Mouse: C57BL/6	Changsha Hunan Silaike Jingda Laboratory Animal Co., Ltd.	N/A
**Antibodies**		
Rabbit polyclonal anti-Bax (1:1000)	Beyotime	Cat# AF0057
Rabbit polyclonal anti-Bcl2 (1:1000)	Beyotime	Cat# AF0060
Rabbit polyclonal anti-Cleaved-caspase3 (1:2000)	Proteintech	Cat# 25128-1-AP
Rabbit polyclonal anti-β-actin (1:10000)	Proteintech	Cat# 20536-1-AP
Rabbit polyclonal anti-Lrpprc (1:10000)	Proteintech	Cat# 21175-1-AP
Rabbit polyclonal anti-Aars2(1:4000)	Proteintech	Cat# 22696-1-AP
Rabbit polyclonal anti-Slirp(1:4000)	Proteintech	Cat# 26006-1-AP
Rabbit polyclonal anti-Polrmt(1:1000)	ABclonal Technology	Cat# A15605
Rabbit polyclonal anti-Mrps27(1:1000)	Proteintech	Cat# 17280-1-AP
Rabbit polyclonal anti-Mrpp3(1:1000)	Proteintech	Cat# 20959-1-AP
Rabbit polyclonal anti-Ptcd1(1:1000)	ABclonal	Cat# A16219
Rabbit polyclonal anti-Ptcd2(1:5000)	Invitrogen	Cat# PA5-113165
Rabbit polyclonal anti-Ptcd3(1:1000)	Proteintech	Cat# 25158-1-AP
Pan Lactic acid-Lysine Rabbit mAb (1:1000)	ABclonal Technology	Cat# A23004
Rabbit monoclonal anti-IgG	Abcam	Cat# ab172730
HRP-conjugated Affinipure Goat Anti-Mouse IgG (1:5000)	Proteintech	Cat# SA00001-1
HRP-conjugated Affinipure Goat Anti-Rabbit IgG (1:5000)	Proteintech	Cat# SA00001-2
**Oligonucleotides and other sequence-based reagents**		
Lrpprc shRNA targeting sequence: 5’- GCAAGTAGTACCTTTGCTCAA-3’	GeneChem	N/A
Aars2 siRNA targeting sequence: 5’- CTCATCATACGTTTTTCGAGATG-3’	GenePharma	N/A
Aars1 siRNA targeting sequence: 5’-CTGGTCTTTTGGGGACTATTTCA-3’	GenePharma	N/A
Cbp siRNA targeting sequence: 5’-ATGGAGAATTAAGCCTTTTAAAC-3’	GenePharma	N/A
Ep300 siRNA targeting sequence: 5’- AGGGATAATGCCCAATCAAGTCA-3’	GenePharma	N/A
Kat2a siRNA targeting sequence: 5’- CACCAAACAAGTCTATTTCTACC-3’	GenePharma	N/A
Tip60 siRNA targeting sequence: (5’-TGCGAATTTTGCCTCAAATATGG-3’	GenePharma	N/A
Kat8 siRNA targeting sequence: (5’- TTCACTATGTGGGCTTTAACAGG-3’	GenePharma	N/A
Hbo1 siRNA targeting sequence: (5’- ACCGAAGATTCCGATTTTTCTAC-3’	GenePharma	N/A
Atat1 siRNA targeting sequence: (5’- CAGCAAATCATGACTATTGTAGA-3’)	GenePharma	N/A
Mouse beta-actin qPCR F: 5’-CGTTGACATCCGTAAAGACCTC-3’ R: 5’-CCACCGATCCACACAGAGTAC-3'	Sangong Biotech	N/A
Mouse Cox1 qPCR F: 5’-TCAACCTTGTCAACACAGCCTCAC-3’ R: 5’- GGCACACGGAAGGAAACATAGGG-3’	Sangong Biotech	N/A
Mouse Atp6 qPCR F: 5’- GCTCACTTGCCCACTTCCTTCC-3’ R: 5’- GCCGGACTGCTAATGCCATTGG-3’	Sangong Biotech	N/A
Mouse Nd1 qPCR F: 5’- TCCGAGCATCTTATCCACGC-3’R: 5’-GTATGGTGGTACTCCCGCTG-3’	Sangong Biotech	N/A
Mouse Cytb qPCR F: 5’-CTAATCCACTAAACACCCCACC-3’ R: 5’-GGCTTCGTTGCTTTGAGGTAT-3’	Sangong Biotech	N/A
**Chemicals, Enzymes and other reagents**		
B-27 (50×)	Thermo Fisher Scientific	Cat# 17504044
BeyoMag™ Oligo (dT) 25 Magnetic Beads	Beyotime	Cat#R0075
Neurobasal medium	Gibco	Cat# 21103049
Glutamine	Gibco, Invitrogen	Cat# 25030081
DMEM	Thermo Fisher Scientific	Cat# 11995065
Trypsin Digestion solutions,0.25% (without phenol red)	Solarbio	Cat# T1350
Penicillin-Streptomycin Liquid	Solarbio	Cat# P1400
Poly-L-lysine	Sigma-Aldrich	Cat# P4707
TRLZOl^TM^ Reagent	Invitrogen	Cat# 15596026
FastKing gDNA Dispelling RT SuperMix	TIANGEN	Cat# KR118
MonAmp^TM^ SYBR Green qPCR Mix	Monad	Cat# MQ10301S
4% Paraformaldehyde	Solarbio	Cat# P1110
D-Glucose,anhydrous	Solarbio	Cat# G8150
Quick Antigen Retrieval Solution for Frozen Sections	Beyotime	Cat# P0090
QuickBlock™ Primary Antibody Dilution Buffer for Immunol Staining	Beyotime	Cat# P0262
QuickBlock™ Secondary Antibody Dilution Buffer for Immunofluorescence	Beyotime	Cat# P0265
QuickBlock™ Blocking Buffer for Immunol Staining	Beyotime	Cat# P0260
DAPI solution	Solarbio	Cat# C0060
QuickBlock™ Blocking Buffer for Western Blot	Beyotime	Cat# P0252
QuickBlock™ Secondary Antibody Dilution Buffer for Western Blot	Beyotime	Cat# P0258
QuickBlock™ Primary Antibody Dilution Buffer for Western Blot	Beyotime	Cat# P0256
ECL reagent	Shanghai Life-iLab Biotech Co., Ltd	Cat# AP34L024
MitoSOX™ Red mitochondrial superoxide indicator	Thermo Fisher Scientific	Cat# M36008
Lipofectamine™ RNAiMAX Transfection Reagent	Invitrogen	Cat# 13778030
Lipofectamine™ 3000 Transfection Reagent	Thermo Fisher Scientific	Cat# L3000001
OSMI-1	Sigma-Aldrich	Cat# SML1621
Streptozotocin	MCE	Cat# HY-13753
**Software**		
GraphPad Prism 9.0	GraphPad	https://www.graphpad.com/scientific-software/prism/
IBM SPSS Statistics 29	IBM	https://www.ibm.com/support/pages/downloading-ibm-spss-statistics-29
Gene Expression Omnibus database	GEO database	https://www.ncbi.nlm.nih.gov/geo/query/acc.cgi?acc=GSE201644
FlowJo v10	FlowJo	https://www.flowjo.com/
**Other**		
Adult Brain Dissociation Kit, mouse and rat	Miltenyi Biotec	Cat# 130-107-677
Adult Neuron Isolation Kit, mouse	Miltenyi Biotec	Cat# 130-126-603
Pierce® Classic Magnetic IP/CoIP Kit	Thermo Fisher Scientific	Cat# 88804
Fastking RT Kit (with gDNA)	TIANGEN	Cat# KR116-02
TUNEL Apoptosis Assay Kit	Beyotime	Cat# C1090
PCR Purification Kit	QIAGEN	Cat# 28104
QuikChange XL site-directed mutagenesis kit	Agilent Technologies	Cat# 200516
BCA Protein Assay Kit	Beyotime	Cat# P0011

### Human subjects

Data were sourced from the Guangxi Diabetes and Metabolic Disorders (GDMD) Study, a longitudinal observational investigation conducted in Guilin, China, aiming to explore the etiology, complications, and associated conditions of type 2 diabetes and metabolic syndrome. From 2013 to 2015, a total of 2292 elderly individuals aged between 60 and 80 years with a confirmed diagnosis of type 2 diabetes were recruited from the Medical Examination Center and the Endocrinology Outpatient Department of the Affiliated Hospital of Guilin Medical University. Exclusion criteria encompassed mild cognitive impairment (MCI), dementia, acute inflammatory or diabetic complications, malignancies, hypothyroidism, autoimmune diseases, hypertensive crises, organ failures (including respiratory, cardiac, hepatic, and renal), neurological disorders related to dementia, severe hypoglycemia, sensory impairments (hearing or vision), psychiatric illnesses, history of substance abuse, and incomplete datasets. Participants underwent annual MCI screenings until 2019. After excluding 422 participants due to mortality or loss to follow-up, 1870 subjects were included in the final analysis. The study received ethical approval from the Ethical Committee of the Affiliated Hospital of Guilin Medical University and adhered to the Declaration of Helsinki, with all participants providing written informed consent. The trial is registered with the Chinese Clinical Trial Registry (ChiCTR-EPC-14005273).

### Mice

BKS.Cg-Dock7^m^ +/+ Lepr ^db^/J (db/m) mice were obtained from Jackson Laboratory, Aars2 ^fl/fl^ mice were sourced from GemPharmatech Co., Ltd., and C57BL/6 mice were supplied by Changsha Hunan Silaike Jingda Laboratory Animal Co., Ltd. The db/db mice were generated by self-breeding db/m mice, as db/db mice are sterile. Aars2-floxed (Aars2 ^fl/fl^) mice were created by inserting two flox sequences (ATAACTTCGTATAGCATACATTATACGAAGTTAT) into the terminals of Aars2 exons 1–5. Genomic DNA extracted from tail tips was used for genotyping, with the following primers utilized for Aars2 ^fl/fl^ mice: F- AAGCAACAGGAGAAGAGGTGTTGG; R-TAACCATCTCAGCAGCCCAGCAT. Aars2 ^fl/+^ mice were intercrossed to obtain Aars2 ^fl/fl^ mice. Aars2 ^fl/fl^ mice and db/m mice were crossed to produce Aars2 ^fl/+^ db/m mice, and Aars2 ^fl/fl^ db/db mice were obtained by further breeding between Aars2 ^fl/+^ db/m heterozygous mice. All mice were weaned at postnatal day 21 and housed in individually ventilated cages under standard laboratory conditions. Animals were maintained on a 12-h light/dark cycle (lights on at 07:00, off at 19:00) at a controlled temperature of 23–24 °C with ad libitum access to standard laboratory chow and water.

Type 1 diabetes was induced in mice using streptozotocin (STZ) (MCE, Cat# HY-13753) in accordance with the Low-Dose Streptozotocin Induction Protocol (Wu et al, 2019). Briefly, 8-week-old C57BL/6 mice received intraperitoneal injections of STZ (50 mg/kg dissolved in citrate buffer, pH 4.5) once daily for five consecutive days. Control mice were injected with an equivalent volume of citrate buffer (vehicle). Blood glucose levels were measured 2 weeks post-injection, and mice with levels exceeding 16.7 mmol/L were classified as diabetic. Experiments were carried out three months after diabetes induction. To evaluate the potential effects of short peptides on cognitive function in diabetic conditions, STZ-treated diabetic mice and db/db mice were administered short peptides (5 mg/kg/day) through daily intraperitoneal injections for 4 weeks. Age-matched controls were given an equivalent volume of saline (vehicle). The total number of mice used in each experiment is detailed in the figure legends.

### Cell isolation and culture

Primary hippocampal neurons were isolated from embryonic (E16–18) mouse brains. In summary, the hippocampus was carefully excised and cut into 1 mm³ pieces using a blade. The fragments were subjected to enzymatic dissociation with 0.25% trypsin (Solarbio, Cat# T1350) at 37 °C for 10 min. To terminate the digestion process, 10% fetal bovine serum (FBS) was added. The resulting cell suspension was centrifuged at 1000 g for 5 min, and the cell pellet was rinsed with cold PBS. Cells were then resuspended in Neurobasal A medium containing 2% B-27 (Thermo Fisher Scientific, Cat# 17504044) and 0.5 mM glutamine (Gibco™, Cat# 25030081). Neurons were plated onto six-well plates pre-coated with poly-L-lysine (Sigma-Aldrich, Cat# P4707) and maintained at 37 °C in a humidified 5% CO₂ atmosphere.

For in vitro studies using primary hippocampal neurons, high-glucose conditions were established by raising glucose concentrations to 25 mM above ambient levels. This concentration was determined through preliminary experiments, which identified a clear dose-dependent relationship between glucose levels and their effects on hippocampal neurons. Additionally, this glucose level was selected to simulate moderate-to-severe type 2 diabetes, equivalent to a blood glucose range of ~450 mg/dL. To rule out confounding osmotic effects associated with elevated glucose, equimolar mannitol was used as an osmotic control.

Hippocampal CA1 neurons were isolated using a previously established protocol (Guo et al, 2012). In brief, adult mice of designated ages were deeply anesthetized with isoflurane, followed by transcardial perfusion with ice-cold saline. The whole brain was rapidly extracted, rinsed in prechilled phosphate-buffered saline (PBS), and sectioned into 400-μm coronal slices using a Tissue Chopper. Hippocampal-containing sections (4–6 slices) were collected with a sterile, damp swab and placed in a petri dish containing 5 mL of 1X HBSS supplemented with 30 mM glucose, 2 mM Hepes, and 26 mM NaHCO₃. Under a dissecting microscope, the CA1 region of the hippocampus was carefully isolated. The dissected hippocampal CA1 tissue was then processed to generate a single-cell suspension through a combination of mechanical dissociation and enzymatic digestion using the Adult Brain Dissociation Kit (Miltenyi Biotec, Cat# 130-107-677). Debris and erythrocytes were eliminated, and the remaining cell pellet was resuspended in PBS with 0.5% BSA. Neurons were enriched by negative selection using the Adult Neuron Isolation Kit (Miltenyi Biotec, Cat# 130-126-603) according to the manufacturer’s protocol. Briefly, non-neuronal cells, including microglia, astrocytes, oligodendrocytes, fibroblasts, and endothelial cells, were labeled with a biotin-conjugated antibody cocktail at 4 °C for 5 min. After washing with PBS containing 0.5% BSA, the cells were pelleted, resuspended, and incubated with anti-biotin microbeads at 4 °C for 10 min. The labeled cells were separated using an LS column in the presence of a magnetic field, yielding a purified neuronal population. Flow cytometry analysis (Appendix Fig. S2H) confirmed that the isolated hippocampal CA1 neurons achieved a purity level exceeding 95%, consistent with the manufacturer’s specifications.

### Stereotaxic injection

Mice aged 4 months from various experimental groups were anesthetized with isoflurane and carefully positioned in a stereotaxic frame featuring a digital position monitor and syringe pump. Viral vectors were bilaterally delivered into the CA1 region of the hippocampus using a Nanoject III (Drummond) at target coordinates: x = 1.0 mm to the midline (left or right), y = −1.9 mm posterior to the bregma, and z = 1.5 mm below the cortical surface. Injections were performed at a controlled rate of 0.05 μL/min, with the needle remaining in place for an additional 5 min post-injection to avoid viral efflux. For AARS2-floxed mice, 0.3 μL of AAV-CAMKII control or AAV-CAMKII-Cre was microinjected into the dorsal CA1 to achieve selective deletion of Aars1 in hippocampal CA1 neurons. In addition, 0.3 μL of either AAV-CAMKII control, AAV-CAMKII-Flag-LRPPRC-WT, or AAV-CAMKII-Flag-LRPPRC-K223R was administered bilaterally in the hippocampal CA1 region of db/db, STZ-induced diabetic mice and their respective controls to specifically express either the wild-type or K223R mutant of LRPPRC within CA1 neurons of the hippocampus.

### Gene silencing and overexpression

Primary hippocampal neurons were stably transduced with lentiviral shRNA targeting LRPPRC (vector backbone: hU6-MCS-CBh-gcGFP-IRES-puromycin, detailed information available at GENECHEM, https://www.genechem.com.cn/index/supports/zaiti_info.html?id=83) to knock down endogenous LRPPRC expression. The knockdown efficiency was validated via immunoblotting. The shRNA sequences used for LRPPRC knockdown was 5’- GCAAGTAGTACCTTTGCTCAA-3’.

For targeted silencing of Aars2, Aars1, Cbp, Ep300, Kat2a, Tip60, Kat8, Hbo1, and Atat1, primary hippocampal neurons were transfected with either targeting siRNAs or a non-targeting control siRNA at a final concentration of 100 nM. Transfection was performed using Lipofectamine RNAiMAX (Invitrogen, Cat# 13778030) according to the manufacturer’s protocol. The siRNA sequence employed were siAars2 (5’- CTCATCATACGTTTTTCGAGATG-3’), siAars1 (5’-CTGGTCTTTTGGGGACTATTTCA-3’), siCbp (5’-ATGGAGAATTAAGCCTTTTAAAC-3’), siEp300 (5’- AGGGATAATGCCCAATCAAGTCA-3’), siKat2a (5’- CACCAAACAAGTCTATTTCTACC-3’), siTip60 (5’-TGCGAATTTTGCCTCAAATATGG-3’), siKat8 (5’- TTCACTATGTGGGCTTTAACAGG-3’), siHbo1 (5’- ACCGAAGATTCCGATTTTTCTAC-3’), siAtat1 (5’- CAGCAAATCATGACTATTGTAGA-3’). Knockdown efficiency was evaluated by immunoblotting 48 h following transfection.

To induce overexpression, neurons were transfected with a plasmid encoding AARS2 using Lipofectamine^TM^ 3000 (Invitrogen, Cat# L3000001) at a ratio of 2 µL of reagent per microgram of DNA. The plasmid was constructed in the GV657-CMV-FLAG-GFP-Puromycin vector backbone supplied by GeneChem (Shanghai, China). Successful protein overexpression was confirmed by Western blot analysis 48 h post-transfection.

To generate the LRPPRC mutant plasmid (K223R and K223T), site-directed mutagenesis was performed using the QuikChange XL Kit (Agilent Technologies, CA, USA, Cat# 200516).

### Immunoblot analysis

Hippocampal neurons were lysed in RIPA buffer supplemented with 1% protease and phosphatase inhibitors on ice for 30 min. The lysate was centrifuged at 12,000 × *g* for 15 min at 4 °C, and the supernatant was carefully collected. Protein concentrations were quantified using a BCA Protein Assay Kit and adjusted to a final concentration of 10 μg/μL. Proteins were resolved using 10% SDS-PAGE and subsequently transferred onto PVDF membranes. After the transfer, membranes were blocked at room temperature for 15 min using QuickBlock^TM^ Blocking Buffer (Beyotime, Cat# P0252) on a shaker. Following blocking, the membranes were rinsed three times with TBST (Tris-buffered saline with 0.1% Tween-20) and incubated overnight at 4 °C with indicated primary antibodies diluted in Primary Antibody Dilution Buffer (Beyotime, Cat# P0256): anti-Bax antibody (1:1000, Beyotime, Cat# AF0057, RRID: AB_2923045), anti-Bcl2 antibody (1:1000, Beyotime, Cat# AF0060, RRID: AB_2923046), anti-Cleaved-caspase3 antibody (1:2000, Proteintech, Cat# 25128-1-AP; RRID: AB_3073913), anti-β-actin antibody (1:10,000, Proteintech, Cat# 20536-1-AP; RRID: AB_10700003), anti-LRPPRC antibody (1:10,000, Proteintech, Cat# 21175-1-AP; RRID: AB_10733879), anti-AARS2 antibody (1:4000, Proteintech, Cat# 22696-1-AP; RRID: AB_11182926), anti-SLIRP antibody (1:4000, Proteintech, Cat# 26006-1-AP; RRID: AB_2880332), anti-Polrmt-antibody (1:1000, ABclonal, Cat# A15605, RRID: AB_2763011), anti-Mrps27-antibody (1:1000, Proteintech, Cat# 17280-1-AP, RRID: AB_2180510), anti-Mrpp3-antibody (1:1000, Proteintech, Cat# 20959-1-AP, RRID: AB_10860109), anti-Ptcd1-antibody (1:1000, ABclonal, Cat# A16219, RRID: AB_2763673), anti-Ptcd2-antibody (1:5000, Invitrogen, Cat# PA5-113165, RRID:AB_2867899), anti-Ptcd3-antibody (1:1000, Proteintech, Cat# 25158-1-AP, RRID: AB_2879931), anti-Nd1-antibody (1:5000, Proteintech, Cat# 19703-1-AP, RRID: AB_10637853), anti-Cytb-antibody (1:2000, Proteintech, Cat# 55090-1-AP, RRID: AB_2881266), anti-Cox1-antibody (1:1000, Abcam, Cat# ab203912, RRID: AB_2801537), anti-Atp6-antibody (1:1000, Proteintech, Cat# 55313-1-AP, RRID: AB_2881305), and anti-Pan Lactic acid-Lysine antibody (1:1000, ABclonal, Cat# A23004). The membranes were rinsed three times with TBST before being treated with a horseradish peroxidase-labeled secondary antibody at a 1:5000 dilution for 1 h. Protein signals were visualized using the ECL Chemiluminescent Substrate Reagent Kit and captured with the ChemiDoc XRS+ System (Bio-Rad). Quantification of the detected signals was performed using Image Lab software.

To prepare a site-specific antibody targeting mouse LRPPRC lactylated at K223, a synthetic polypeptide containing a lactyl group on lysine 223 (ILGFMK(lac)TKDLP) was utilized. Polyclonal antibodies were generated by immunizing New Zealand White rabbits with 0.5 mL of peptide (1 mg/mL) mixed with an equal volume of complete adjuvant, followed by three weekly booster injections, each containing 0.5 ml of the polypeptide (1 mg/ml) plus incomplete adjuvant. Antiserum was harvested via carotid artery 7 days post final immunization and affinity-purified using a Protein A/G column. The human LRPPRC K224 is homologous to mouse LRPPRC K223, and the antibody prepared by the above method was also used to detect human LRPPRC lactylated at K224.

### Immunoprecipitation (IP) and CoIP analysis

Immunoprecipitation (IP) and co-immunoprecipitation (CoIP) of the target proteins were carried out using the Pierce^TM^ Magnetic IP/CoIP kit (Thermo Fisher Scientific, Cat# 88804). After treatment, hippocampal neuron cells were lysed on ice for 5 min in IP lysis buffer supplemented with protease inhibitors, followed by centrifugation at 13,000×*g* for 10 min at 4 °C. The supernatant was collected and incubated with specific antibodies overnight at 4 °C on a shaker. Normal IgG served as the immunoprecipitation control. Antibody targeting LRPPRC was used at a concentration of 10 μg per 1 mg of total protein. The antibody-bound proteins were then mixed with prewashed protein A/G magnetic beads and incubated for 1 h at room temperature. After thorough washing, the bound proteins were eluted from the beads and analyzed via gel electrophoresis and immunoblotting with antibodies against AARS2, LRPPRC, SLIRP, and lactyl lysine.

### Mitochondrial RNA extraction and RT-qPCR

Mitochondria were isolated from primary hippocampal neurons or hippocampal CA1 neurons isolated from different groups of mice using a procedure as described by the manufacturer (Cell Mitochondria Isolation Kit, Beyotime). The BeyoMag Oligo (dT)25 Magnetic Beads company protocol was used in order to extract poly(A) RNA. Reverse transcription (RT) was performed with the FastKing RT Kit (TIANGEN, Cat# KR116-02). The reaction mix (20 μL total volume) consisted of 1 μL of diluted cDNA, 0.5 μL of primers (Nd1: F: 5’- TCCGAGCATCTTATCCACGC-3’, R: 5’-GTATGGTGGTACTCCCGCTG-3’; Cytb: F: 5’-CTAATCCACTAAACACCCCACC-3’, R: 5’-GGCTTCGTTGCTTTGAGGTAT-3’; Cox1: F: 5’-TCAACCTTGTCAACACAGCCTCAC-3’, R: 5’- GGCACACGGAAGGAAACATAGGG-3’; Atp6: F: 5’- GCTCACTTGCCCACTTCCTTCC-3’, R: 5’- GCCGGACTGCTAATGCCATTGG-3’; β-actin: F: 5’-CGTTGACATCCGTAAAGACCTC-3’, R: 5’-CCACCGATCCACACAGAGTAC-3’), 10 μL of SYBR mix (Monad, Cat# MQ10301S), and 8 μL of nuclease-free water. RT-qPCR was conducted using the Bio-Rad CFX96™ Real-Time PCR Detection System to measure gene expression. Relative expression levels of the target genes were normalized to β-actin and calculated using the 2^–ΔΔCT^ method.

### ELISA

The levels of LRPPRC K224la in plasma were quantified using ELISA. The standard was recombinant human LRPPRC lactylated at K224. Ninety-six-well plates were prepared by coating with anti-LRPPRC antibody (Proteintech, Cat No. 21175-1-AP) overnight at 4 °C. Following three washes with PBST, wells were blocked by incubating with blocking buffer for 2 h at 37 °C. After an additional three washes with PBST, plasma samples were added to the wells. The plates were incubated for 2 h at 37 °C, and then washed three more times with PBST. Subsequently, the detection antibody, anti-LRPPRC K224la antibody, was added to each well, followed by a 2-h incubation at 37 °C. After another wash, a horseradish peroxidase (HRP)-conjugated goat anti-rabbit secondary antibody (Proteintech, Cat# SA00001-2) was applied for 1 h at 37 °C. The plates were then washed with PBST. TMB Microwell Peroxidase Substrate was added, and color was allowed to develop at room temperature for 30 min. The stop solution was then applied, and absorbance was measured at 450 nm using a microplate reader.

### Measurement of mitochondrial reactive oxygen species (mtROS)

Mitochondrial reactive oxygen species (mtROS) levels in primary hippocampal neurons were measured using MitoSOX™ Red (Thermo Fisher Scientific, Cat# M36008). Neurons were incubated with a 2.5 μM working solution of MitoSOX™ for 10 min at 37 °C, followed by rinsing with HBSS to remove any residual MitoSOX. The fluorescence intensity was then assessed by flow cytometry, and the resulting data were analyzed with FlowJo (v10) software.

### Measurement of oxygen consumption rate (OCR)

OCR was assessed using a Seahorse XF24 Extracellular Flux Analyzer (Agilent Technologies). Primary hippocampal neurons were plated onto Seahorse XF24 microplates at a density of 75,000 cells per well and exposed to high-glucose conditions for 72 h before the assay. After recording baseline respiration, mitochondrial function was evaluated by injections of oligomycin, p-trifluoromethoxy carbonyl cyanide phenylhydrazone (FCCP), and rotenone plus antimycin A. OCR was measured three times at 10-minute intervals. The resulting OCR data were processed through Seahorse XF24 analysis software, and then normalized to cell count.

### TUNEL staining

Tissue sections were subjected to TUNEL staining with the Beyotime TUNEL Apoptosis Assay Kit (Cat# C1088). The sections were first permeabilized for 5 min using 0.1% Triton X-100, then exposed to the TdT reaction mix for a 1-h period at 37 °C. After PBS rinsing, nuclear counterstaining was carried out using 5 µg/ml DAPI for 10 min at room temperature, and fluorescence images were then captured using a fluorescence microscope.

### Morris water maze

The Morris water maze (MWM) test was performed in a circular pool with a diameter of 120 cm and a height of 50 cm, filled with opaque water maintained at a temperature of 20 ± 1 °C. A 10 cm diameter platform was submerged 1 cm beneath the water surface. The pool was divided into four equal quadrants, and mice were released from four different starting points, facing the pool’s walls, to explore the platform for 60 s. If a mouse did not find the platform within this time, it was guided to the platform by the experimenter. Each mouse underwent four trials per day over a five-day period, following a single day of habituation. After the fifth day, the platform was removed, and a probe trial was performed. Behavioral data, including escape latency, number of platform crossings, time spent in the target quadrant, and swimming trajectories, were recorded using Video Tracker software (Anymaze).

### Peptide synthesis

The peptides used in this study were synthesized by Guoping Pharmaceutic Inc. and purified through high-performance liquid chromatography to ensure a purity level above 98%, making them suitable for both in vitro and in vivo experiments. For in vitro studies, peptides were prepared as 20 mM stock solutions in phosphate-buffered saline (PBS). For in vivo applications, K223-pe and K223R-pe were dissolved in PBS, maintained on ice until injection, and brought to room temperature just before administration.

### Half-life measurement of K223-pe

C57BL/6 mice (*n* = 6) were intravenously injected with 5 mg/kg of K223-pe. Plasma levels of K223-pe were quantified using ELISA following injection. The plasma elimination half-life (t₁/₂) of K223-pe was determined using GraphPad Prism 9.5.1 software.

### Evaluation of blood–brain barrier penetration of K223-pe

To assess the blood–brain barrier permeability of K223-pe, C57BL/6 mice were administered a single intraperitoneal injection of FITC-labeled K223-pe (5 mg/kg). Brains were collected 4 h after injection, and brain tissues were processed for fluorescence imaging to evaluate the distribution of K223-pe.

### Pan-Kla-based PTM enrichment

Primary hippocampal neurons were exposed to either normal glucose (5.5 mmol/L D-glucose) or high glucose (25 mmol/L D-glucose) for 72 h. Cells were lysed and sonicated on ice using an ultrasonic processor. The resulting protein lysate was digested with trypsin. The tryptic peptides were incubated overnight at 4 °C with Pan-Kla antibody and prewashed beads to selectively enrich Kla-modified peptides. The captured peptides were purified using a self-priming desalting column. Finally, the desalted peptides were dissolved in 0.1% formic acid and prepared for subsequent analysis.

### LC-MS/MS analysis and database search

LC-MS/MS analysis was performed in collaboration with BiotechPack Scientific Co., Ltd (Beijing, China). Tryptic peptides were separated on a homemade nanocolumn (180 mm in length, 100-μm inner diameter) using the Easy-nLC 1200 system (Thermo Fisher Scientific, USA). The LC-MS/MS analysis was conducted on an Orbitrap Fusion™ Lumos™ Tribrid™ mass spectrometer (Thermo Fisher Scientific, USA). Data processing and database searches were carried out using PEAKS Studio software (version 10.6). The relative quantification of modified peptides was achieved by normalizing signal intensity values across different samples. Kla peptide ratios were further adjusted based on their corresponding protein expression levels.

### Statistical analysis

Clinical data were statistically analyzed using IBM SPSS Statistics 29 software. Normality of data distribution was assessed with the Kolmogorov–Smirnov test. Skewed variables were log-transformed prior to analysis. Continuous variables were presented as mean ± SD if normally distributed, or median (interquartile range) if skewed. Pearson’s correlation was used to examine associations between plasma LRPPRC K224la and continuous covariates.

To assess the association between baseline plasma LRPPRC K224la levels and the risk of MCI, Cox proportional hazards regression models were employed. Hazard ratios (HRs) and 95% confidence intervals (CIs) were calculated, with the lowest quartile of LRPPRC K224la serving as the reference. Two models were constructed: Model 1 (unadjusted) and Model 2, adjusted for a comprehensive set of potential confounders including age, gender, BMI, cigarette smoking, habitual alcohol consumption, leisure-time physical activity, education level, annual income, diabetes therapy, statin use, NSAID use, duration of diabetes, diabetic nephropathy, cardiovascular disease, SBP, TG, HDL-C, and HbA1c. The Kaplan–Meier method and log-rank test were used to visualize and compare the incidence of MCI across LRPPRC K224la quartiles.

Restricted cubic splines were employed to explore the potential non-linear relationship between plasma LRPPRC K224la and MCI risk. Knots were placed at the 10th, 50th, and 90th percentiles of LRPPRC K224la levels, with the median as the reference.

For experiments in mice and cell culture, data are expressed as mean ± standard error of the mean (SEM) and were analyzed using Prism version 9.0 (GraphPad). The Kolmogorov–Smirnov test was used to assess the normality of data distribution. For normally distributed data, differences between two groups were evaluated with a two-tailed Student’s *t*-test, while non-normally distributed data were compared using the Mann–Whitney *U*-test. For comparisons involving multiple groups with a single variable, one-way ANOVA followed by Tukey’s post hoc test was applied. When multiple variables were involved, a two-way ANOVA with Tukey’s post hoc test was used. For non-normally distributed data, the Kruskal–Wallis test followed by Dunn’s post hoc test was conducted. Statistical details, including the test used, sample size (*n*), and *p* values, are provided in the figure legends. Statistical significance was indicated by asterisks (*P < 0.05, **P < 0.01, ***P < 0.001). Mice were randomly assigned to experimental groups, and investigators were blinded to group assignments during surgeries and outcome assessments. Exclusion criteria were based on animal health considerations, though no animals were excluded. Sample sizes were determined based on previous experience with diabetic mice in the lab. For behavioral tests, sample sizes were further estimated using G*Power (v3.1.9.4) based on previous studies (Choi et al, 2025; Choi et al, 2023; Monteiro et al, 2016) with partial η² = 0.15, power = 0.8, and α = 0.05.

## Supplementary information

Appendix

Peer Review File

Source data Fig. 1

Source data Fig. 2

Source data Fig. 3

Source data Fig. 4

Source data Fig. 5

Source data Fig. 6

Source data Fig. 7

## Data Availability

This study includes no data deposited in external repositories. No unique datasets or codes were generated, and all other data can be made available from the authors on reasonable request. The source data of this paper are collected in the following database record: biostudies:S-SCDT-10_1038-S44321-026-00422-8.
