# Supplementary material for: Hyperglycemia impairs cognitive function by inducing mitochondrial damage through lactylation of LRPPRC at K223
Source: EMBO Mol Med. 2026 Apr 6;18(6):2038–61. doi: 10.1038/s44321-026-00422-8 (PMC13269535; doi:10.1038/s44321-026-00422-8)
Supplement: Supplementary file 1 — Appendix [file 44321_2026_422_MOESM1_ESM.pdf]

# Table of contents

Appendix Figure S1. .......... 2

Appendix Figure S2. .......... 3

Appendix Figure S3. .......... 5

Appendix Figure S4. .......... 8

Appendix Figure S5. .......... 10

Appendix Figure S6. .......... 12

Appendix Figure S7. .......... 15

Appendix Figure S8. .......... 16

Appendix Figure S9 .......... 18

Appendix Figure S10 .......... 19

Appendix Figure S11. .......... 21

Appendix Figure S12. .......... 23

Appendix Table S1. .......... 24

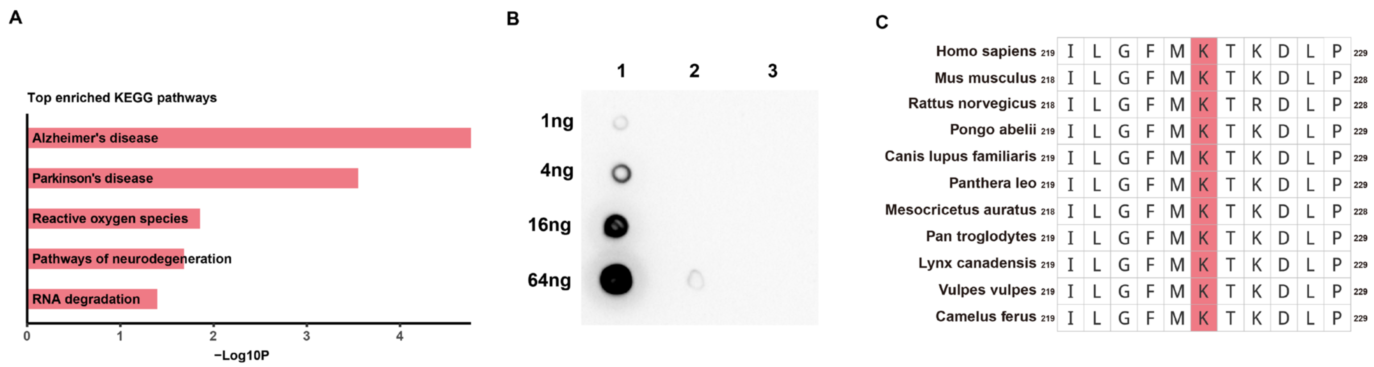


**Appendix Figure S1. The mitochondrial protein LRPPRC K223 is lactylated under high glucose conditions, related to Figure 1.**

**(A):** KEGG pathways enriched in differentially lactylated mitochondrial proteins.

**(B):** Verification of the specificity of the LRPPRC K223la antibody by dot blots experiment. 1: modified peptide 1 (ILGFMK(lac)TKDLP); 2: modified peptide 2 (ILGFMKTK(lac)DLP); and 3: non-modified peptide (ILGFMKTKDLP).

**(C):** Cross-species sequence alignment of LRPPRC.

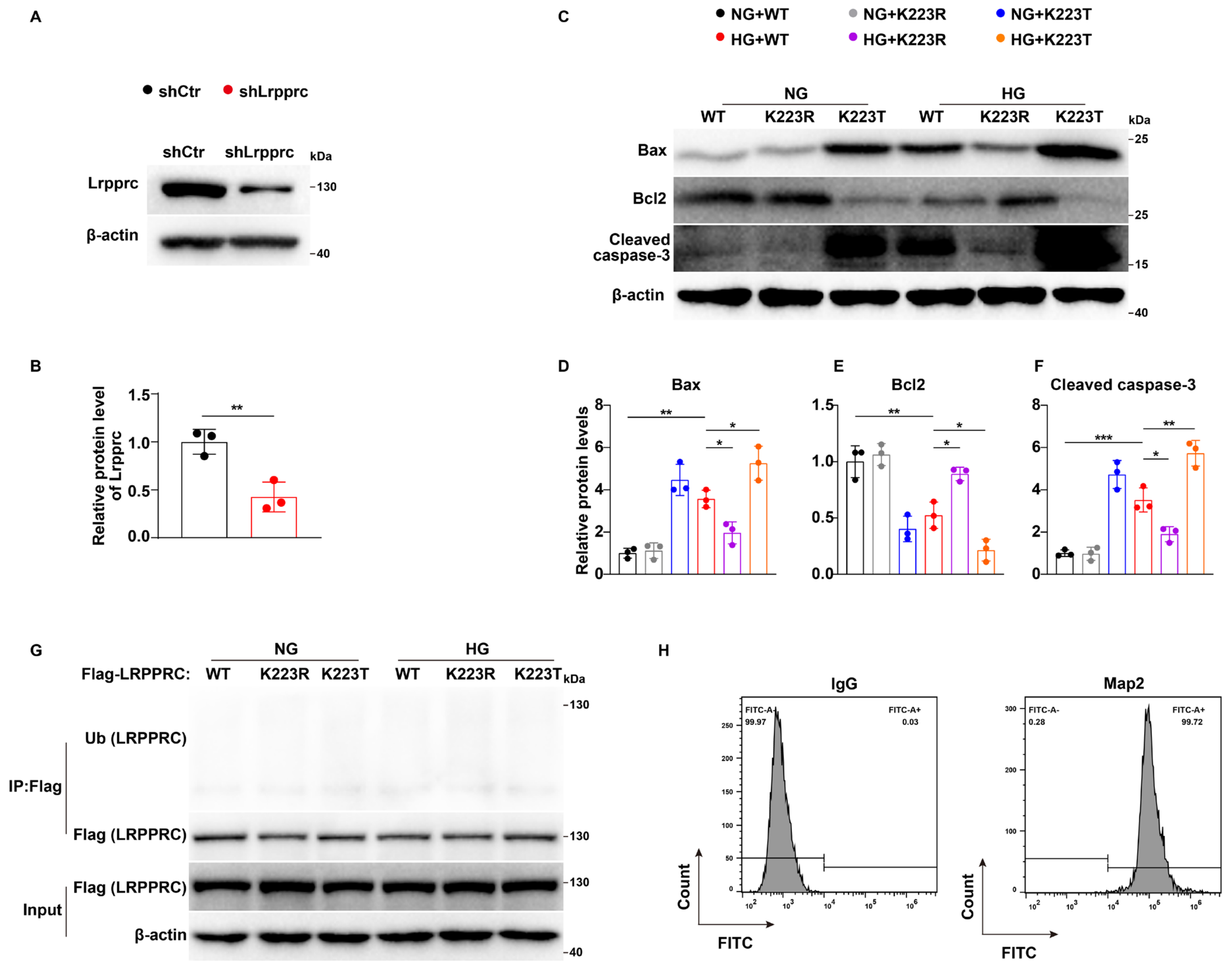


**Appendix Figure S2. LRPPRC K223 lactylation results in hippocampal neuronal apoptosis and cognitive impairment, related to Figure 2.**

**(A-B):** Immunoblots (A) and quantification analysis (B) of protein level of LRPPRC in primary hippocampal neurons transduced with shCtr or shLRPPRC (n = 3 biological replicates).

**(C-F):** Immunoblots (C) and quantification (D-F) analysis of Bax, Bcl-2 and Cleaved-caspase3 expression levels in the primary hippocampal neurons with endogenous LRPPRC stably silenced and expressing either wild-type (WT) LRPPRC, K223R LRPPRC or K223T LRPPRC, treated with normal glucose (NG, 5.5 mmol/L D-glucose) or high glucose (HG, 25 mmol/L D-glucose) (n = 3 biological replicates).

**(G):** Primary hippocampal neurons, which were stably silenced endogenous LRPPRC and expressed Flag-tagged wild-type (WT) LRPPRC, K223R LRPPRC or K223T LRPPRC, were treated with normal glucose (NG, 5.5 mmol/L D-glucose) or high glucose (HG, 25 mmol/L D-glucose). Cell lysates were

immunoprecipitated with anti-Flag antibodies and western blotted with the indicated antibodies (n = 3 biological replicates).

**(H):** Purity of isolated hippocampal CA1 neurons by flow cytometry.

Data are means ± SEM. *$p<0.05$, **$P<0.01$, ***$p< 0.001$. Two-tailed Student's unpaired t test analysis (B), Two-way ANOVA followed by Tukey's test (D-F).

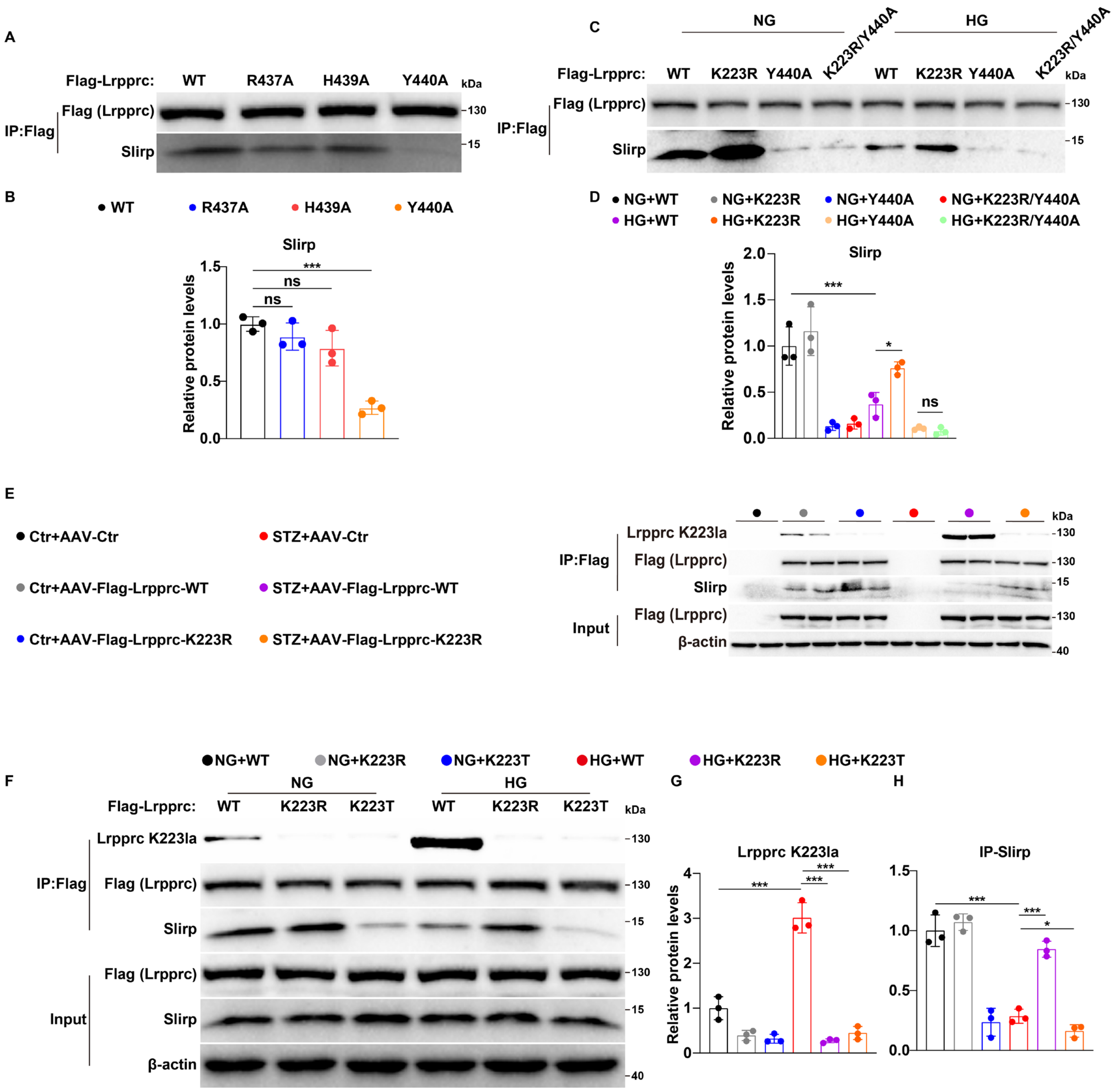


**Appendix Figure S3. LRPPRC K223 lactylation reduces its binding to SLIRP, related to Figure 3.**

**(A-B):** Primary hippocampal neurons were transfected with the indicated plasmids, Slirp were analyzed by immunoprecipitation with anti-Flag antibodies and western blotted with the indicated antibodies (A). Quantification analysis of Slirp (B) in the indicated groups (n = 3 biological replicates).

**(C-D):** Primary hippocampal neurons transfected with the indicated plasmids were treated with normal glucose (NG, 5.5 mmol/L D-glucose) or high glucose (HG, 25 mmol/L D-glucose). Cell lysates were

immunoprecipitated with anti-Flag antibodies and western blotted with the indicated antibodies (C). Quantification analysis of Slirp (D) levels in the indicated groups (n = 3 biological replicates).

**(E):** Hippocampal CA1 neurons were isolated from Ctr or STZ mice received stereotactic injections of AAV-Ctr, AAV-Flag-LRPPRC-WT or AAV-Flag-LRPPRC-K223R to specifically express either wild-type LRPPRC or the K223R mutant LRPPRC, and cell lysates were immunoprecipitated with anti-Flag antibodies and western blotted with the indicated antibodies (n = 6 mice per group).

**(F-H):** Primary hippocampal neurons, which were stably silenced endogenous LRPPRC and expressed Flag-tagged WT LRPPRC, K223R LRPPRC or K223T LRPPRC, were treated with normal glucose (NG, 5.5 mmol/L D-glucose) or high glucose (HG, 25 mmol/L D-glucose). Cell lysates were immunoprecipitated with anti-Flag antibodies and western blotted with the indicated antibodies (F). Quantification analysis of Lrpprc K223la (G) and IP-Slirp (H) levels in the indicated groups (n = 3 biological replicates).

Data are means ± SEM. *p<0.05, **P<0.01, ***p< 0.001. One-way ANOVA followed by Tukey's test (B). Two-way ANOVA followed by Tukey's test (D and G-H).

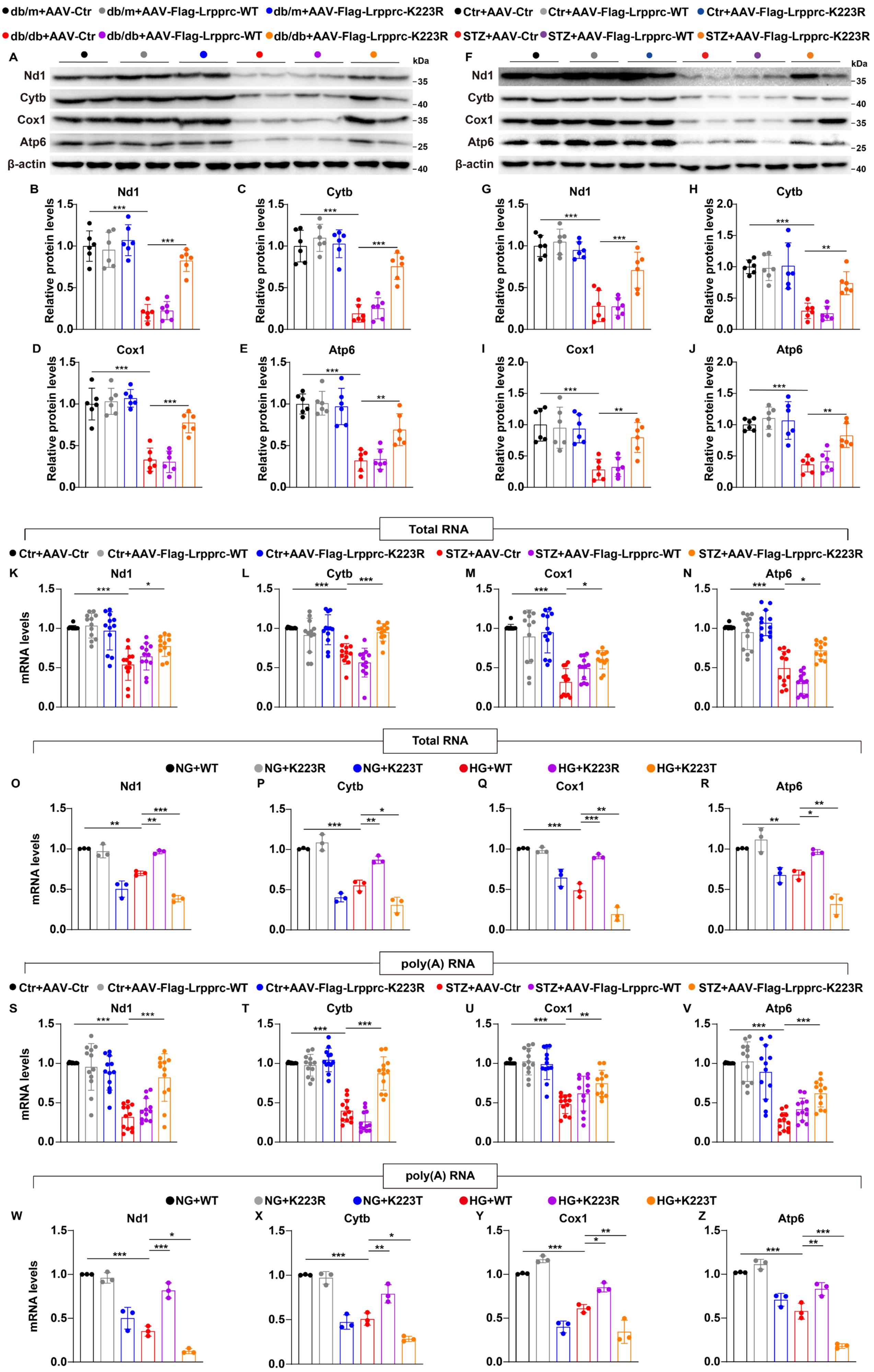
db/m+AAV-Ctr
db/m+AAV-Flag-Lrpprc-WT
db/m+AAV-Flag-Lrpprc-K223R
db/db+AAV-Ctr
db/db+AAV-Flag-Lrpprc-WT
db/db+AAV-Flag-Lrpprc-K223R
Ctr+AAV-Ctr
Ctr+AAV-Flag-Lrpprc-WT
Ctr+AAV-Flag-Lrpprc-K223R
STZ+AAV-Ctr
STZ+AAV-Flag-Lrpprc-WT
STZ+AAV-Flag-Lrpprc-K223R
A
F
kDa
Nd1
Cytb
Cox1
Atp6
β-actin
35
40
35
25
40
B
C
D
E
G
H
I
J
Relative protein levels
Total RNA
K
L
M
N
mRNA levels
NG+WT
NG+K223R
NG+K223T
HG+WT
HG+K223R
HG+K223T
O
P
Q
R
poly(A) RNA
S
T
U
V
W
X
Y
Z

**Appendix Figure S4. LRPPRC K223 lactylation reduces its binding to SLIRP, and subsequently weakens mitochondrial mRNA stability, related to Figure 3.**

**(A-E):** Immunoblots (A) and quantification analysis of Nd1 (B), Cytb (C), Cox1 (D), and Atp6 (E) expression levels in the hippocampal CA1 neurons of db/m, db/db mice received stereotactic injections of AAV-Ctr, AAV-Flag-LRPPRC-WT or AAV-Flag-LRPPRC-K223R to specifically express either wild-type LRPPRC or the K223R mutant LRPPRC (n = 6 mice per group).

**(F-J):** Immunoblots (F) and quantification analysis of Nd1 (G), Cytb (H), Cox1 (I), and Atp6 (J) expression levels in the hippocampal CA1 neurons of Ctr, STZ mice received stereotactic injections of AAV-Ctr, AAV-Flag-LRPPRC-WT or AAV-Flag-LRPPRC-K223R to specifically express either wild-type LRPPRC or the K223R mutant LRPPRC (n = 6 mice per group).

**(K-N):** Total RNA was isolated from the hippocampal CA1 neurons of Ctr or STZ mice received stereotactic injections of AAV-Ctr, AAV-Flag-LRPPRC-WT or AAV-Flag-LRPPRC-K223R to specifically express either wild-type LRPPRC or the K223R mutant LRPPRC. The levels of each mtRNA species were determined by PCR (n = 12 mice per group).

**(O-R):** Primary hippocampal neurons, which were stably silenced endogenous LRPPRC and expressed WT LRPPRC, K223R LRPPRC or K223T LRPPRC, were treated with normal glucose (NG, 5.5 mmol/L D-glucose) or high glucose (HG, 25 mmol/L D-glucose). Total RNA was isolated, and the levels of each mtRNA species were determined by PCR (n = 3 biological replicates).

**(S-V)** Poly(A)-tailed RNA was isolated from the hippocampal CA1 neurons of Ctr or STZ mice received stereotactic injections of AAV-Ctr, AAV-Flag-LRPPRC-WT or AAV-Flag-LRPPRC-K223R to specifically express either wild-type LRPPRC or the K223R mutant LRPPRC. The levels of each mtRNA species were determined by PCR (n = 12 mice per group).

**(W-Z):** Primary hippocampal neurons, which were stably silenced endogenous LRPPRC and expressed WT LRPPRC, K223R LRPPRC or K223T LRPPRC, were treated with normal glucose (NG, 5.5 mmol/L D-glucose) or high glucose (HG, 25 mmol/L D-glucose). Poly(A)-tailed RNA was isolated, and the levels of each mtRNA species were determined by PCR (n = 3 biological replicates).

Data are means ± SEM. *$p<0.05$, **$P<0.01$, ***$p< 0.001$. Two-way ANOVA followed by Tukey's test (B-E, G-J, and K-Z).

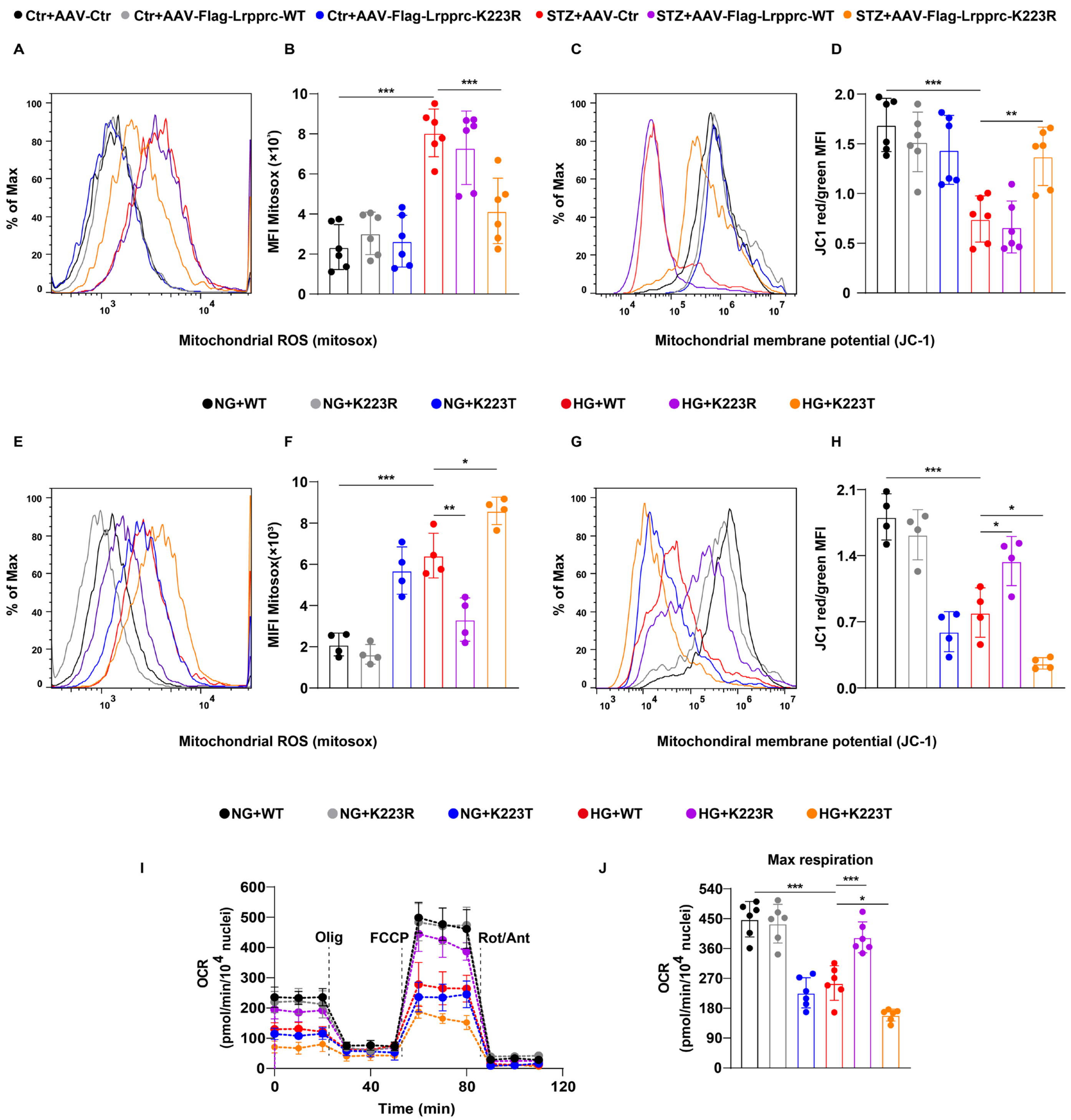


**Appendix Figure S5. LRPPRC K223 lactylation reduces its binding to SLIRP, weakens mitochondrial mRNA stability, and triggers subsequent mitochondrial dysfunction, related to Figure 3.**

**(A-B):** Flow cytometry (A) and quantification analysis (B) of mtROS levels in the hippocampal CA1 neurons of Ctr or STZ mice received stereotactic injections of AAV-Ctr, AAV-Flag-LRPPRC-WT or AAV-Flag-

LRPPRC-K223R to specifically express either wild-type LRPPRC or the K223R mutant LRPPRC (n = 6 mice per group).

**(C-D):** Flow cytometry (C) and quantification analysis (D) of MMP levels in the hippocampal CA1 neurons of Ctr or STZ mice received stereotactic injections of AAV-Ctr, AAV-Flag-LRPPRC-WT or AAV-Flag-LRPPRC-K223R to specifically express either wild-type LRPPRC or the K223R mutant LRPPRC (n = 6 mice per group).

**(E-F):** Flow cytometry (E) and quantification analysis (F) of mtROS levels in primary hippocampal neurons with endogenous LRPPRC stably silenced and expressing either wild-type (WT) LRPPRC, K223R LRPPRC or K223T LRPPRC, treated with normal glucose (NG, 5.5 mmol/L D-glucose) or high glucose (HG, 25 mmol/L D-glucose) (n = 4 biological replicates).

**(G-H):** Flow cytometry (G) and quantification analysis (H) of MMP levels in primary hippocampal neurons with endogenous LRPPRC stably silenced and expressing either wild-type (WT) LRPPRC, K223R LRPPRC or K223T LRPPRC, treated with normal glucose (NG, 5.5 mmol/L D-glucose) or high glucose (HG, 25 mmol/L D-glucose) (n = 4 biological replicates).

**(I-J):** OCR measurements of primary hippocampal neurons with endogenous LRPPRC stably silenced and expressing either wild-type (WT) LRPPRC, K223R LRPPRC or K223T LRPPRC, treated with normal glucose (NG, 5.5 mmol/L D-glucose) or high glucose (HG, 25 mmol/L D-glucose) under basal conditions and in response to oligomycin (Olig), *p*-trifluoromethoxy carbonyl cyanide phenylhydrazone (FCCP), antimycin A/rotenone (Rot/Ant) (I); the quantification of the maximal OCR (J) (n= 6 biological replicates).

Data are means ± SEM. *p<0.05, **P<0.01, ***p< 0.001. Two-way ANOVA followed by Tukey's test (B, D, F, H, and J).

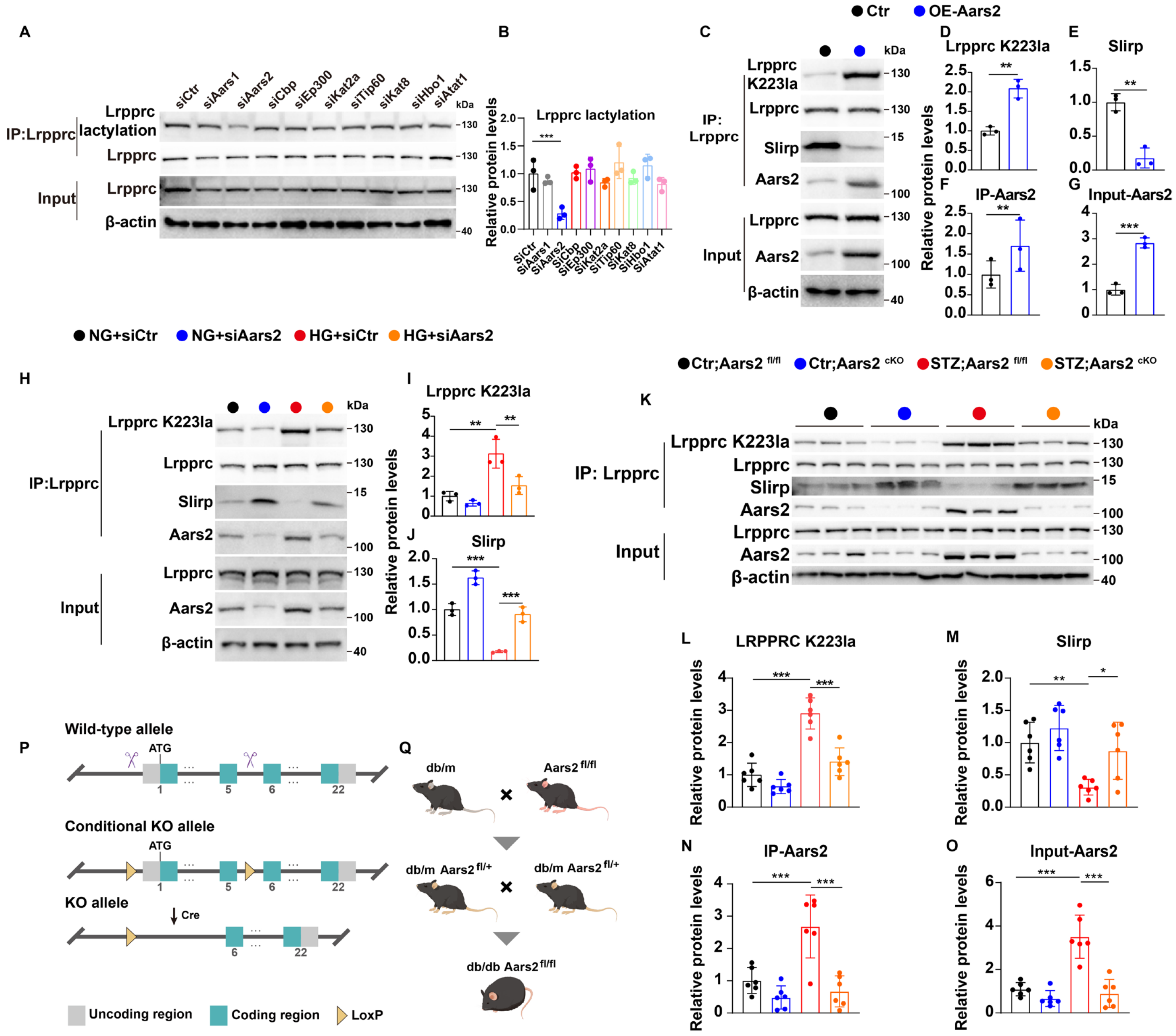

**Appendix Figure S6. LRPPRC K223 is lactylated by Aars2 under high-glucose conditions, related to Figure 4.**

**(A-B):** Screening of LRPPRC lactylation writer. Lactyltransferases (Aars1, Aars2, Cbp, Ep300, Kat2a, Tip60, Kat8, Hbo1 and Atat1) were knocked down in primary hippocampal neurons, and the lactylation level of LRPPRC was detected by immunoprecipitation and Western blot (A). Quantification analysis of LRPPRC lactylation (B) levels in the indicated groups (n = 3 biological replicates).

**(C-G):** Primary hippocampal neurons were transfected with Aars2 overexpression plasmid or control plasmid, and LRPPRC K223 lactylation was determined by immunoprecipitation and western blotted using the

indicated antibodies (C). Quantification analysis of LRPPRC K223la (D), Slirp (E), IP-Aars2 (F) and Input-Aars2 (G) levels in the indicated groups (n = 3 biological replicates).

**(H-J):** Immunoblots (H) and quantification analysis of protein levels of LRPPRC K223la (I) and SLIRP (J) in primary hippocampal neurons treated with Aars2 siRNA or control siRNA in the presence of normal glucose (NG, 5.5 mmol/L D-glucose) or high glucose (HG, 25 mmol/L D-glucose) (n = 3 biological replicates).

**(K-O):** Hippocampal CA1 neurons were isolated from Ctr Aars2 $^{fl/fl}$ or STZ Aars2 $^{fl/fl}$ mice injected with AAV-CAMKII (as a control vector) or AAV-CAMKII-Cre, and cell lysates were immunoprecipitated with LRPPRC antibodies and western blotted with the indicated antibodies (K). Quantification analysis of LRPPRC K223la (L), Slirp (M), IP-Aars2 (N) and Input-Aars2 (O) levels in the indicated groups (n = 6 mice per group).

**(P):** Strategy for production of Aars2 $^{fl/fl}$ mice.

**(Q):** Experimental scheme for generating the db/db Aars2 $^{fl/fl}$ mice.

Data are means ± SEM. $^*p<0.05$, $^{**}P<0.01$, $^{***}p< 0.001$. Two-tailed Student's unpaired t test analysis (D-G), One-way ANOVA followed by Tukey's test (B), Two-way ANOVA followed by Tukey's test (I-J, and L-O).

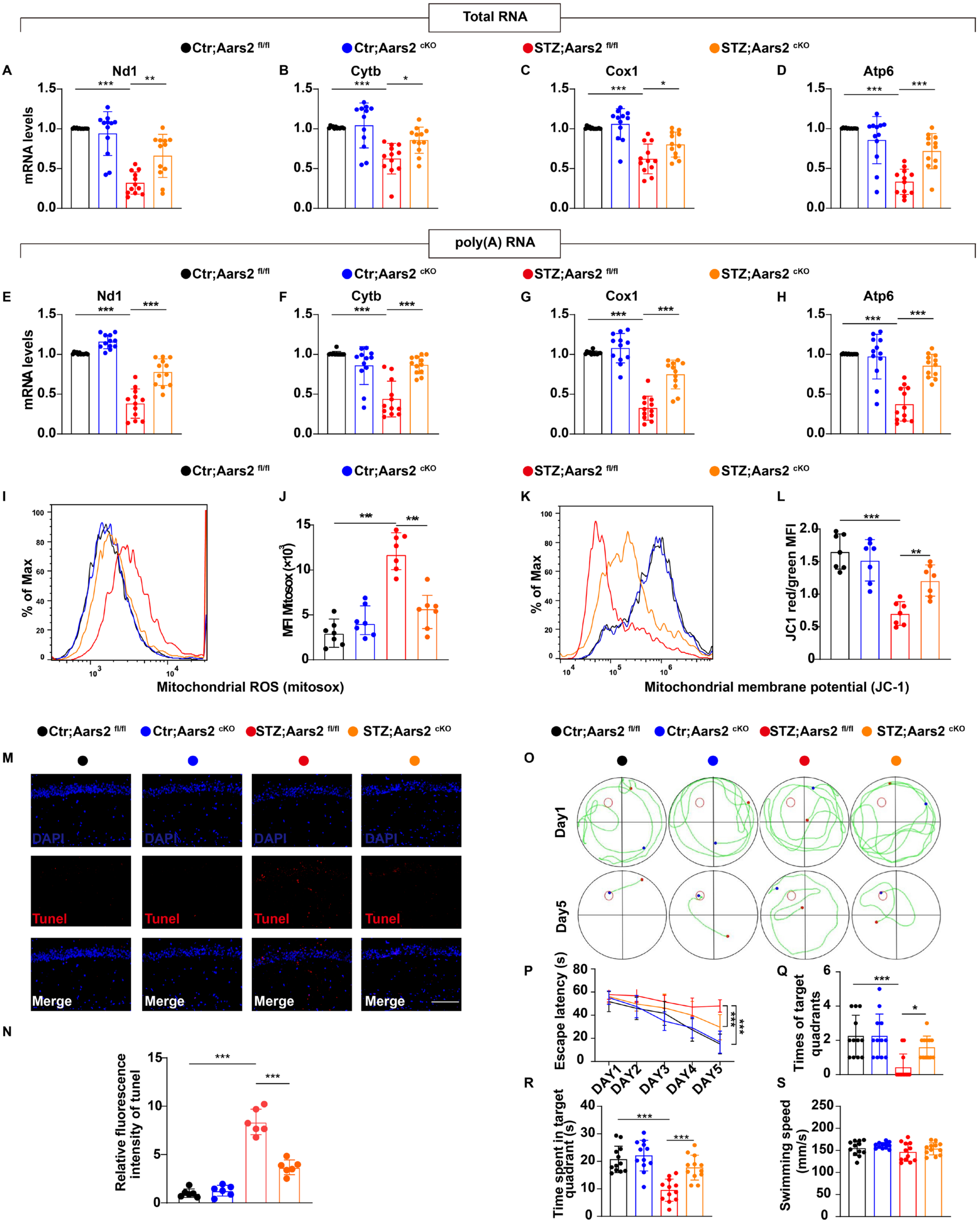

Total RNA
Ctr;Aars2 fl/fl
Ctr;Aars2 cKO
STZ;Aars2 fl/fl
STZ;Aars2 cKO
A
Nd1
B
Cytb
C
Cox1
D
Atp6
mRNA levels
poly(A) RNA
E
F
G
H
I
J
K
L
% of Max
Mitochondrial ROS (mitosox)
MFI Mitosox (×10³)
Mitochondrial membrane potential (JC-1)
JC1 red/green MFI
M
DAPI
Tunel
Merge
N
Relative fluorescence intensity of tunel
O
Day1
Day5
P
Escape latency (s)
DAY1
DAY2
DAY3
DAY4
DAY5
Q
Times of target quadrants
R
Time spent in target quadrant (s)
S
Swimming speed (mm/s)

**Appendix Figure S7. High glucose induces Aars2-mediated lactylation of LRPPRC at K223, thereby disrupting LRPPRC-SLIRP interaction, destabilizes mitochondrial mRNA and triggers mitochondrial dysfunction, ultimately leading to hippocampal neuronal apoptosis and cognitive impairment, related to Figure 4.**

**(A-H):** Total RNA (A-D) or poly(A)-tailed RNA (E-H) was isolated from the hippocampal CA1 neurons of Ctr Aars2 $^{fl/fl}$ or STZ Aars2 $^{fl/fl}$ mice injected with AAV-CAMKII (as a control vector) or AAV-CAMKII-Cre (n = 12 mice per group). The levels of each mtRNA species were determined by PCR.

**(I-J):** Flow cytometry (I) and quantification analysis (J) of mtROS levels in the hippocampal CA1 neurons of Ctr Aars2 $^{fl/fl}$ or STZ Aars2 $^{fl/fl}$ mice injected with AAV-CAMKII (as a control vector) or AAV-CAMKII-Cre (n = 7 mice per group).

**(K-L):** Flow cytometry (K) and quantification analysis (L) of MMP levels in the hippocampal CA1 neurons of Ctr Aars2 $^{fl/fl}$ or STZ Aars2 $^{fl/fl}$ mice injected with AAV-CAMKII (as a control vector) or AAV-CAMKII-Cre (n = 7 mice per group).

**(M-N):** Representative images (M) and quantifications (N) showing IF staining of Tunel (red) in the hippocampal CA1 neurons of Ctr Aars2 $^{fl/fl}$, STZ Aars2 $^{fl/fl}$ mice injected with AAV-CAMKII or AAV-CAMKII-Cre (n = 6 mice per group). Scale bar: 100 μm.

**(O-S):** Representative track images (O), escape latency to the platform (P) and swimming speed (S) during the training trials, target entries (Q) and time spent in target quadrant (R) in the probe trial of Morris water maze of Ctr Aars2 $^{fl/fl}$, STZ Aars2 $^{fl/fl}$ mice injected with AAV-CAMKII or AAV-CAMKII-Cre (n = 12 mice per group).

Data are means ± SEM. *$p<0.05$, **$P<0.01$, ***$p< 0.001$. Two-way ANOVA followed by Tukey's test (A-H, J, L, N and P-S).

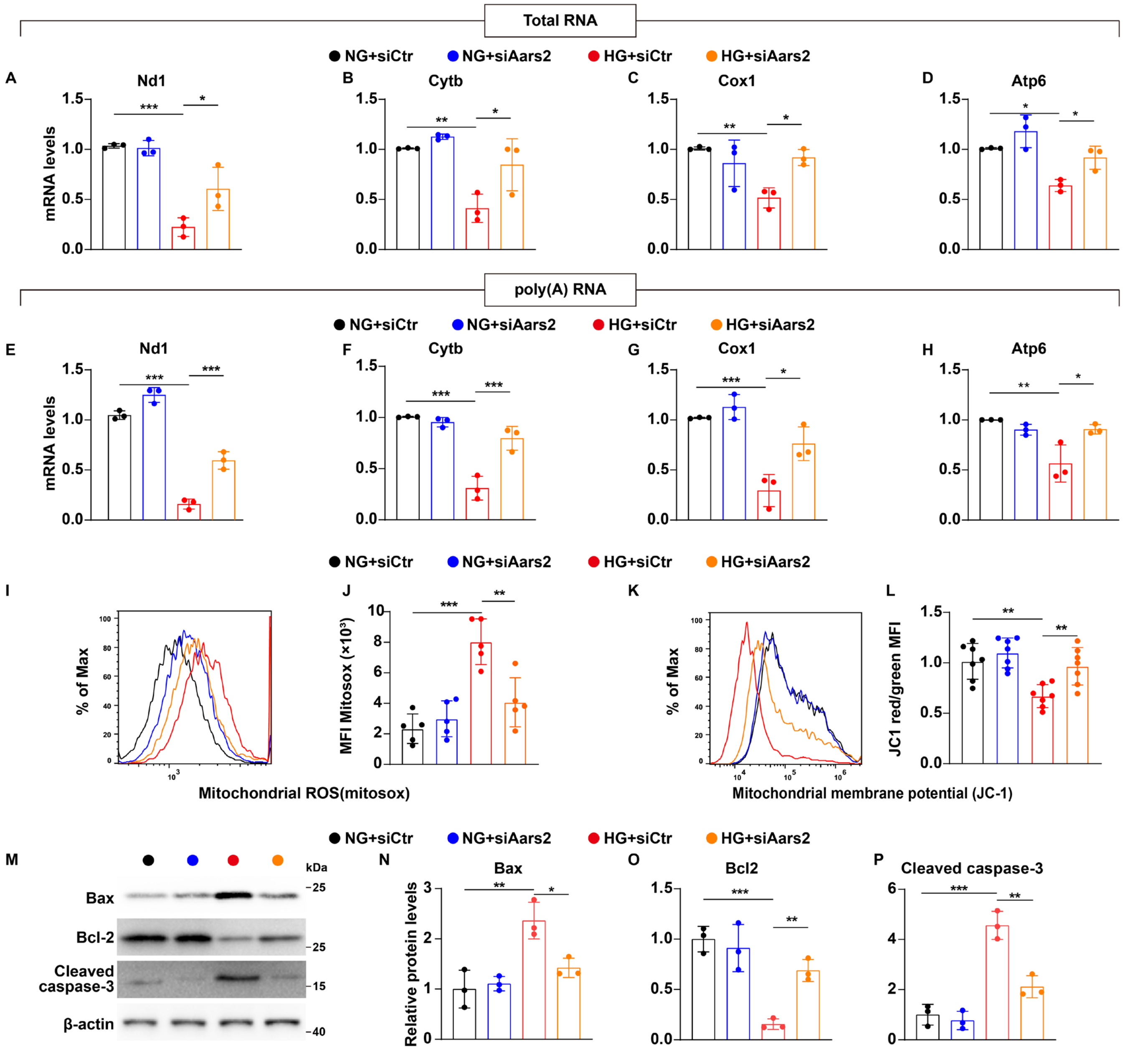


**Appendix Figure S8. High glucose induces Aars2-mediated lactylation of LRPPRC at K223, thereby disrupting LRPPRC-SLIRP interaction, destabilizes mitochondrial mRNA and triggers mitochondrial dysfunction, ultimately resulting in neuronal apoptosis, related to Figure 4.**

**(A-H):** Primary hippocampal neurons, which were treated with Aars2 siRNA or control siRNA in the presence of normal glucose (NG, 5.5 mmol/L D-glucose) or high glucose (HG, 25 mmol/L D-glucose). Total RNA (A-D) or poly(A)-tailed RNA (E-H) was isolated, and the levels of each mtRNA species were determined by PCR (n = 3 biological replicates).

**(I-J):** Flow cytometry (I) and quantification analysis (J) of mtROS levels in primary hippocampal neurons treated with Aars2 siRNA or control siRNA in the presence of normal glucose (NG, 5.5 mmol/L D-glucose) or high glucose (HG, 25 mmol/L D-glucose) (n = 5 biological replicates).

**(K-L):** Flow cytometry (K) and quantification analysis (L) of MMP levels in primary hippocampal neurons treated with Aars2 siRNA or control siRNA in the presence of normal glucose (NG, 5.5 mmol/L D-glucose) or high glucose (HG, 25 mmol/L D-glucose) (n = 7 biological replicates).

**(M-P):** Immunoblots (M) and quantification analysis of Bax (N), Bcl-2 (O) and Cleaved-caspase3 (P) expression levels in the primary hippocampal neurons treated with Aars2 siRNA or control siRNA in the presence of normal glucose (NG, 5.5 mmol/L D-glucose) or high glucose (HG, 25 mmol/L D-glucose). (n = 3 biological replicates).

Data are means ± SEM. *p<0.05, **P<0.01, ***p< 0.001. Two-way ANOVA followed by Tukey's test (A-H, J, L and N-P).

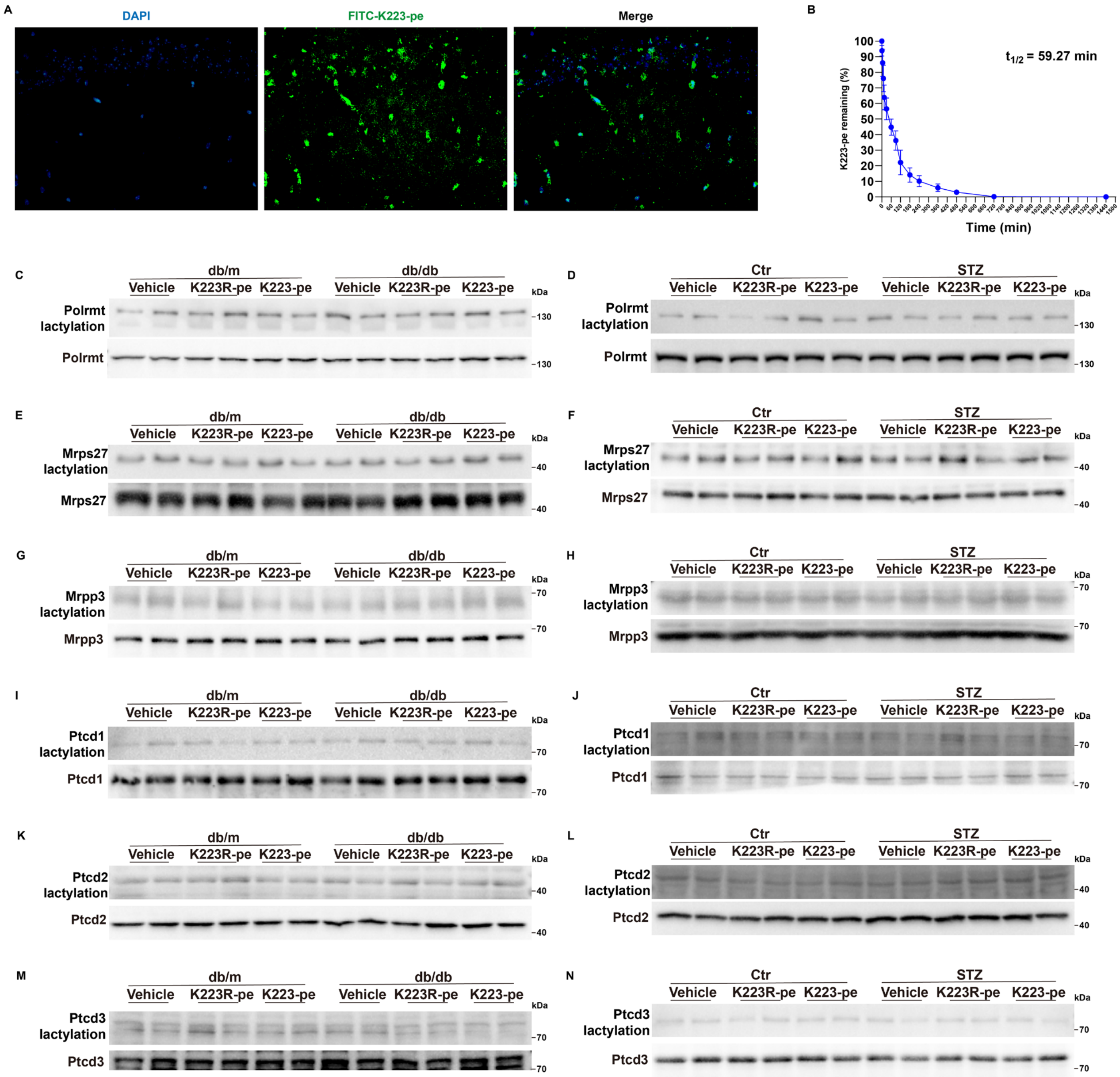


**Appendix Figure S9. K223-pe affected the lactylation level of only LRPPRC, related to Figure 5**

**(A):** DAPI staining, FITC fluorescence signal, and merged images of hippocampal CA1 tissue sections at 4 h after FITC-K223-pe injection.

**(B):** Half-life ($t_{1/2}$) of K223-pe (5mg/kg) (n = 6 mice per group).

**(C-L):** Hippocampal CA1 neurons were isolated from db/m, db/db (C, E, G ,I ,K and M), Ctr or STZ mice (D, F, H, J, L and N) treated with vehicle, K223R-pe (5 mg/kg) or K223-pe (5 mg/kg), and cell lysates were immunoprecipitated with Polrmt (C-D), Mrps27 (E-F), Mrpp3 (G-H), Ptcd1 (I-J)), Ptcd2 (K-L) or Ptcd3 (M-N) antibodies and western blotted with the indicated antibodies.

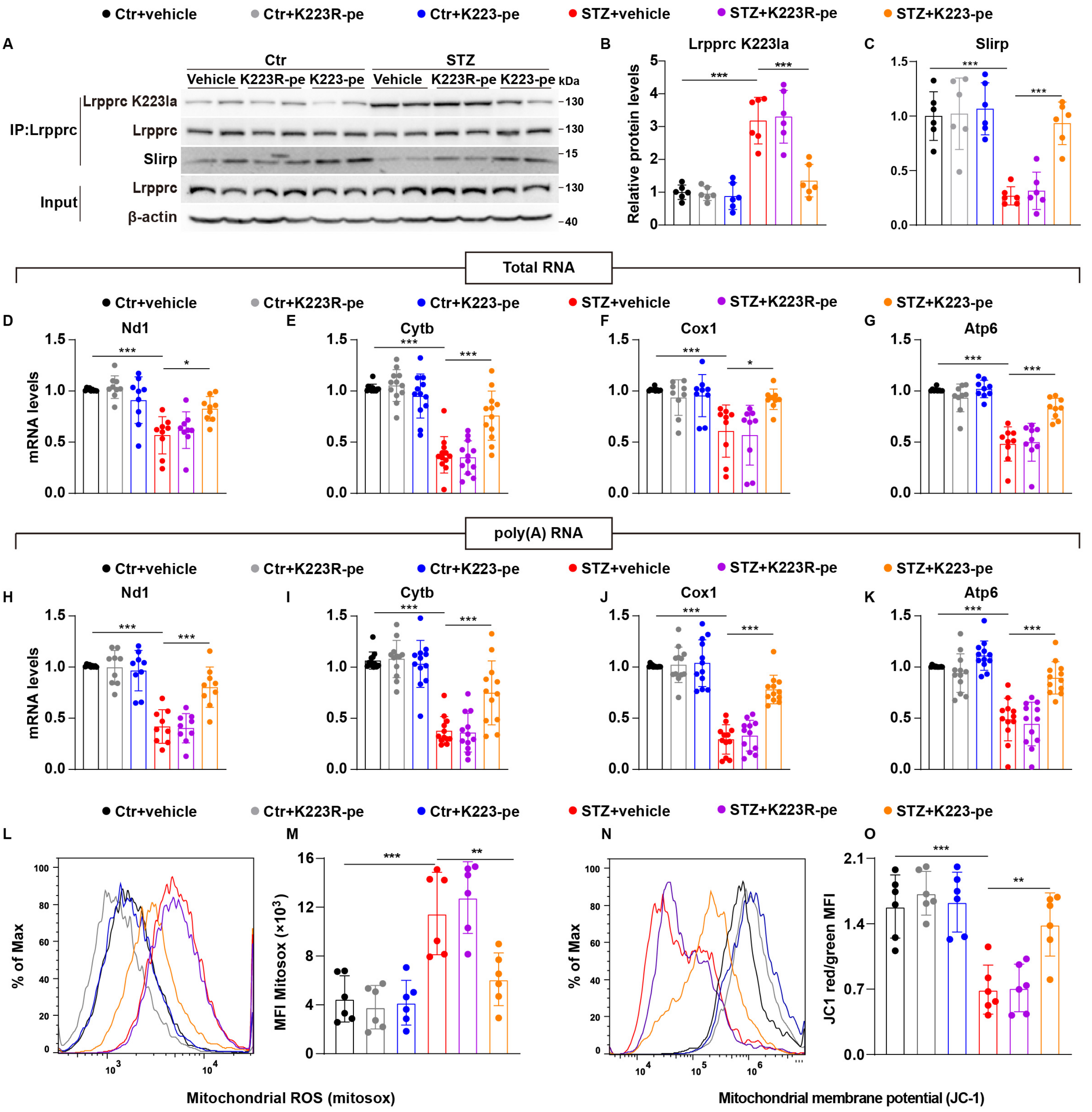


**Appendix Figure S10. K223-pe enhances mitochondrial function by regulating the LRPPRC/SLIRP/mitochondrial mRNA signaling pathway, related to Figure 5**

**(A-C):** Hippocampal CA1 neurons were isolated from Ctr or STZ mice treated with vehicle, K223R-pe (scrambled peptide, 5 mg/kg) or K223-pe (5 mg/kg), and cell lysates were immunoprecipitated with LRPPRC antibodies and western blotted with the indicated antibodies (A). Quantification analysis of LRPPRC K223la (B) and Slirp (C) levels in the indicated groups (n = 6 mice per group).

**(D-K):** Total RNA (D-G) or poly(A)-tailed RNA (H-K) was isolated from the hippocampal CA1 neurons of Ctr or STZ mice treated with vehicle, K223R-pe (scrambled peptide, 5 mg/kg) or K223-pe (5 mg/kg). The levels of each mtRNA species were determined by PCR (n = 9-12 mice per group).

**(L-M):** Flow cytometry (L) and quantification analysis (M) of mtROS levels in the hippocampal CA1 neurons of Ctr or STZ mice treated with vehicle, K223R-pe (scrambled peptide, 5 mg/kg) or K223-pe (5 mg/kg) (n = 6 mice per group).

**(N-O):** Flow cytometry (N) and quantification analysis (O) of MMP levels in the hippocampal CA1 neurons of Ctr or STZ mice treated with vehicle, K223R-pe (scrambled peptide, 5 mg/kg) or K223-pe (5 mg/kg) (n = 6 mice per group).

Data are means ± SEM. *$p<0.05$, **$P<0.01$, ***$p< 0.001$. Two-way ANOVA followed by Tukey's test (B-K, M and O).

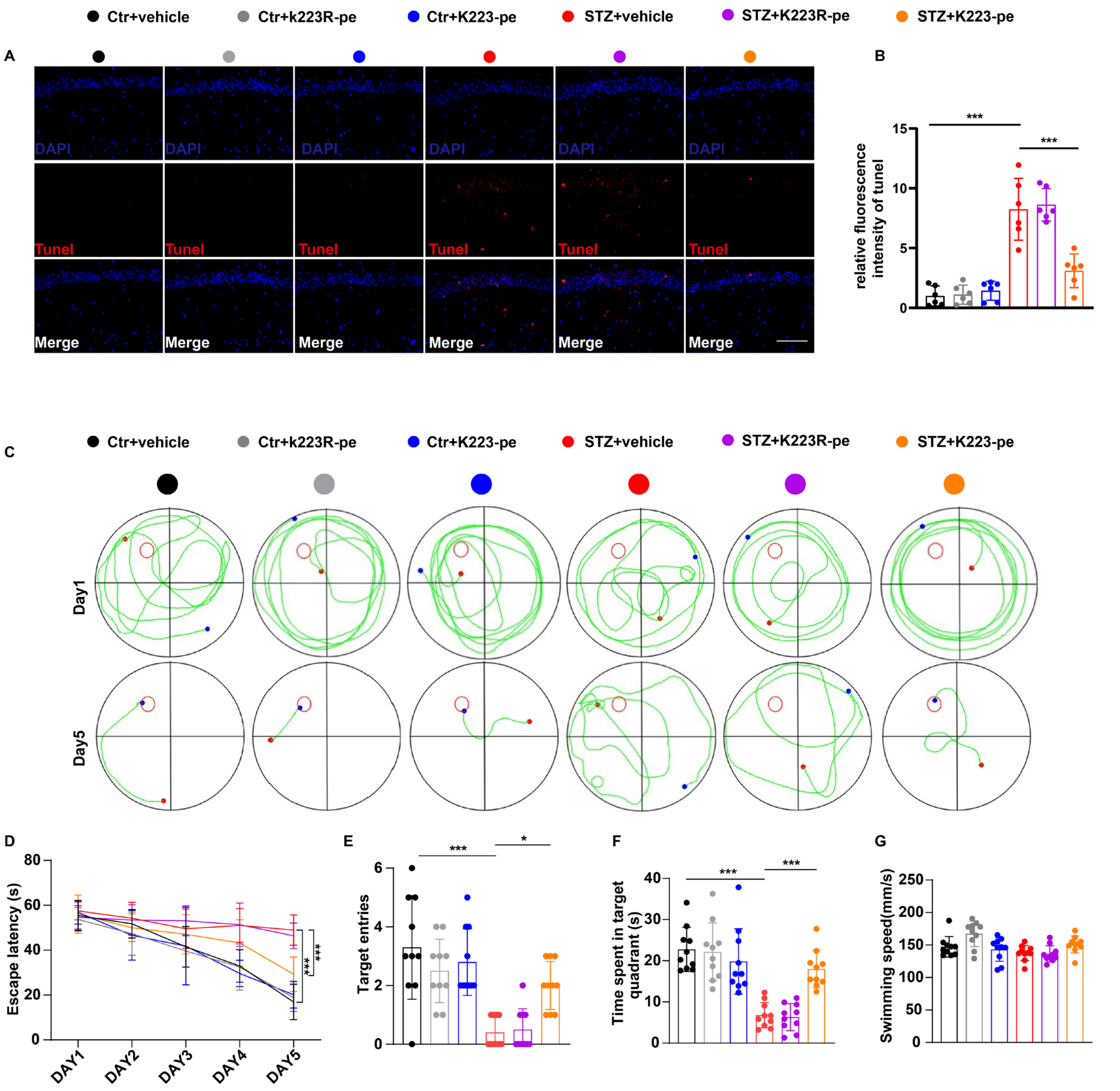


**Appendix Figure S11. K223-pe reduces neuronal apoptosis and improves cognitive function by regulating the LRPPRC/SLIRP/mitochondrial mRNA signaling pathway, related to Figure 6.**

**(A-B):** Representative images (A) and quantifications (B) showing IF staining of Tunel (red) in the hippocampal CA1 neurons of Ctr, STZ mice treated with vehicle, K223R-pe (scrambled peptide, 5 mg/kg) or K223-pe (5 mg/kg) (n = 6 mice per group). Scale bar: 100 μm.

**(C-G):** Representative track images (C), escape latency to the platform (D) and swimming speed (G) during the training trials, target entries (E) and time spent in target quadrant (F) in the probe trial of Morris water maze of Ctr, STZ mice treated with vehicle, K223R-pe (scrambled peptide, 5 mg/kg) or K223-pe (5 mg/kg)

(n =10 mice per group).

Data are means ± SEM. *p<0.05, **P<0.01, ***p< 0.001. Two-way ANOVA followed by Tukey's test (B and D-G).

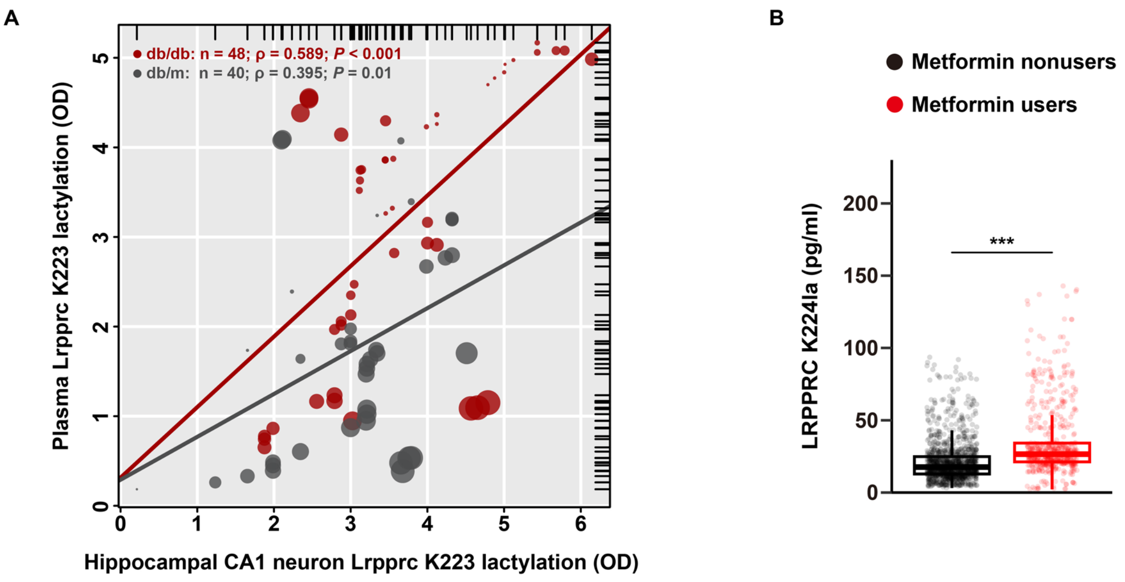


**Appendix Figure S12. Elevated plasma LRPPRC K224la levels serve as an independent risk factor for predicting mild cognitive impairment in individuals with diabetes, related to Figure 7.**

**(A):** Association between plasma LRPPRC K223 lactylation and hippocampal CA1 neuron LRPPRC K223 lactylation in db/m and db/db mice.

**(B):** LRPPRC K224la levels for metformin nonusers and users.

**Appendix Table S1. HRs for the risks of MCI according to LRPPRC K224la quartiles.**

| | Q1 | Q2 | Q3 | Q4 |
|---|---|---|---|---|
| **Without taking metformin: number of MCI (%)** | 28(8.8%) | 40(12.6%) | 48(15.1%) | 95(29.9%) |
| Model 1 | 1.0 | 1.4 (0.8, 2.2) 0.202 | 1.8 (1.1, 2.8) 0.018 | 3.7 (2.4, 5.6) <0.001 |
| Model 2 | 1.0 | 1.2 (0.8, 2.0) 0.391 | 1.5 (0.9, 2.4) 0.084 | 3.1 (2.0, 4.7) <0.001 |
| **With taking metformin: number of MCI (%)** | 19(12.8%) | 23(15.3%) | 36(24.2%) | 58(39.2%) |
| Model 1 | 1.0 | 1.3 (0.7, 2,3) 0.455 | 2.3 (1.3, 4.0) 0.004 | 3.8 (2.2, 6.3) <0.001 |
| Model 2 | 1.0 | 1.1 (0.6, 1.9) 0.891 | 1.5 (0.8, 2.7) 0.203 | 2.4 (1.4, 4.2) 0.002 |

Model1: crude model; Model2: Model1+ age + gender + BMI + cigarette smoking + habitual alcohol consumption + leisure-time physical activity + education level + annual income+ diabetes therapy + statin use + NSAID use + duration of diabetes + diabetic nephropathy + cardiovascular disease + SBP + TG + HDL-C + HbA1c; With the lowest quartile of LRPPRC K224la (Q1) as the reference category, differences between two groups were considered statistically significant at $P < 0.05$.
