# Supplementary figures and images for: Hyperglycemia impairs cognitive function by inducing mitochondrial damage through lactylation of LRPPRC at K223
Source: EMBO Mol Med. 2026 Apr 6;18(6):2038–61. doi: 10.1038/s44321-026-00422-8 (PMC13269535; doi:10.1038/s44321-026-00422-8)

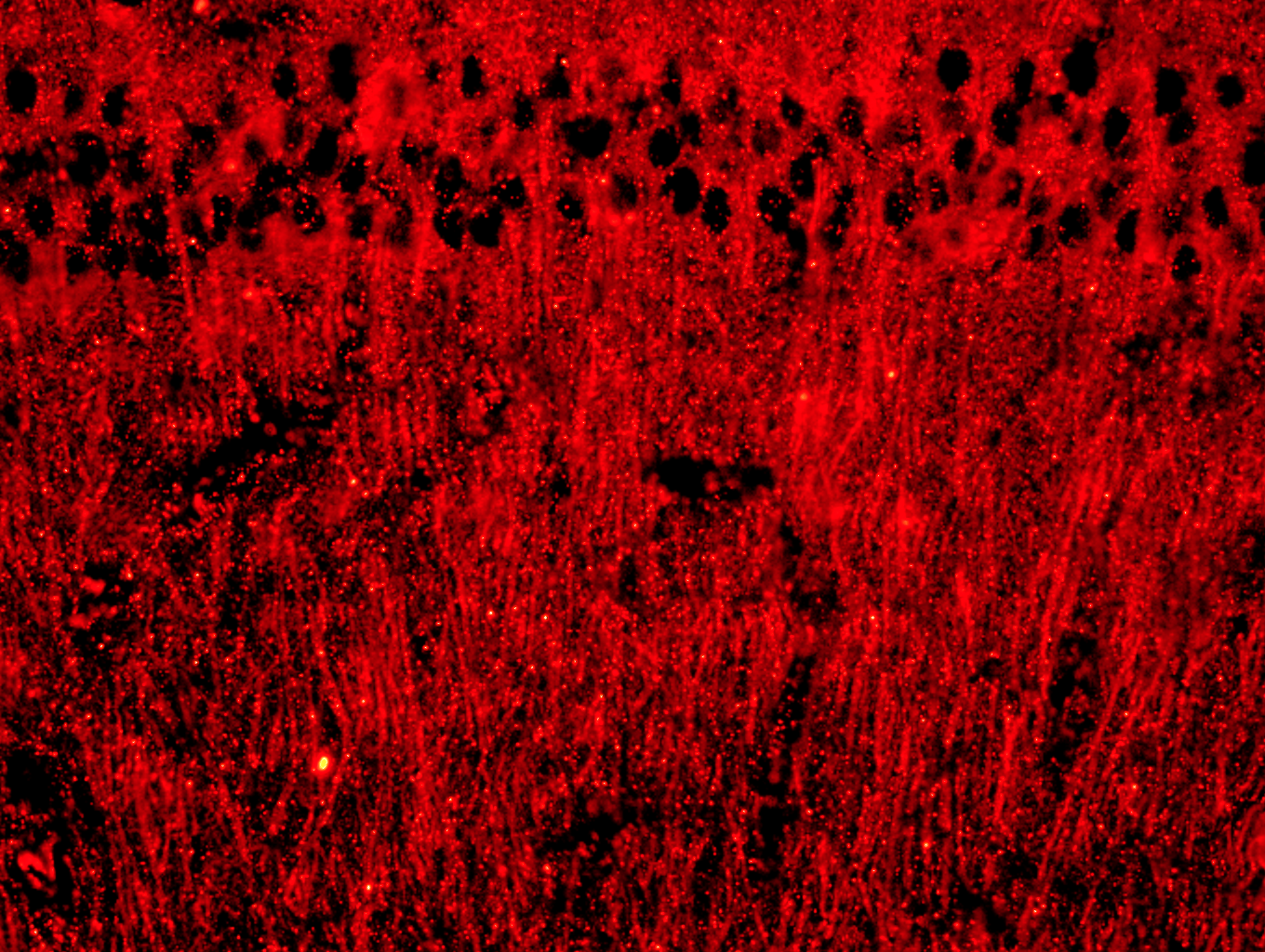

Supplement: Supplementary file 3 — Source data Fig. 1 [file 44321_2026_422_MOESM3_ESM.zip › Figure 1/1G/dbdb/Map-2.tif]

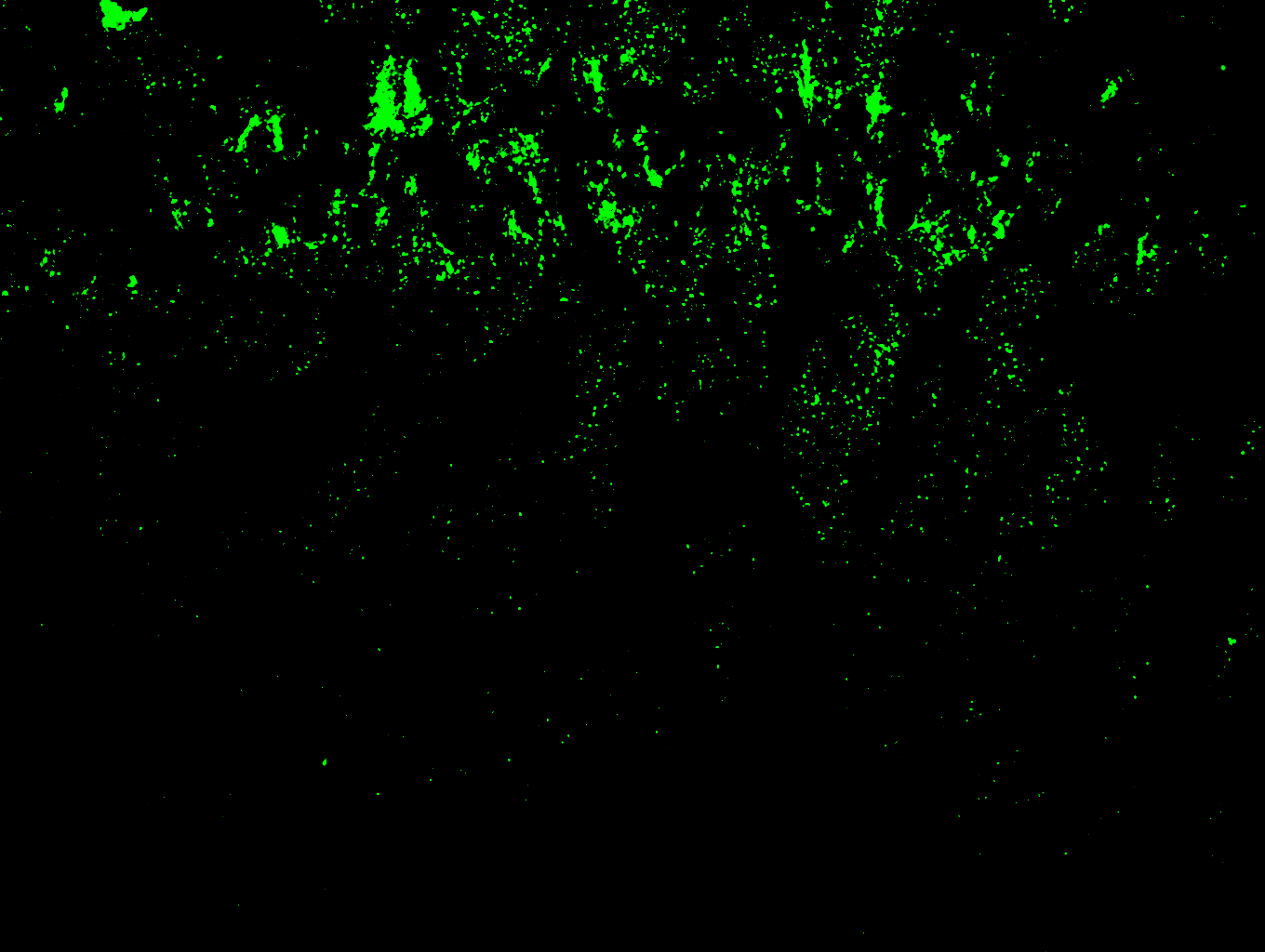

Supplement: Supplementary file 3 — Source data Fig. 1 [file 44321_2026_422_MOESM3_ESM.zip › Figure 1/1G/dbdb/Lrpprc K223la.tif]

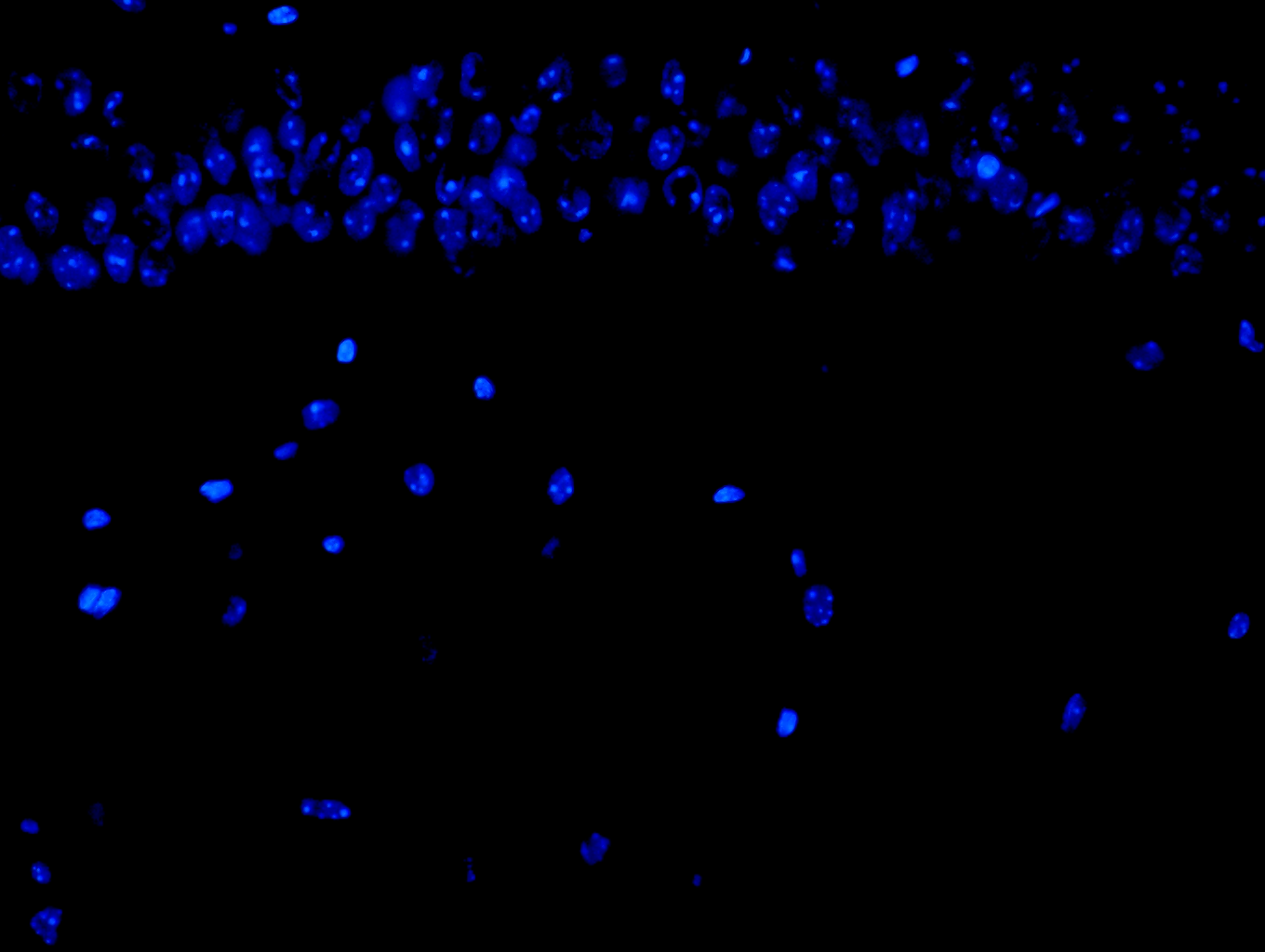

Supplement: Supplementary file 3 — Source data Fig. 1 [file 44321_2026_422_MOESM3_ESM.zip › Figure 1/1G/dbdb/DAPI.tif]

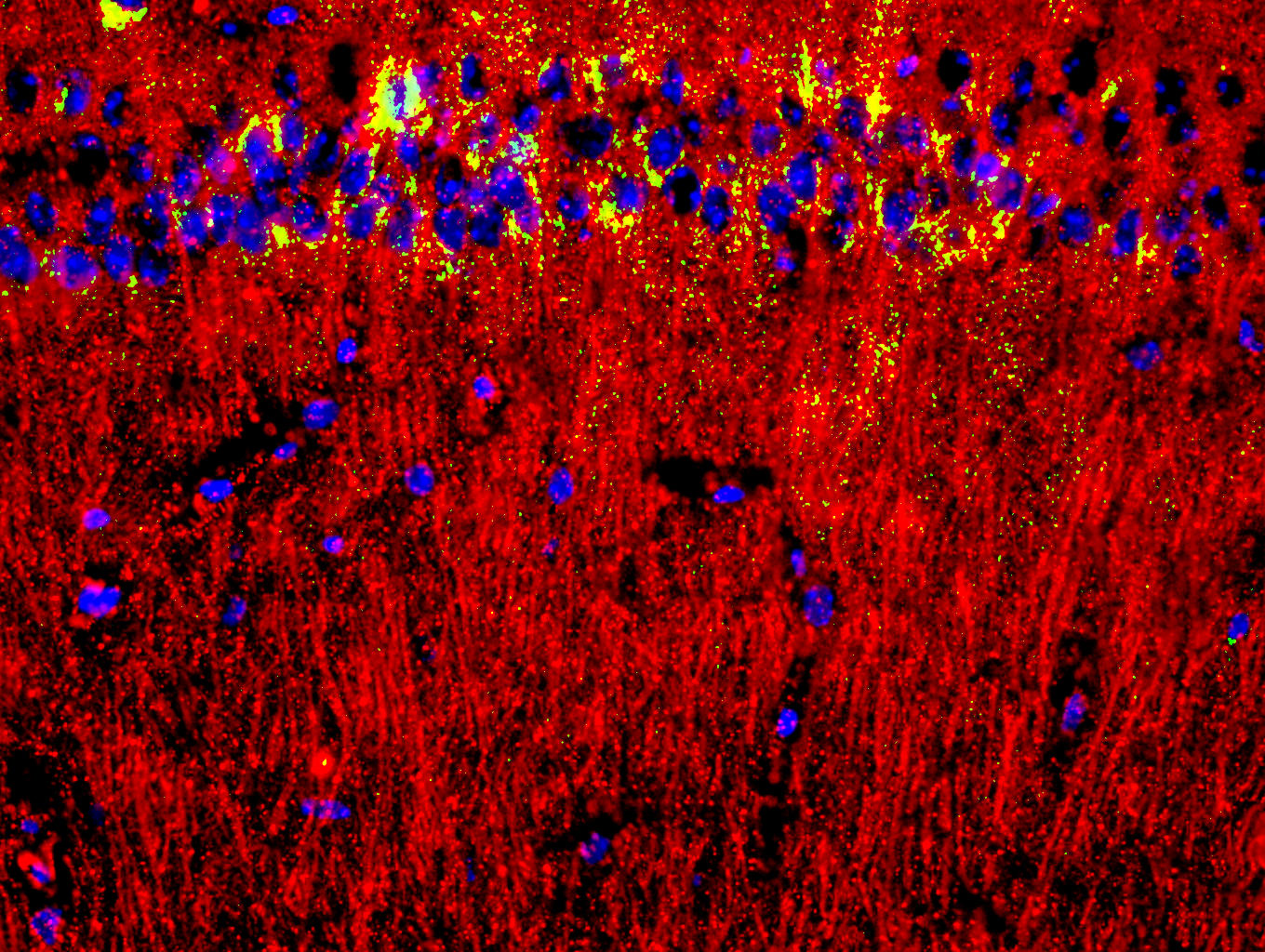

Supplement: Supplementary file 3 — Source data Fig. 1 [file 44321_2026_422_MOESM3_ESM.zip › Figure 1/1G/dbdb/Merge.tif]

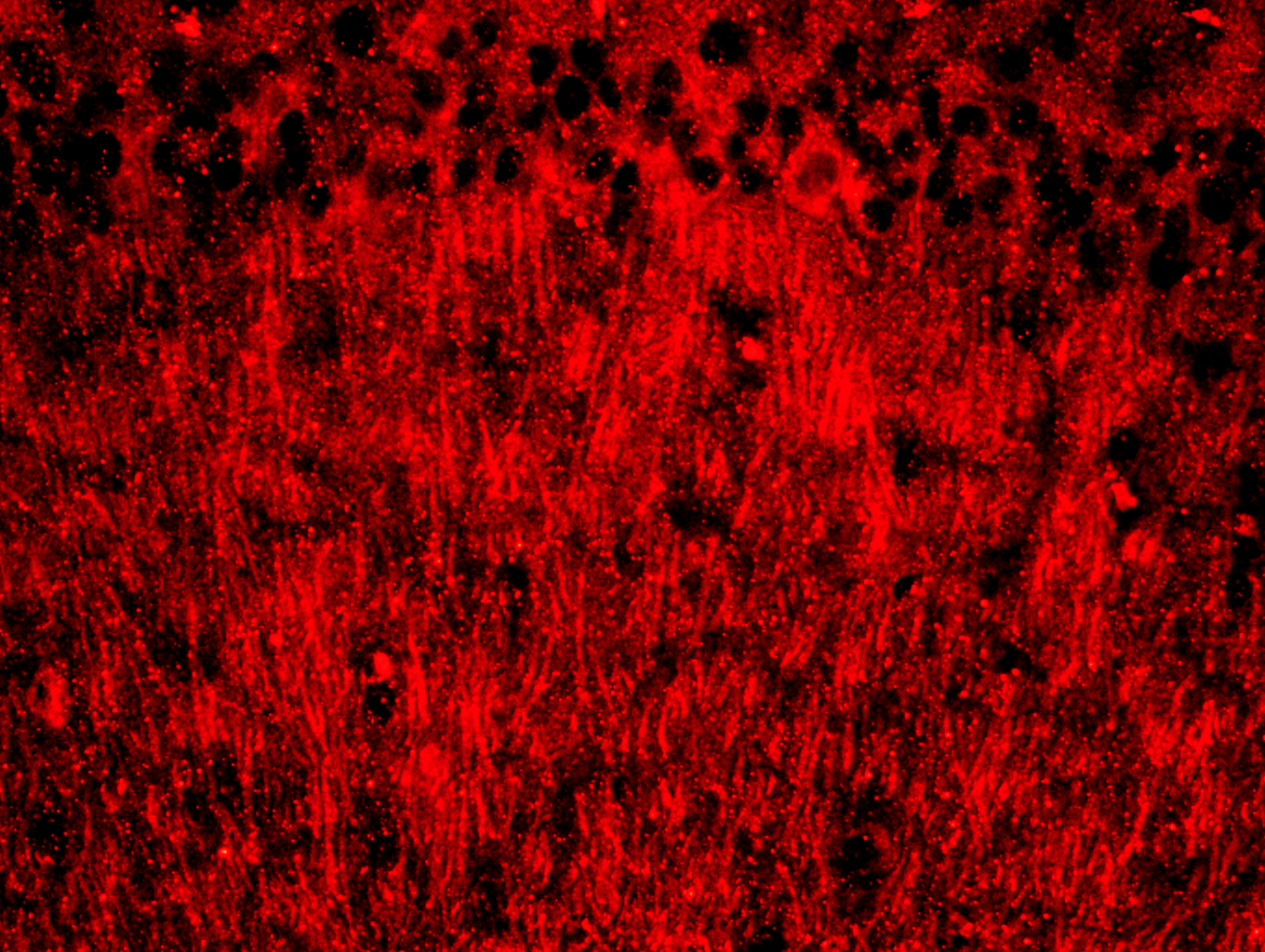

Supplement: Supplementary file 3 — Source data Fig. 1 [file 44321_2026_422_MOESM3_ESM.zip › Figure 1/1G/dbm/Map-2.tif]

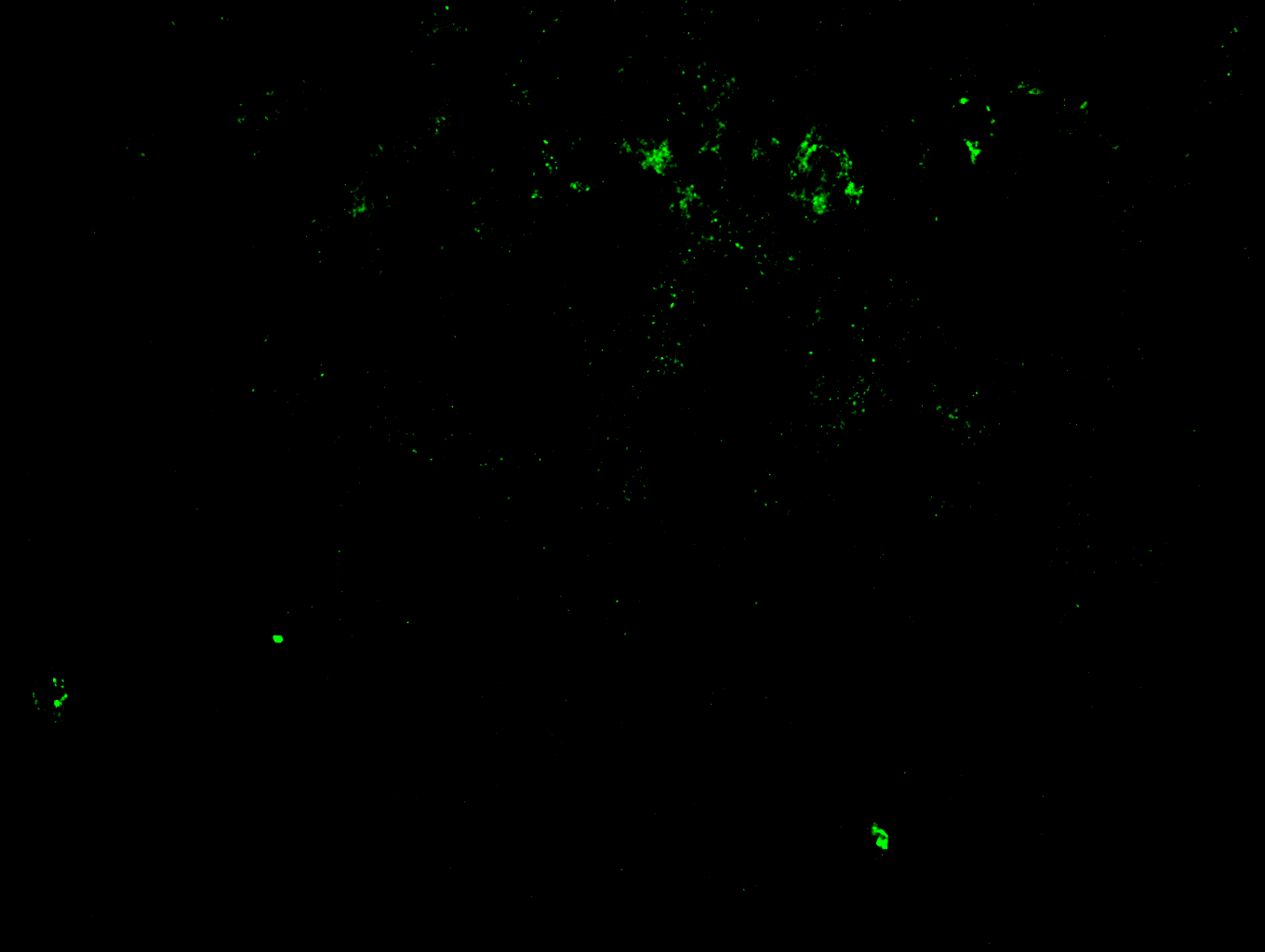

Supplement: Supplementary file 3 — Source data Fig. 1 [file 44321_2026_422_MOESM3_ESM.zip › Figure 1/1G/dbm/Lrpprc K223la.tif]

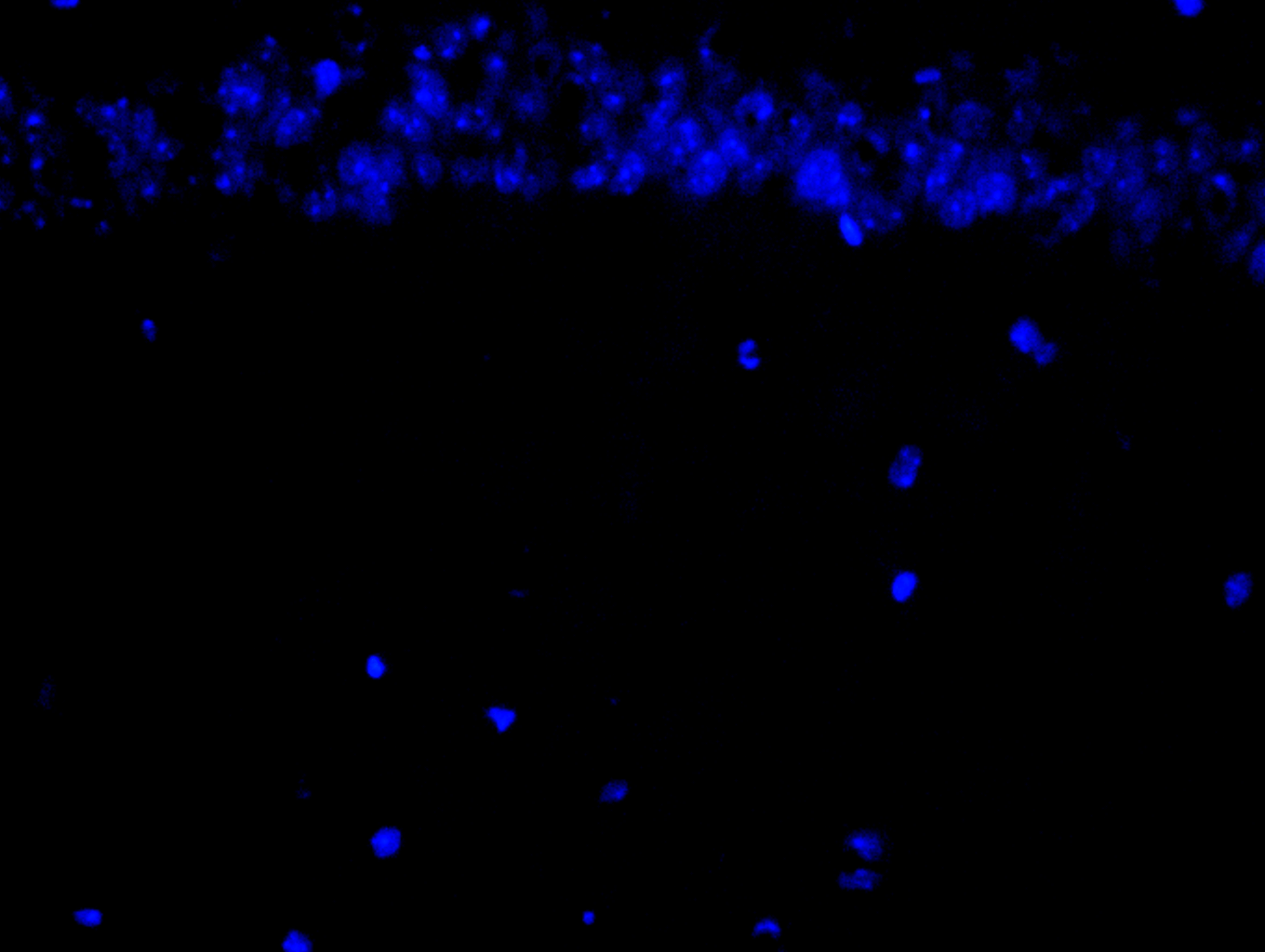

Supplement: Supplementary file 3 — Source data Fig. 1 [file 44321_2026_422_MOESM3_ESM.zip › Figure 1/1G/dbm/DAPI.tif]

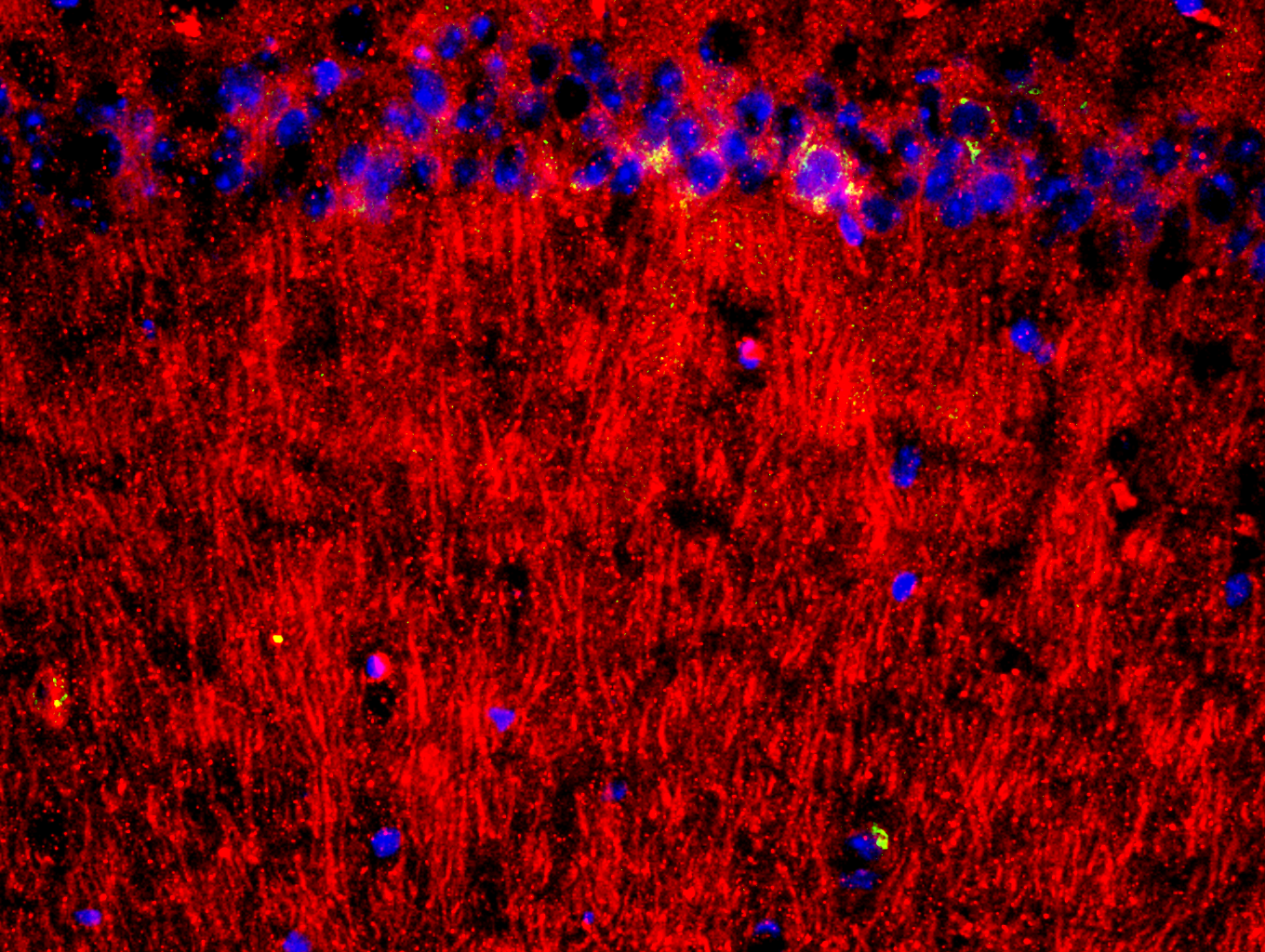

Supplement: Supplementary file 3 — Source data Fig. 1 [file 44321_2026_422_MOESM3_ESM.zip › Figure 1/1G/dbm/Merge.tif]

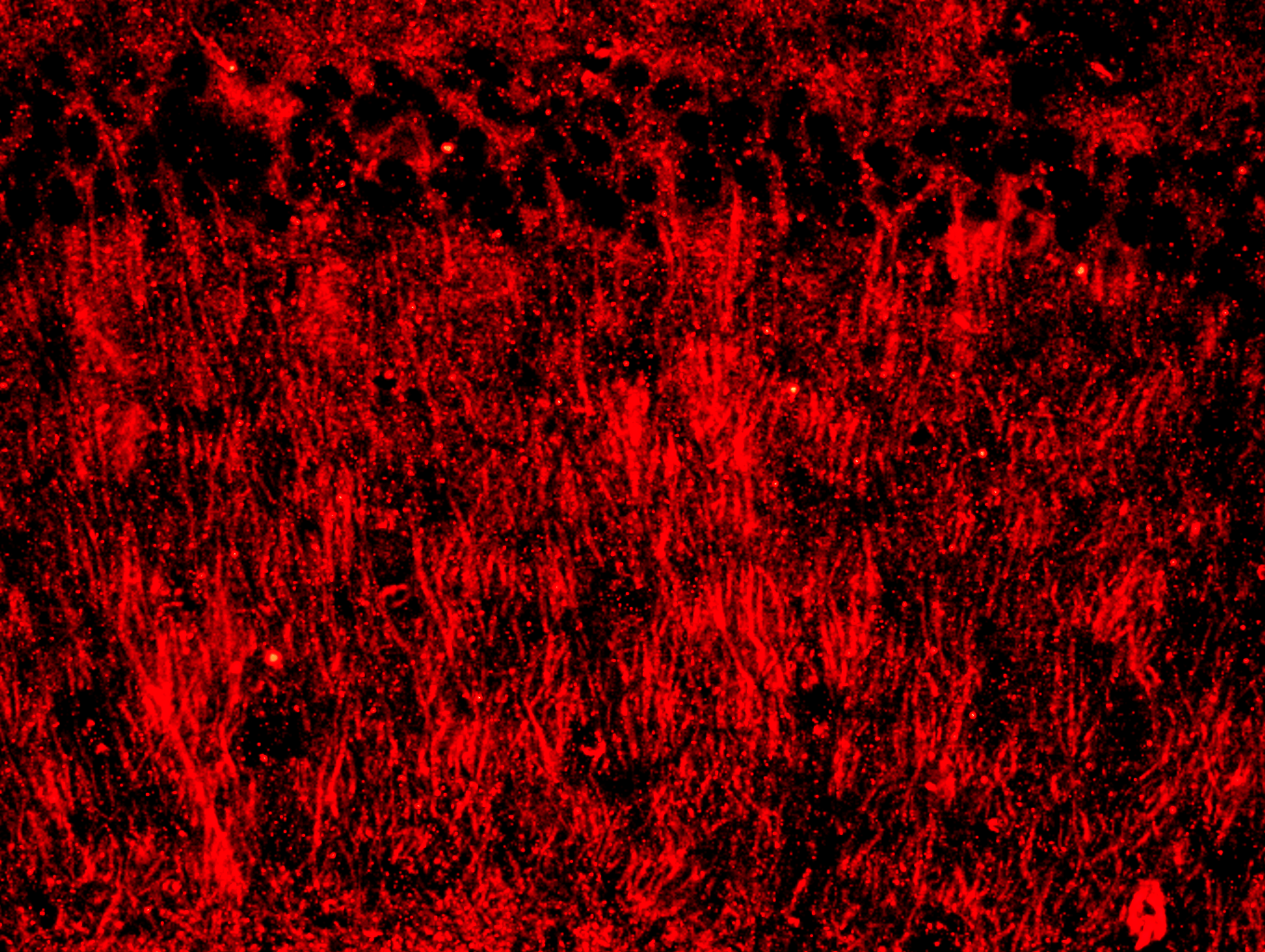

Supplement: Supplementary file 3 — Source data Fig. 1 [file 44321_2026_422_MOESM3_ESM.zip › Figure 1/1I/Ctr/Map-2.tif]

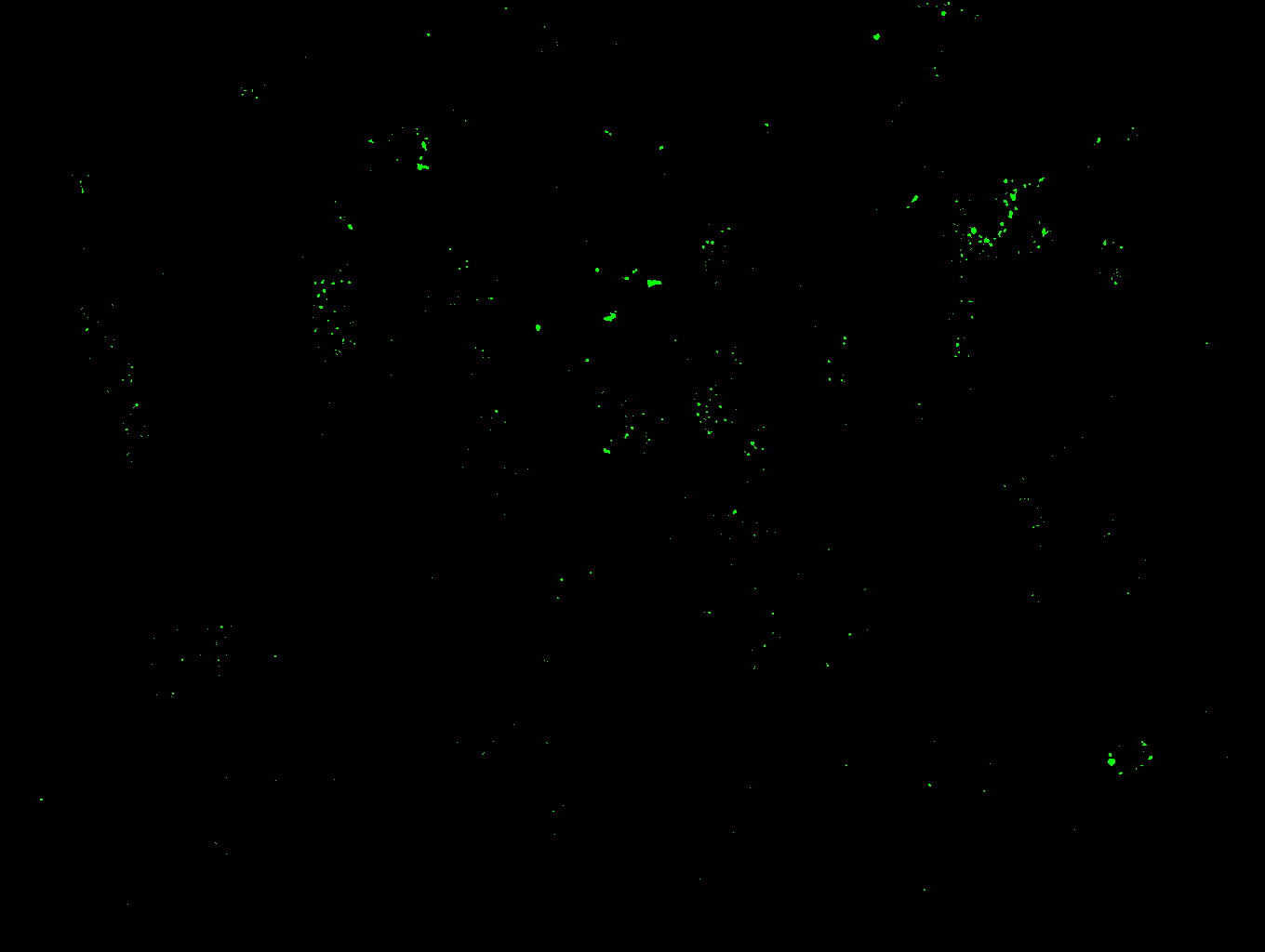

Supplement: Supplementary file 3 — Source data Fig. 1 [file 44321_2026_422_MOESM3_ESM.zip › Figure 1/1I/Ctr/Lrpprc K223la.tif]

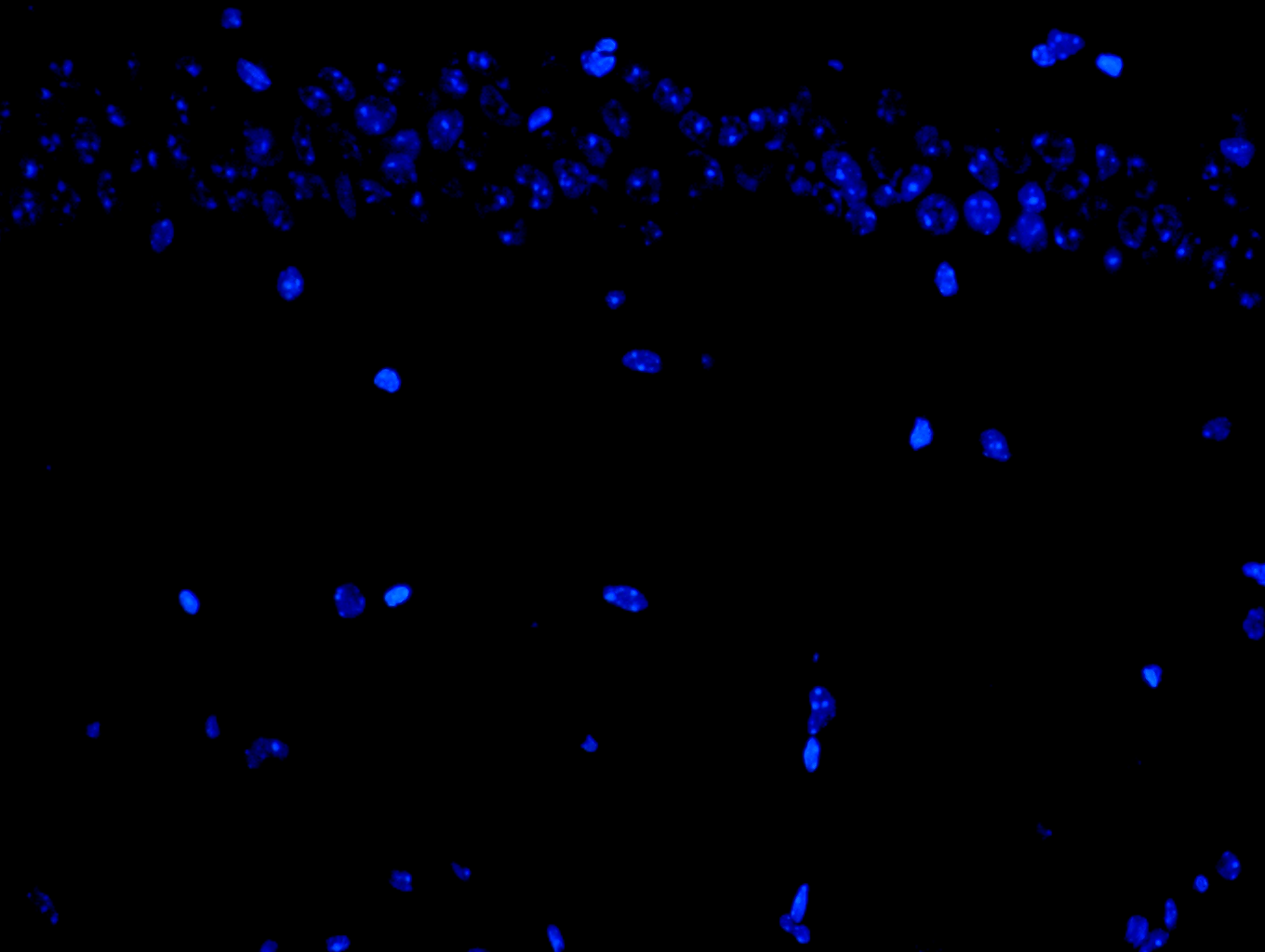

Supplement: Supplementary file 3 — Source data Fig. 1 [file 44321_2026_422_MOESM3_ESM.zip › Figure 1/1I/Ctr/DAPI.tif]

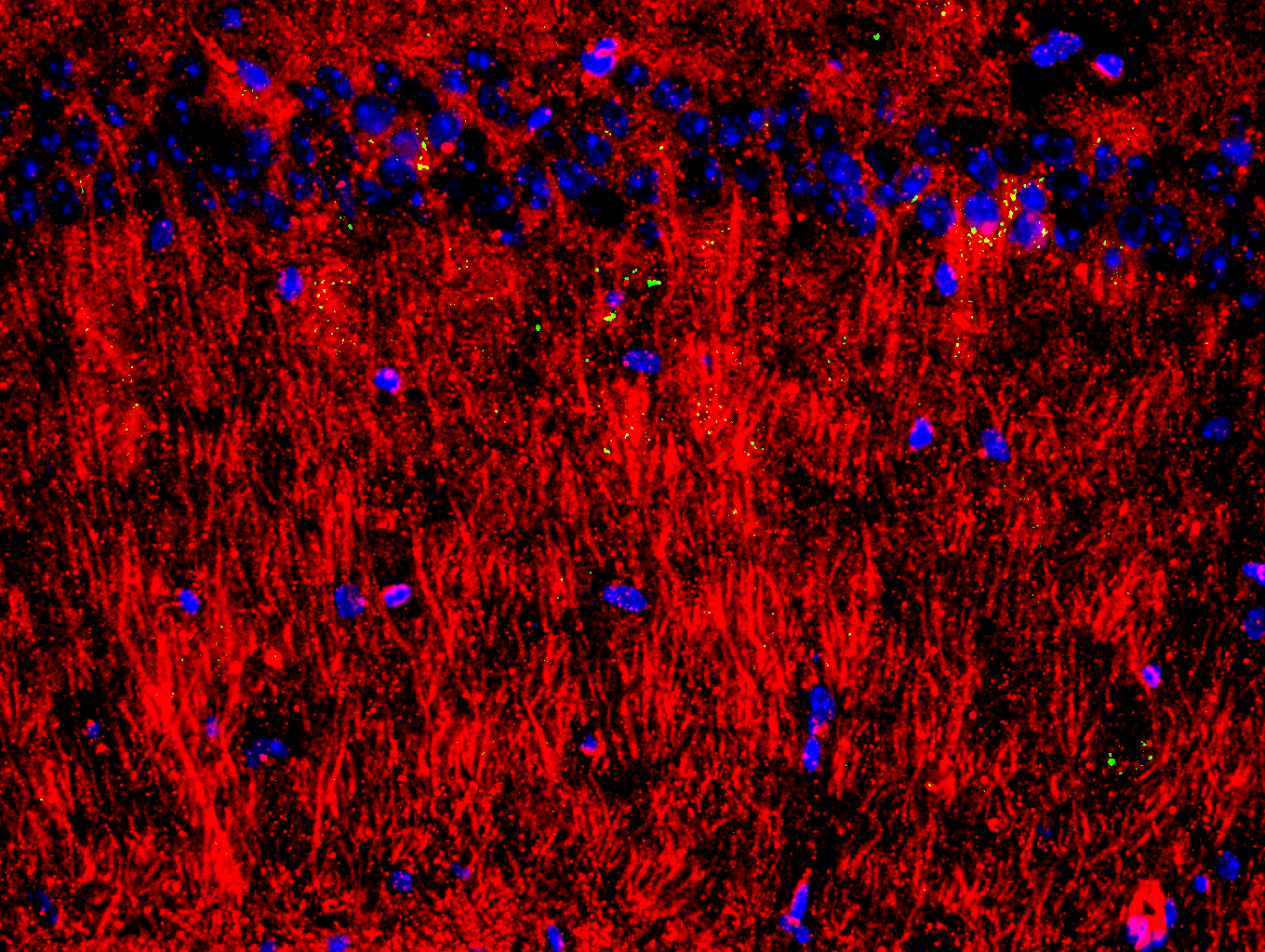

Supplement: Supplementary file 3 — Source data Fig. 1 [file 44321_2026_422_MOESM3_ESM.zip › Figure 1/1I/Ctr/Merge.tif]

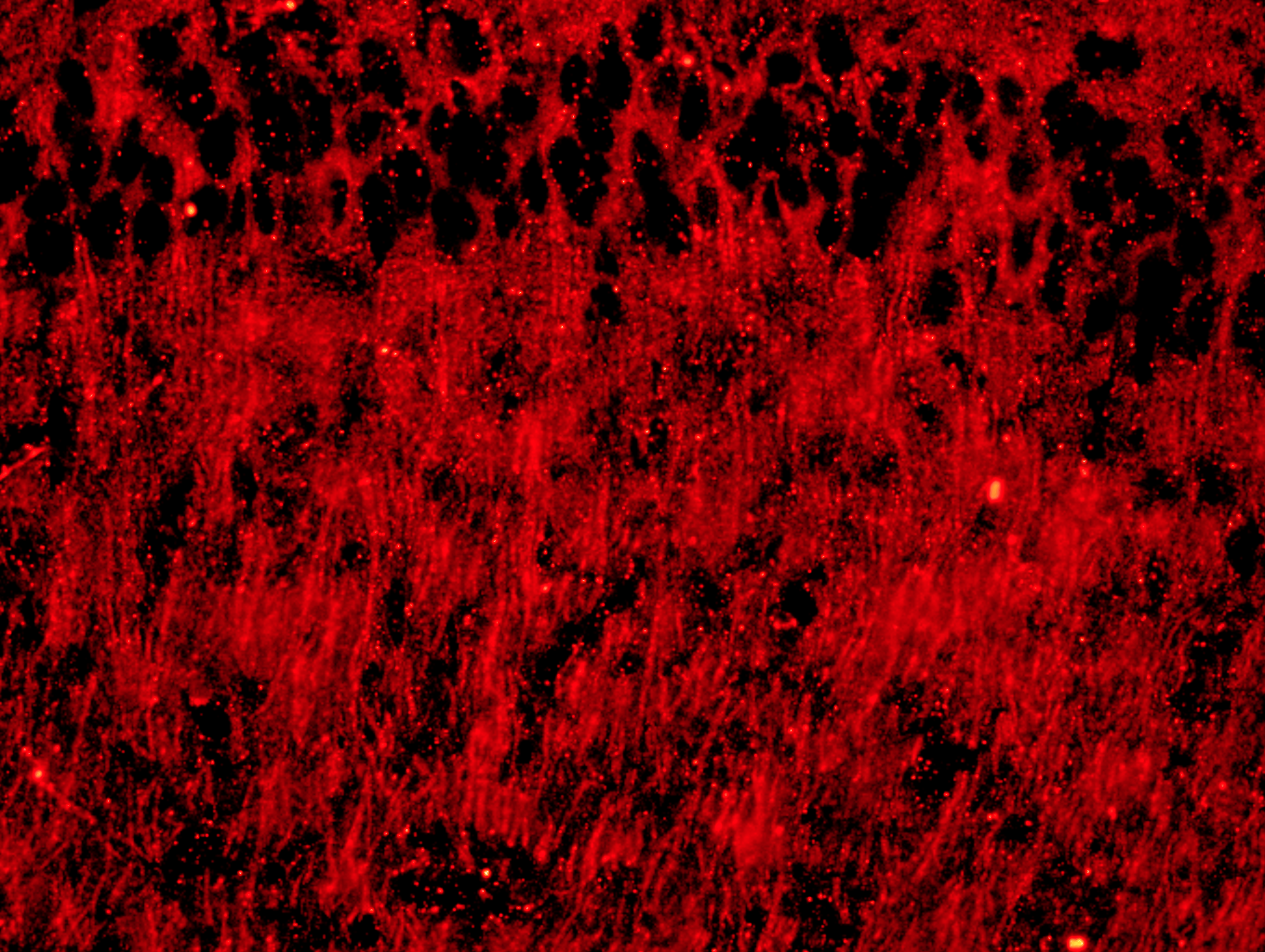

Supplement: Supplementary file 3 — Source data Fig. 1 [file 44321_2026_422_MOESM3_ESM.zip › Figure 1/1I/STZ/Map-2.tif]

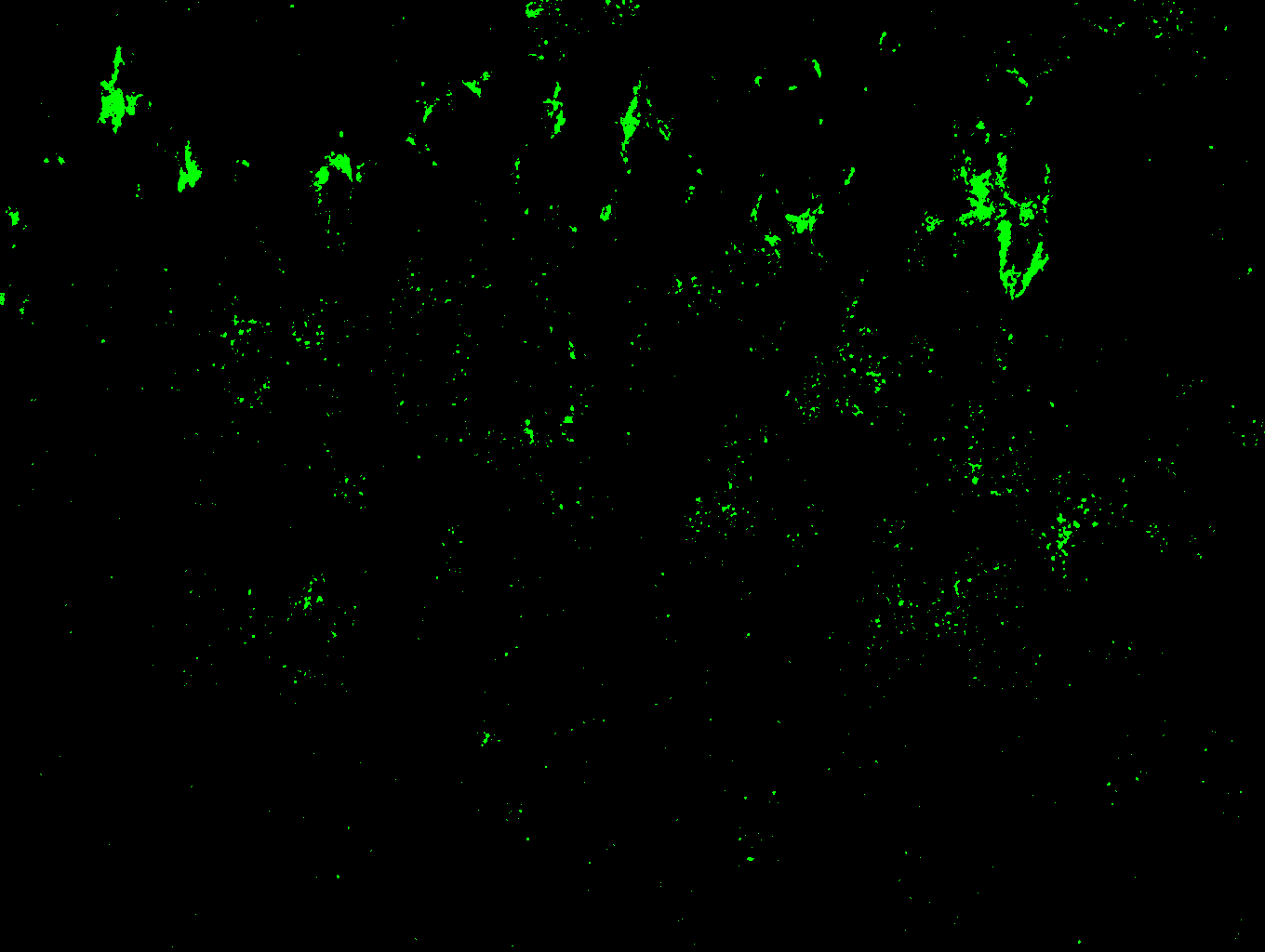

Supplement: Supplementary file 3 — Source data Fig. 1 [file 44321_2026_422_MOESM3_ESM.zip › Figure 1/1I/STZ/Lrpprc K223la.tif]

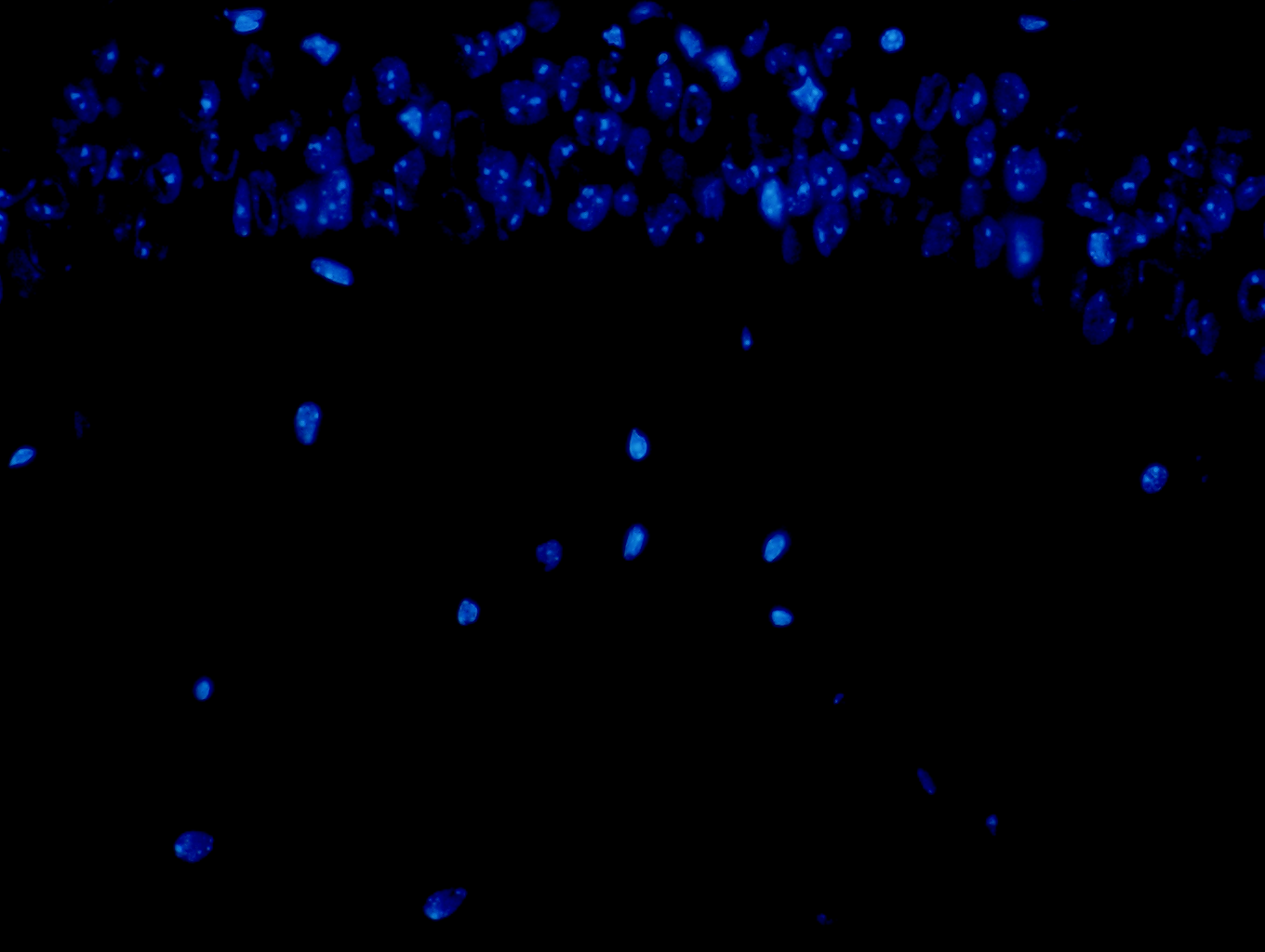

Supplement: Supplementary file 3 — Source data Fig. 1 [file 44321_2026_422_MOESM3_ESM.zip › Figure 1/1I/STZ/DAPI.tif]

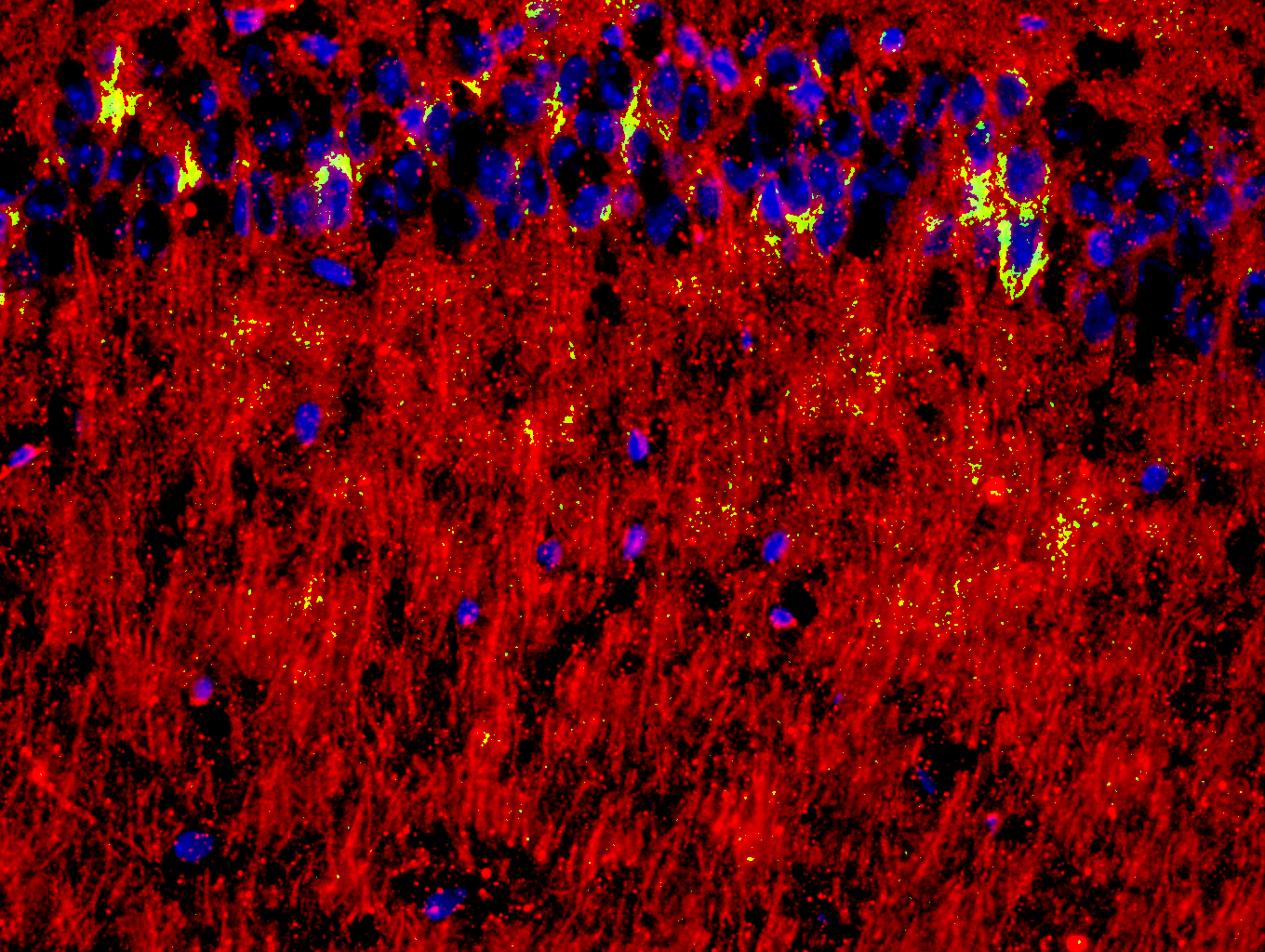

Supplement: Supplementary file 3 — Source data Fig. 1 [file 44321_2026_422_MOESM3_ESM.zip › Figure 1/1I/STZ/Merge.tif]

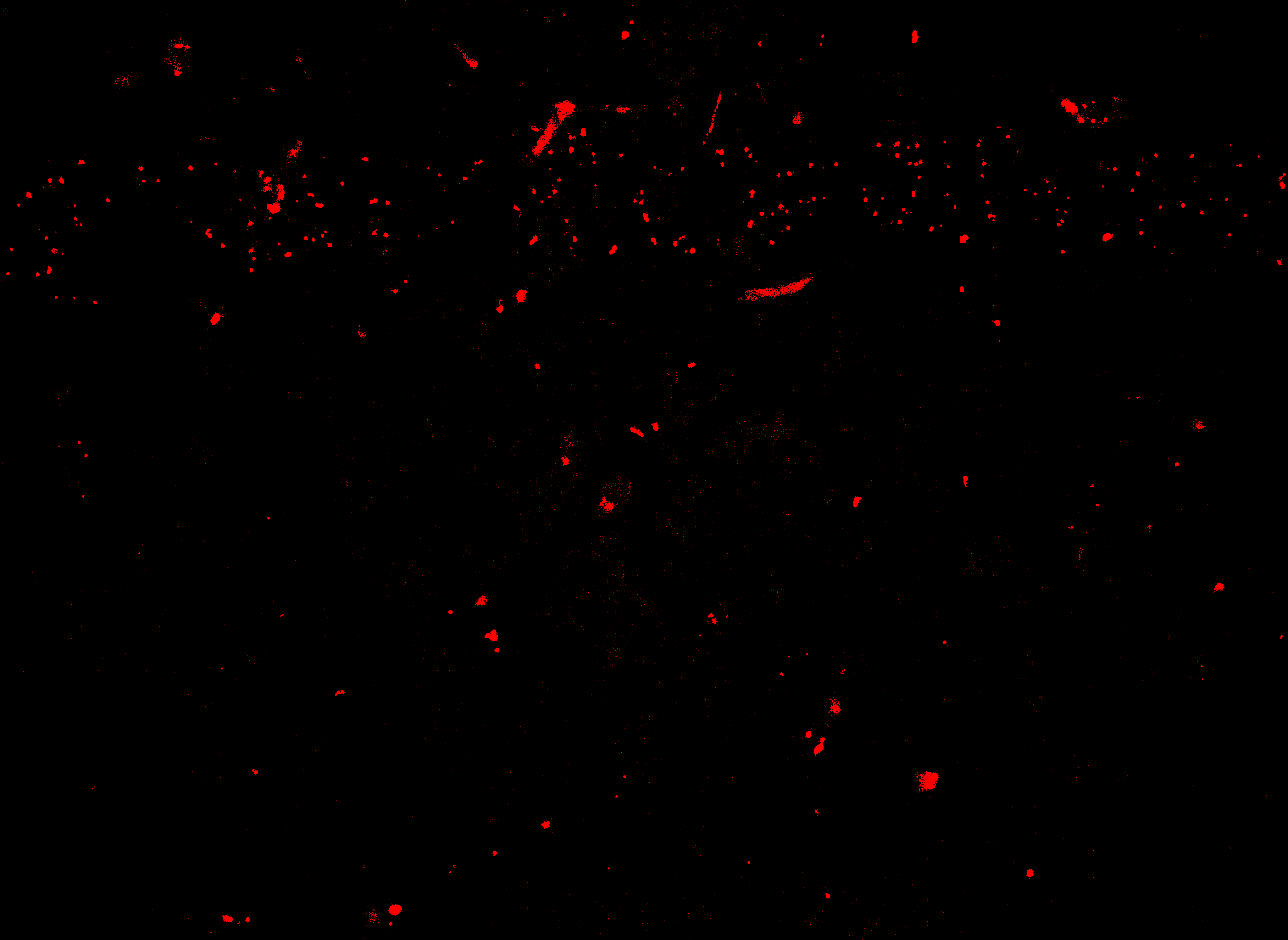

Supplement: Supplementary file 4 — Source data Fig. 2 [file 44321_2026_422_MOESM4_ESM.zip › Figure 2/2A/dbdb+AAV-Ctr/Tunel.tif]

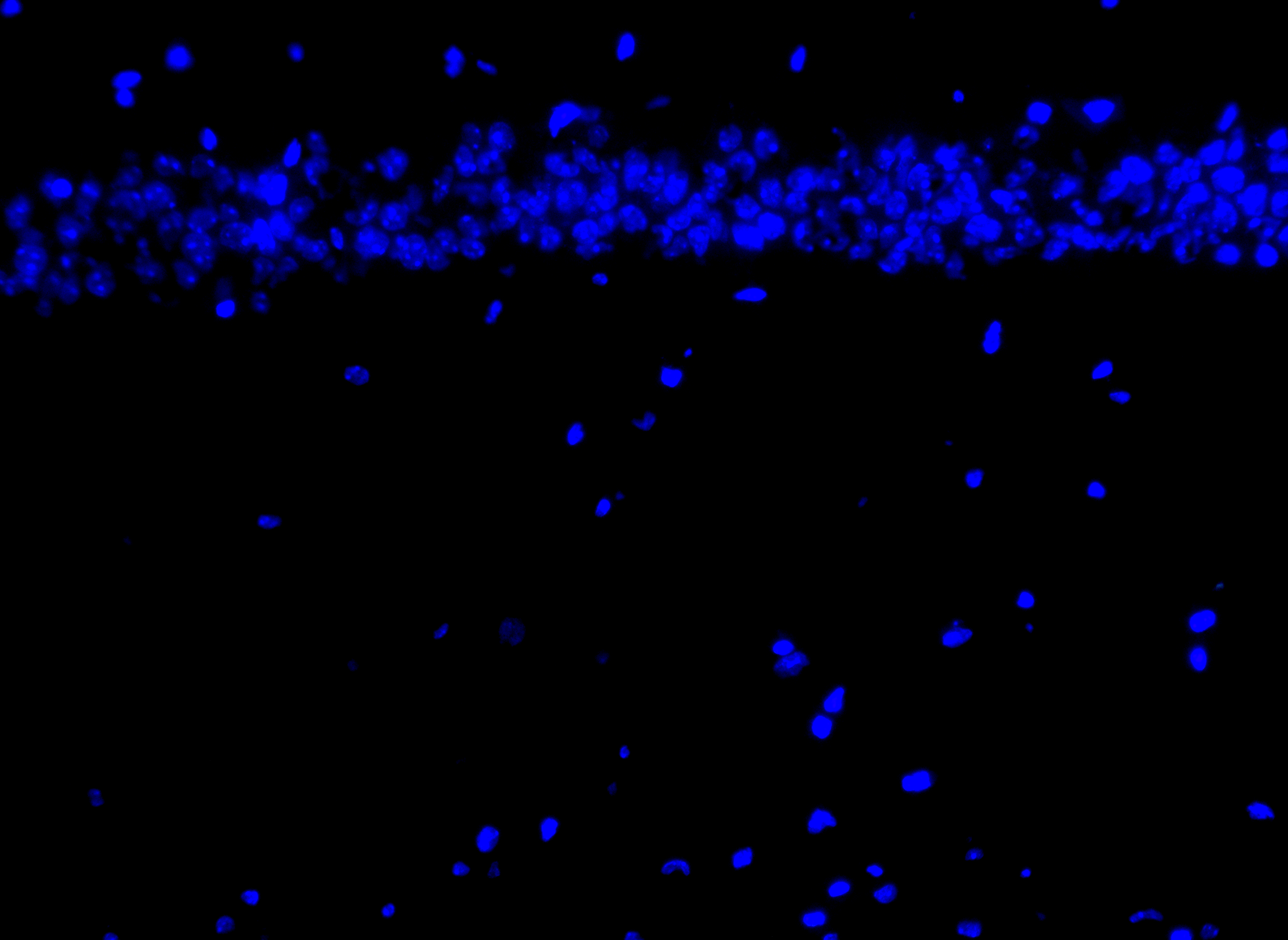

Supplement: Supplementary file 4 — Source data Fig. 2 [file 44321_2026_422_MOESM4_ESM.zip › Figure 2/2A/dbdb+AAV-Ctr/DAPI.tif]

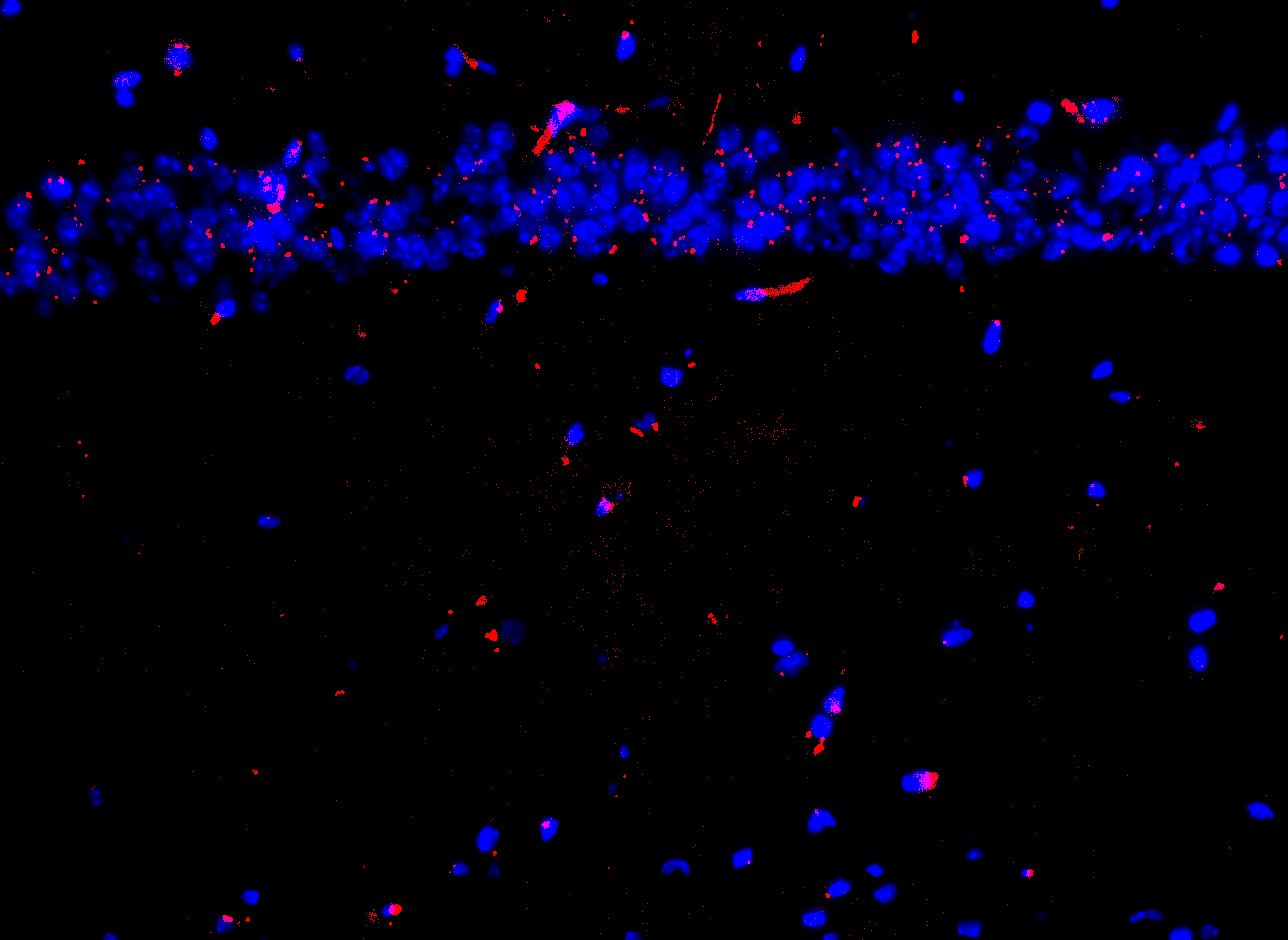

Supplement: Supplementary file 4 — Source data Fig. 2 [file 44321_2026_422_MOESM4_ESM.zip › Figure 2/2A/dbdb+AAV-Ctr/Merge.tif]

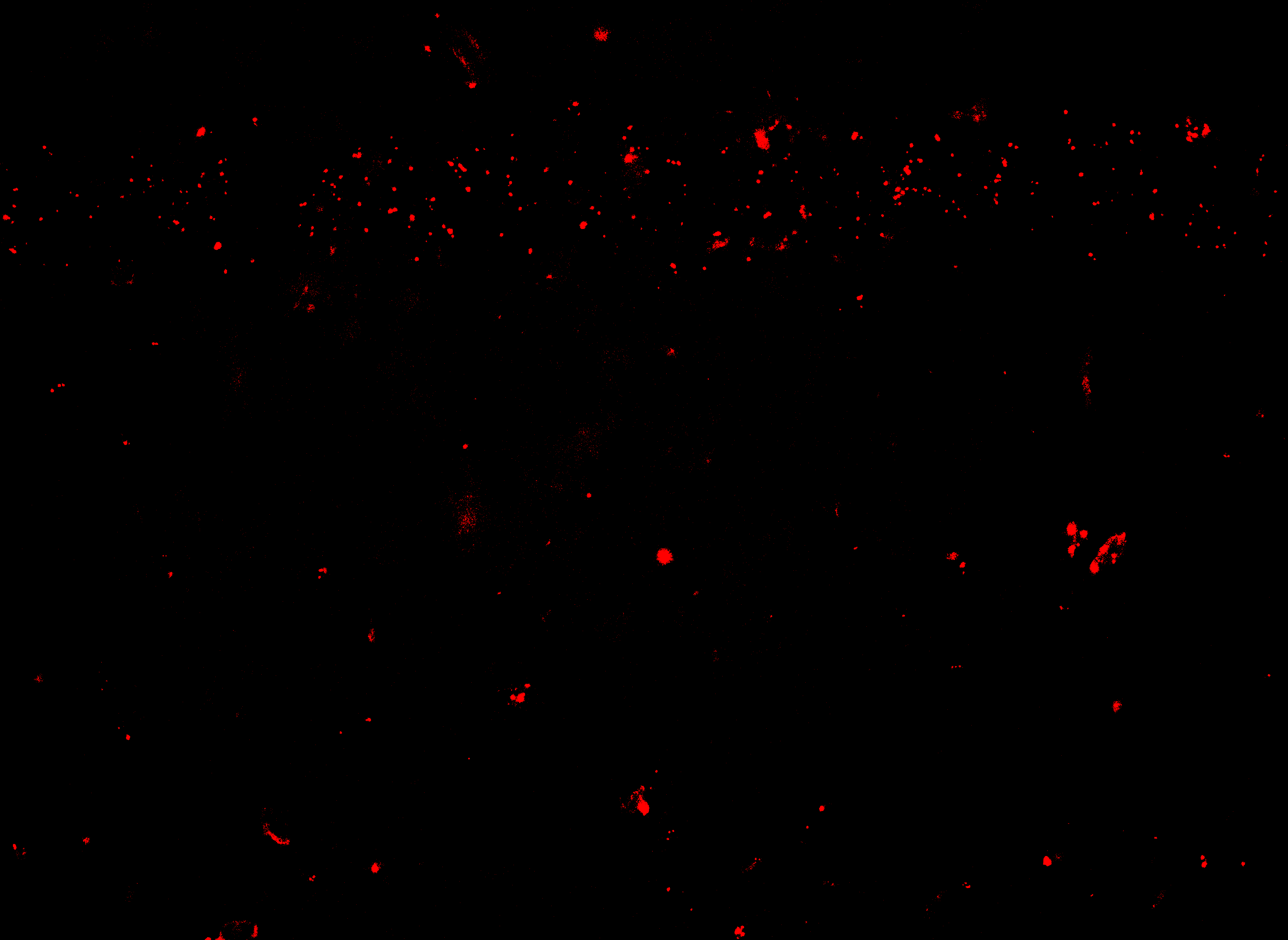

Supplement: Supplementary file 4 — Source data Fig. 2 [file 44321_2026_422_MOESM4_ESM.zip › Figure 2/2A/dbdb+AAV-Flag-Lrpprc-WT/Tunel.tif]

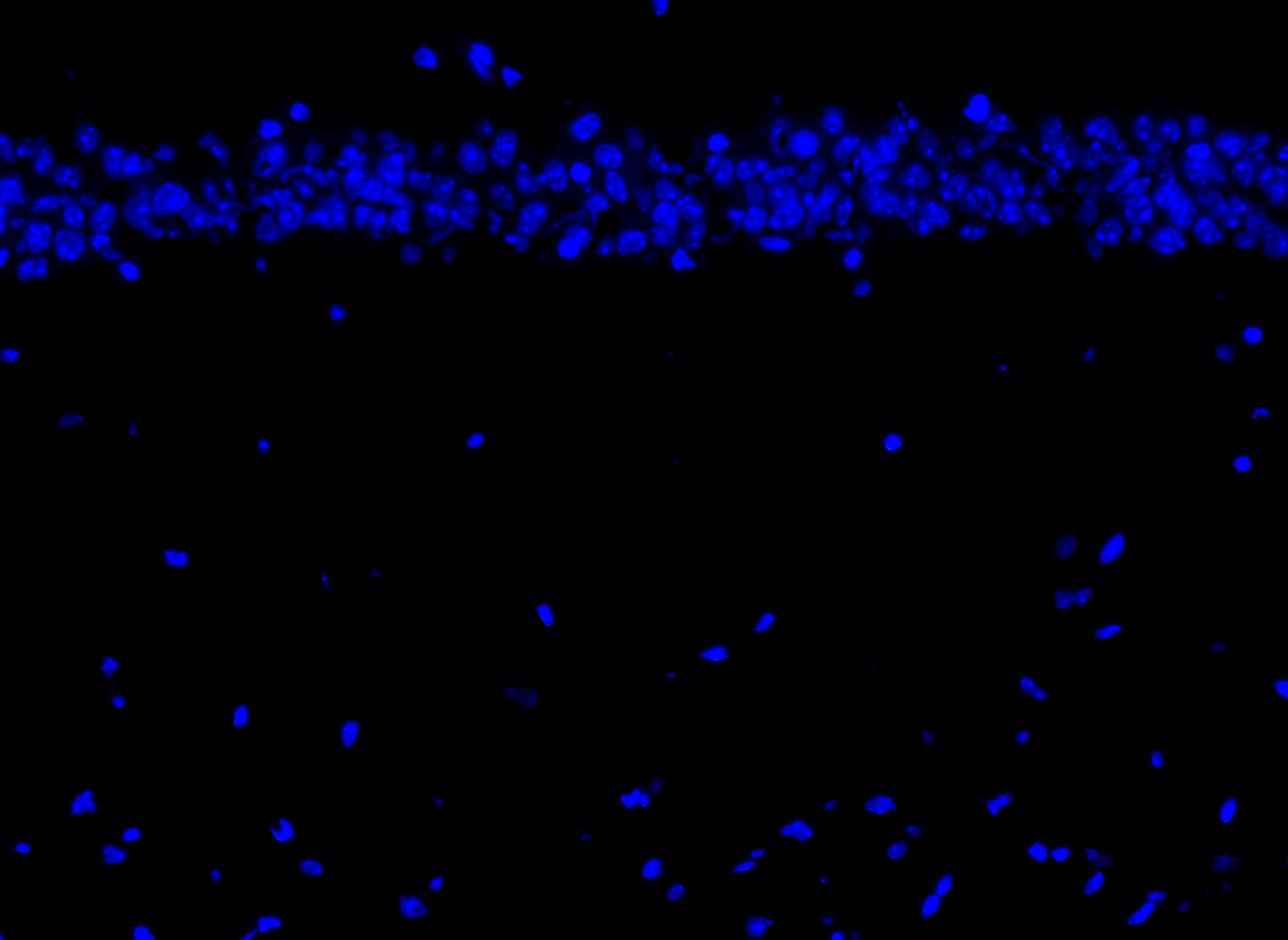

Supplement: Supplementary file 4 — Source data Fig. 2 [file 44321_2026_422_MOESM4_ESM.zip › Figure 2/2A/dbdb+AAV-Flag-Lrpprc-WT/DAPI.tif]

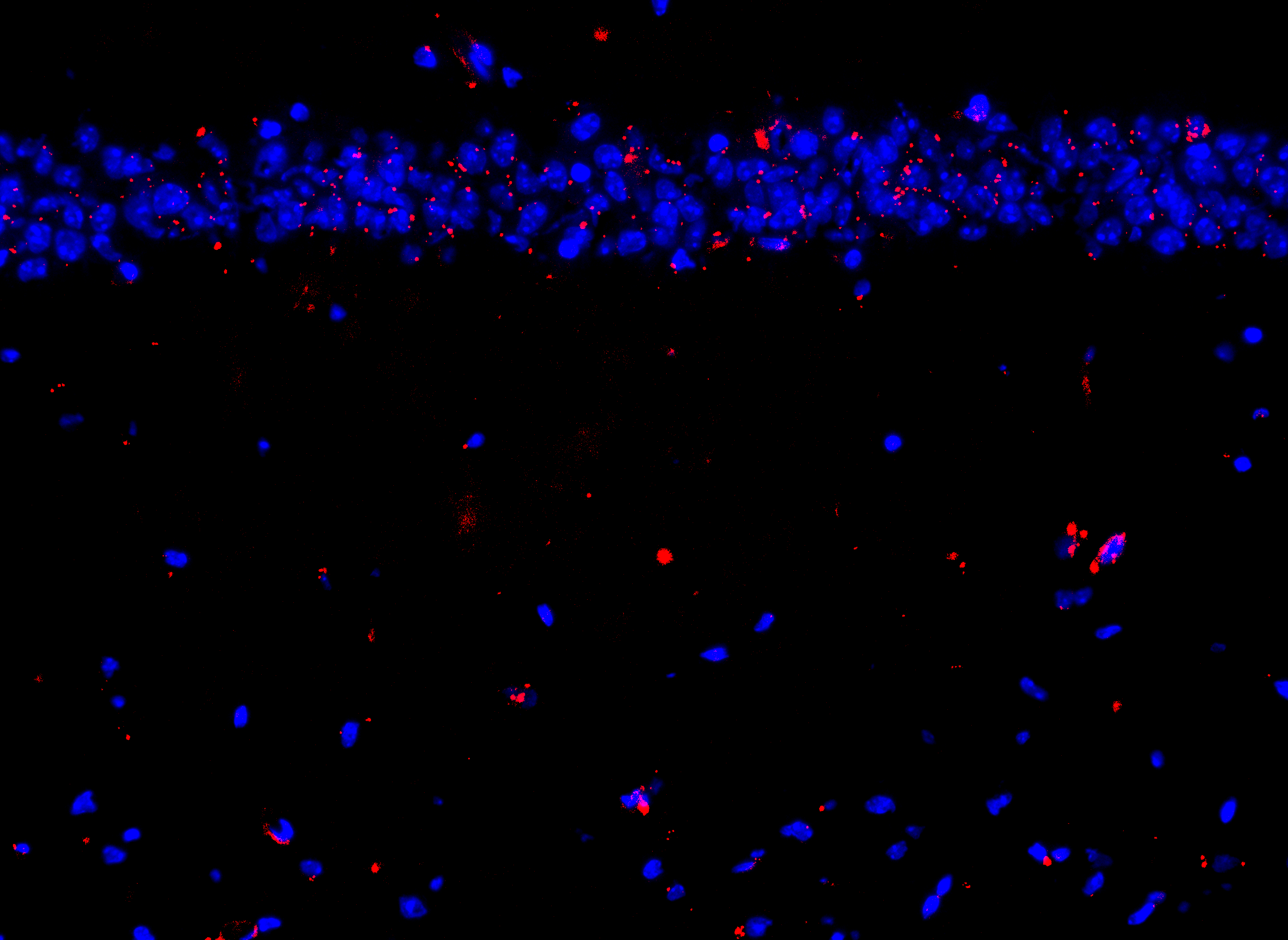

Supplement: Supplementary file 4 — Source data Fig. 2 [file 44321_2026_422_MOESM4_ESM.zip › Figure 2/2A/dbdb+AAV-Flag-Lrpprc-WT/Merge.tif]

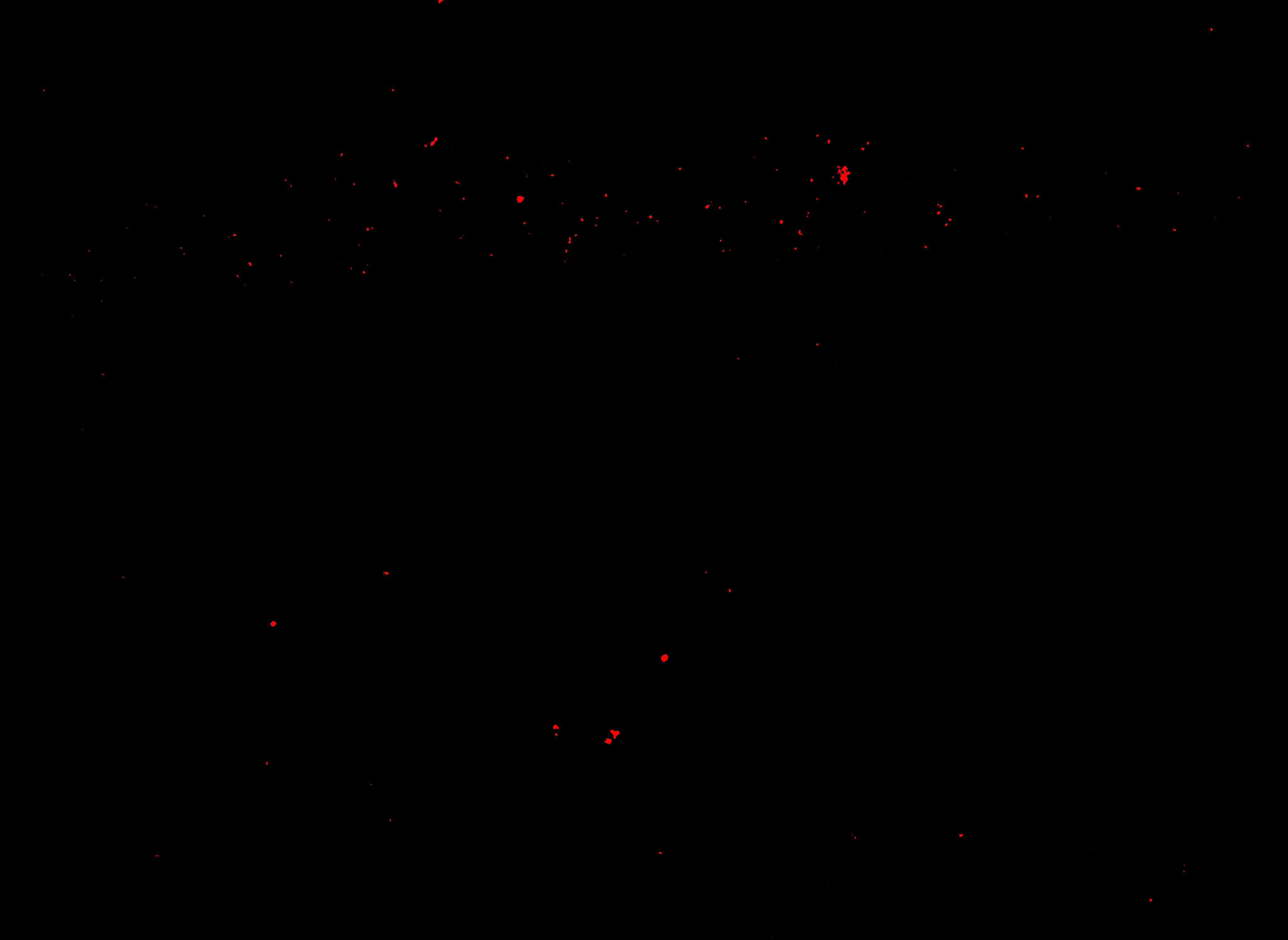

Supplement: Supplementary file 4 — Source data Fig. 2 [file 44321_2026_422_MOESM4_ESM.zip › Figure 2/2A/dbm+AAV-Flag-Lrpprc-WT/Tunel.tif]

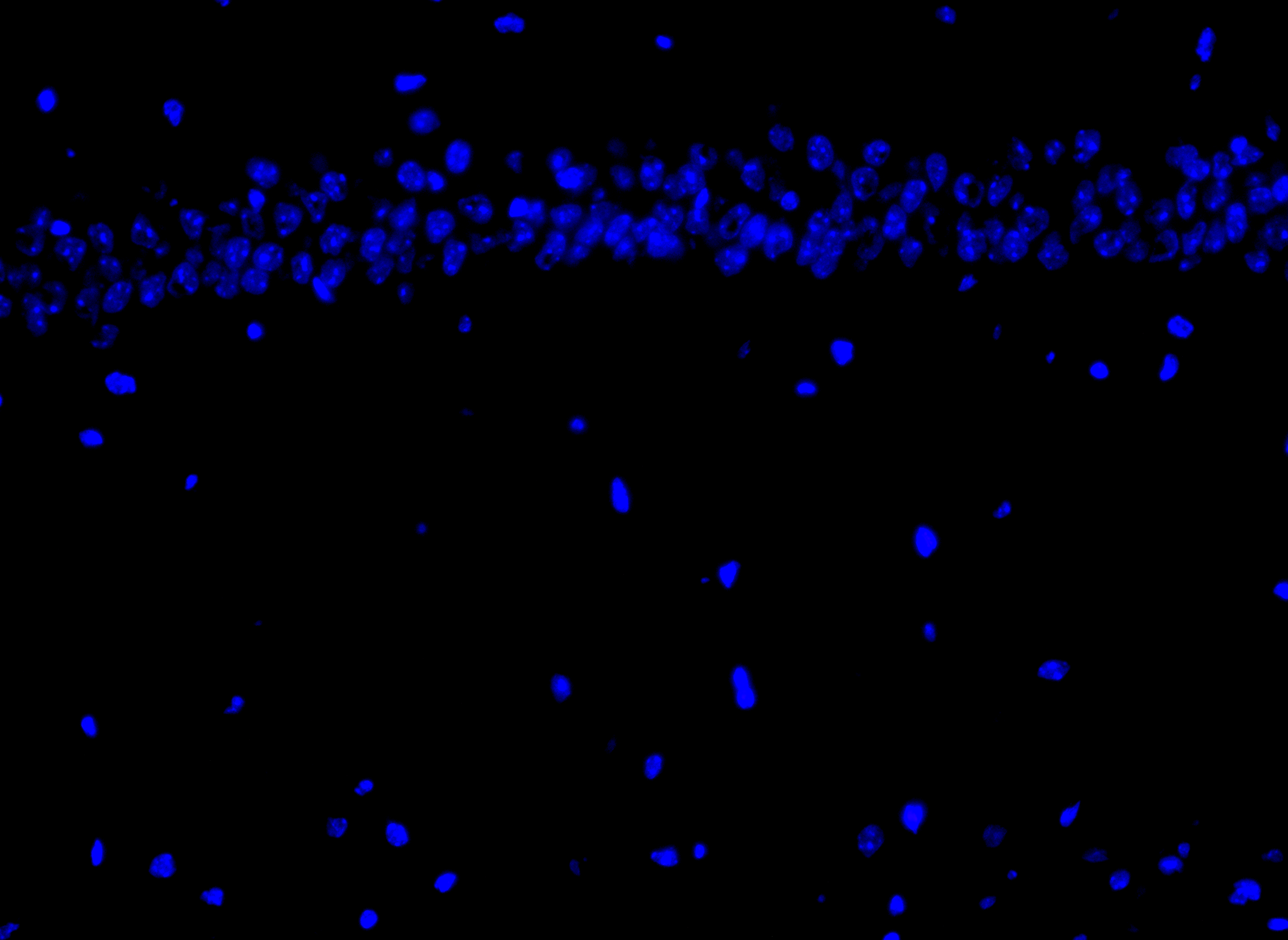

Supplement: Supplementary file 4 — Source data Fig. 2 [file 44321_2026_422_MOESM4_ESM.zip › Figure 2/2A/dbm+AAV-Flag-Lrpprc-WT/DAPI.tif]

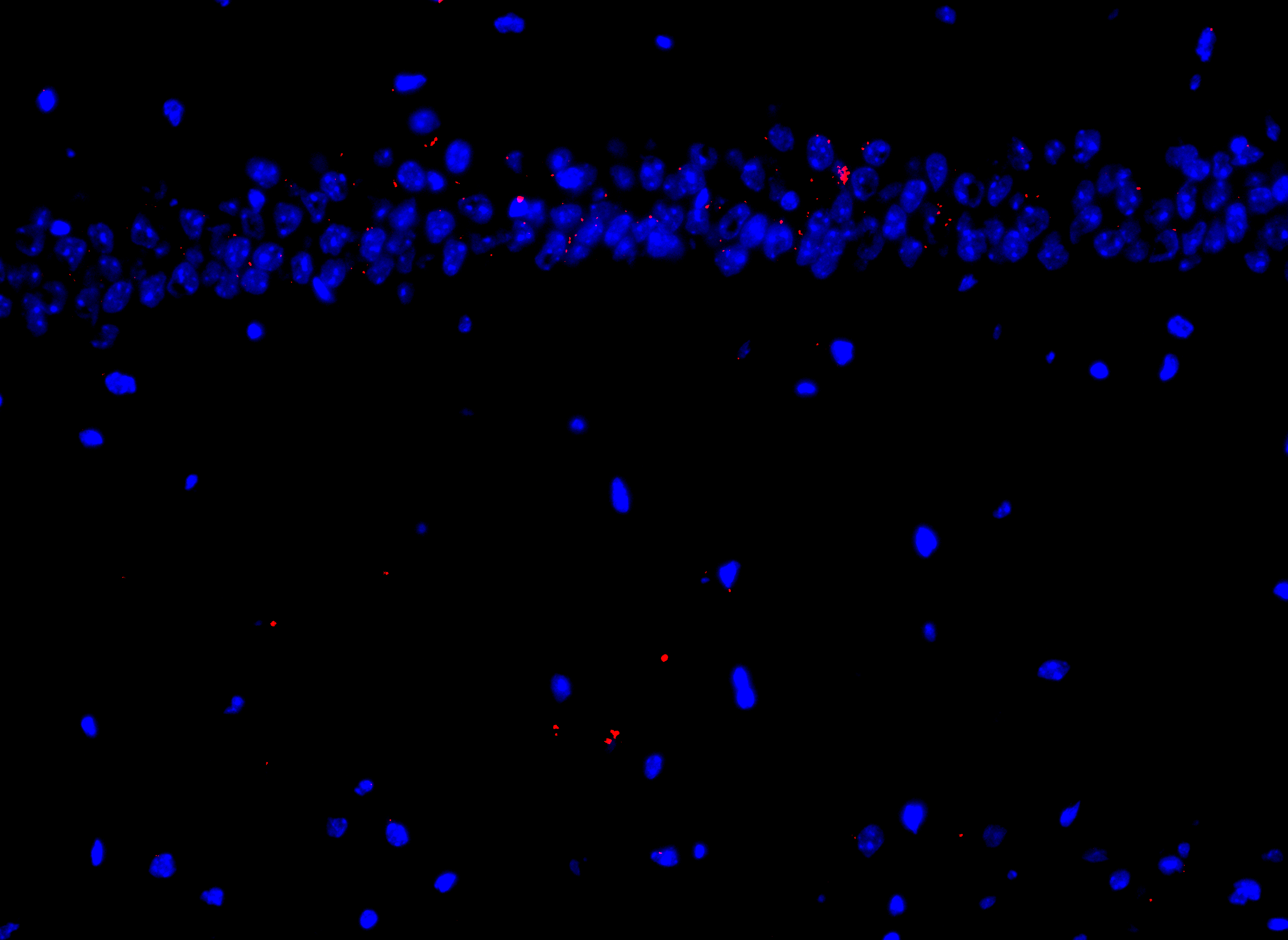

Supplement: Supplementary file 4 — Source data Fig. 2 [file 44321_2026_422_MOESM4_ESM.zip › Figure 2/2A/dbm+AAV-Flag-Lrpprc-WT/Merge.tif]

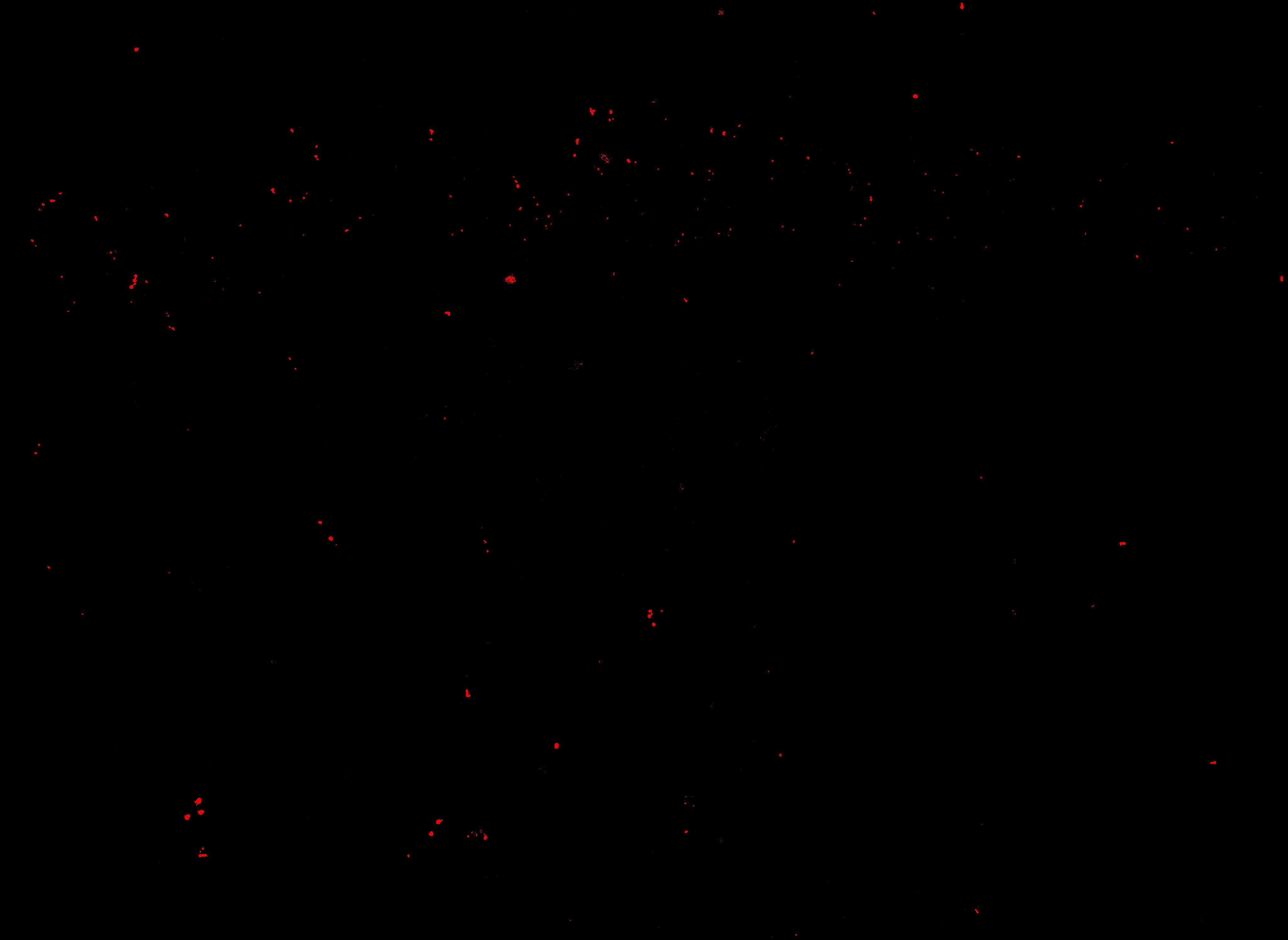

Supplement: Supplementary file 4 — Source data Fig. 2 [file 44321_2026_422_MOESM4_ESM.zip › Figure 2/2A/dbm+AAV-Flag-Lrpprc-K223R/Tunel.tif]

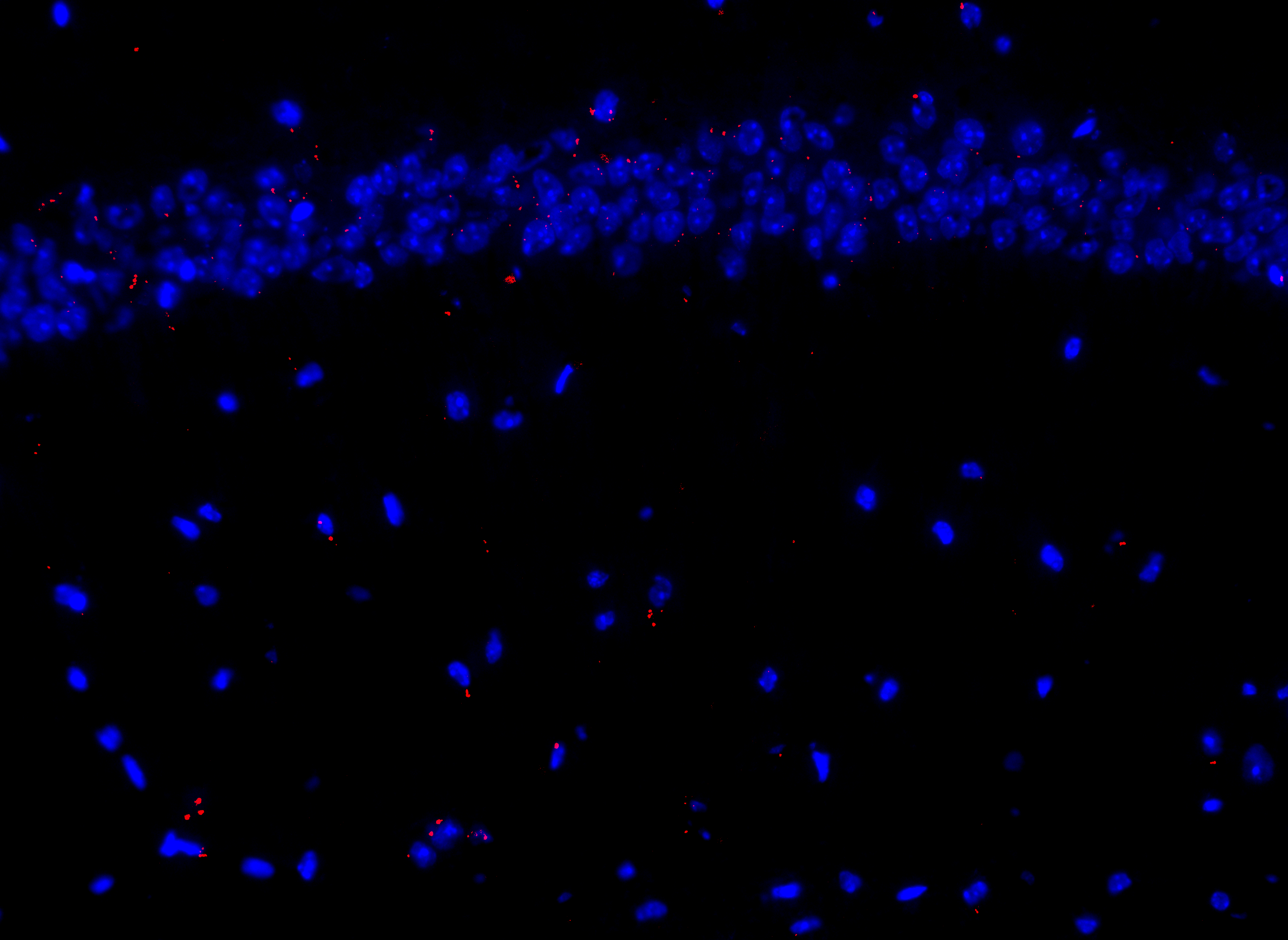

Supplement: Supplementary file 4 — Source data Fig. 2 [file 44321_2026_422_MOESM4_ESM.zip › Figure 2/2A/dbm+AAV-Flag-Lrpprc-K223R/d1+r1.tif]

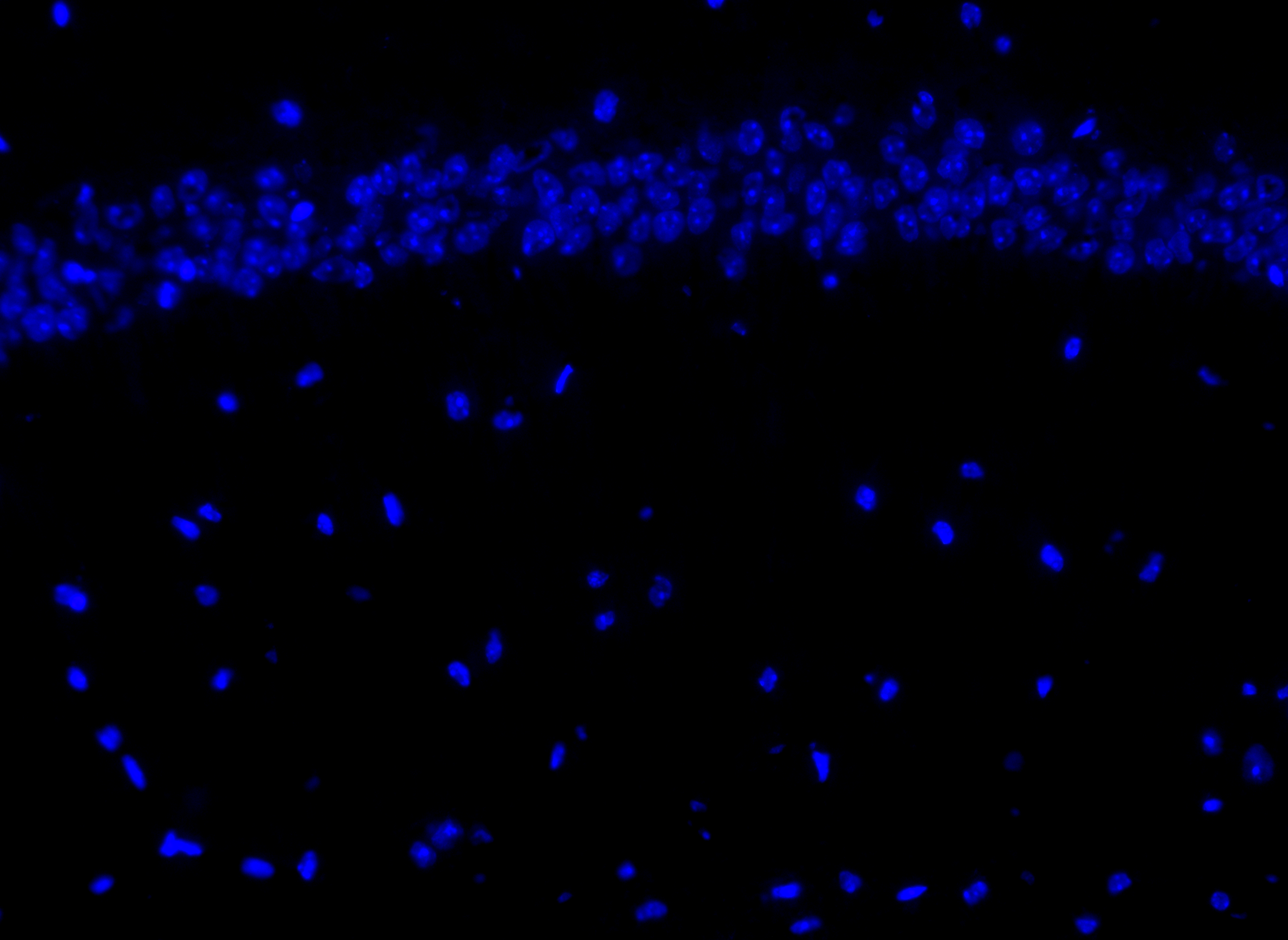

Supplement: Supplementary file 4 — Source data Fig. 2 [file 44321_2026_422_MOESM4_ESM.zip › Figure 2/2A/dbm+AAV-Flag-Lrpprc-K223R/Merge.tif]

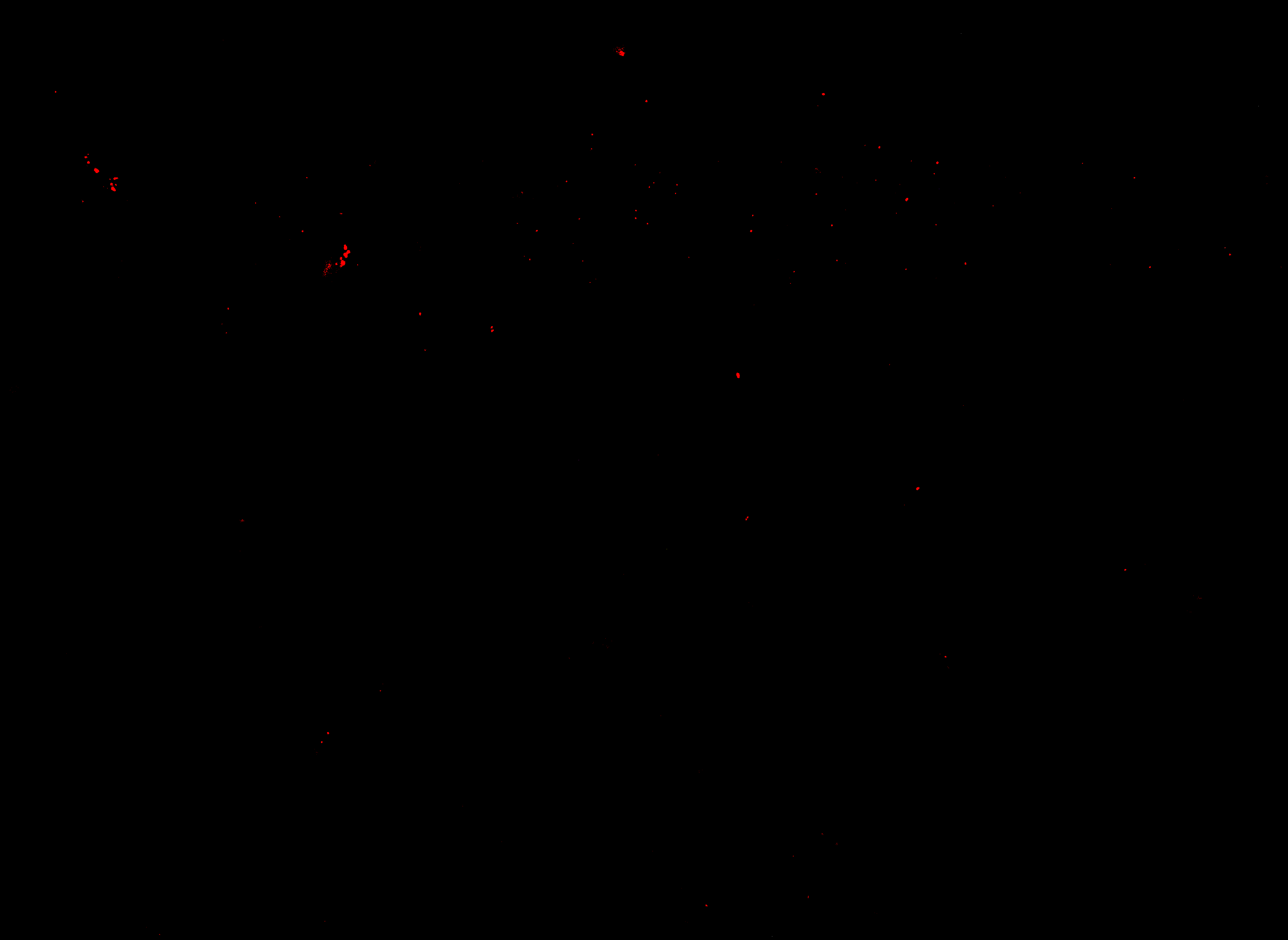

Supplement: Supplementary file 4 — Source data Fig. 2 [file 44321_2026_422_MOESM4_ESM.zip › Figure 2/2A/dbm+AAV-Ctr/Tunel.tif]

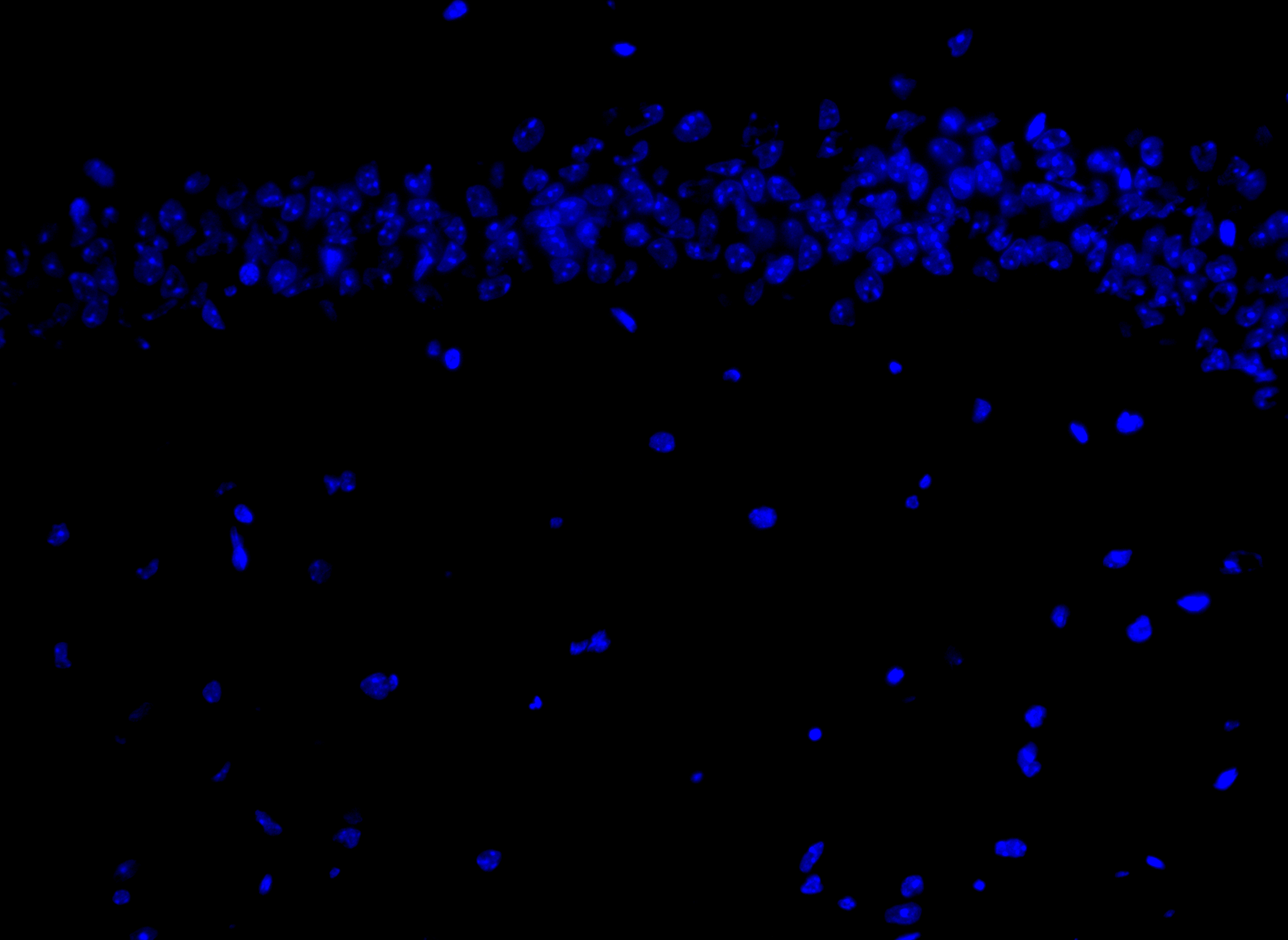

Supplement: Supplementary file 4 — Source data Fig. 2 [file 44321_2026_422_MOESM4_ESM.zip › Figure 2/2A/dbm+AAV-Ctr/DAPI.tif]

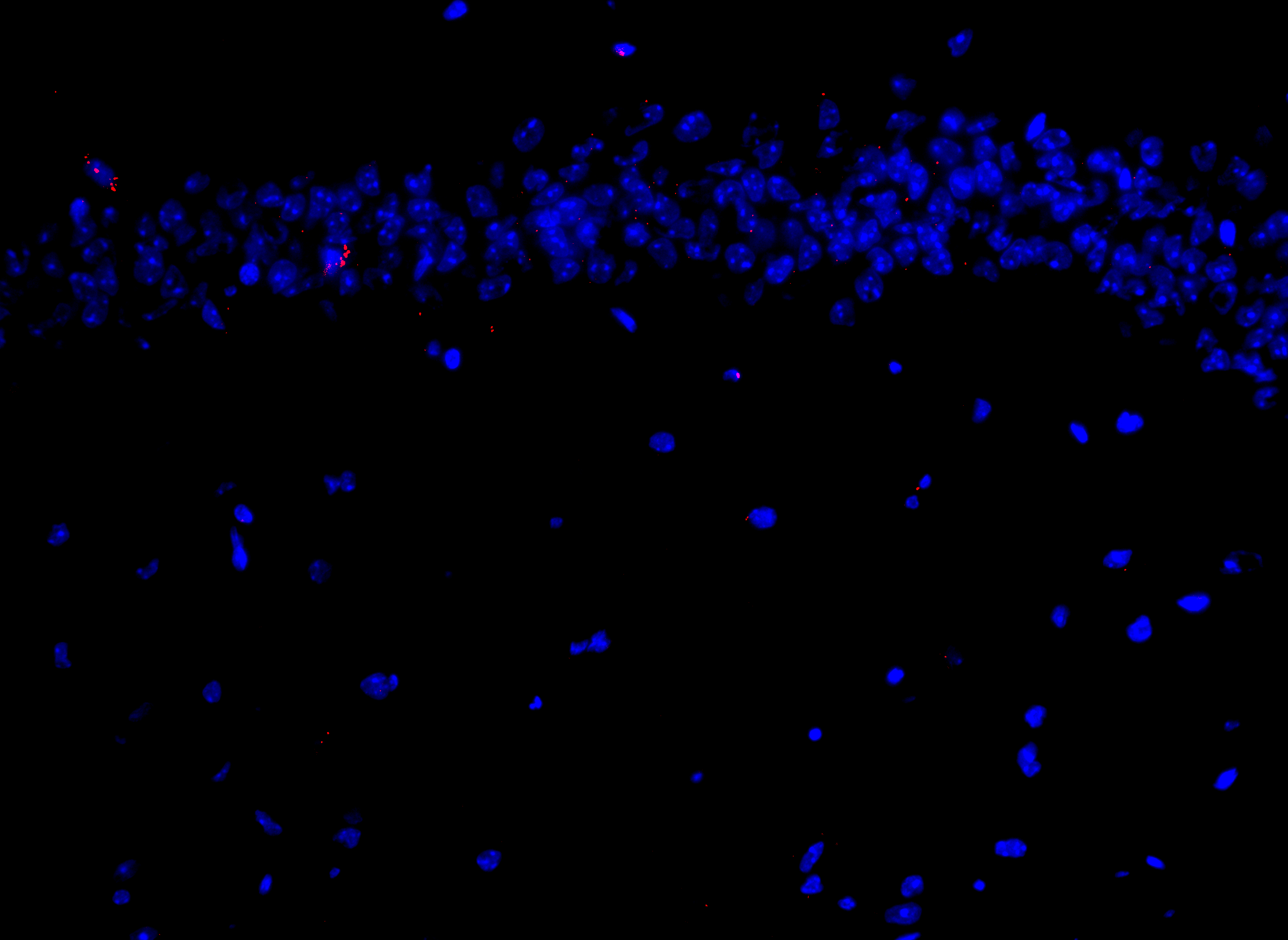

Supplement: Supplementary file 4 — Source data Fig. 2 [file 44321_2026_422_MOESM4_ESM.zip › Figure 2/2A/dbm+AAV-Ctr/Merge.tif]

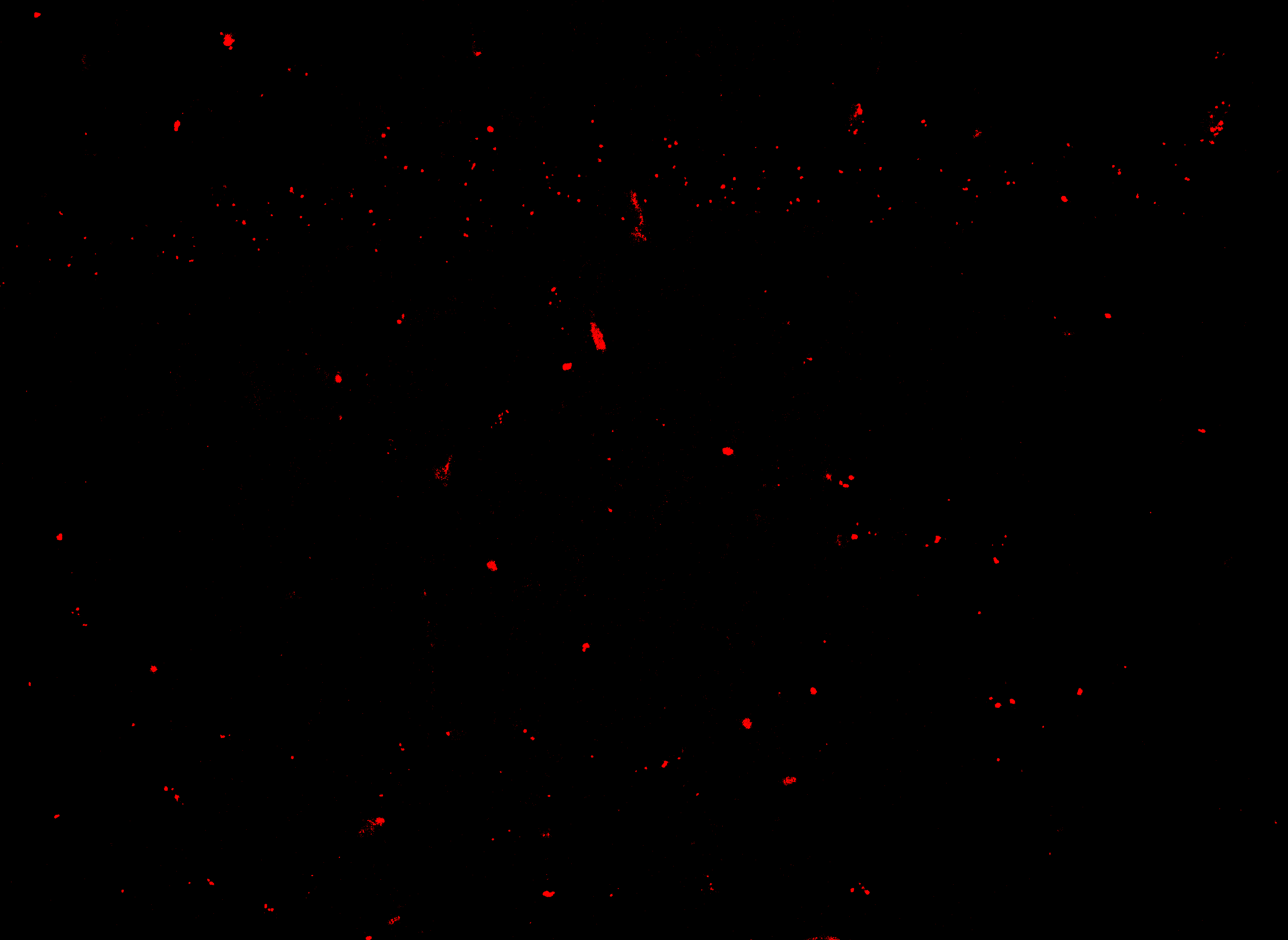

Supplement: Supplementary file 4 — Source data Fig. 2 [file 44321_2026_422_MOESM4_ESM.zip › Figure 2/2A/dbdb+AAV-Flag-Lrpprc-K223R/Tunel.tif]

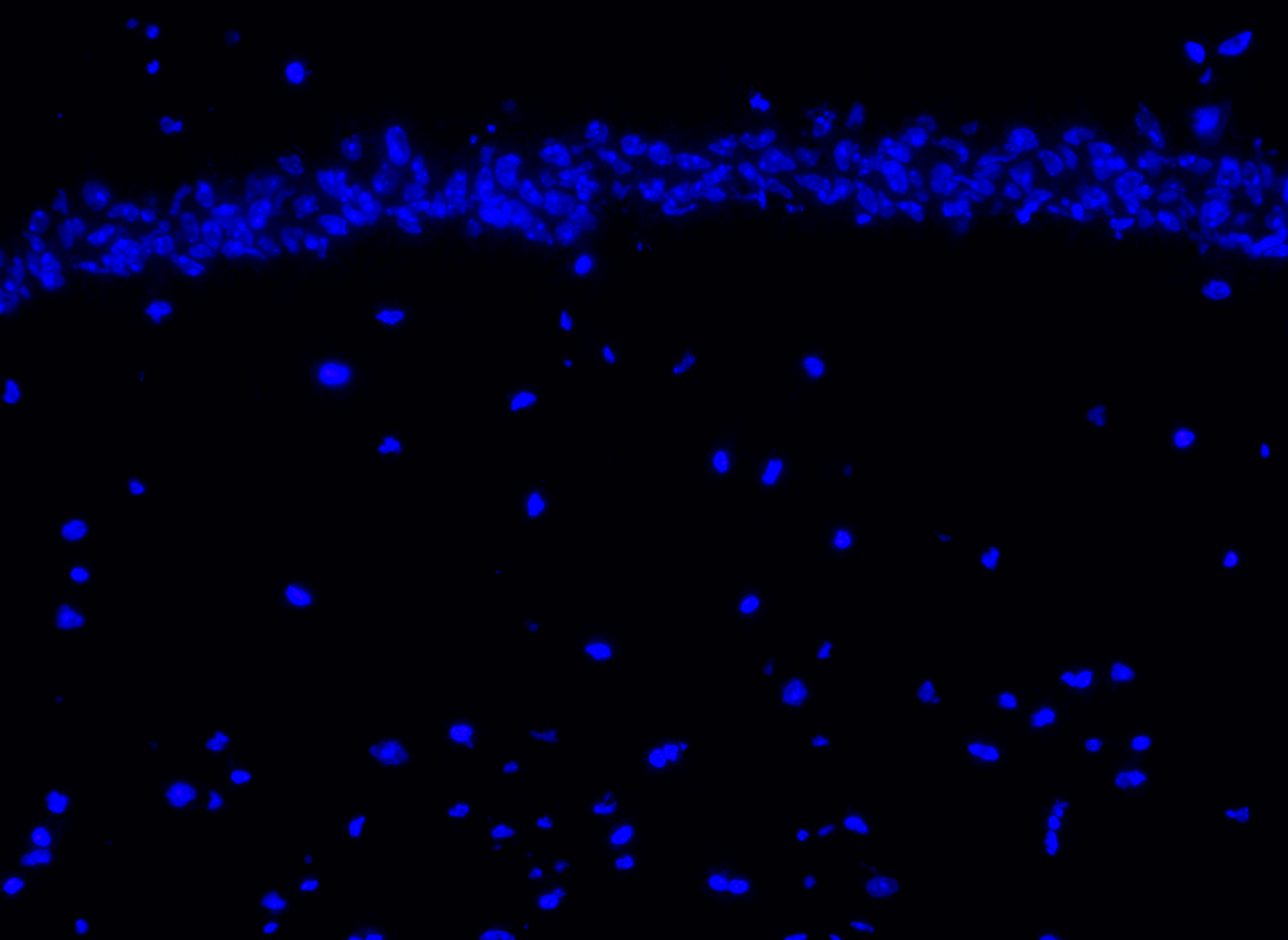

Supplement: Supplementary file 4 — Source data Fig. 2 [file 44321_2026_422_MOESM4_ESM.zip › Figure 2/2A/dbdb+AAV-Flag-Lrpprc-K223R/DAPI.tif]

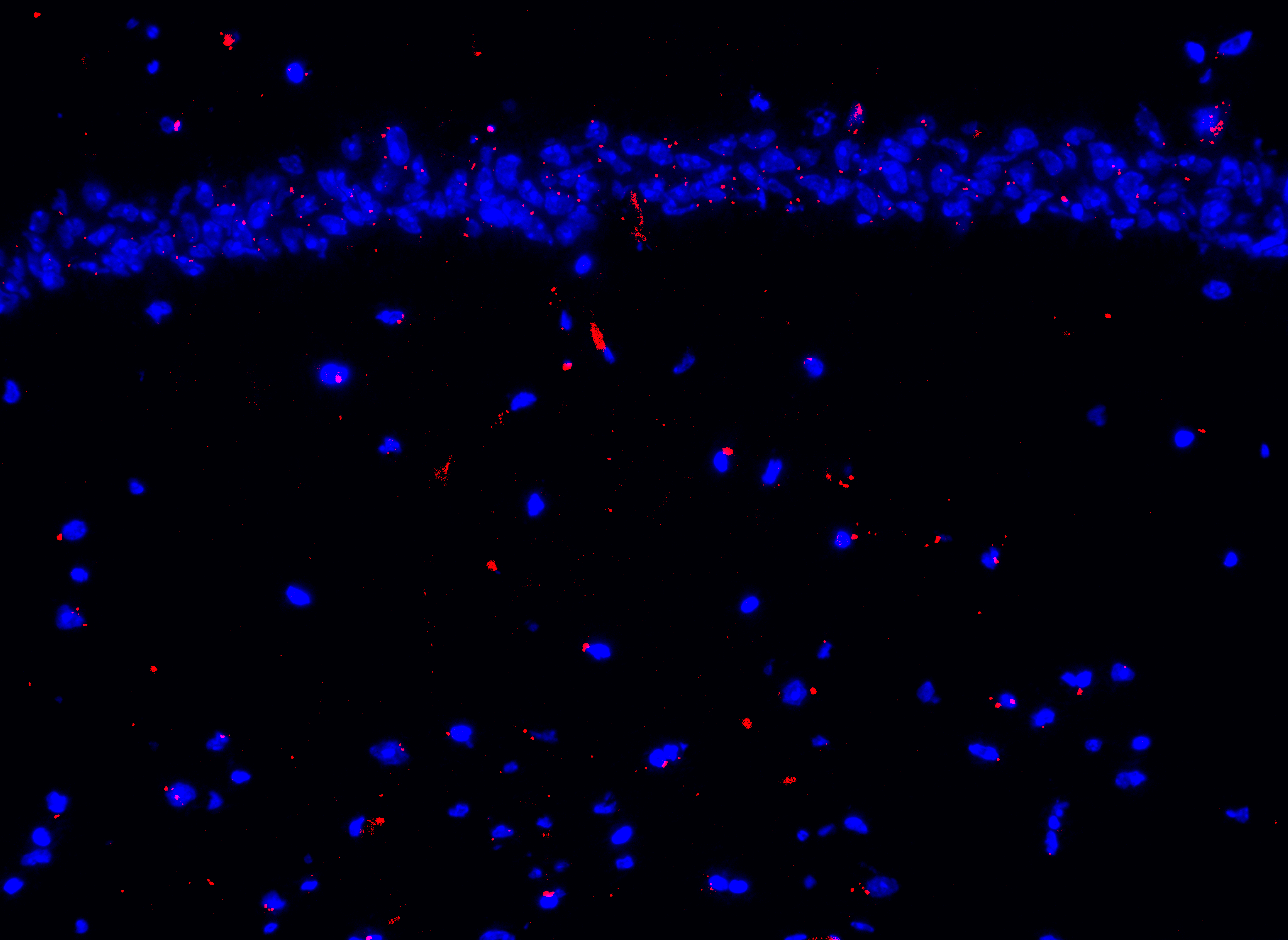

Supplement: Supplementary file 4 — Source data Fig. 2 [file 44321_2026_422_MOESM4_ESM.zip › Figure 2/2A/dbdb+AAV-Flag-Lrpprc-K223R/Merge.tif]

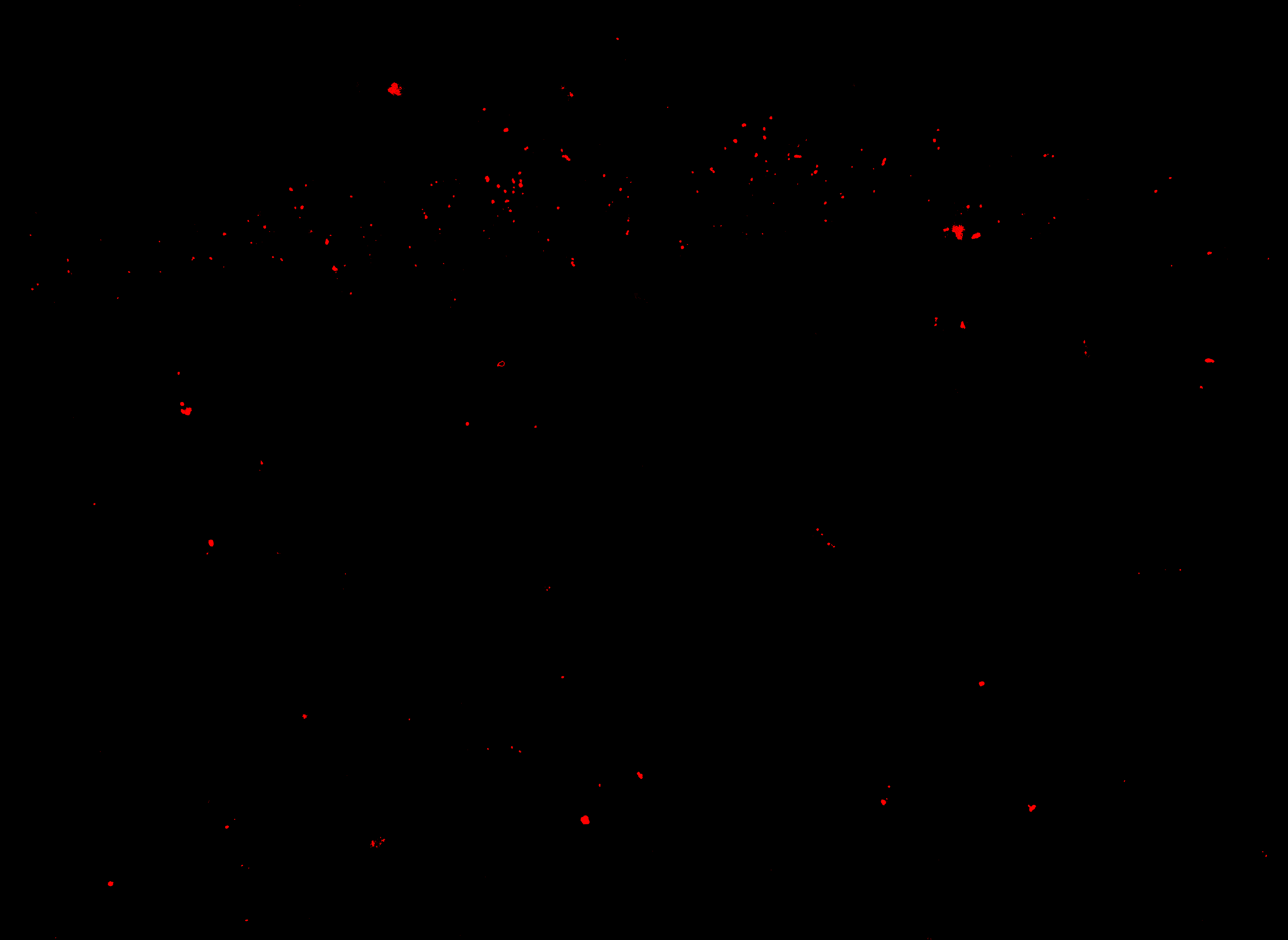

Supplement: Supplementary file 4 — Source data Fig. 2 [file 44321_2026_422_MOESM4_ESM.zip › Figure 2/2C/STZ+AAV-Flag-Lrpprc-K223R/Tunel.tif]

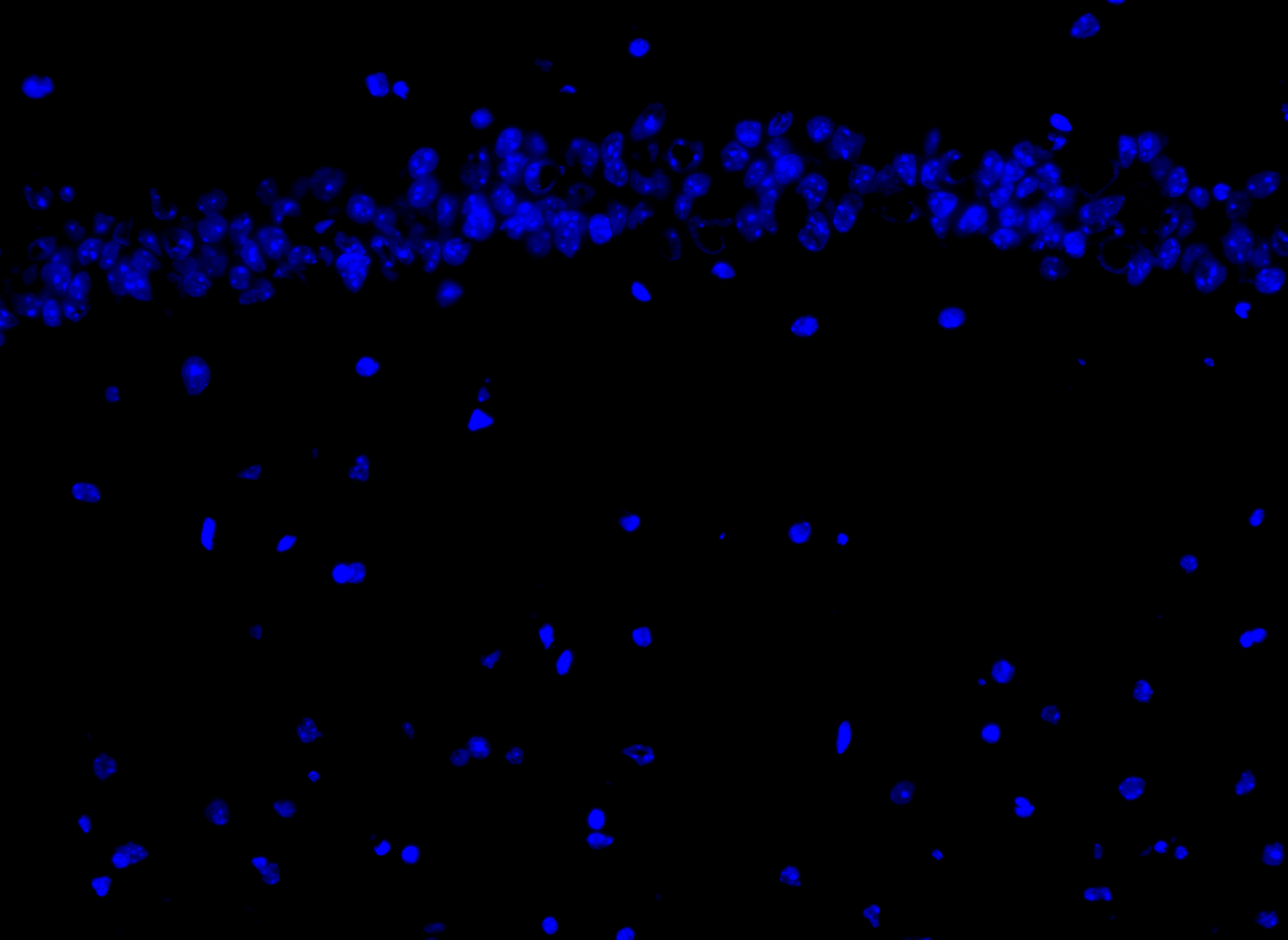

Supplement: Supplementary file 4 — Source data Fig. 2 [file 44321_2026_422_MOESM4_ESM.zip › Figure 2/2C/STZ+AAV-Flag-Lrpprc-K223R/DAPI.tif]

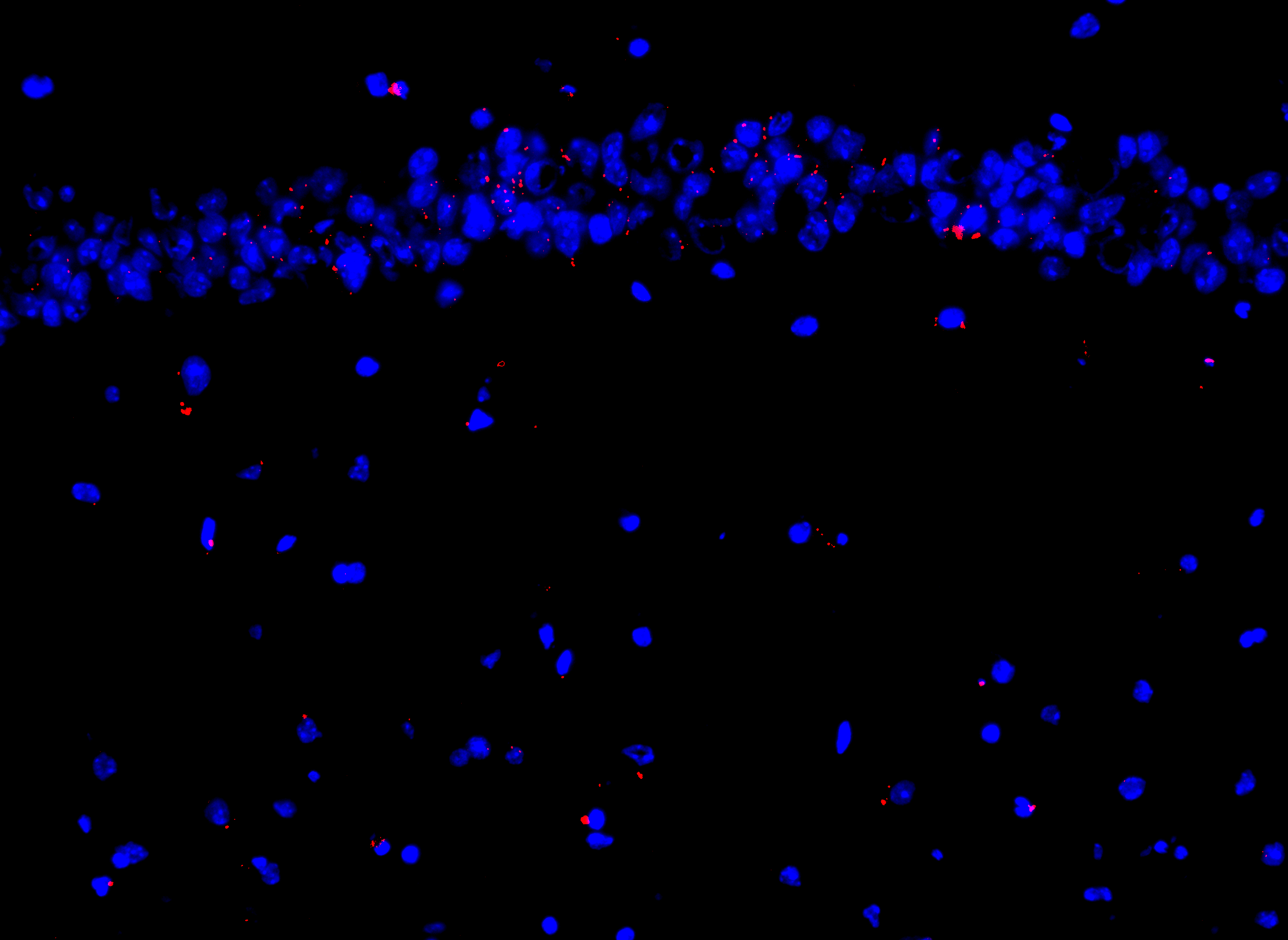

Supplement: Supplementary file 4 — Source data Fig. 2 [file 44321_2026_422_MOESM4_ESM.zip › Figure 2/2C/STZ+AAV-Flag-Lrpprc-K223R/Merge.tif]

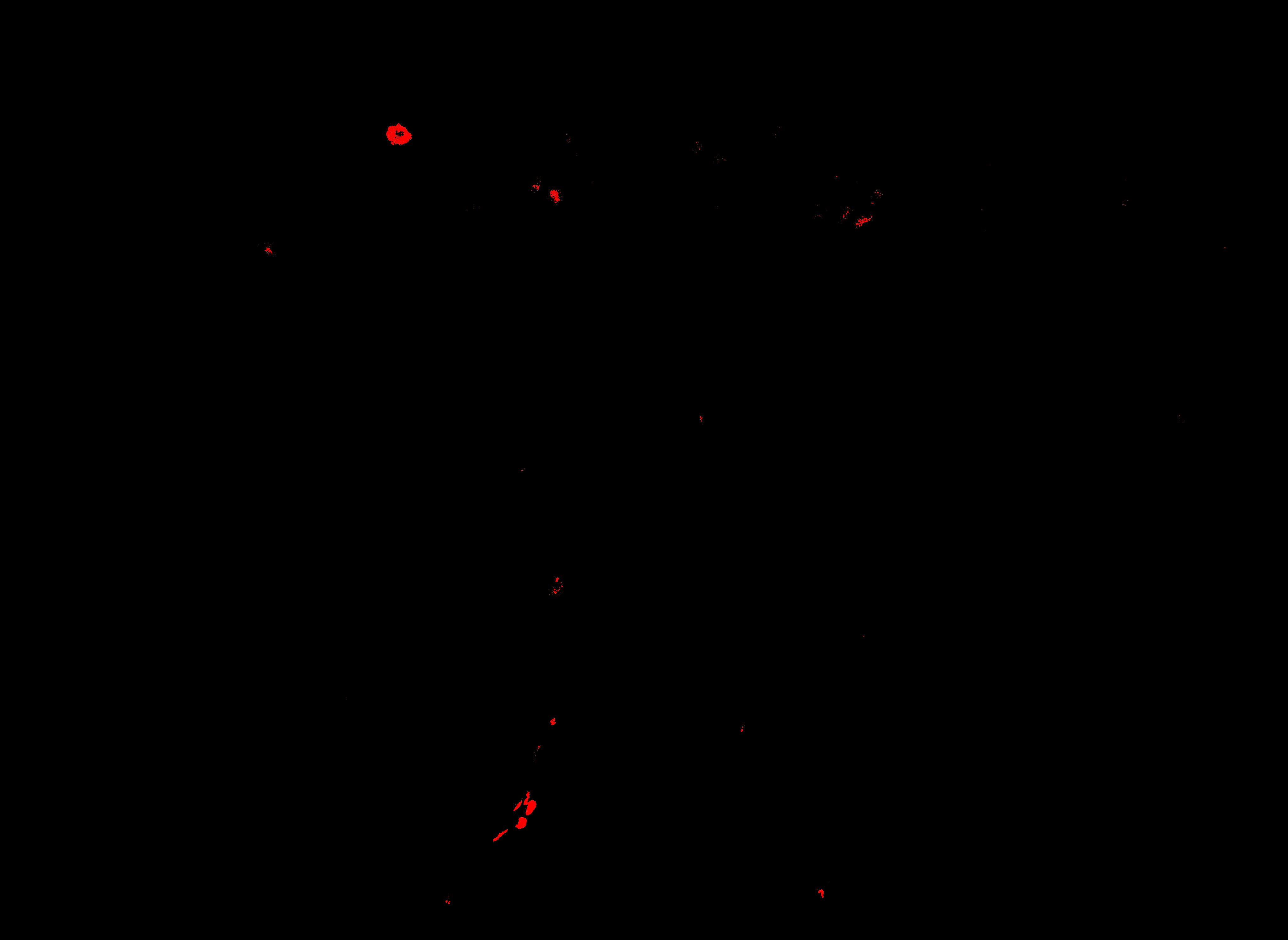

Supplement: Supplementary file 4 — Source data Fig. 2 [file 44321_2026_422_MOESM4_ESM.zip › Figure 2/2C/Ctr+AAV-Ctr/Tunel.tif]

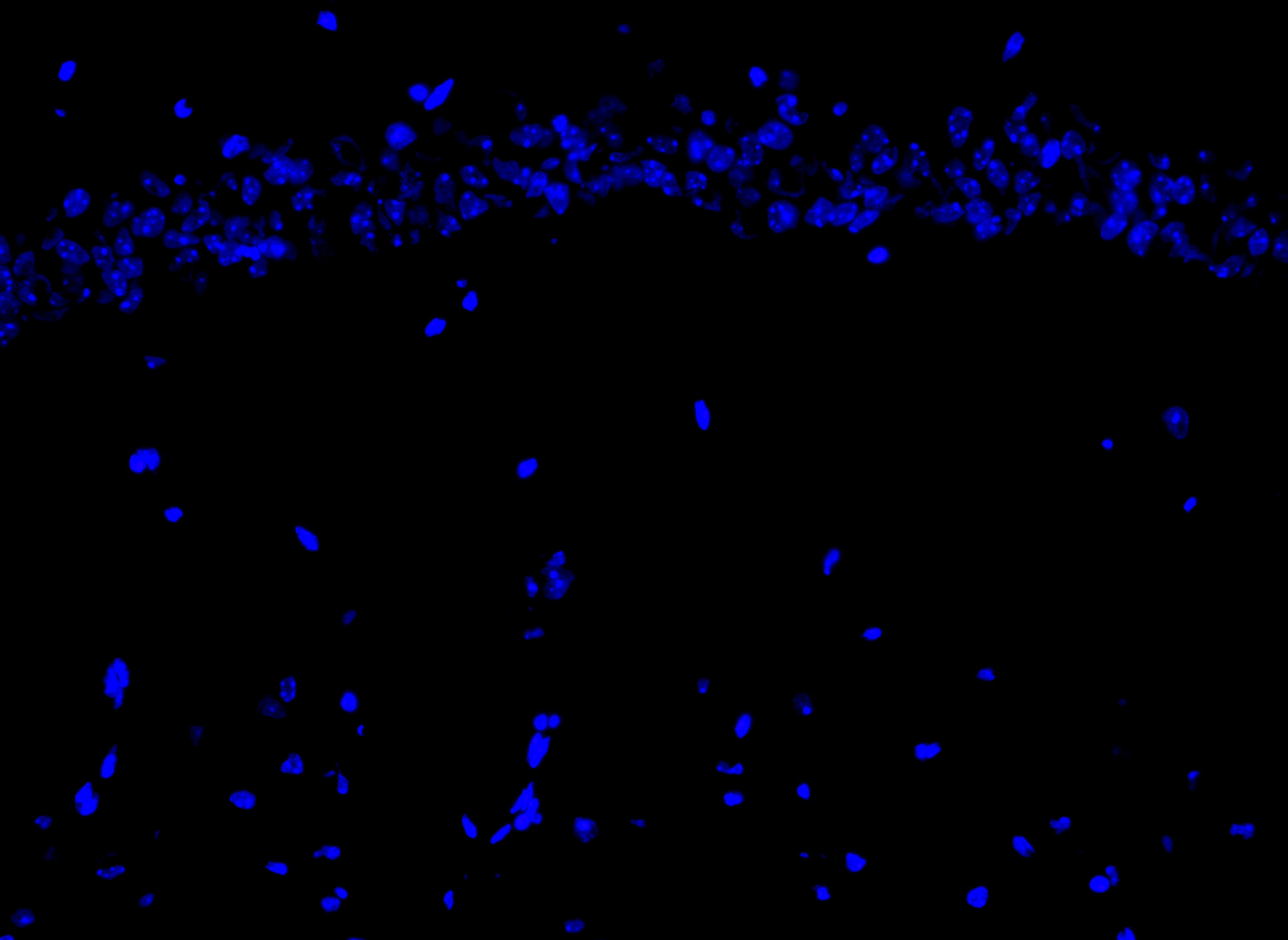

Supplement: Supplementary file 4 — Source data Fig. 2 [file 44321_2026_422_MOESM4_ESM.zip › Figure 2/2C/Ctr+AAV-Ctr/DAPI.tif]

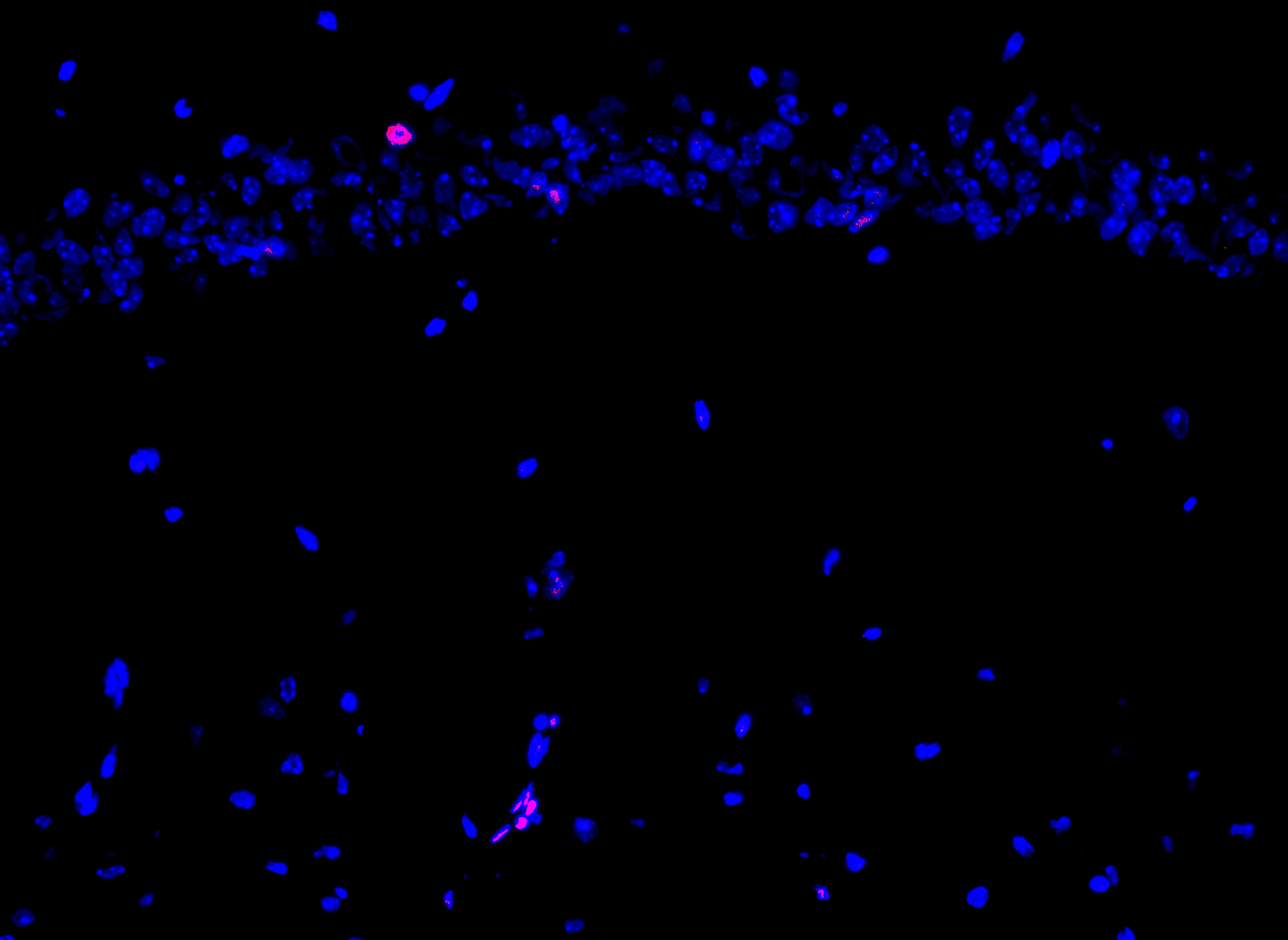

Supplement: Supplementary file 4 — Source data Fig. 2 [file 44321_2026_422_MOESM4_ESM.zip › Figure 2/2C/Ctr+AAV-Ctr/Merge.tif]

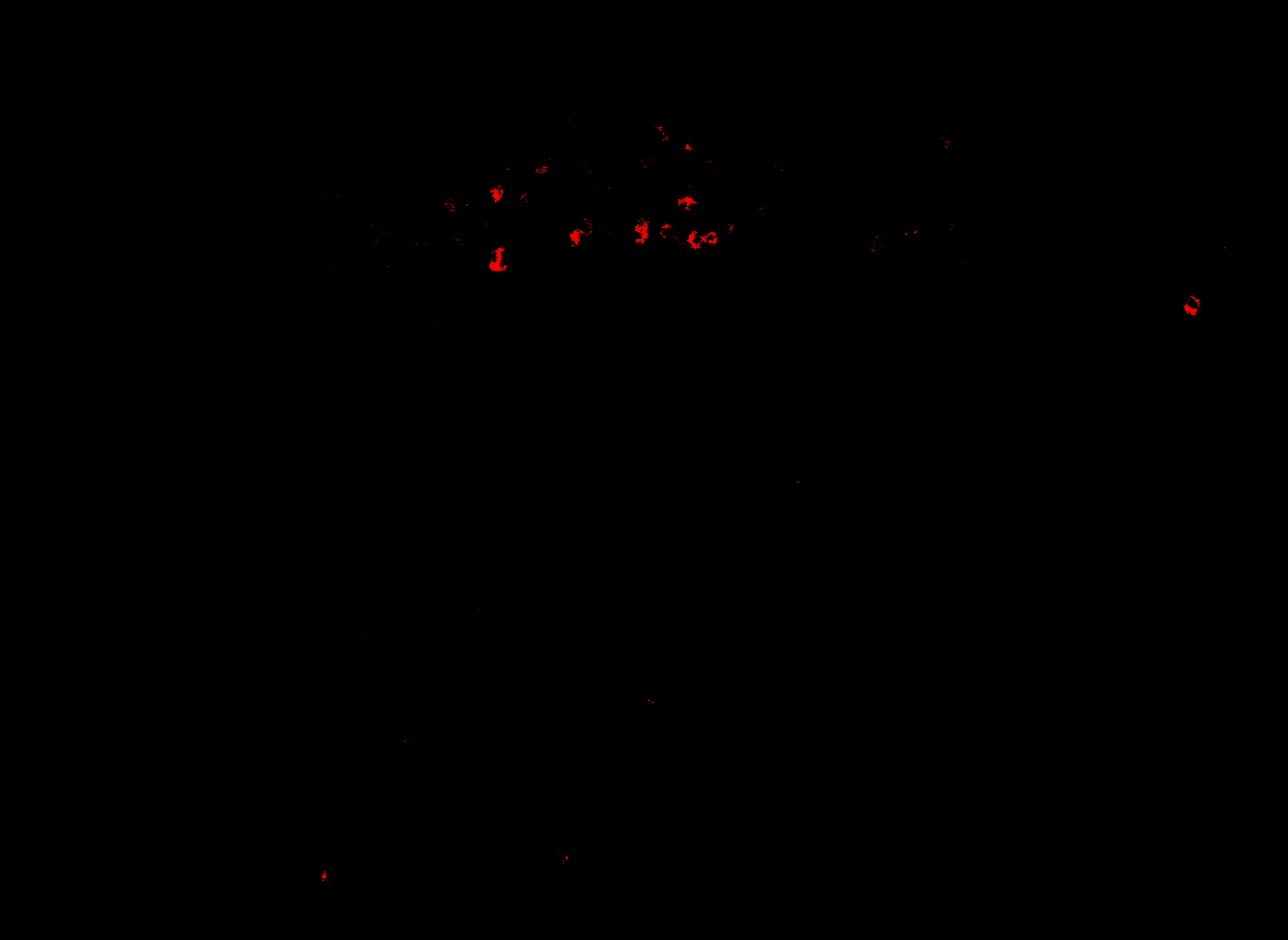

Supplement: Supplementary file 4 — Source data Fig. 2 [file 44321_2026_422_MOESM4_ESM.zip › Figure 2/2C/Ctr+AAV-Flag-Lrpprc-WT/Tunel.tif]

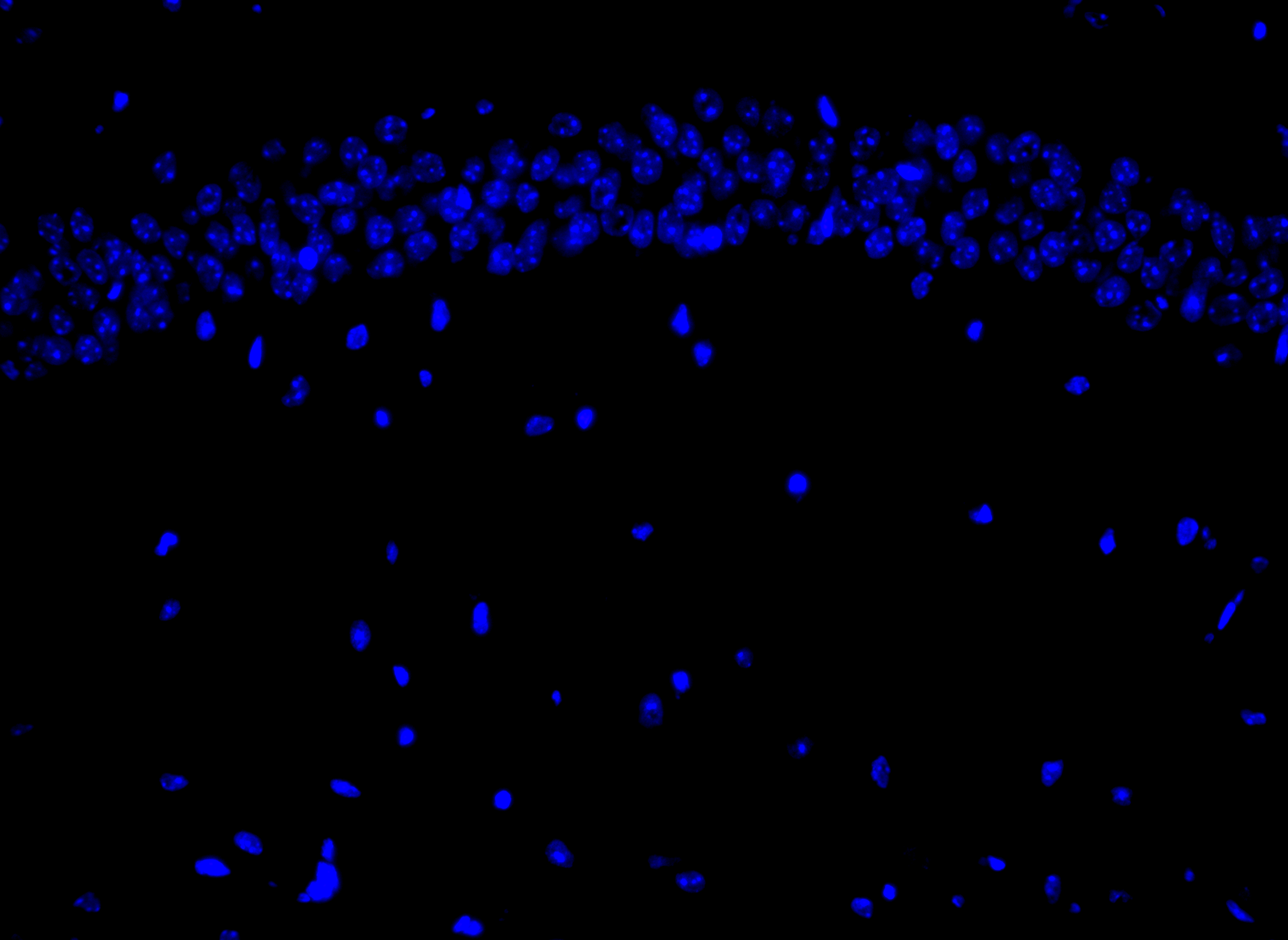

Supplement: Supplementary file 4 — Source data Fig. 2 [file 44321_2026_422_MOESM4_ESM.zip › Figure 2/2C/Ctr+AAV-Flag-Lrpprc-WT/DAPI.tif]

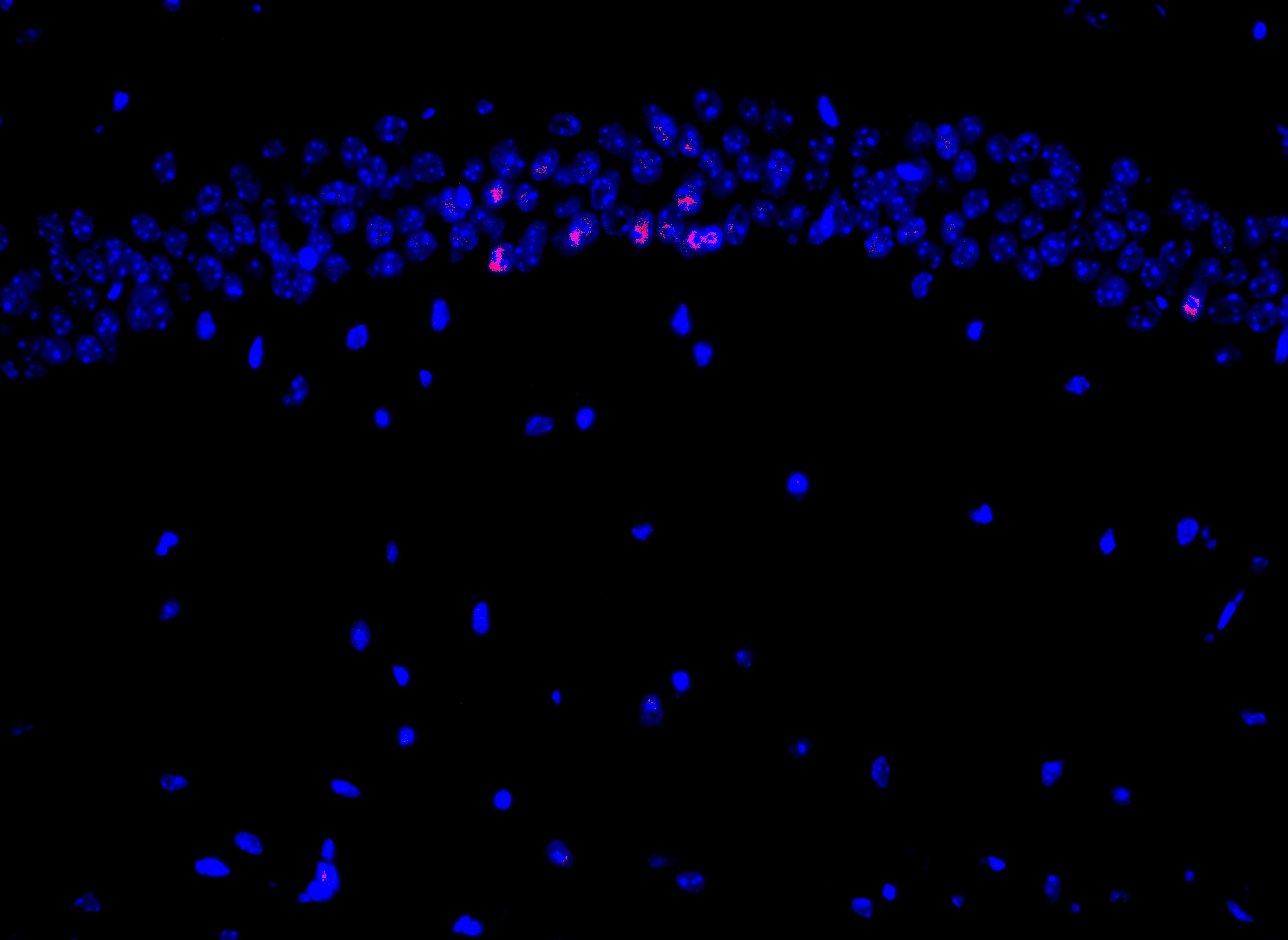

Supplement: Supplementary file 4 — Source data Fig. 2 [file 44321_2026_422_MOESM4_ESM.zip › Figure 2/2C/Ctr+AAV-Flag-Lrpprc-WT/Merge.tif]

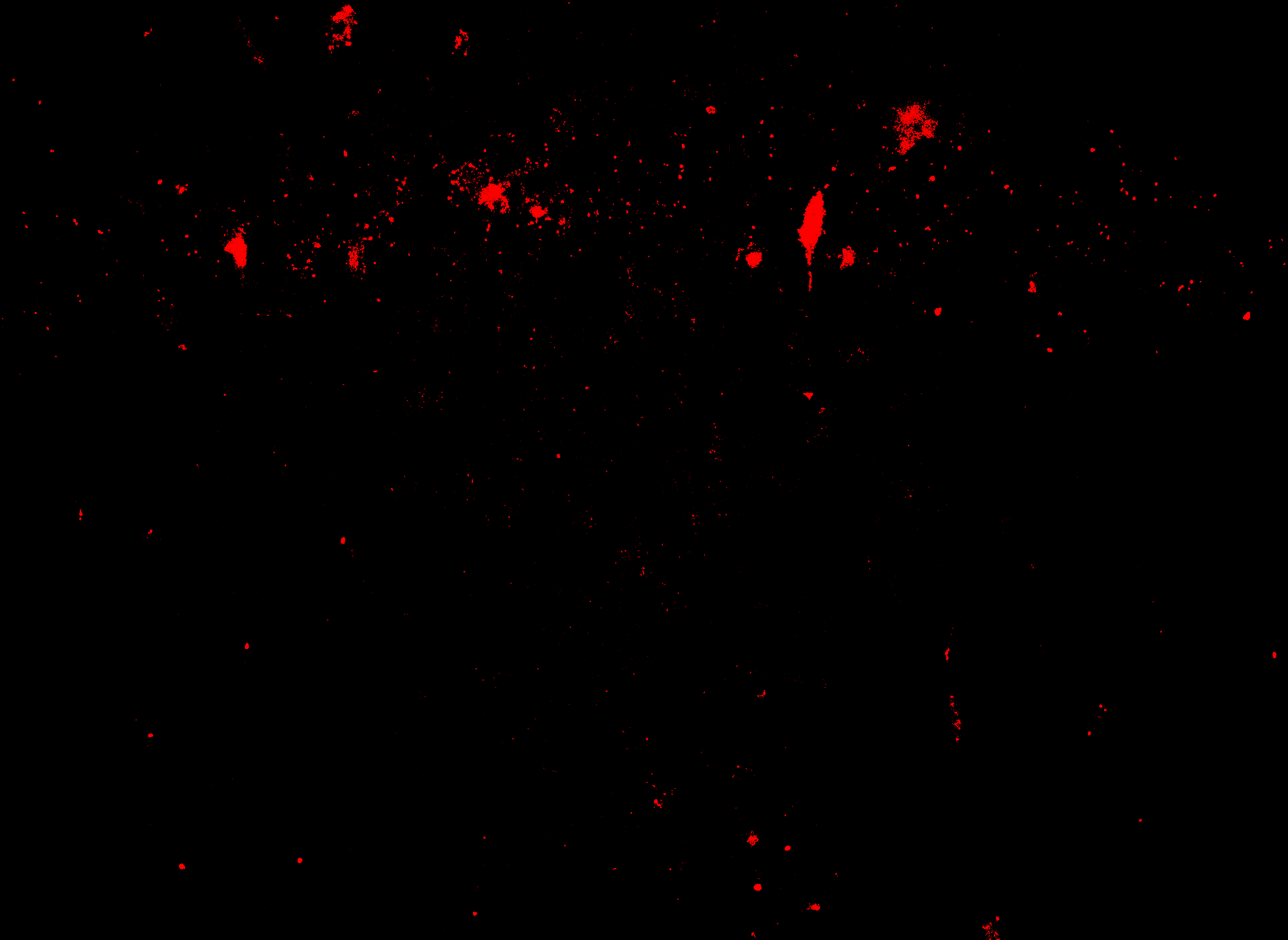

Supplement: Supplementary file 4 — Source data Fig. 2 [file 44321_2026_422_MOESM4_ESM.zip › Figure 2/2C/STZ+AAV-Flag-Lrpprc-WT/Tunel.tif]

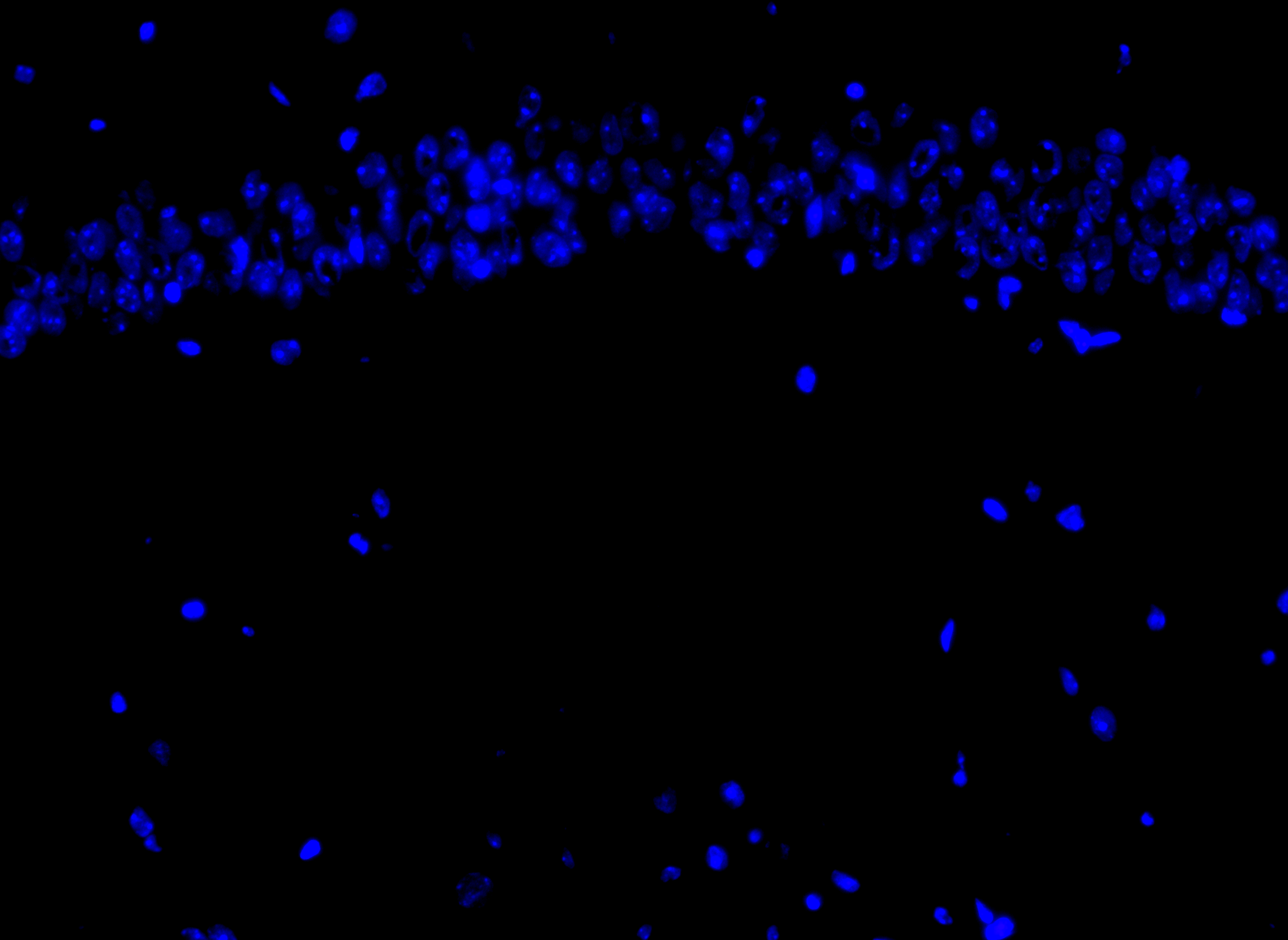

Supplement: Supplementary file 4 — Source data Fig. 2 [file 44321_2026_422_MOESM4_ESM.zip › Figure 2/2C/STZ+AAV-Flag-Lrpprc-WT/DAPI.tif]

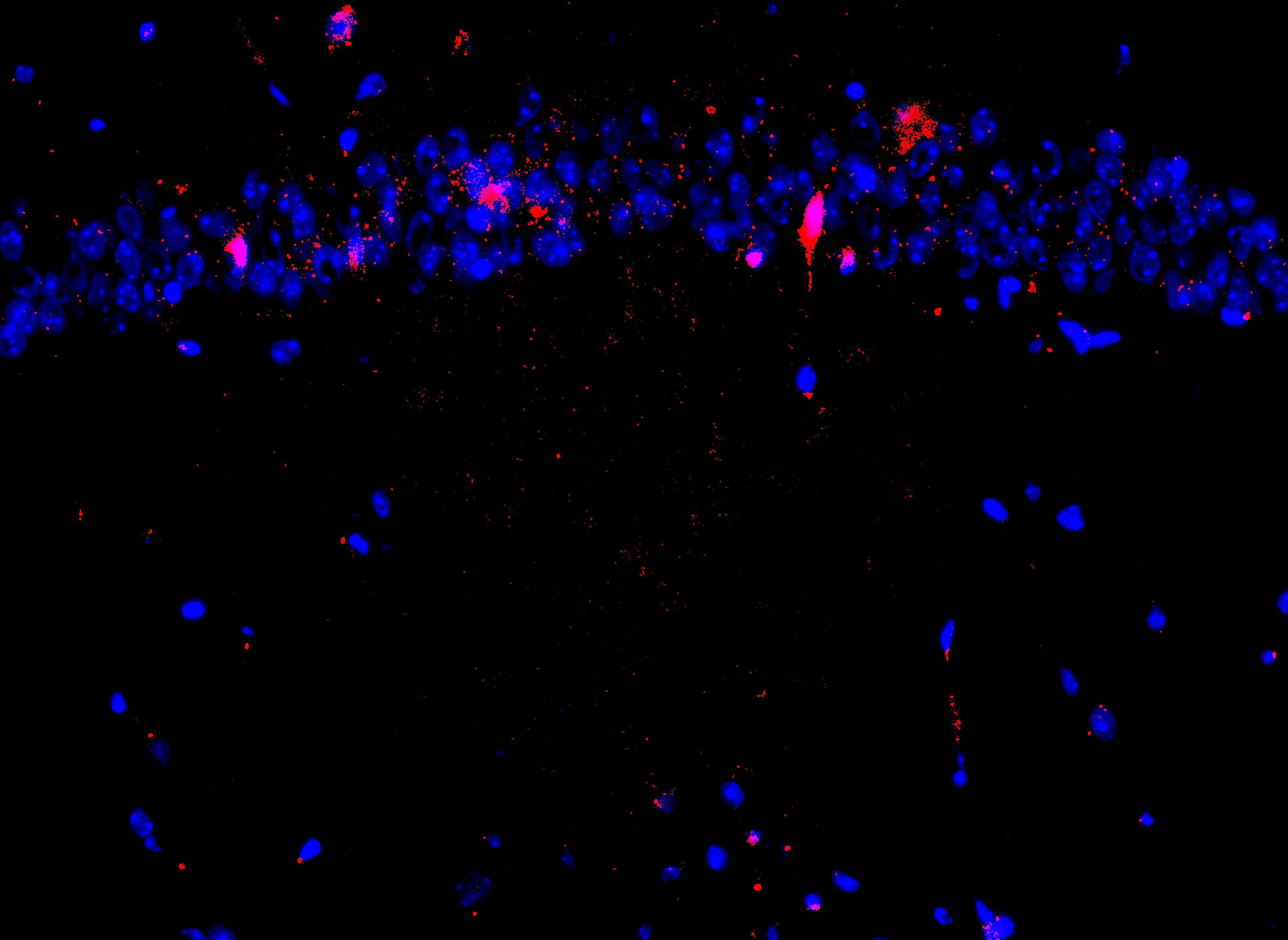

Supplement: Supplementary file 4 — Source data Fig. 2 [file 44321_2026_422_MOESM4_ESM.zip › Figure 2/2C/STZ+AAV-Flag-Lrpprc-WT/Merge.tif]

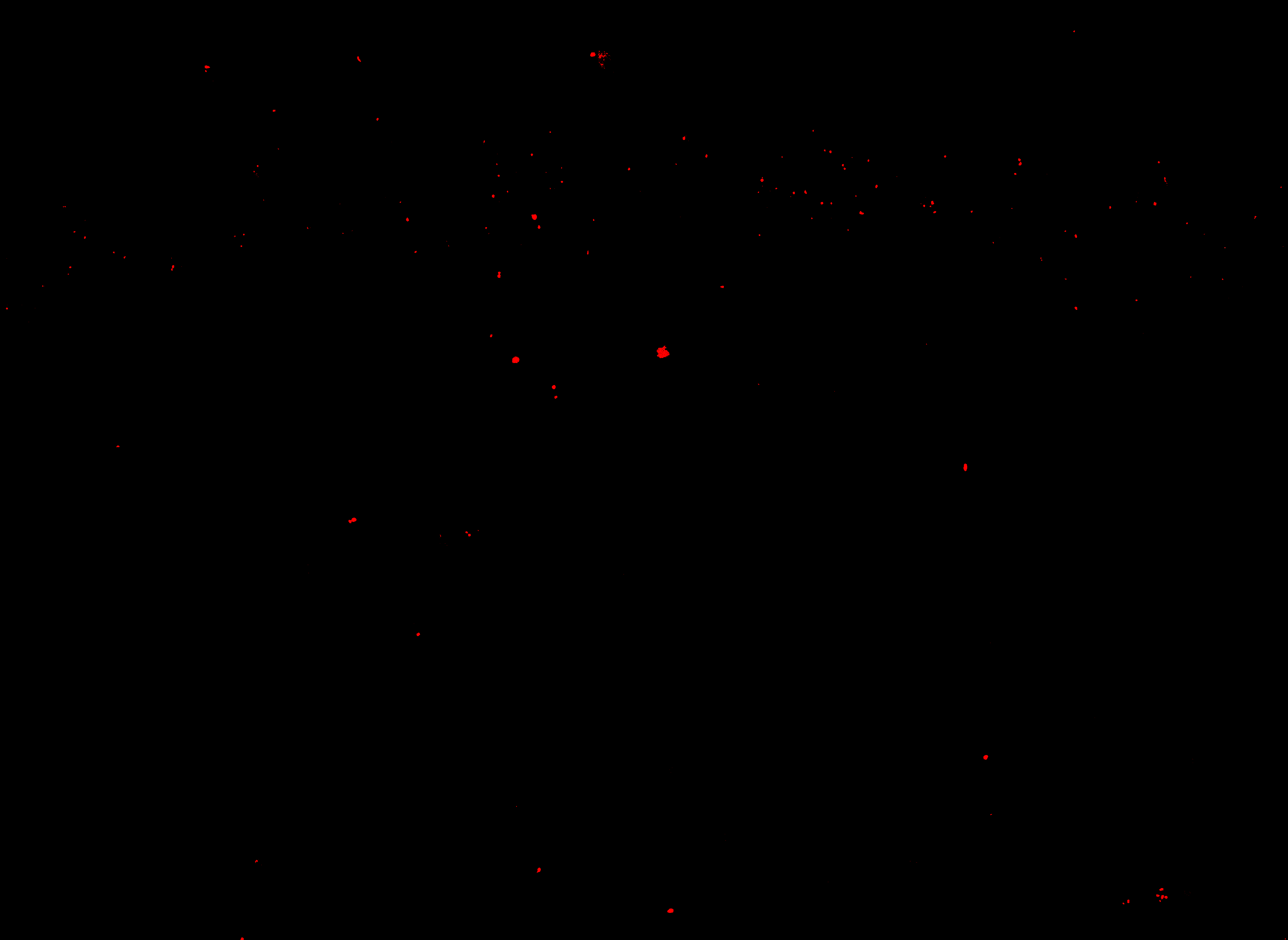

Supplement: Supplementary file 4 — Source data Fig. 2 [file 44321_2026_422_MOESM4_ESM.zip › Figure 2/2C/Ctr+AAV-Flag-Lrpprc-K223R/Tunel.tif]

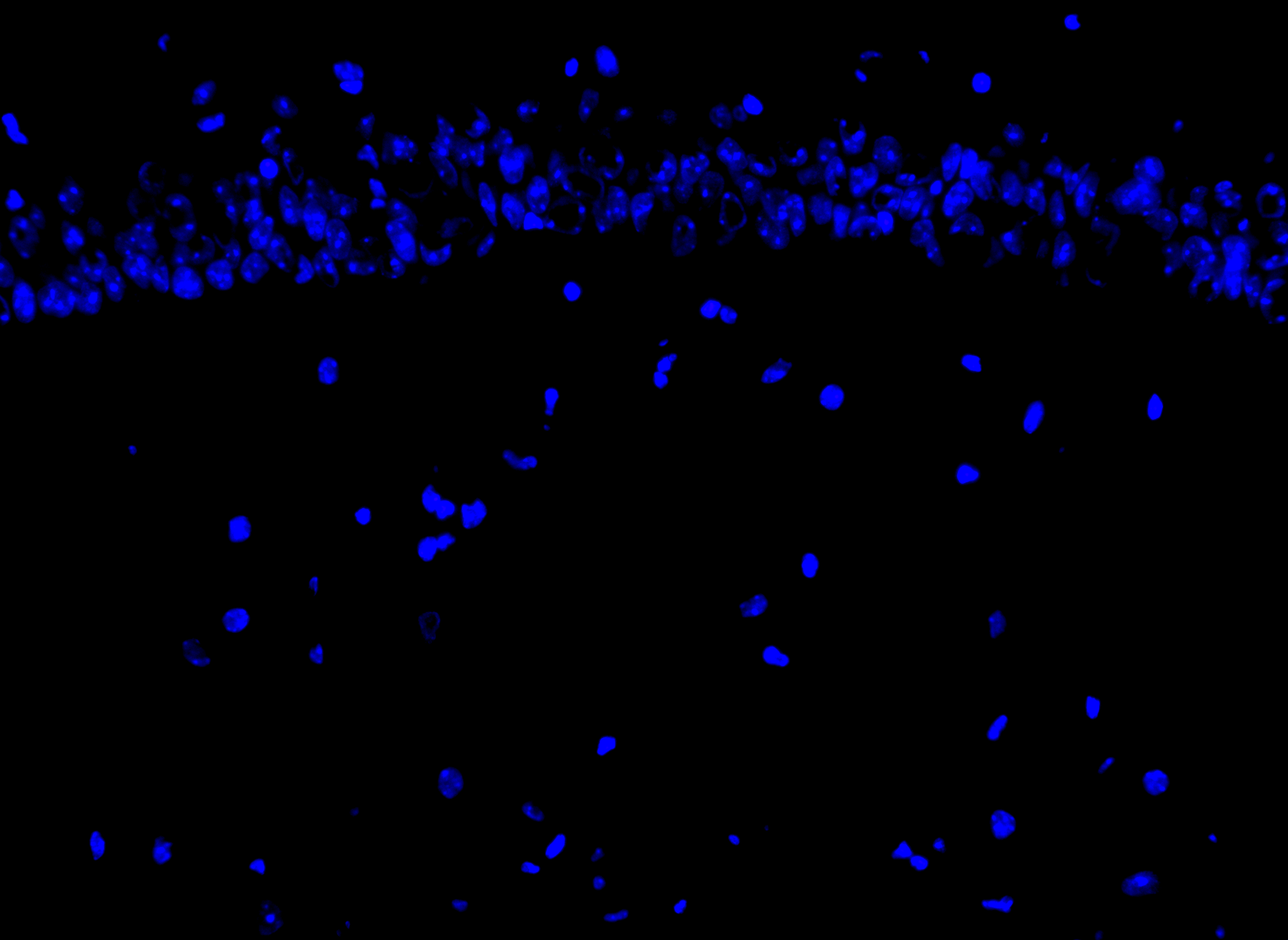

Supplement: Supplementary file 4 — Source data Fig. 2 [file 44321_2026_422_MOESM4_ESM.zip › Figure 2/2C/Ctr+AAV-Flag-Lrpprc-K223R/DAPI.tif]

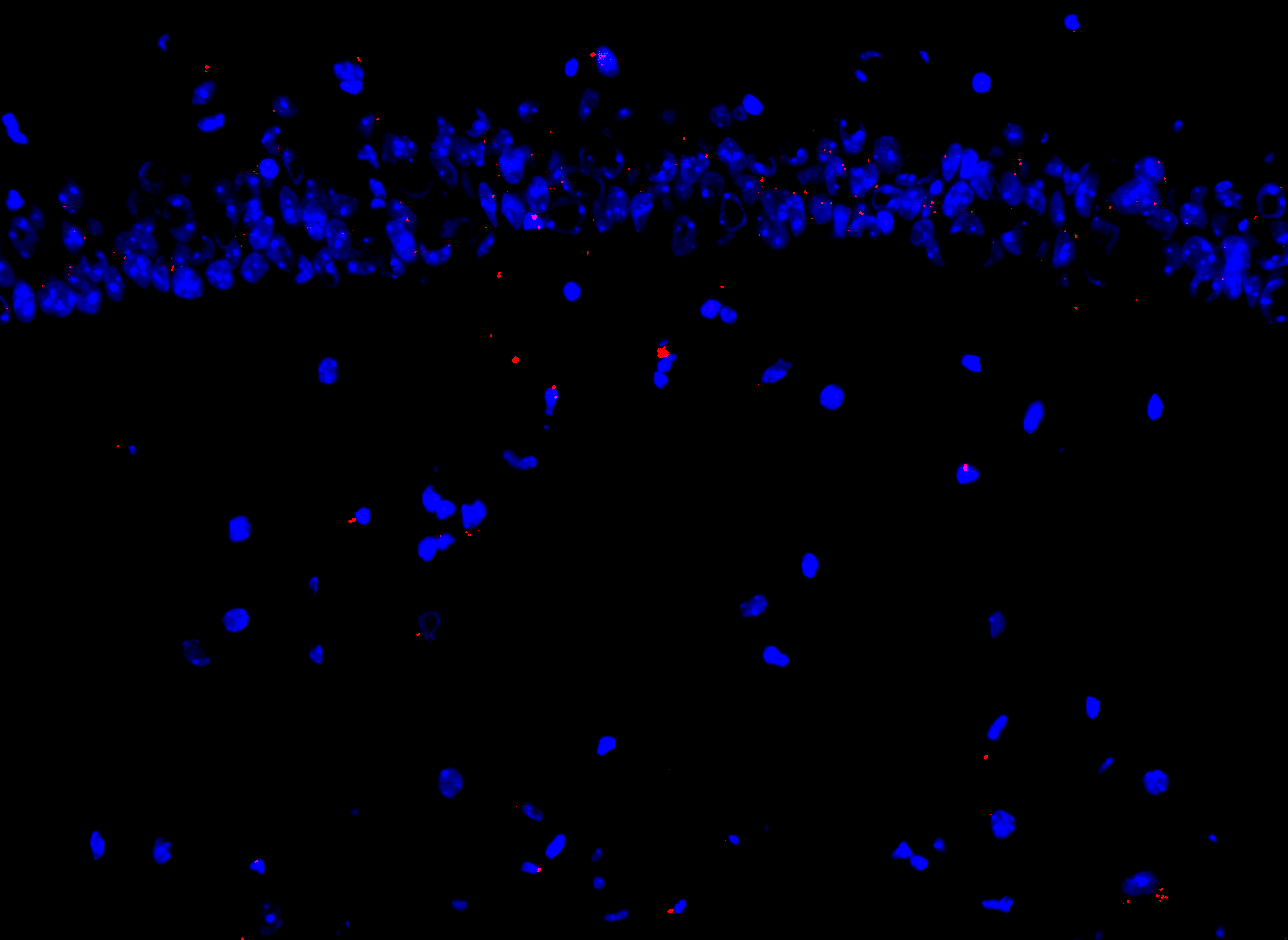

Supplement: Supplementary file 4 — Source data Fig. 2 [file 44321_2026_422_MOESM4_ESM.zip › Figure 2/2C/Ctr+AAV-Flag-Lrpprc-K223R/Merge.tif]

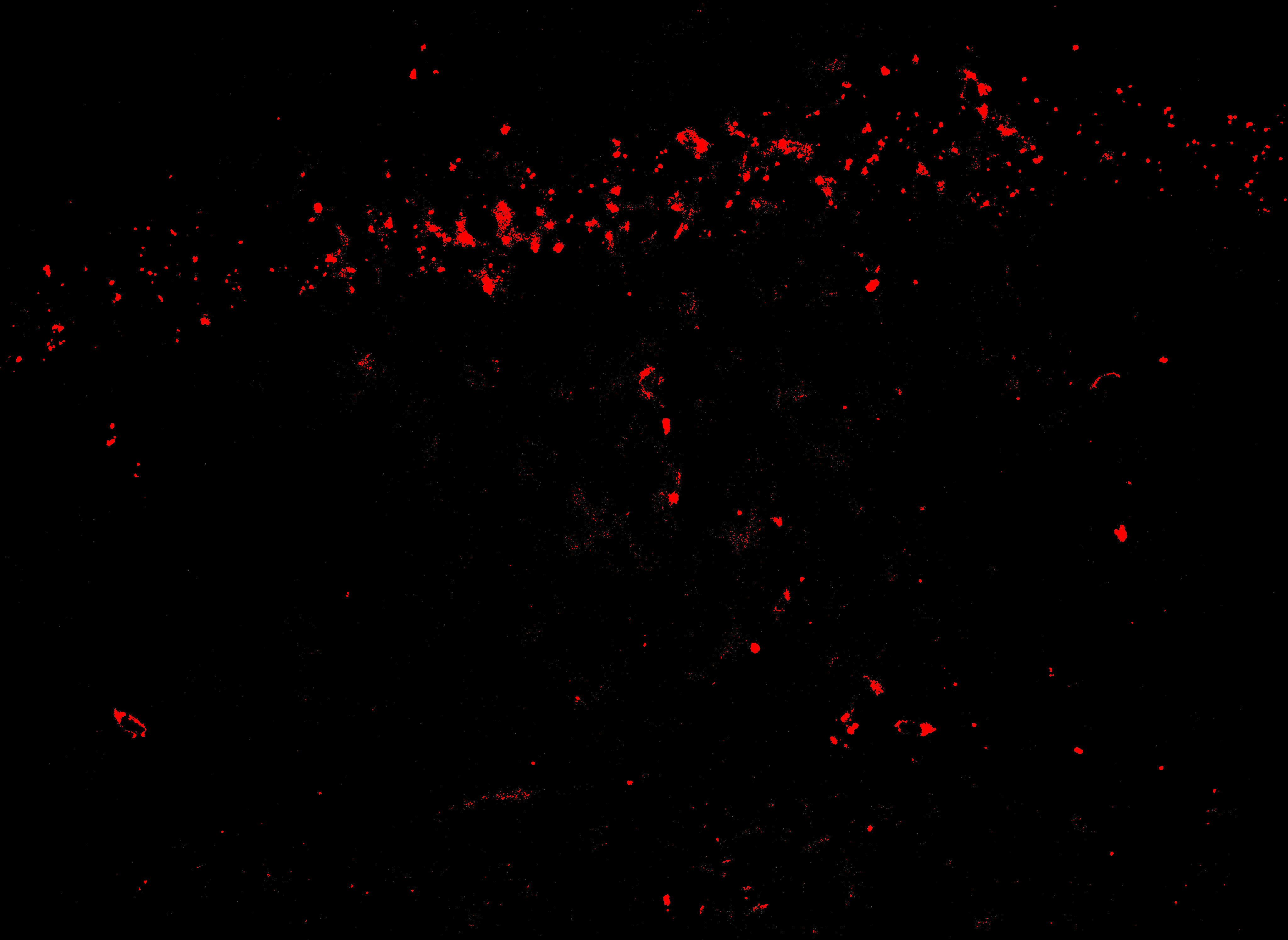

Supplement: Supplementary file 4 — Source data Fig. 2 [file 44321_2026_422_MOESM4_ESM.zip › Figure 2/2C/STZ+AAV-Ctr/Tunel.tif]

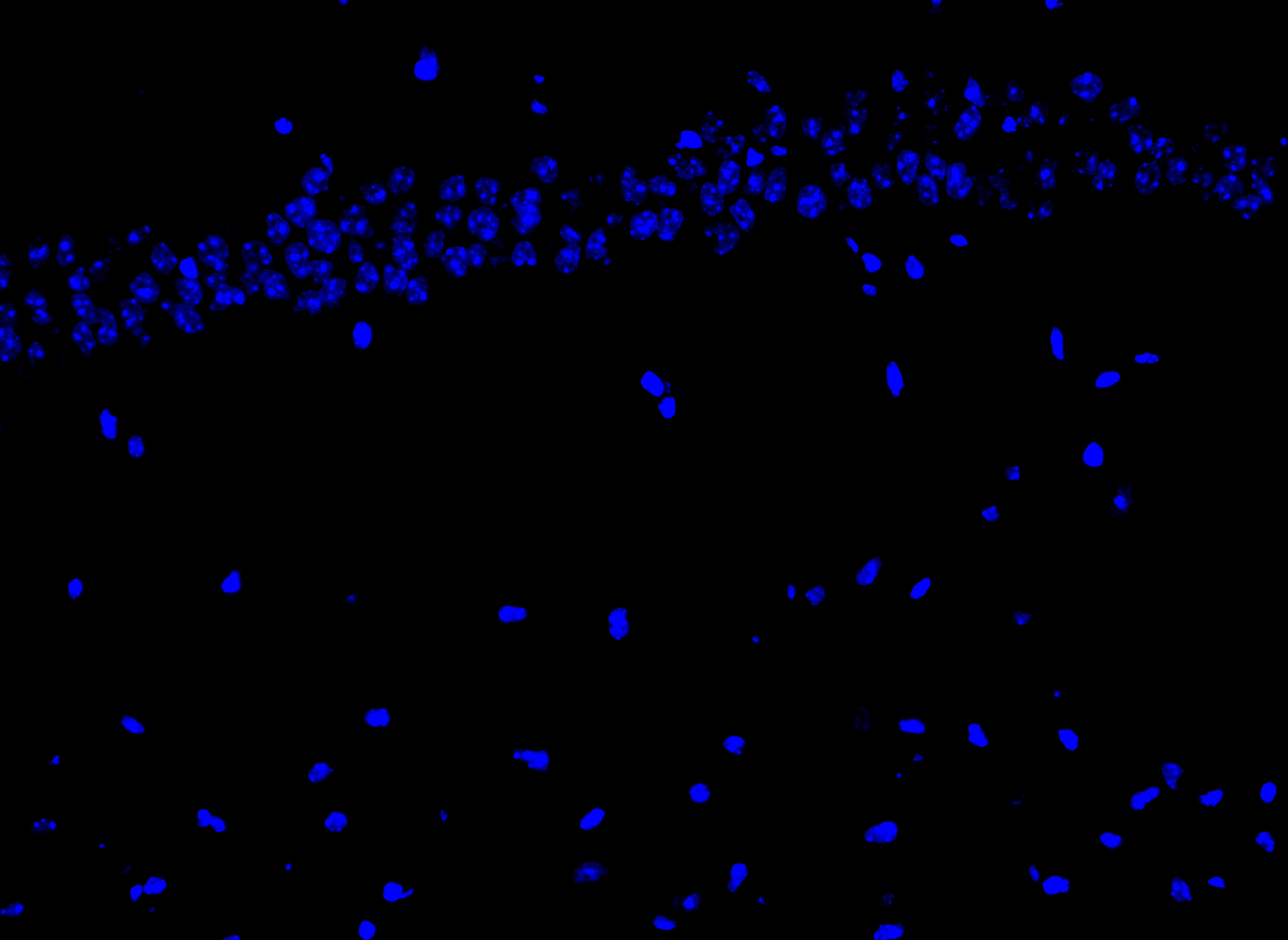

Supplement: Supplementary file 4 — Source data Fig. 2 [file 44321_2026_422_MOESM4_ESM.zip › Figure 2/2C/STZ+AAV-Ctr/DAPI.tif]

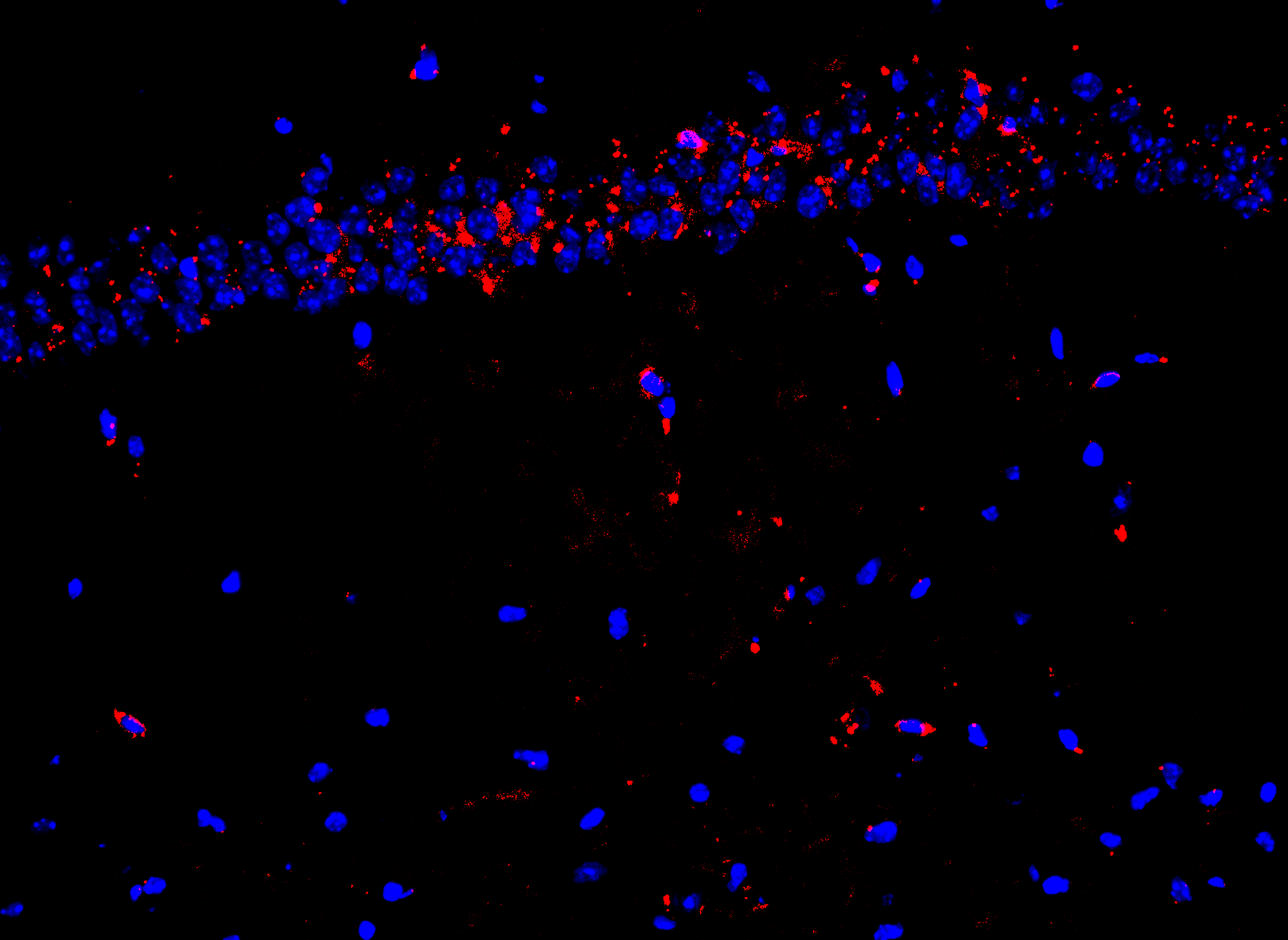

Supplement: Supplementary file 4 — Source data Fig. 2 [file 44321_2026_422_MOESM4_ESM.zip › Figure 2/2C/STZ+AAV-Ctr/Merge.tif]

## IP

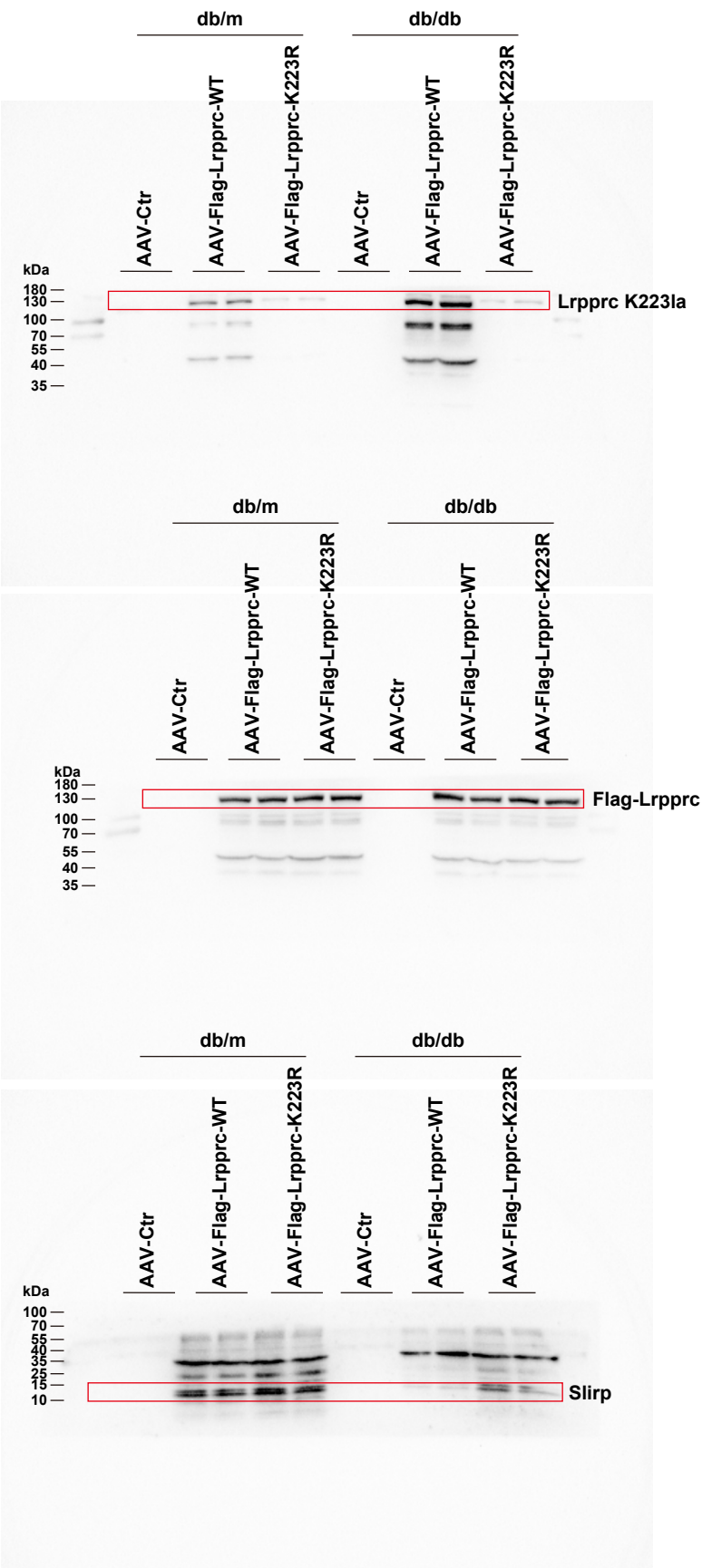

## Input

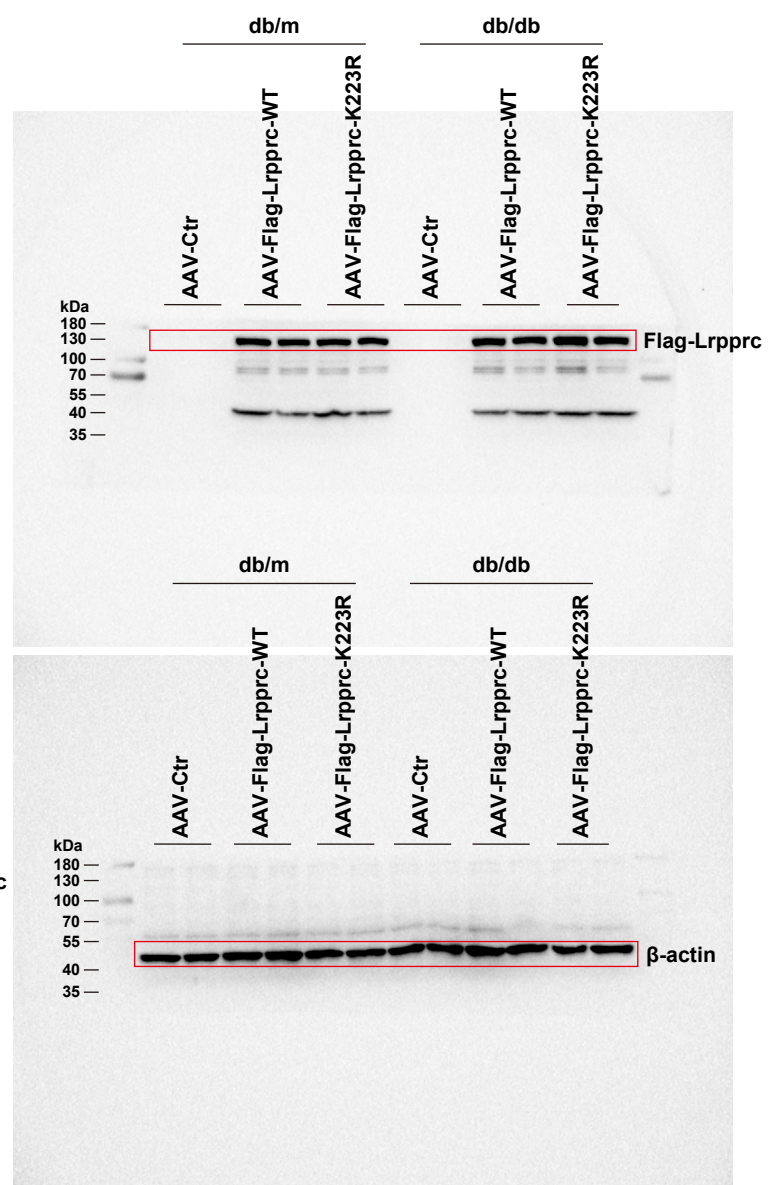

Supplement: Supplementary file 5 — Source data Fig. 3 [file 44321_2026_422_MOESM5_ESM.zip › Figure 3/Figure 3B/Figure 3B.pdf]

# IP

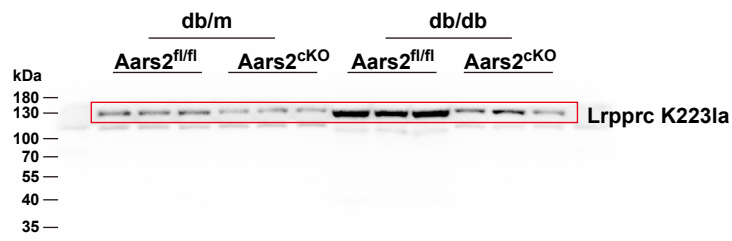

# Input

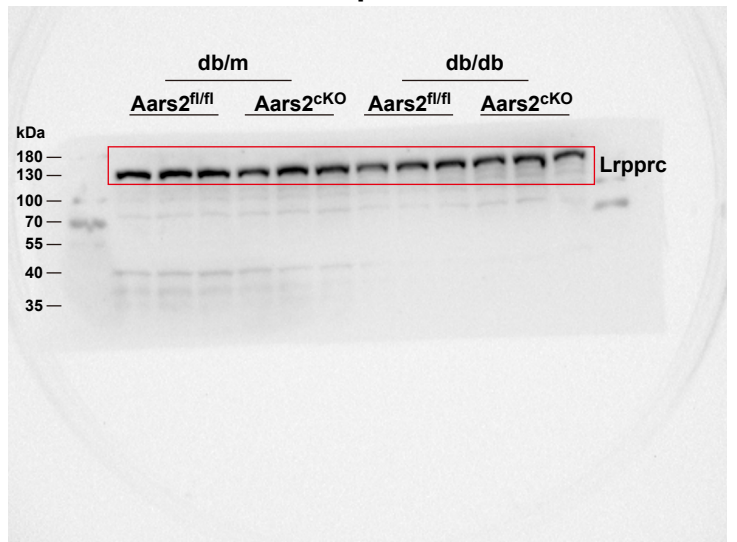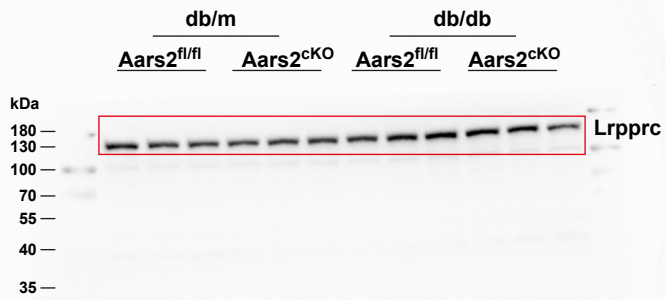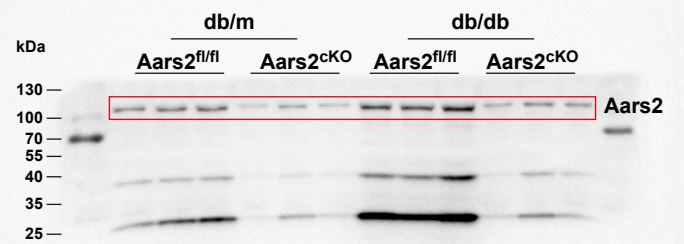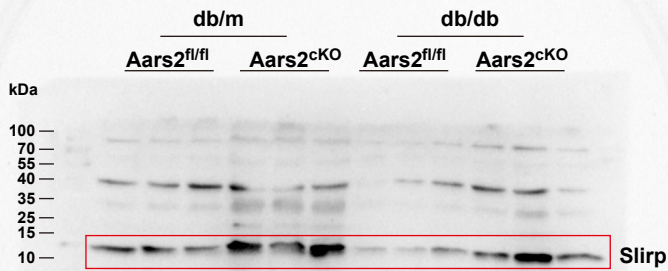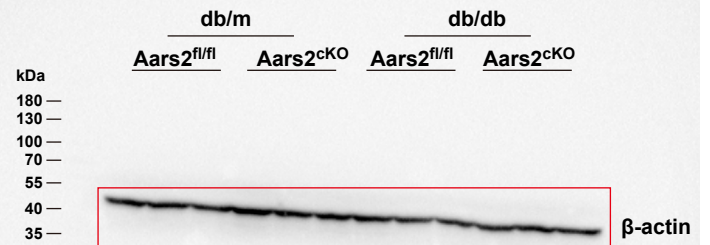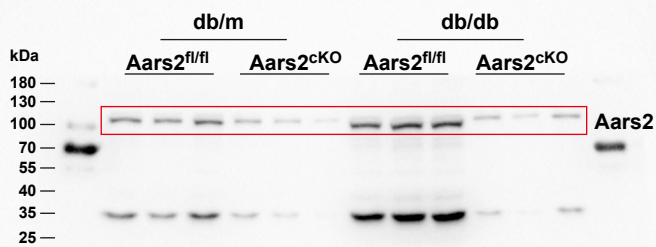

Supplement: Supplementary file 6 — Source data Fig. 4 [file 44321_2026_422_MOESM6_ESM.zip › Figure 4/Figure 4A/Figure 4A.pdf]

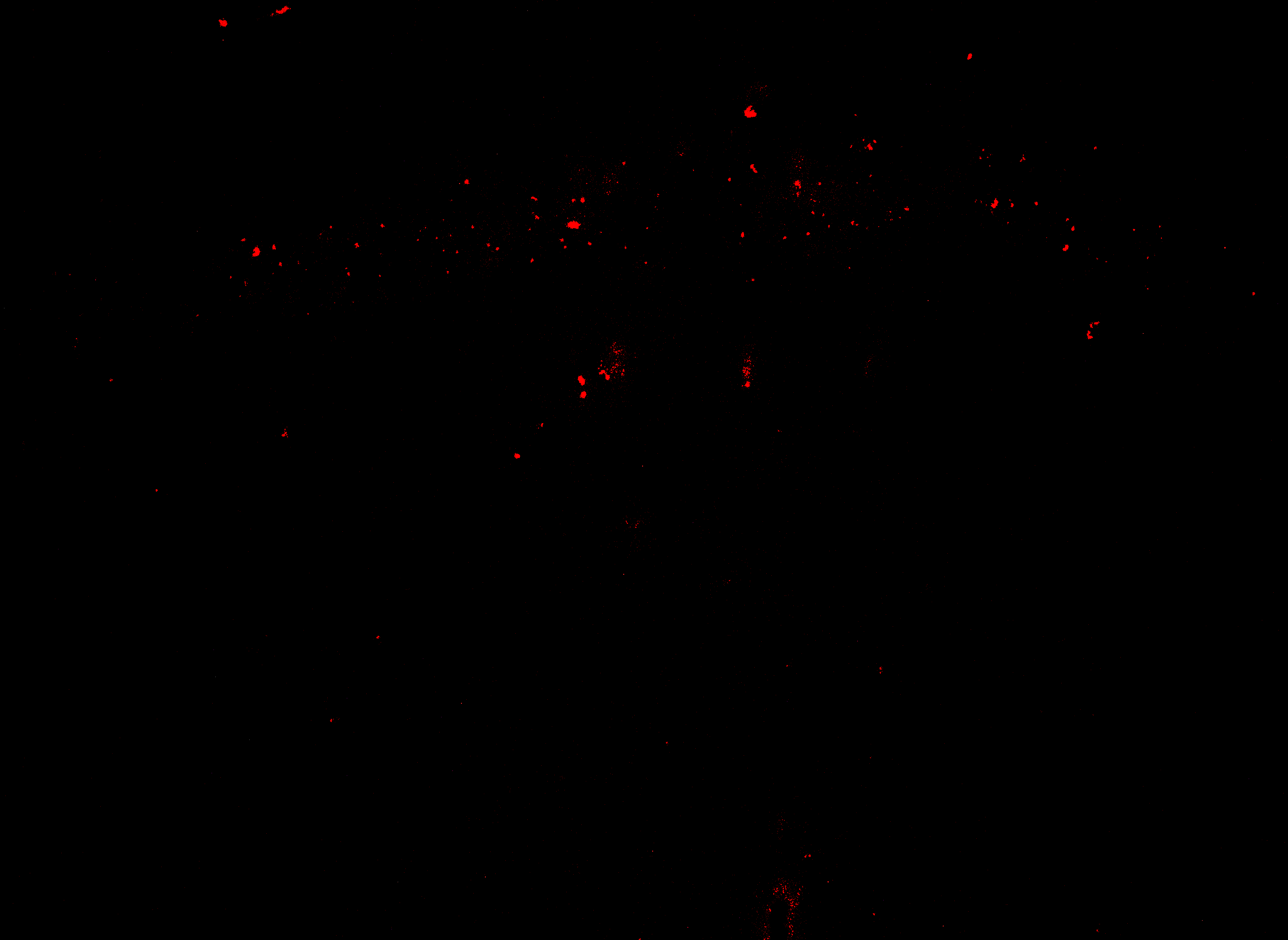

Supplement: Supplementary file 6 — Source data Fig. 4 [file 44321_2026_422_MOESM6_ESM.zip › Figure 4/Figure 4R/dbdb;Aars2 cKO/Tunel.tif]

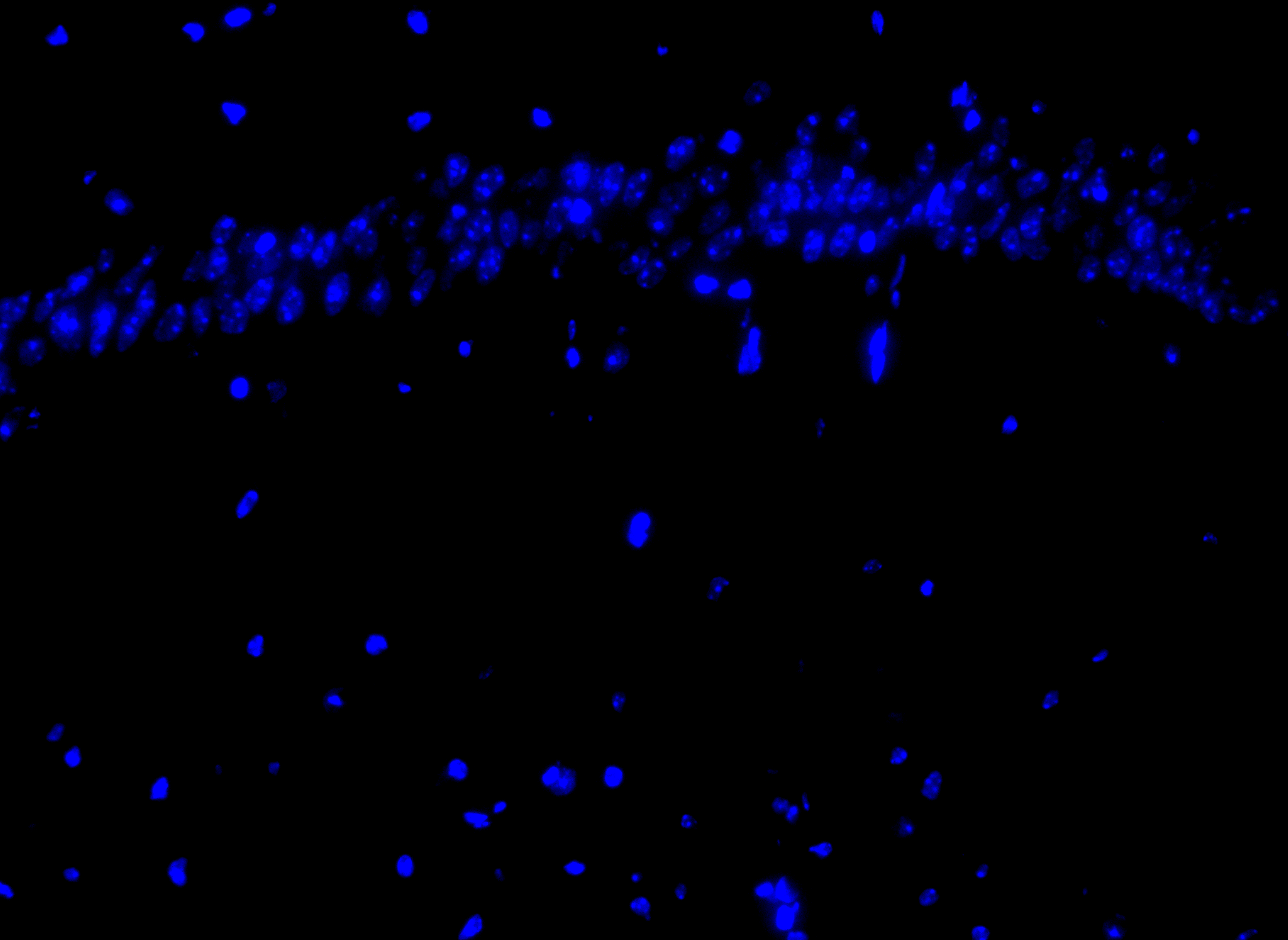

Supplement: Supplementary file 6 — Source data Fig. 4 [file 44321_2026_422_MOESM6_ESM.zip › Figure 4/Figure 4R/dbdb;Aars2 cKO/DAPI.tif]

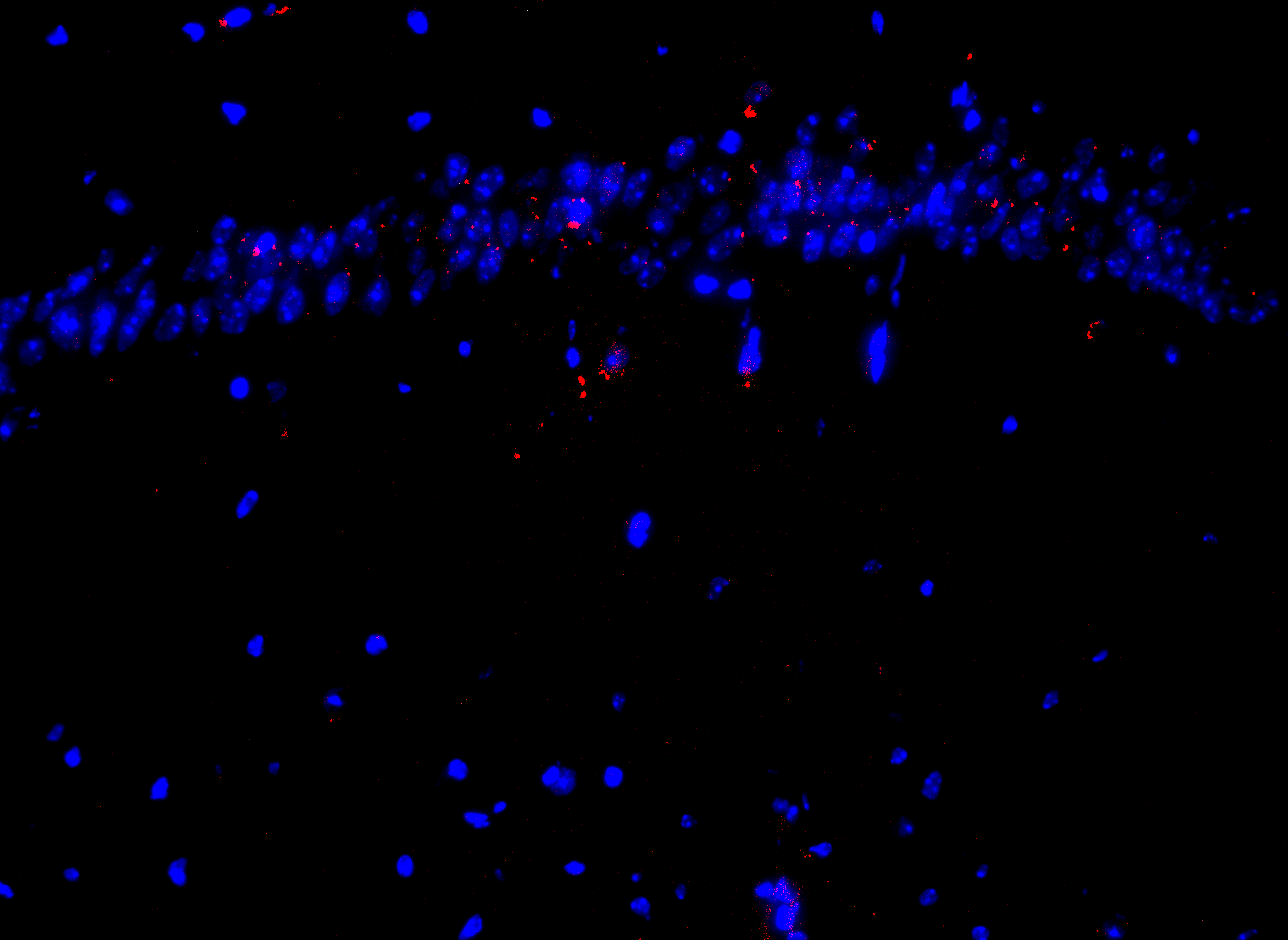

Supplement: Supplementary file 6 — Source data Fig. 4 [file 44321_2026_422_MOESM6_ESM.zip › Figure 4/Figure 4R/dbdb;Aars2 cKO/Merge.tif]

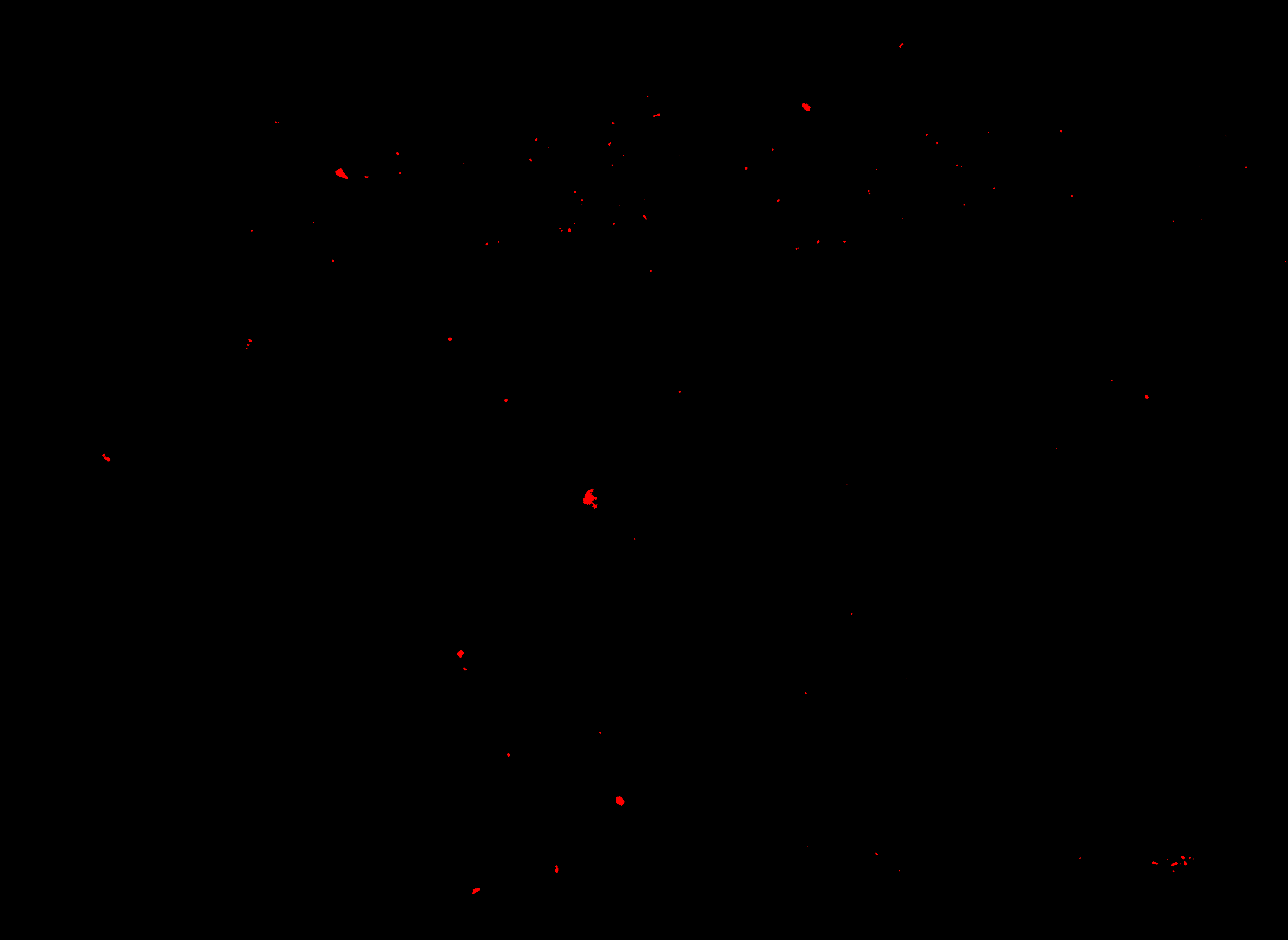

Supplement: Supplementary file 6 — Source data Fig. 4 [file 44321_2026_422_MOESM6_ESM.zip › Figure 4/Figure 4R/dbm;Aars2 cKO/Tunel.tif]

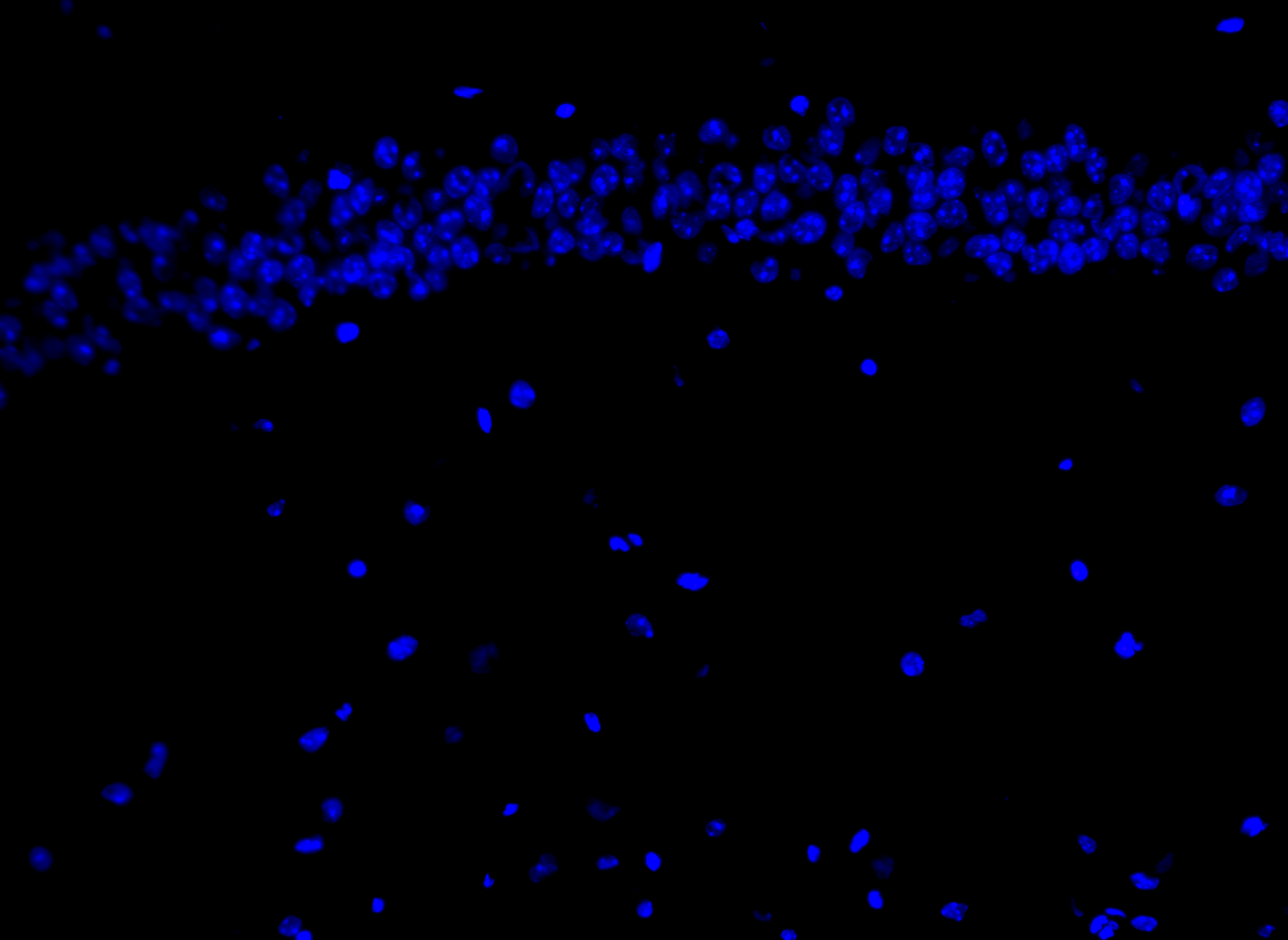

Supplement: Supplementary file 6 — Source data Fig. 4 [file 44321_2026_422_MOESM6_ESM.zip › Figure 4/Figure 4R/dbm;Aars2 cKO/DAPI.tif]

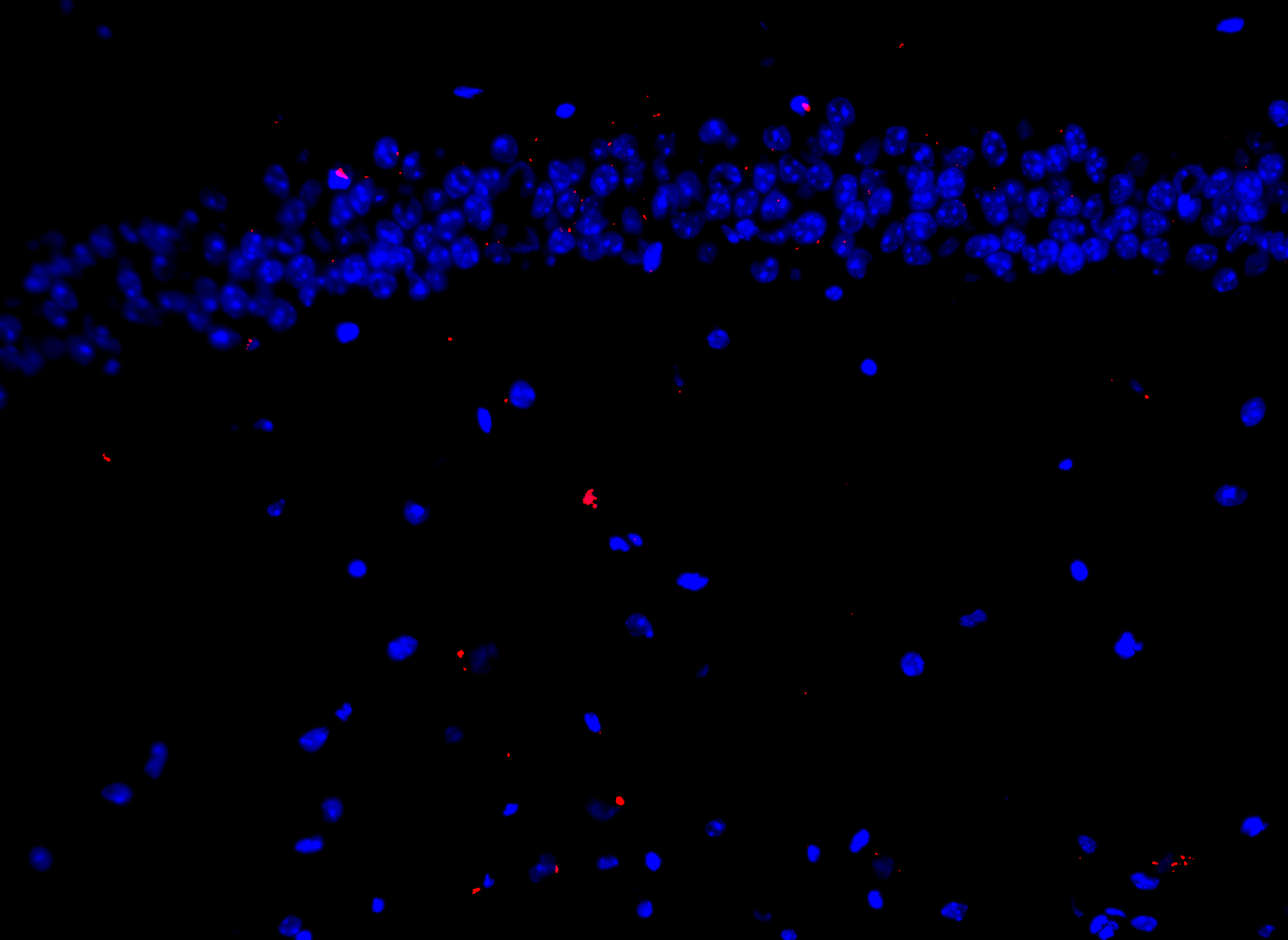

Supplement: Supplementary file 6 — Source data Fig. 4 [file 44321_2026_422_MOESM6_ESM.zip › Figure 4/Figure 4R/dbm;Aars2 cKO/Merge.tif]

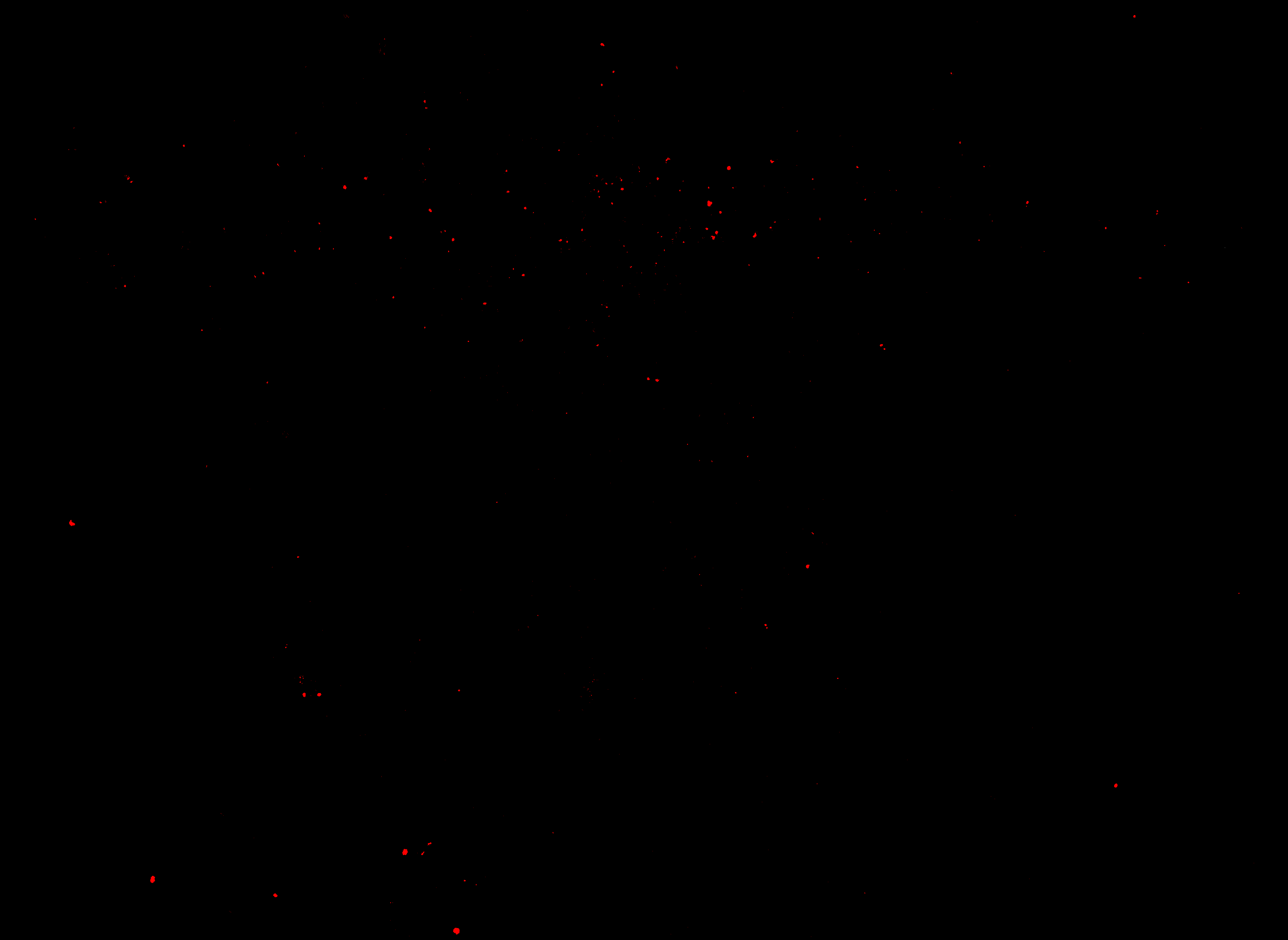

Supplement: Supplementary file 6 — Source data Fig. 4 [file 44321_2026_422_MOESM6_ESM.zip › Figure 4/Figure 4R/dbm;Aars2 flfl/Tunel.tif]

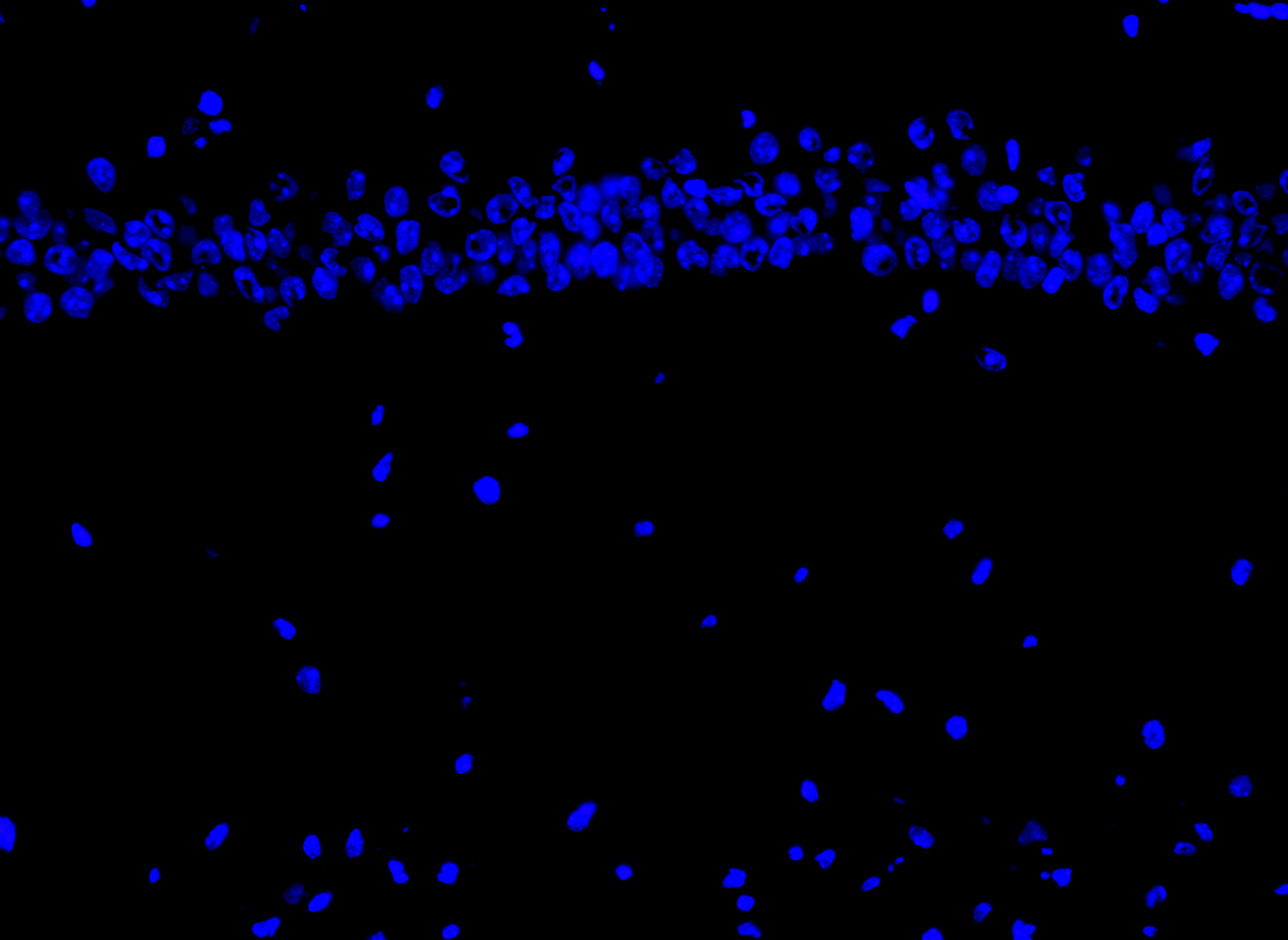

Supplement: Supplementary file 6 — Source data Fig. 4 [file 44321_2026_422_MOESM6_ESM.zip › Figure 4/Figure 4R/dbm;Aars2 flfl/DAPI.tif]

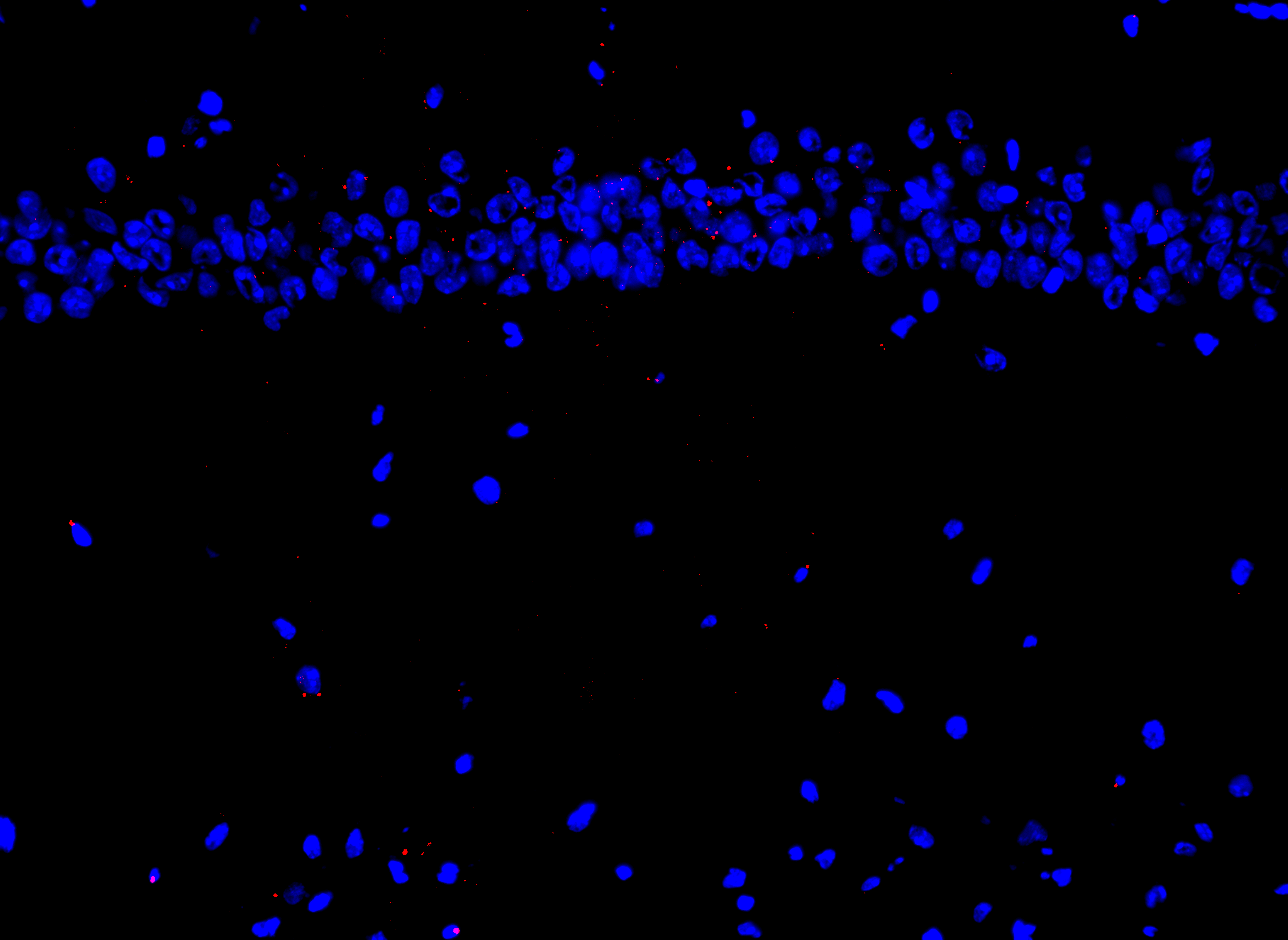

Supplement: Supplementary file 6 — Source data Fig. 4 [file 44321_2026_422_MOESM6_ESM.zip › Figure 4/Figure 4R/dbm;Aars2 flfl/Merge.tif]

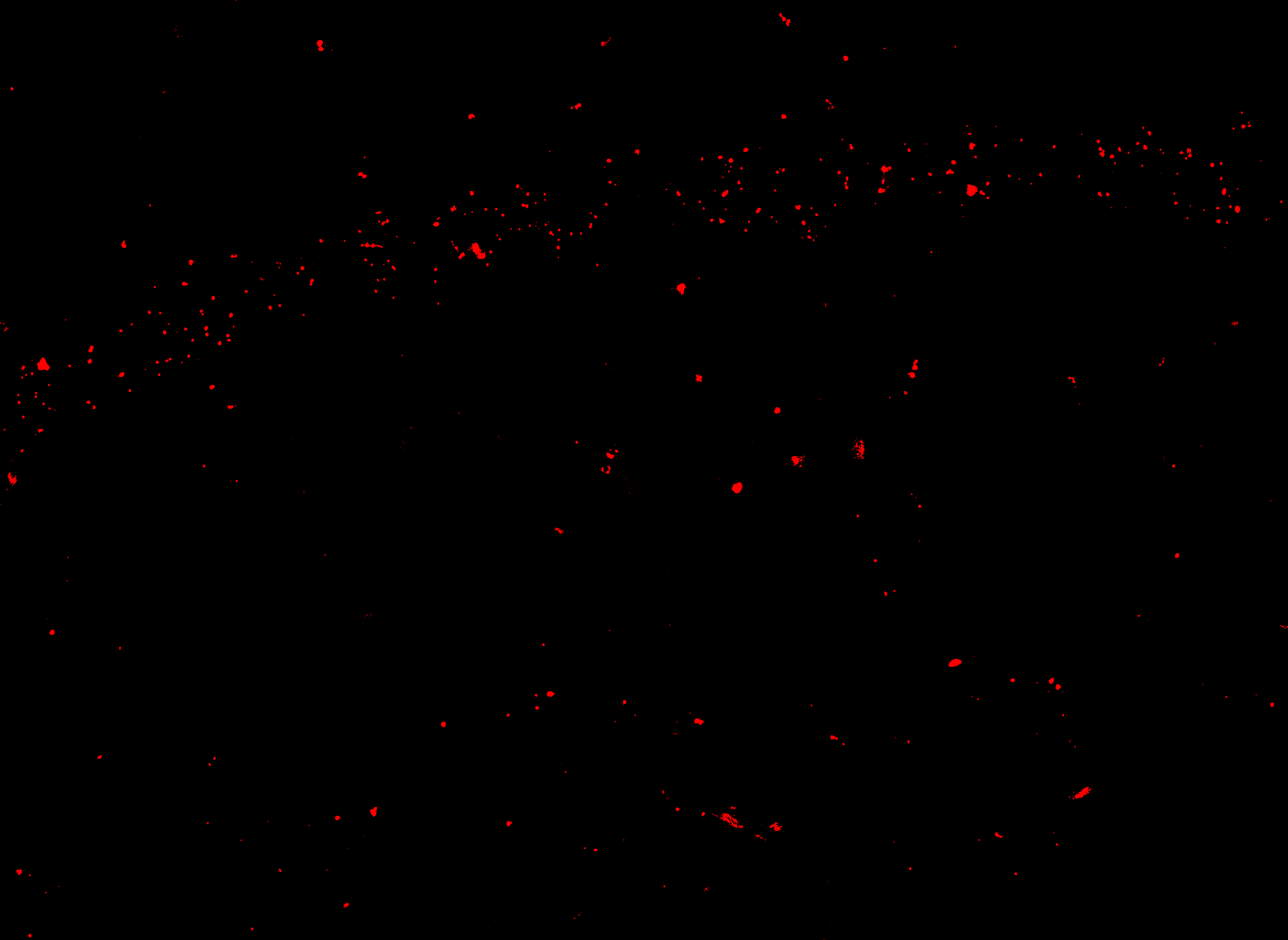

Supplement: Supplementary file 6 — Source data Fig. 4 [file 44321_2026_422_MOESM6_ESM.zip › Figure 4/Figure 4R/dbdb;Aars2 flfl/Tunel.tif]

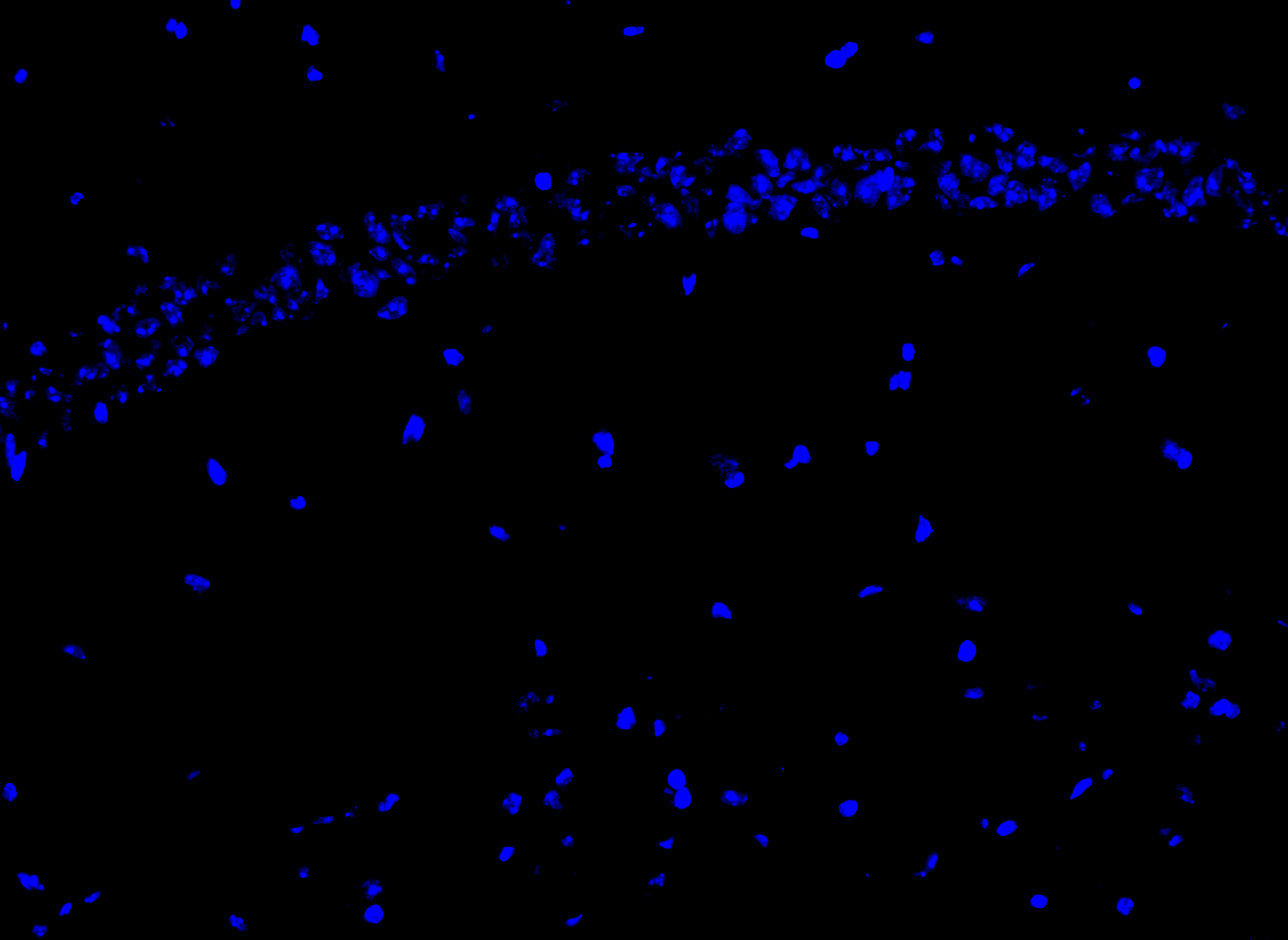

Supplement: Supplementary file 6 — Source data Fig. 4 [file 44321_2026_422_MOESM6_ESM.zip › Figure 4/Figure 4R/dbdb;Aars2 flfl/DAPI.tif]

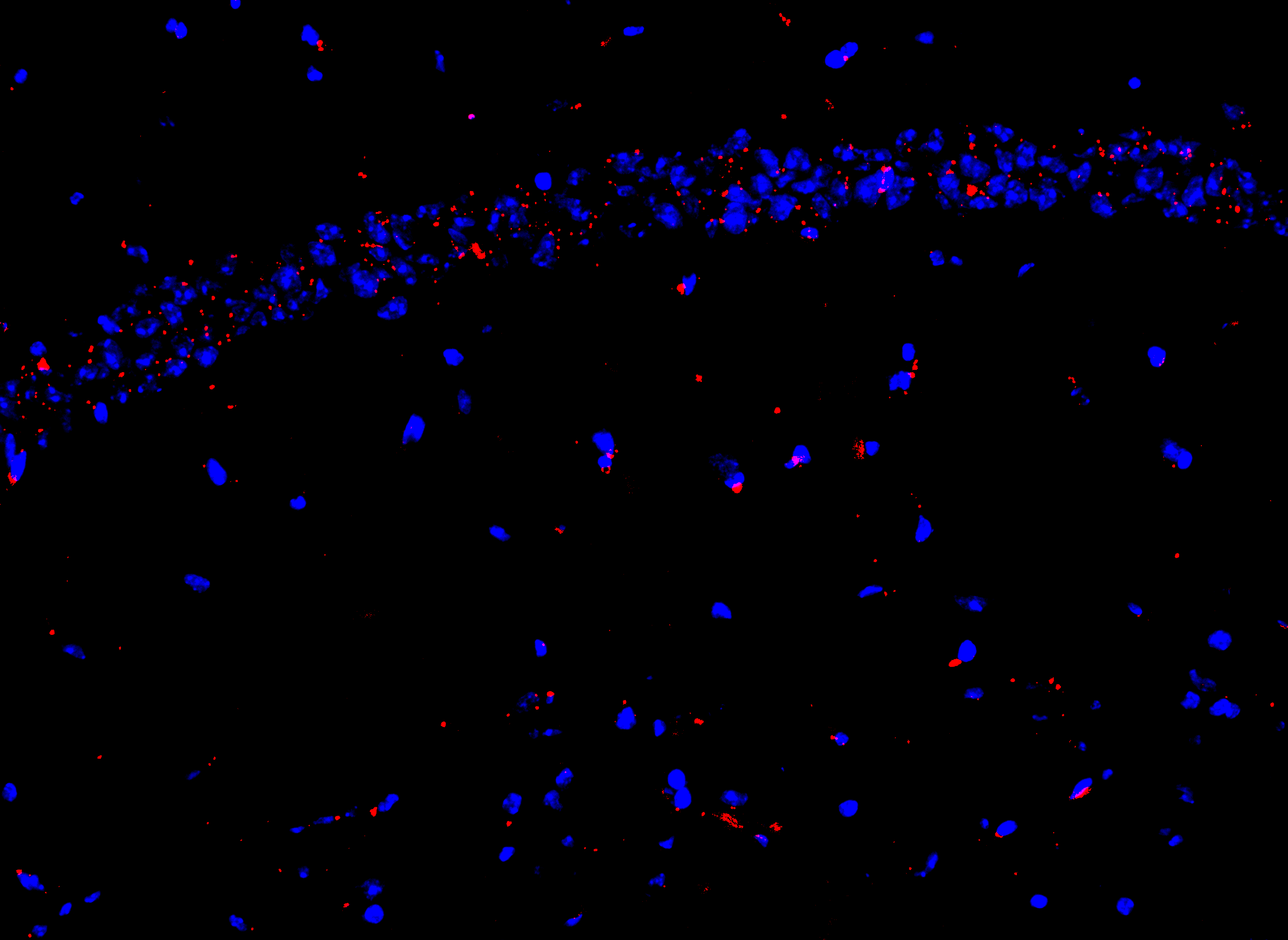

Supplement: Supplementary file 6 — Source data Fig. 4 [file 44321_2026_422_MOESM6_ESM.zip › Figure 4/Figure 4R/dbdb;Aars2 flfl/Merge.tif]

## IP

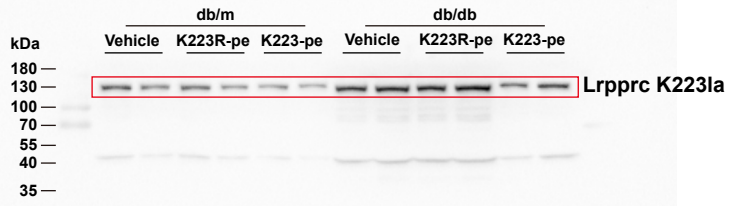

## Input

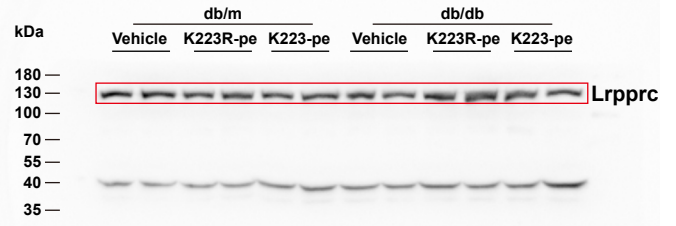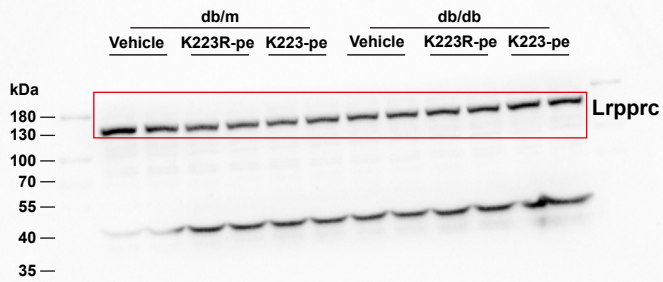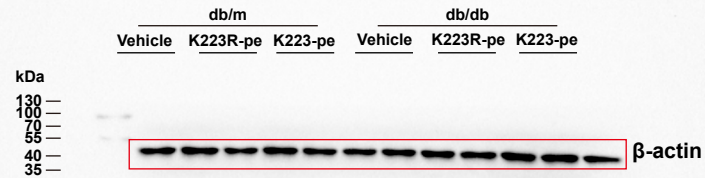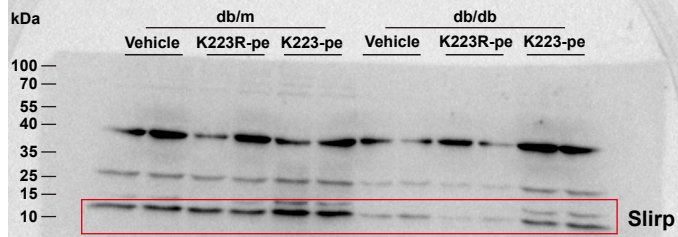

Supplement: Supplementary file 7 — Source data Fig. 5 [file 44321_2026_422_MOESM7_ESM.zip › Figure 5/Figure 5C/Figure 5C.pdf]

# IP

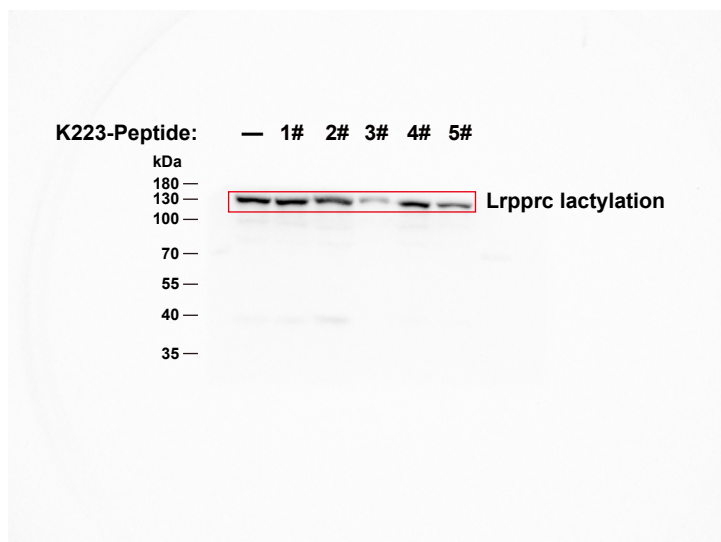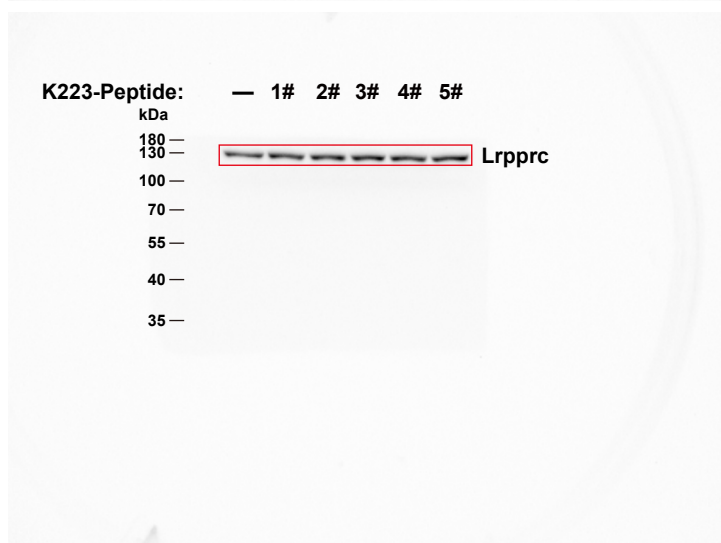

Supplement: Supplementary file 7 — Source data Fig. 5 [file 44321_2026_422_MOESM7_ESM.zip › Figure 5/Figure 5B/Figure 5B.pdf]

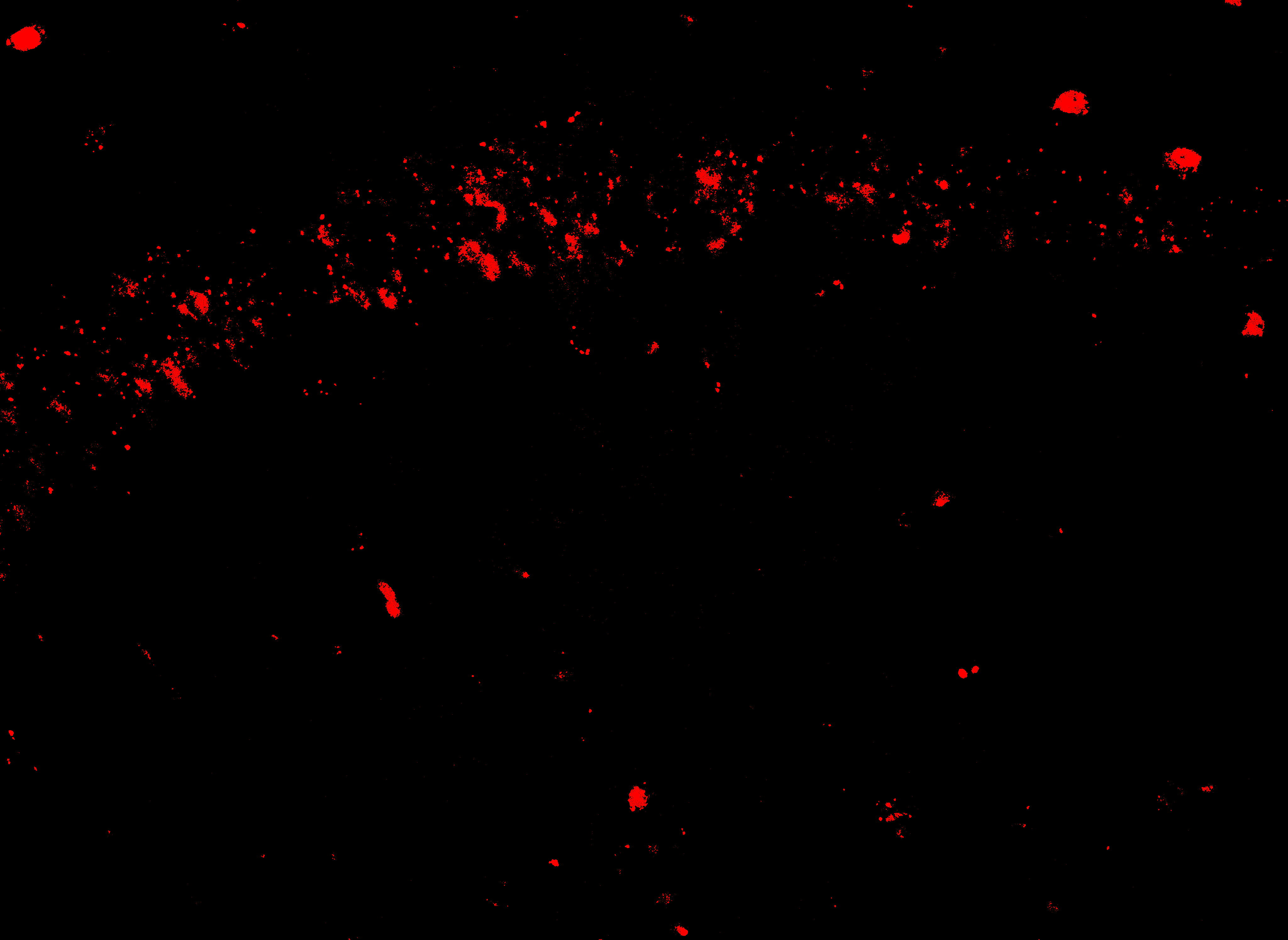

Supplement: Supplementary file 8 — Source data Fig. 6 [file 44321_2026_422_MOESM8_ESM.zip › Figure 6/Figure 6A/dbdb+vehicle/Tunel.tif]

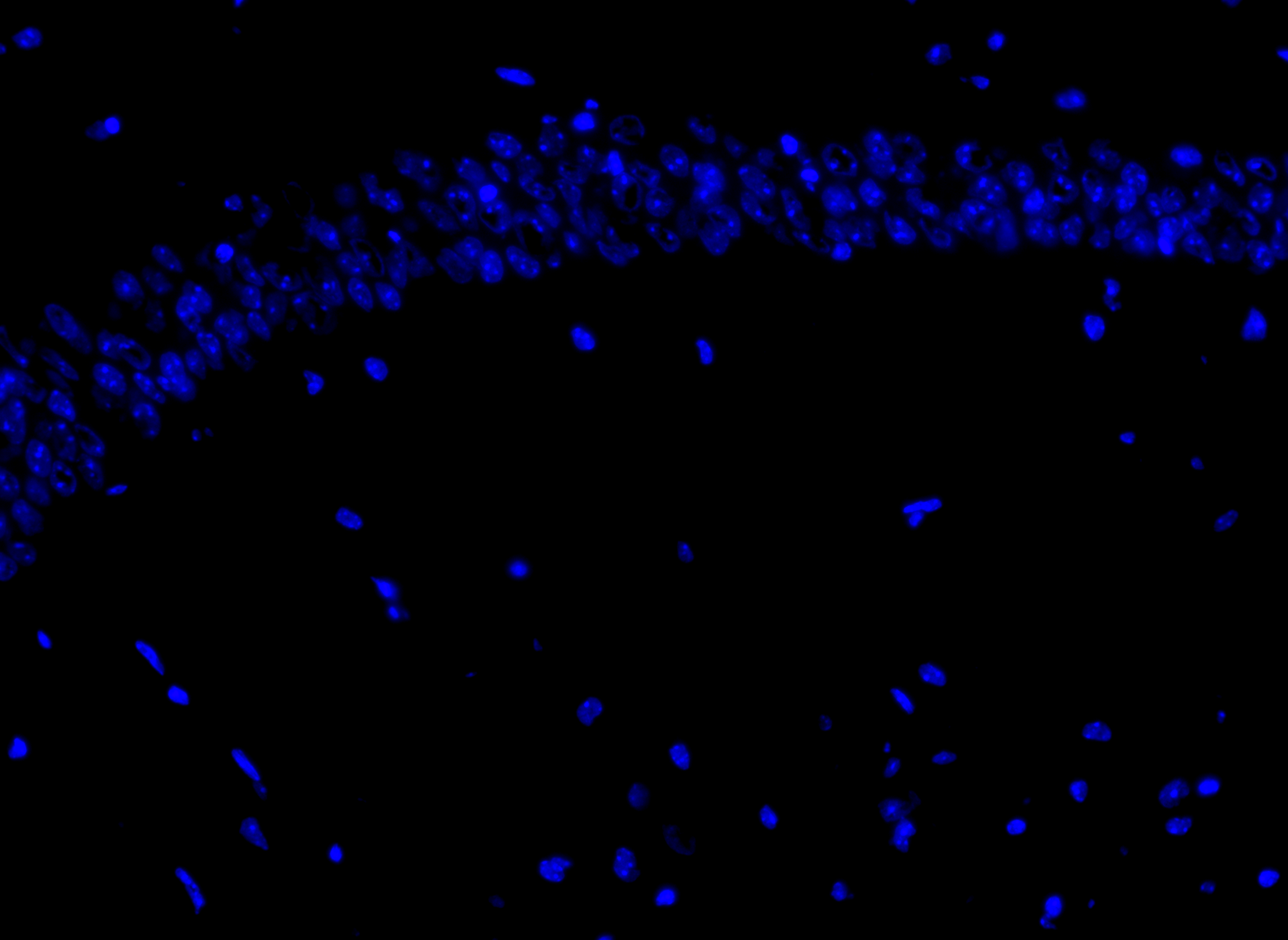

Supplement: Supplementary file 8 — Source data Fig. 6 [file 44321_2026_422_MOESM8_ESM.zip › Figure 6/Figure 6A/dbdb+vehicle/DAPI.tif]

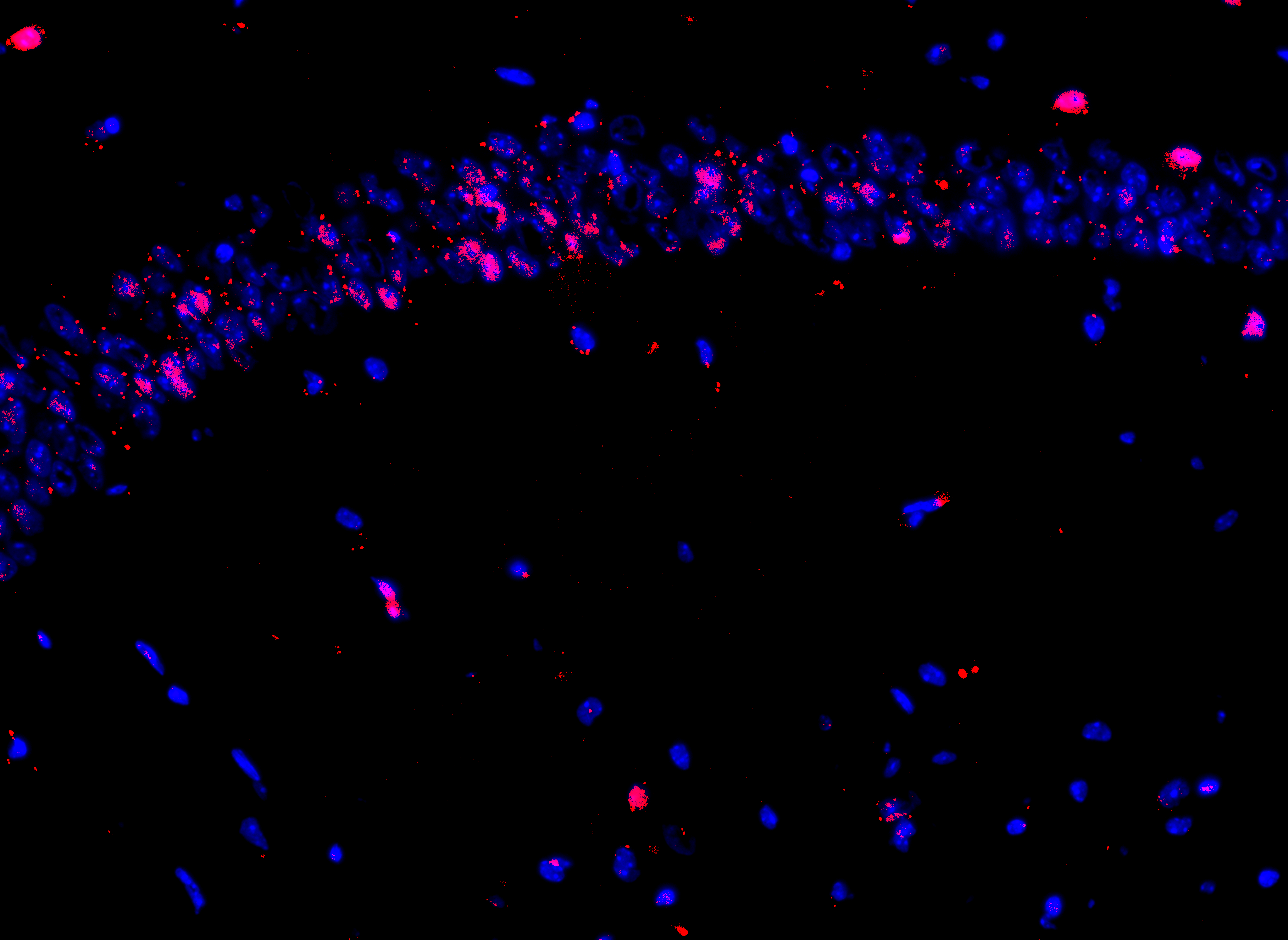

Supplement: Supplementary file 8 — Source data Fig. 6 [file 44321_2026_422_MOESM8_ESM.zip › Figure 6/Figure 6A/dbdb+vehicle/Merge.tif]

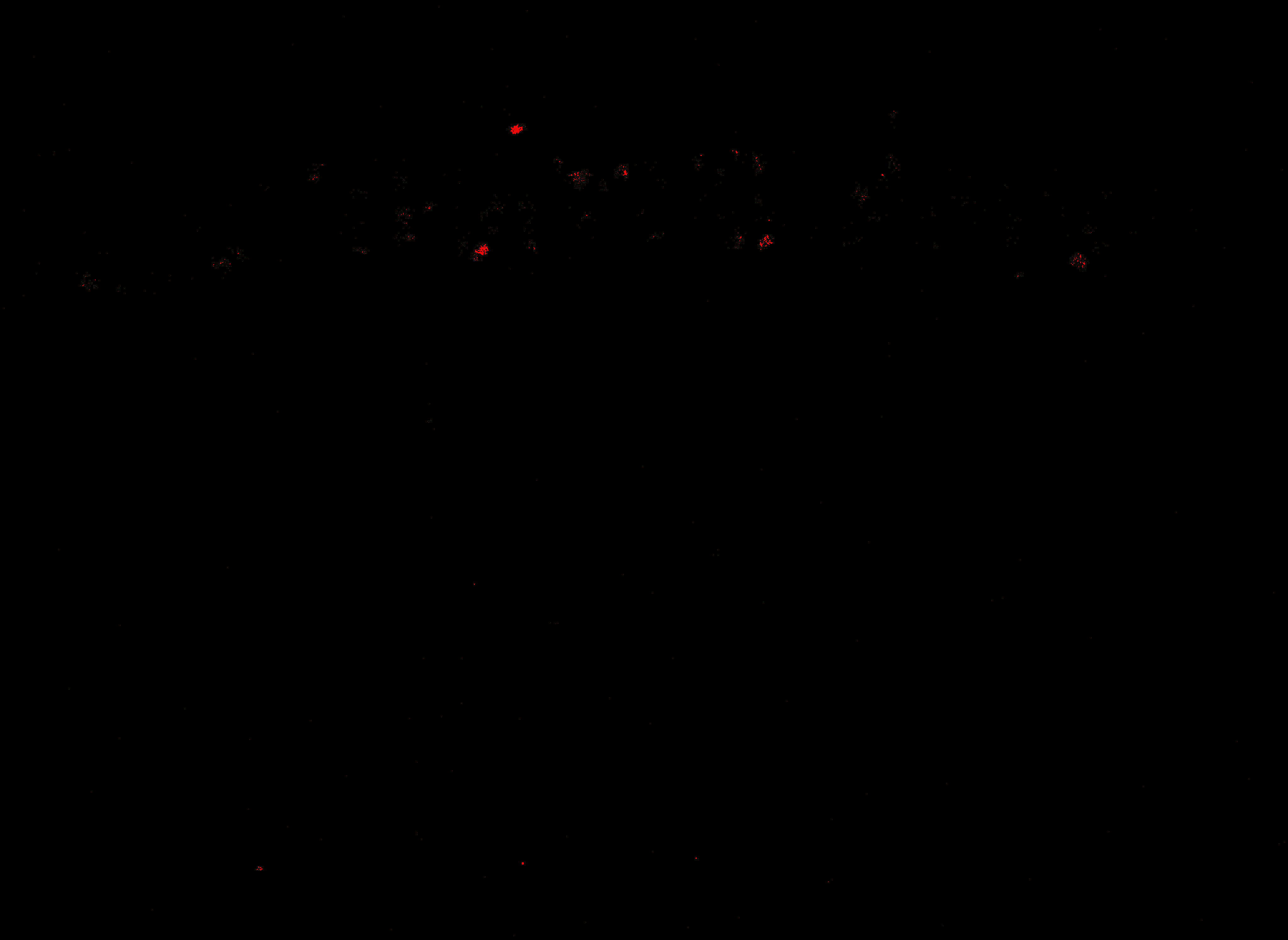

Supplement: Supplementary file 8 — Source data Fig. 6 [file 44321_2026_422_MOESM8_ESM.zip › Figure 6/Figure 6A/dbm+K223-pe/Tunel.tif]

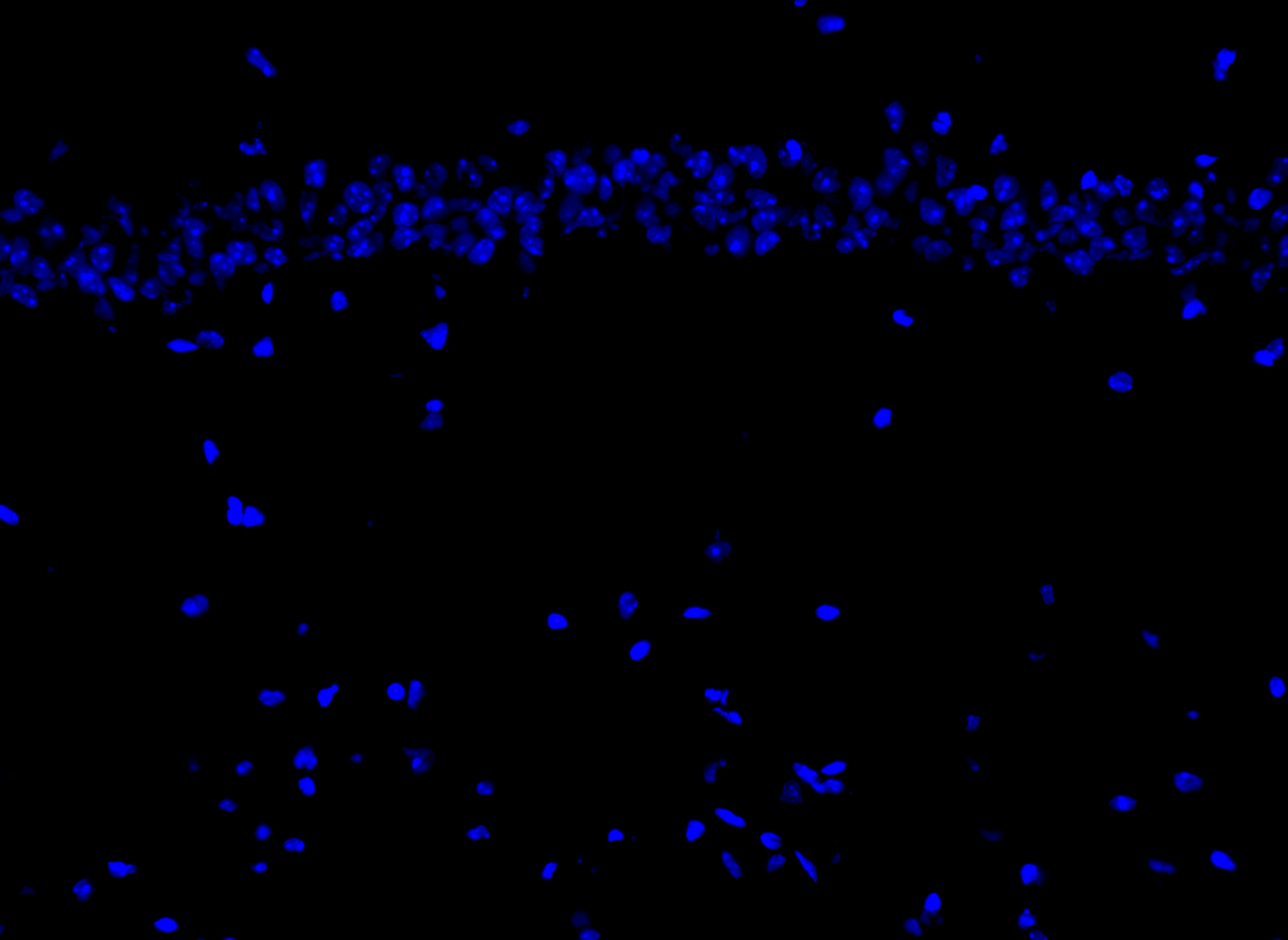

Supplement: Supplementary file 8 — Source data Fig. 6 [file 44321_2026_422_MOESM8_ESM.zip › Figure 6/Figure 6A/dbm+K223-pe/DAPI.tif]

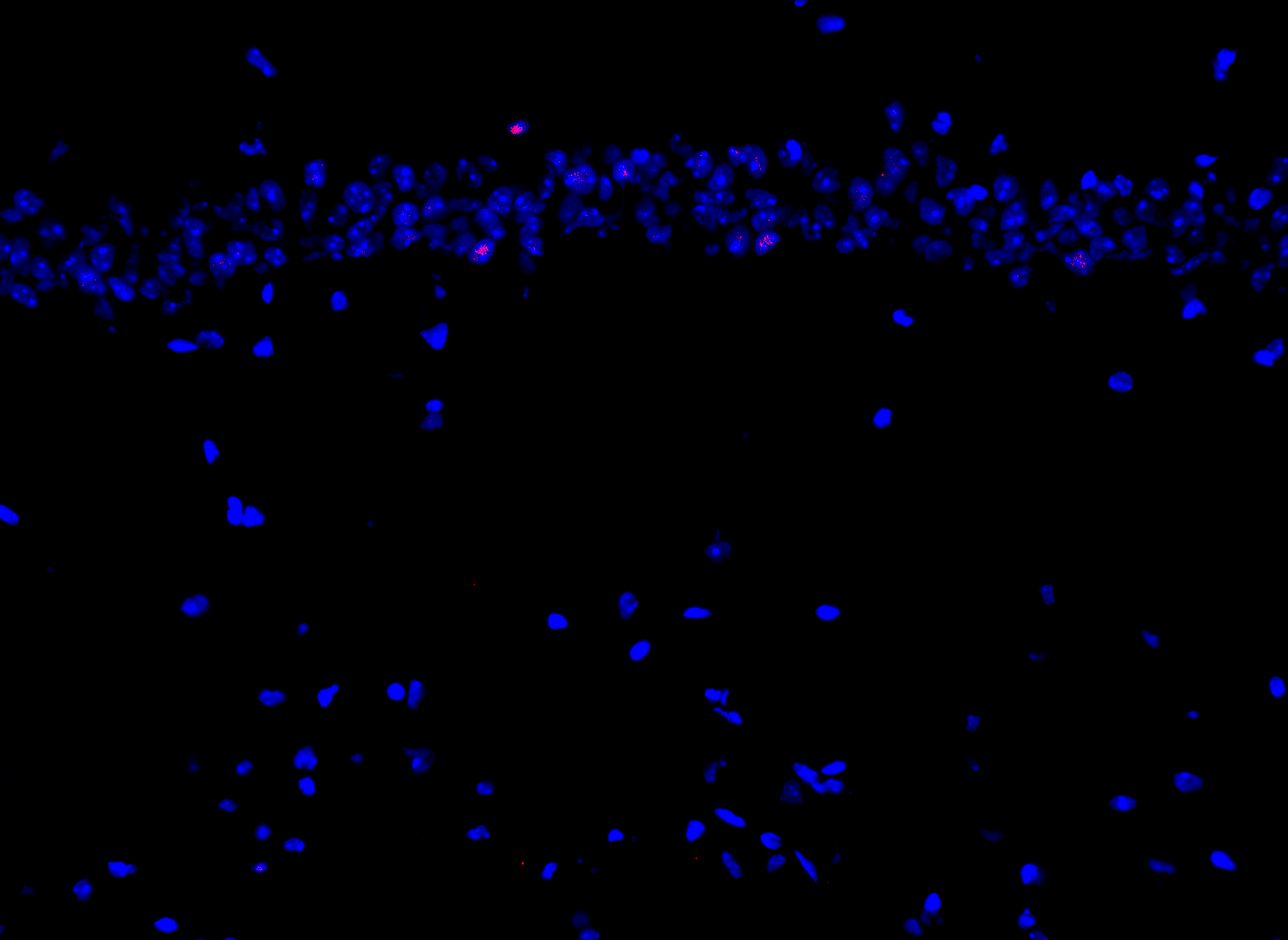

Supplement: Supplementary file 8 — Source data Fig. 6 [file 44321_2026_422_MOESM8_ESM.zip › Figure 6/Figure 6A/dbm+K223-pe/Merge.tif]

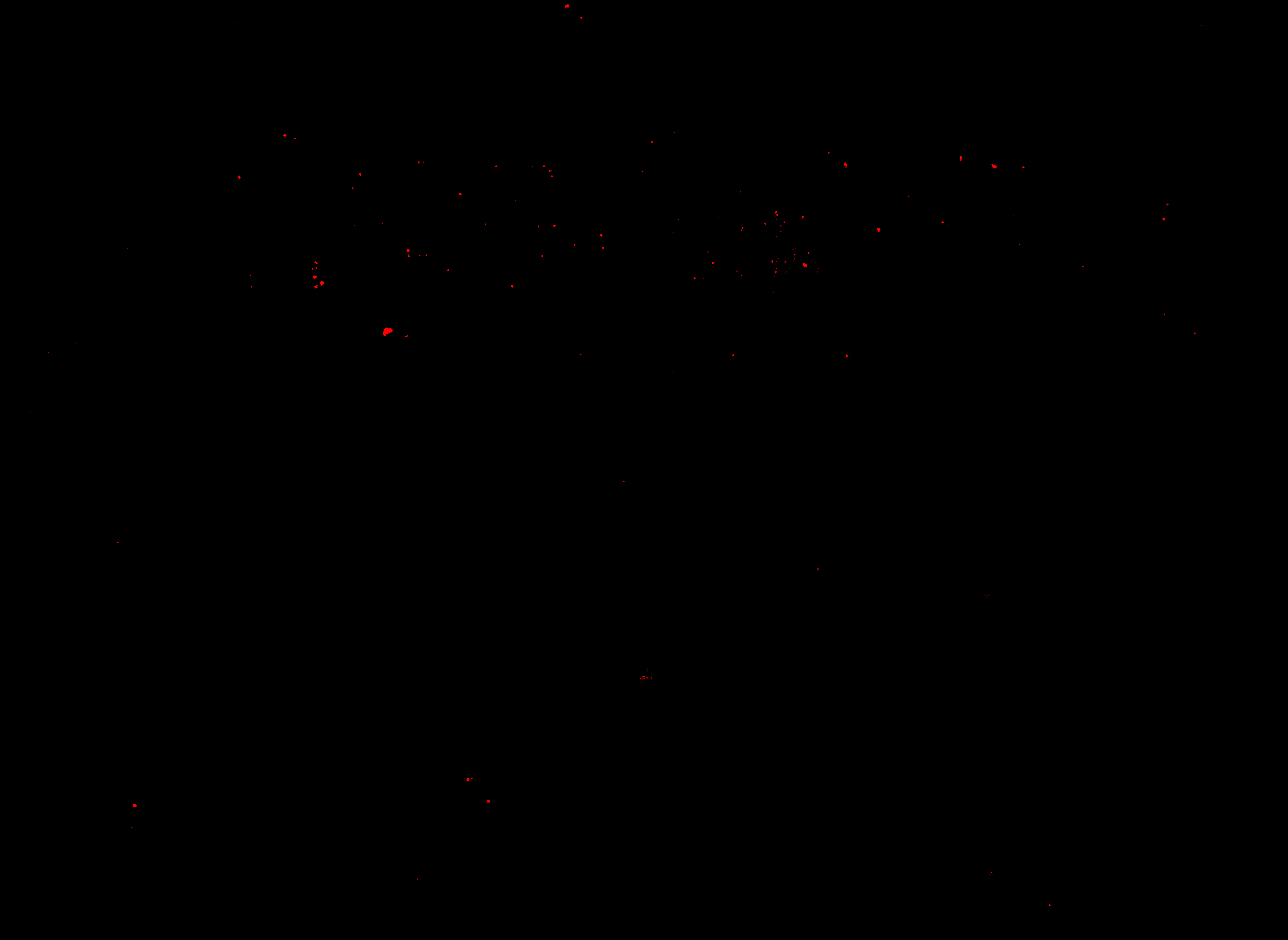

Supplement: Supplementary file 8 — Source data Fig. 6 [file 44321_2026_422_MOESM8_ESM.zip › Figure 6/Figure 6A/dbm+K223R-pe/Tunel.tif]

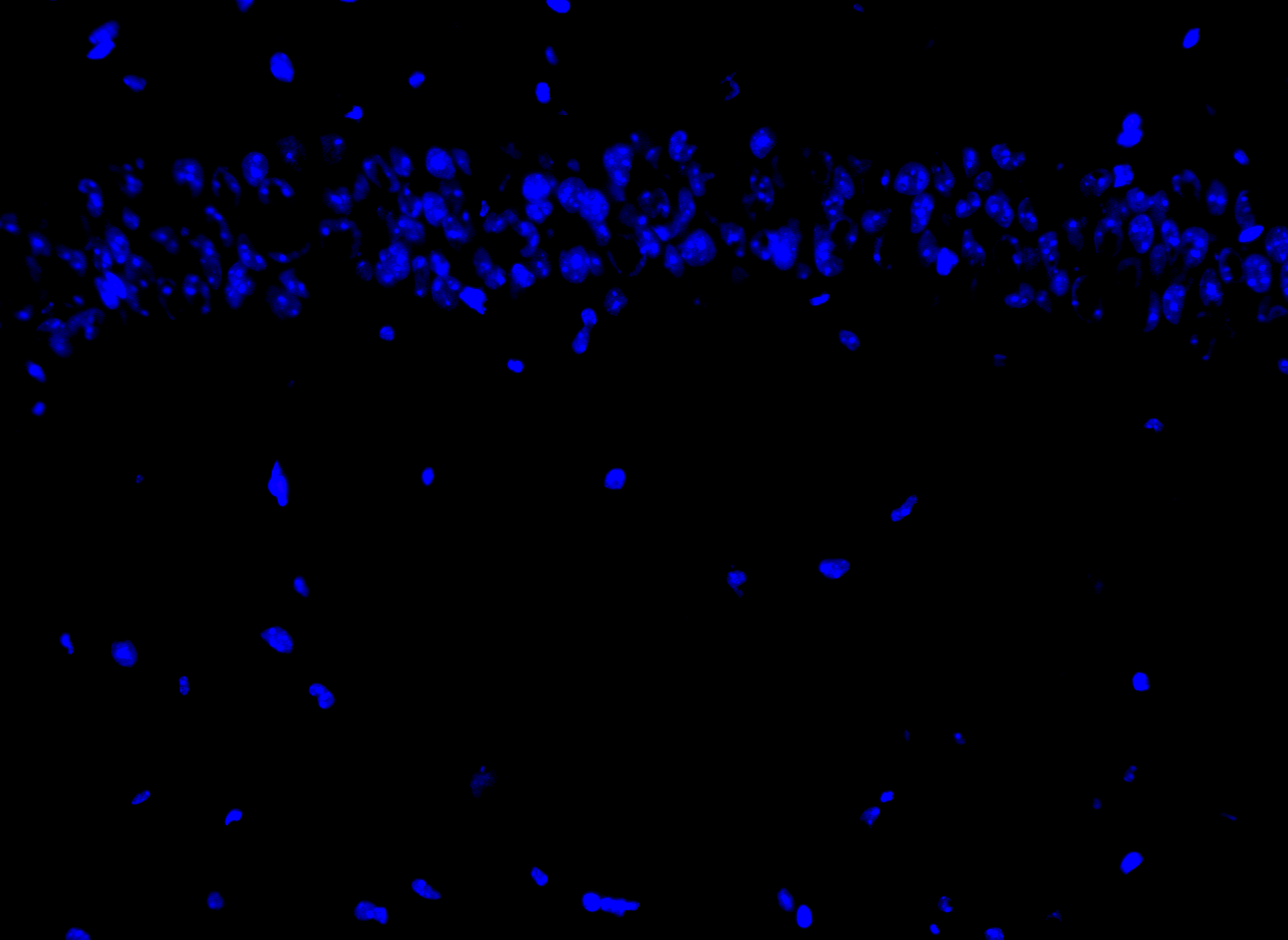

Supplement: Supplementary file 8 — Source data Fig. 6 [file 44321_2026_422_MOESM8_ESM.zip › Figure 6/Figure 6A/dbm+K223R-pe/DAPI.tif]

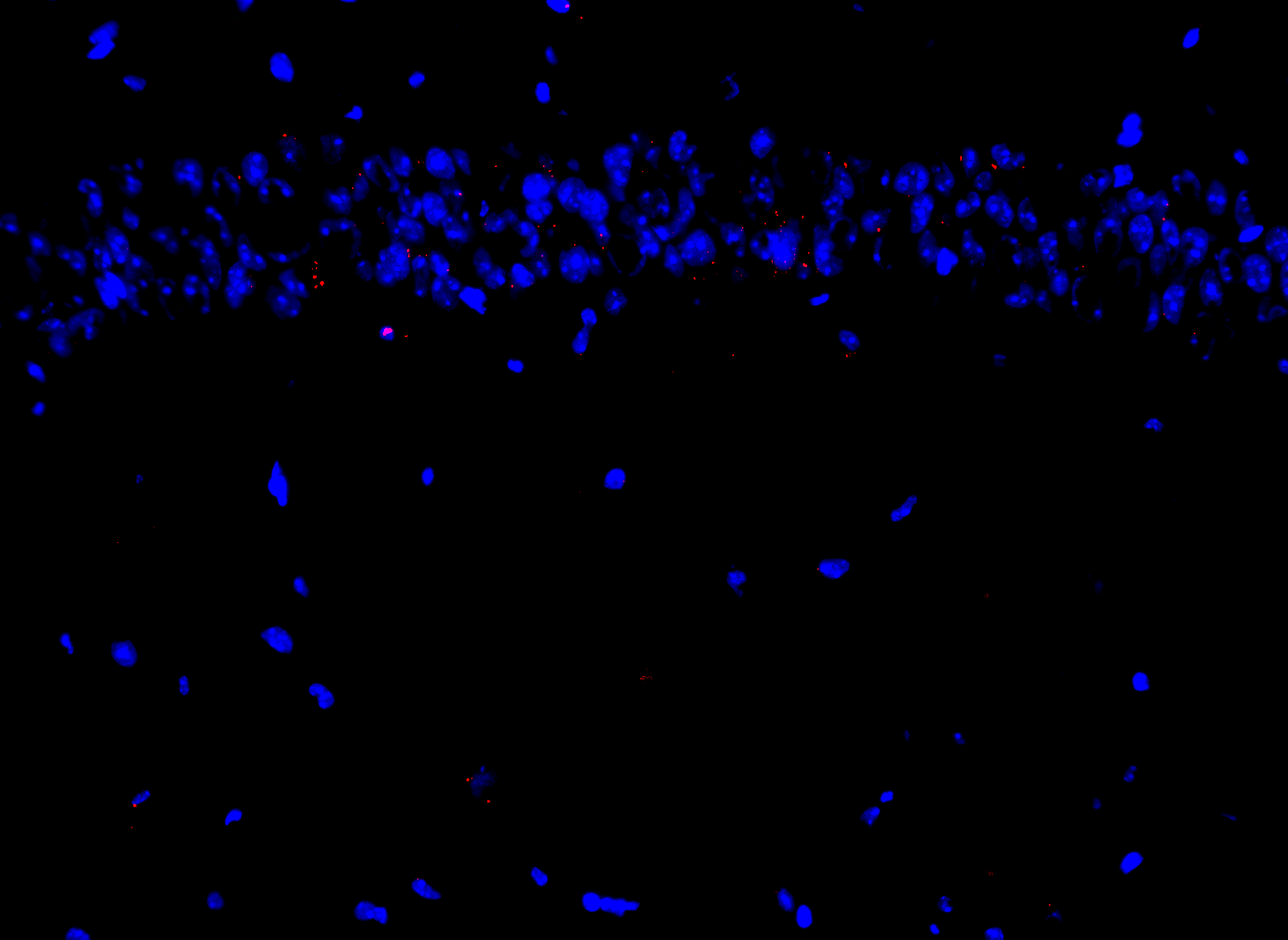

Supplement: Supplementary file 8 — Source data Fig. 6 [file 44321_2026_422_MOESM8_ESM.zip › Figure 6/Figure 6A/dbm+K223R-pe/Merge.tif]

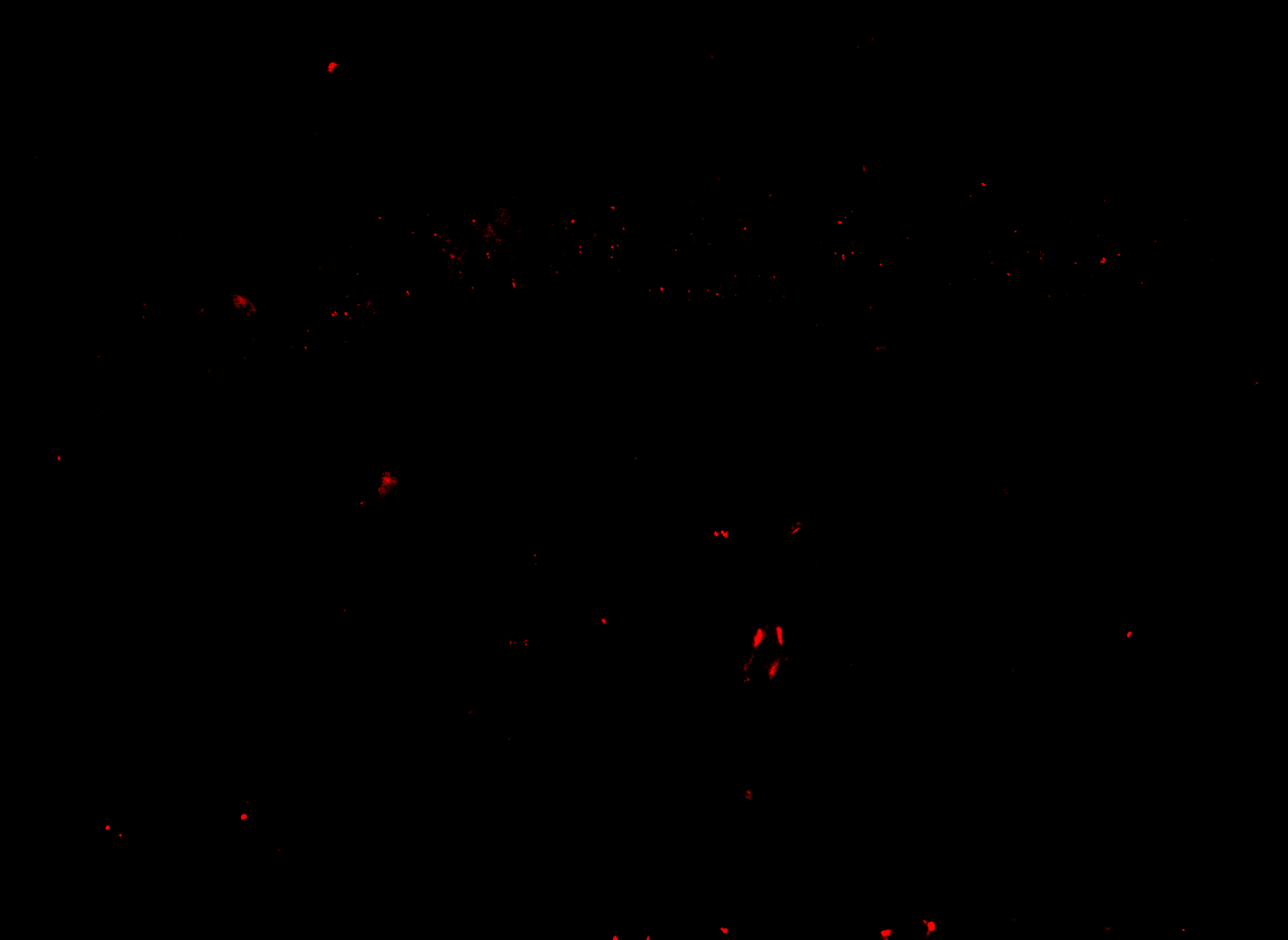

Supplement: Supplementary file 8 — Source data Fig. 6 [file 44321_2026_422_MOESM8_ESM.zip › Figure 6/Figure 6A/dbm+vehicle/Tunel.tif]

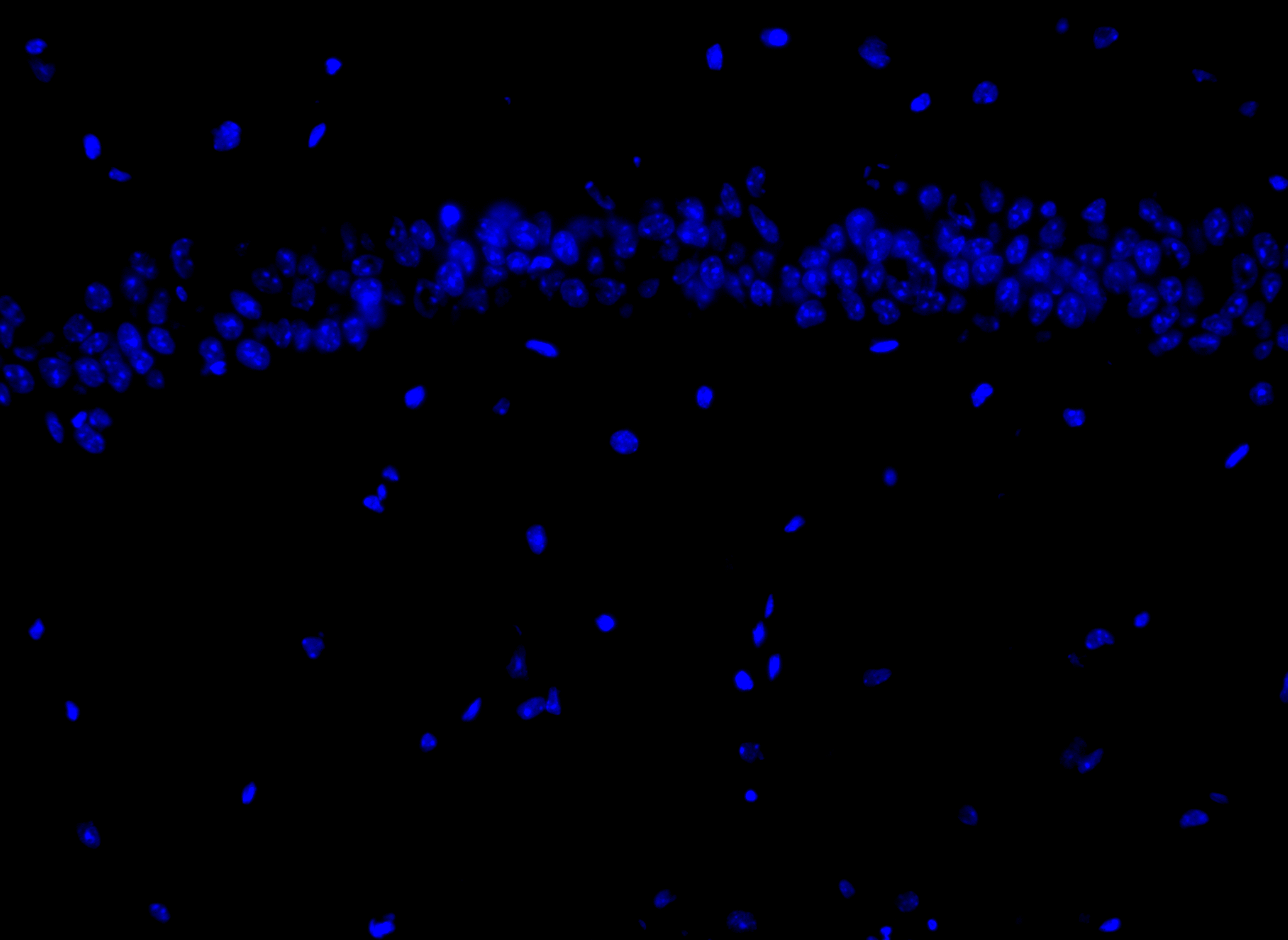

Supplement: Supplementary file 8 — Source data Fig. 6 [file 44321_2026_422_MOESM8_ESM.zip › Figure 6/Figure 6A/dbm+vehicle/DAPI.tif]

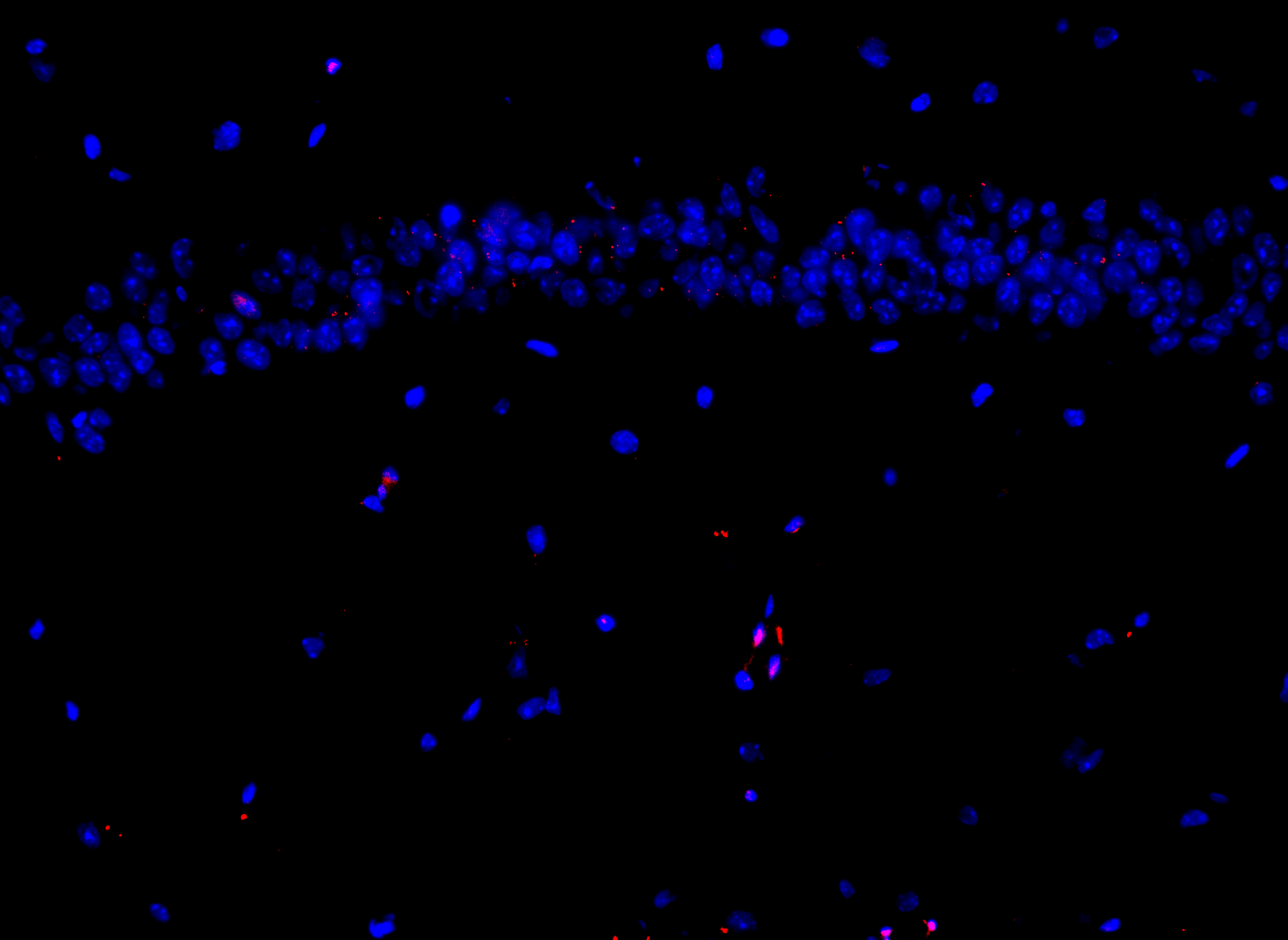

Supplement: Supplementary file 8 — Source data Fig. 6 [file 44321_2026_422_MOESM8_ESM.zip › Figure 6/Figure 6A/dbm+vehicle/Merge.tif]

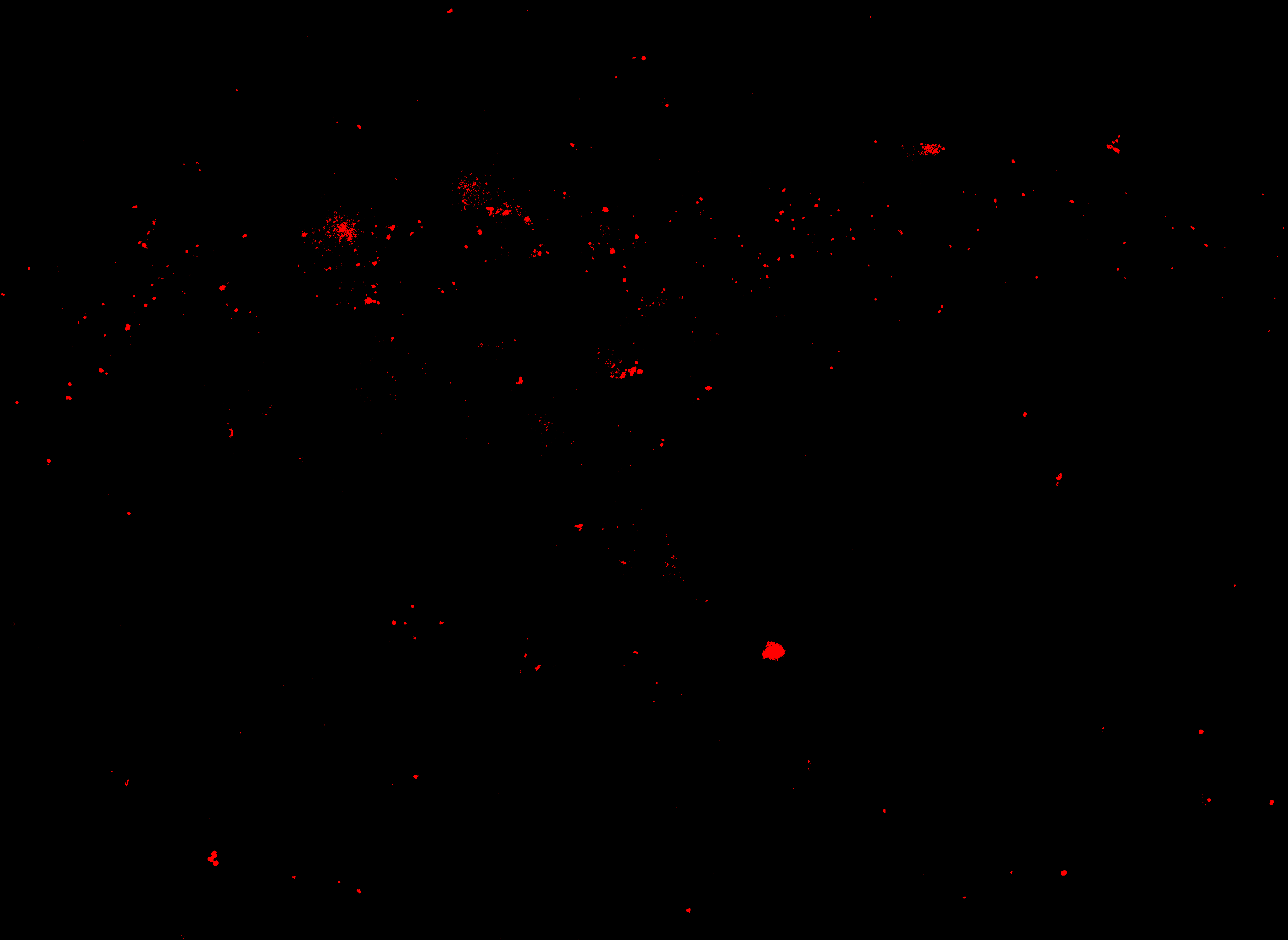

Supplement: Supplementary file 8 — Source data Fig. 6 [file 44321_2026_422_MOESM8_ESM.zip › Figure 6/Figure 6A/dbdb+K223-pe/Tunel.tif]

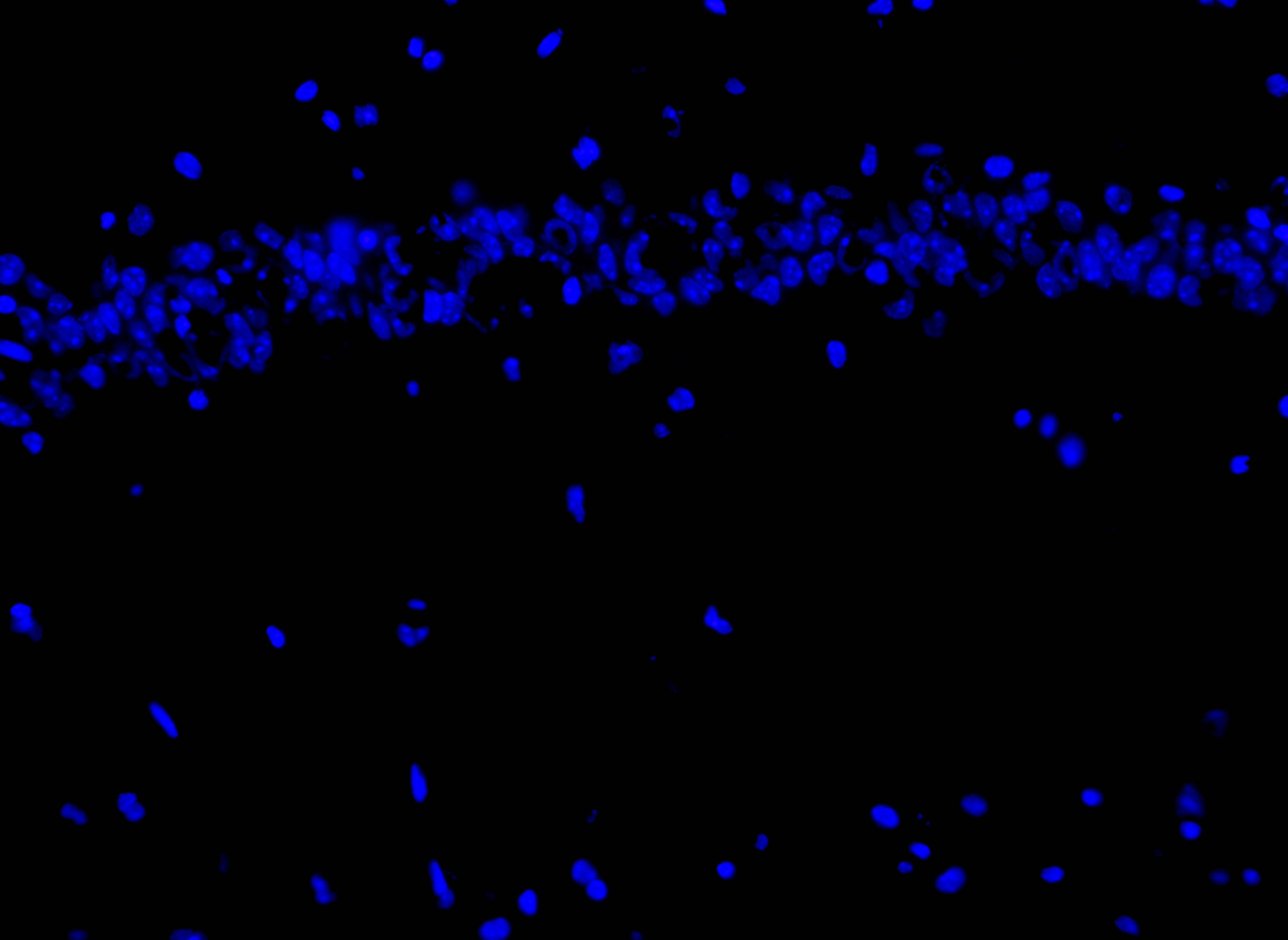

Supplement: Supplementary file 8 — Source data Fig. 6 [file 44321_2026_422_MOESM8_ESM.zip › Figure 6/Figure 6A/dbdb+K223-pe/DAPI.tif]

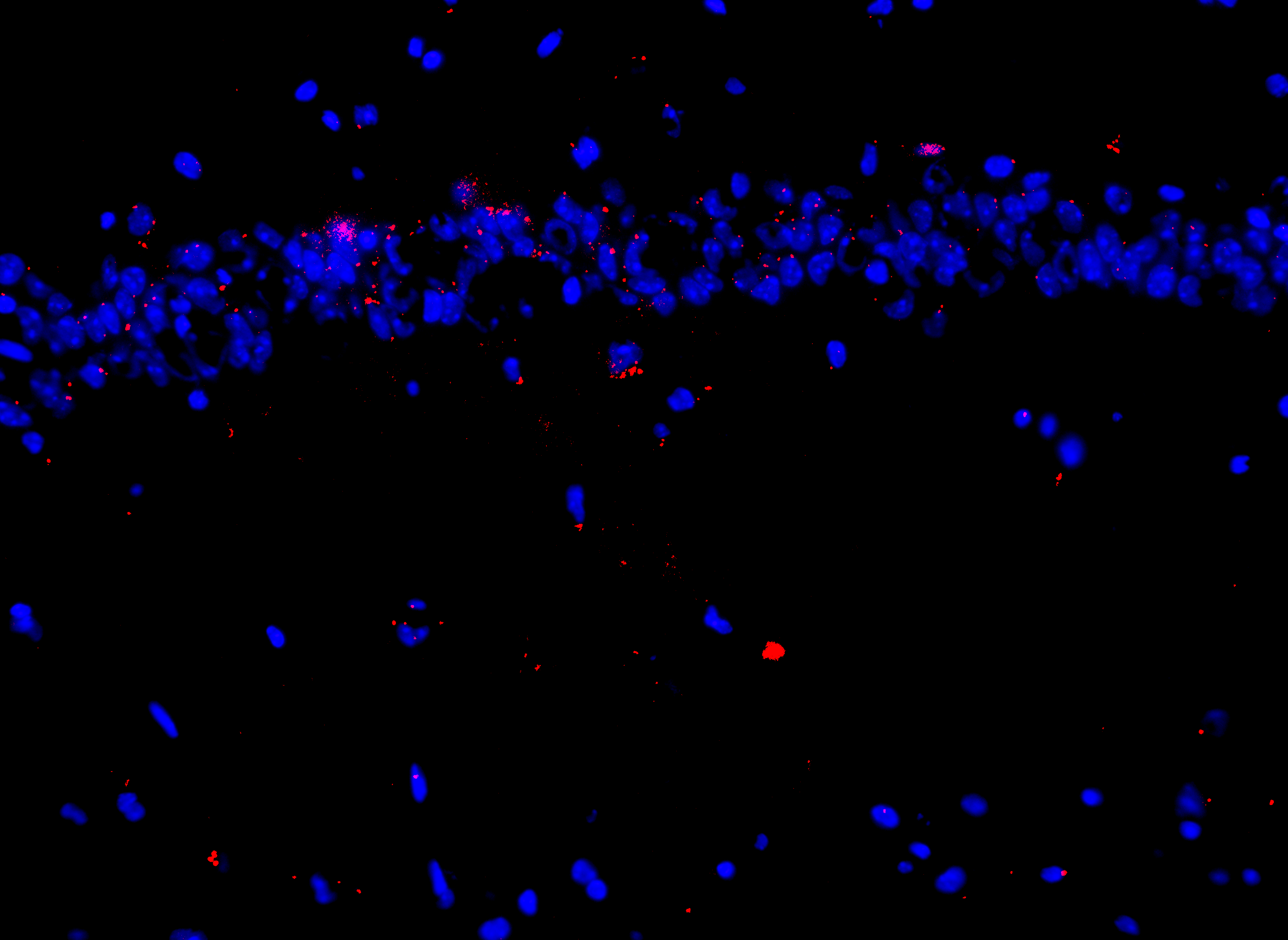

Supplement: Supplementary file 8 — Source data Fig. 6 [file 44321_2026_422_MOESM8_ESM.zip › Figure 6/Figure 6A/dbdb+K223-pe/Merge.tif]

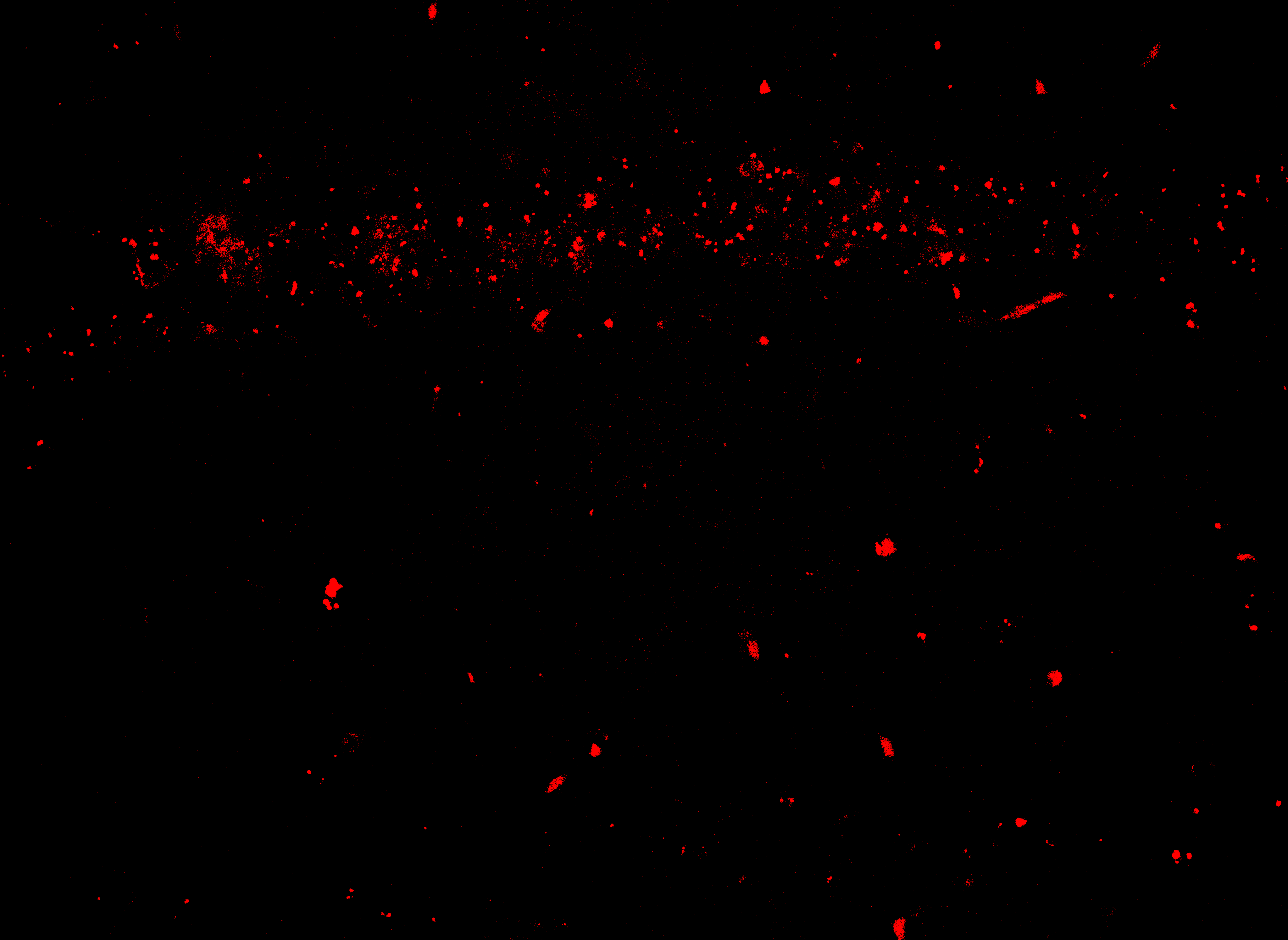

Supplement: Supplementary file 8 — Source data Fig. 6 [file 44321_2026_422_MOESM8_ESM.zip › Figure 6/Figure 6A/dbdb+K223R-pe/Tunel.tif]

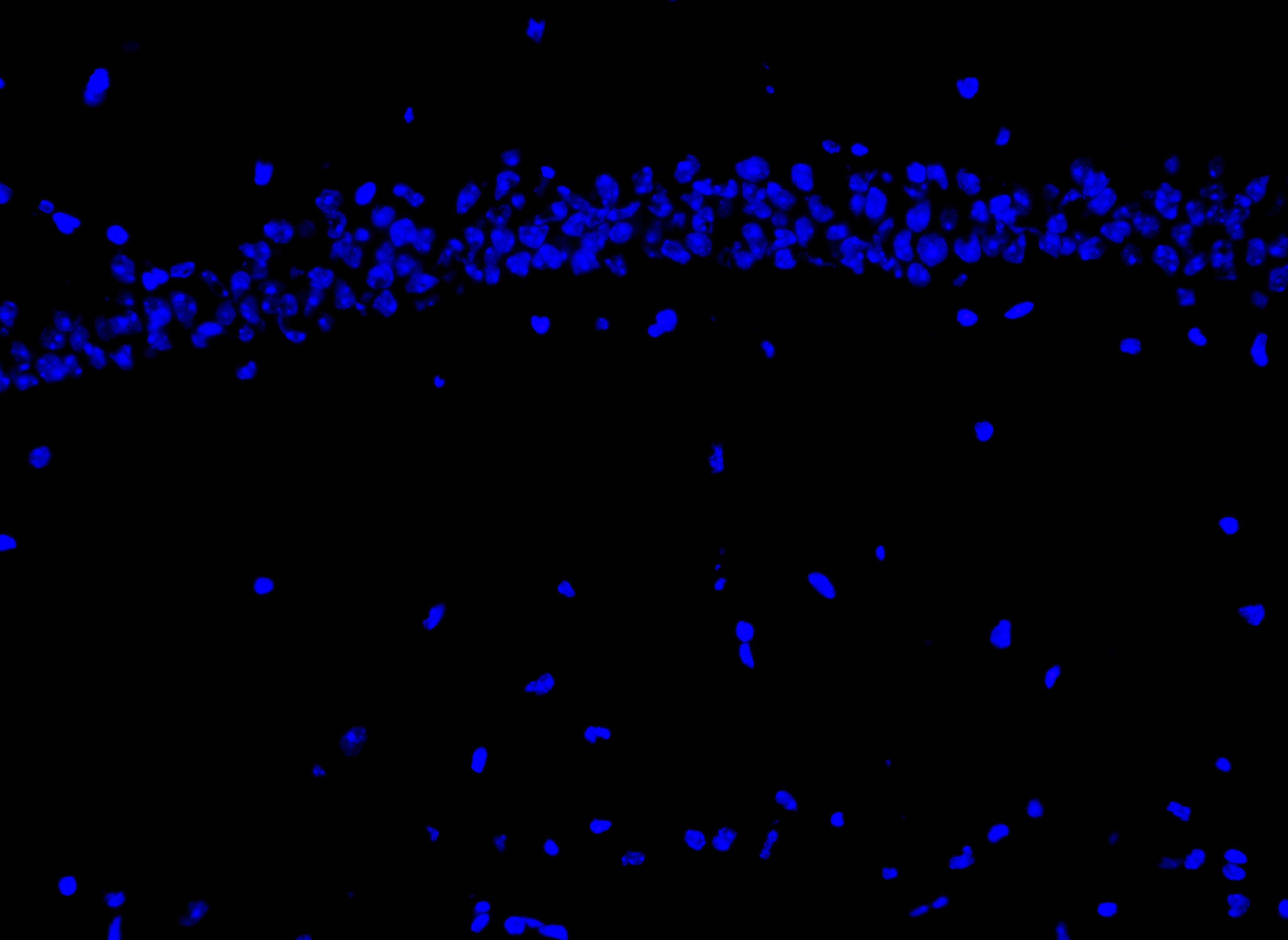

Supplement: Supplementary file 8 — Source data Fig. 6 [file 44321_2026_422_MOESM8_ESM.zip › Figure 6/Figure 6A/dbdb+K223R-pe/DAPI.tif]

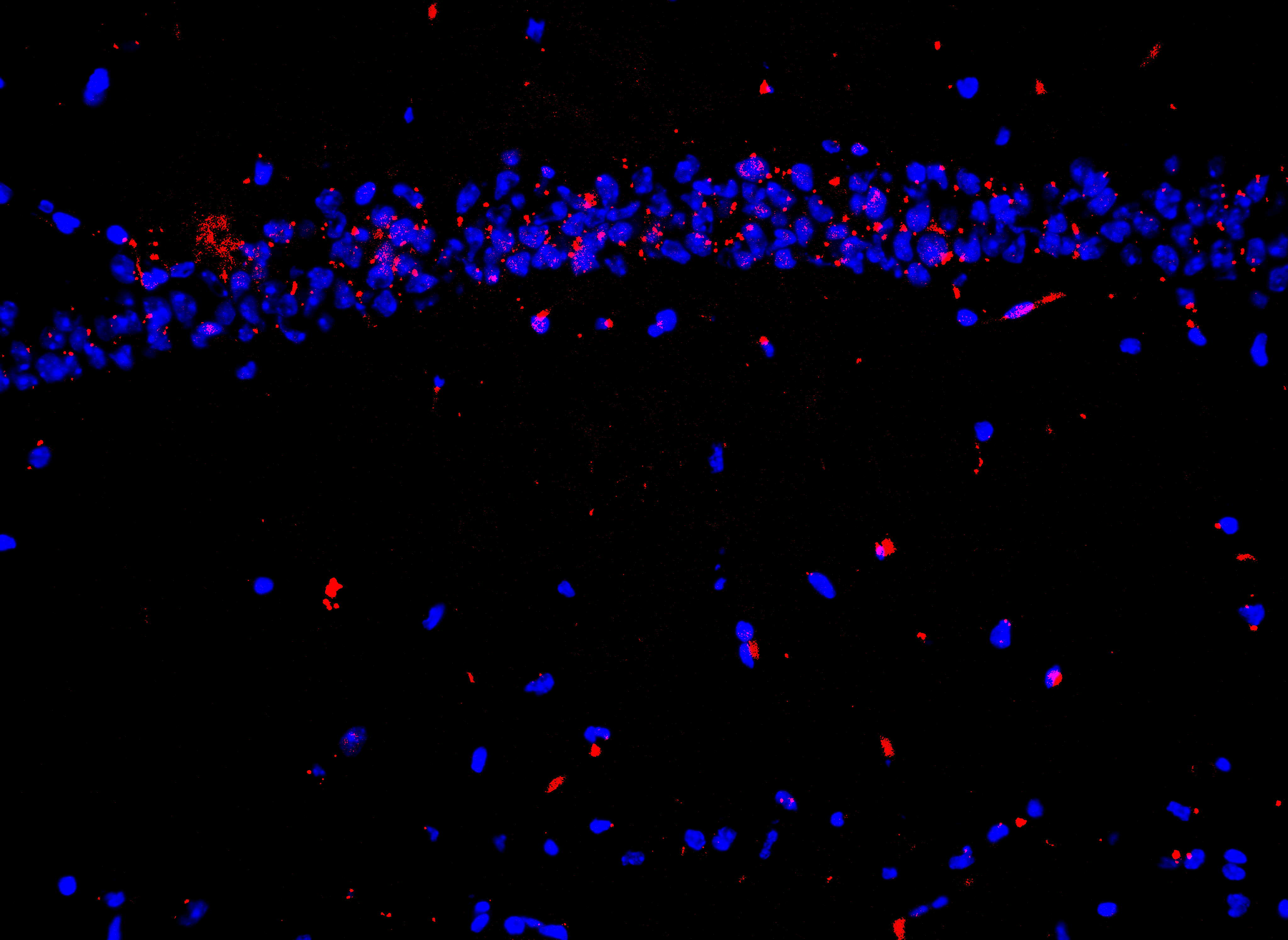

Supplement: Supplementary file 8 — Source data Fig. 6 [file 44321_2026_422_MOESM8_ESM.zip › Figure 6/Figure 6A/dbdb+K223R-pe/Merge.tif]
