# Supplementary material for: Hyperglycemia impairs cognitive function by inducing mitochondrial damage through lactylation of LRPPRC at K223
Source: EMBO Mol Med. 2026 Apr 6;18(6):2038–61. doi: 10.1038/s44321-026-00422-8 (PMC13269535; doi:10.1038/s44321-026-00422-8)
Supplement: Supplementary file 2 — Peer Review File [file 44321_2026_422_MOESM2_ESM.pdf]

EMBO Molecular Medicine

Hyperglycemia impairs cognitive function by causing mitochondrial damage via LRPPRC K223 lactylation

Jingxi Xu, Yuqi Hao, Jingxue Cao, Xing Yang, Yun Gao, Xiaodong Sun, Rongrong Nie, Qiongsui Zhong, Yuanmei Zhong, Junjia Zhong, and Tianpeng Zheng

Corresponding author: Tianpeng Zheng (ZhengTP@glmc.edu.cn)

Review Timeline:

Submission Date:	19th Aug 25
Editorial Decision:	16th Sep 25
Revision Received:	31st Jan 26
Editorial Decision:	28th Feb 26
Revision Received:	19th Mar 26
Accepted:	20th Mar 26

Editor: Jingyi Hou

Transaction Report:

(Note: With the exception of the correction of typographical or spelling errors that could be a source of ambiguity, letters and reports are not edited. Depending on transfer agreements, referee reports obtained elsewhere may or may not be included in this compilation. Referee reports are anonymous unless the Referee chooses to sign their reports.)

16th Sep 2025

Dear Prof. Zheng,

Thank you again for submitting your work to EMBO Molecular Medicine. We have now heard back from the three referees who agreed to evaluate your manuscript. As you will see below, the referees raise significant concerns about your work, which unfortunately preclude its publication in EMBO Molecular Medicine.

While the referees found the findings to be novel and interesting, they raised substantial concerns about the manuscript. Specifically, they noted the absence of several key experiments related to mitochondrial function and brain section analyses, highlighted issues with the animal behavioral data, as well as the adequacy of the mouse models used and the validity of the resulting conclusions. Additionally, both Referees #1 and #3 raised concerns about the interpretation of post-translational modifications at K223, specifically regarding potential ubiquitination and the lack of direct evidence for lactylation.

During the pre-decision cross-commenting process (where referees are invited to respond to each other's reports), both Referees #1 and #3 thought that the current data are insufficient and indicated that the manuscript goes beyond what could reasonably be addressed in a major revision.

In light of these comments and the overall referee feedback, I regret to inform you that we are unable to consider the manuscript further at this stage.

However, given the reviewers did have positive comments about the clinical relevance and novelty of the study, we would be open to considering a substantially revised and extended version of the manuscript, provided that all reviewer concerns are convincingly addressed. We understand that, given the scope of the revisions required, you may prefer to submit your work elsewhere.

Should you choose to resubmit to us, please note that the manuscript would receive a new number and receipt date. It will be editorially evaluated afresh, and its novelty will be re-assessed at the time of submission. As you probably understand, we can give no guarantee about its eventual acceptability. If you do decide to follow this course, then we would ask you to enclose with your re-submission a point-by-point response to the points raised in the present review.

I am sorry that the review of your work did not result in a more favorable outcome on this occasion, but I hope that you will not be discouraged from sending your work to EMBO Molecular Medicine in the future. In any case, thank you for the opportunity to examine this work.

Sincerely,
Jingyi

Jingyi Hou
Senior Editor
EMBO Molecular Medicine

***** Reviewer's comments *****

Referee #1 (Remarks for Author):

In this study, the authors and their colleagues elucidate a novel mechanism whereby high glucose induces mitochondrial dysfunction and cognitive impairment through lactylation of LRPPRC at K223. They further develop a targeted intervention peptide that competitively inhibits LRPPRC-K223 lactylation, significantly ameliorating cognitive dysfunction in diabetic mice. The study design is comprehensive and the data are well documented, giving the work both scientific value and translational potential. However, the methodologies employed and the results presented are insufficient to fully support the conclusions, and the description of several experimental findings lacks clarity, raising serious concerns. These issues are reflected in the following points:

Major concerns:

On page 8, lines 8-11 (Figure 5C), the authors investigated the therapeutic potential of K223-pe in diabetic mice. They report

that K223-pe, but not K223R-pe, significantly reversed high glucose-induced lactylation of LRPPRC at K223 in hippocampal CA1 neurons from both type 1 and type 2 diabetic mice, thereby restoring the LRPPRC-SLIRP interaction. This result appears contradictory to their previous findings. Earlier, the authors demonstrated that exogenous AAV-mediated expression of LRPPRC-K223R markedly enhanced LRPPRC-SLIRP interaction, ameliorating neuronal apoptosis and cognitive impairment. However, when K223-pe was administered to LRPPRC-K223R mice, the treatment competitively inhibited lactylation at the K223 site in vivo. This dual inhibition of LRPPRC-K223 lactylation not only failed to show therapeutic benefit in diabetic mice but also appeared to further suppress LRPPRC-SLIRP binding (Figure 5C), seemingly exacerbating pathology compared with vehicle treatment. This inconsistency raises serious concerns regarding the accuracy and interpretation of the results.

In the animal experiments, the authors employed AAV-LRPPRC-WT viral injections and used anti-LRPPRC antibody (Proteintech, Cat# 21175-1-AP) together with anti-Pan Lactic Acid-Lysine antibody (ABclonal, Cat# A23004). These approaches can only demonstrate the overall lactylation level of LRPPRC, not specifically that of K223. Even with the AAV-LRPPRC-K223R variant, the results merely suggest suppression of lactylation at K223. Throughout the manuscript, however, the authors consistently describe changes in the "lactylation level of LRPPRC-K223." Unless a custom site-specific antibody targeting LRPPRC-K223 was used, these claims are not substantiated. To more rigorously validate the findings, the authors might also consider generating and testing LRPPRC-K223T (a lactylation-mimic variant) as an additional control.

The authors used exogenous AAV-LRPPRC-WT/K223R injections to study effects on neuronal apoptosis and cognitive impairment in diabetic mice. Given the elevated endogenous lactylation of LRPPRC in diabetic mice, the potential confounding influence of endogenous LRPPRC on SLIRP interaction cannot be excluded. To eliminate this interference, exogenous labeling (e.g., with tagged constructs) combined with antibody-based immunoprecipitation should be incorporated to strengthen the validity of the conclusions.

Neuronal apoptosis could be more clearly demonstrated by TUNEL staining. Moreover, swimming speed is an important confounding factor in the Morris water maze test, as it can affect learning and memory performance. Therefore, Figures 2C-D, 4G, and 6B should include the swimming speed of mice. Additionally, the small sample size in behavioral experiments resulted in large intra-group variability and scattered data. Increasing the number of animals per group would improve the robustness and stability of the behavioral findings.

Minor concerns:

In Figure 3A, the authors mapped the LRPPRC-SLIRP binding sites. It would be logical to subsequently examine which binding site is functionally critical for the LRPPRC-SLIRP interaction and whether LRPPRC-K223R affects this binding. Unfortunately, this potentially informative question was not addressed in later experiments.

On page 5, lines 10-11 (Figure 3), the authors state: "LRPPRC K223 lactylation reduces its binding to SLIRP, weakens mitochondrial mRNA stability, and triggers mitochondrial dysfunction." These findings demonstrate that in diabetic mice (but not controls), LRPPRC-K223R promotes LRPPRC-SLIRP interaction, enhances mitochondrial mRNA stability, and ameliorates mitochondrial dysfunction in hippocampal neurons.

On page 5, line 22, the authors report that in hippocampal CA1 neurons from both type 1 and type 2 diabetic mice, lactylation of LRPPRC at K223 is elevated, while the LRPPRC-SLIRP interaction is reduced. However, the data only appear to demonstrate an overall increase in LRPPRC lactylation, rather than specifically at K223. The description should therefore be revised to ensure accuracy.

Please provide the statistical data plots for Figure S5A and indicate the sample size (n). Additionally, the manuscript states on page 6, lines 27-28 (Figure S5B): "thereby elevating LRPPRC K223 lactylation levels," and on page 7, lines 5-6 (Figure 4A): "resulting in elevated LRPPRC K223 lactylation." Since LRPPRC contains multiple lysine lactylation sites, and AARS2-mediated lactylation is unlikely to be specific for K223, the descriptions should instead refer to "overall lactylation of LRPPRC" rather than specifically to K223.

The pharmacokinetic properties of the short peptide, including half-life and blood-brain barrier penetration efficiency, have not been described and should be elaborated upon.

Referee #2 (Comments on Novelty/Model System for Author):

Detailed in remarks to authors.

Referee #2 (Remarks for Author):

In this manuscript, the authors demonstrate that high glucose-induced LRPPRC K223 lactylation impairs mitochondrial functions, leading to neuronal apoptosis and cognitive decline in diabetic model mice. The underlying mechanisms involve reduced LRPPR-SLIRP interaction and decreased mitochondrial mRNA stability. The authors further show that a short peptide targeting

LRPPRC K223 lactylation can reverse these defects. Additionally, they show that T2D patients have an elevated plasma LRPPRC K224 lactylation levels. While these findings are intriguing and clinically relevant, several critical issues need to be addressed:
1. The conclusion that LRPPRC K223 lactylation induces neuronal apoptosis relies heavily on flow cytometry data. Additional supportive evidence-such as TUNEL, NeuN staining in brain sections-would strengthen this claim.
2. It is unclear whether the authors generated a antibody against LRPPRC K223/k224 lactylation or not, if not, how they specifically measured the plasma LRPPRC K223/k224 lactylation levels in mice and human patients; if yes, the specificity of the antibody needs to be validate.
3. The authors show a significant decrease in mitochondrial RNA levels, but corresponding protein levels were not examined. Since proteins are the functional effectors of mitochondrial activity, assessing their expression is essential.
4. The source of elevated plasma LRPPRC K224 lactylation in T2D patients remains unclear. Is it derived from brain neurons or peripheral tissues?
5. Figure S10B, LRPPRC K224 lactylation is elevated in metformin users despite the authors' suggestion that lower lactylation is beneficial in T2D. This apparent contradiction requires clarification.
6. The sample size used for behavioral tests appears insufficient. Increasing the number of animals would improve the statistical robustness of the conclusions.
7. Molecular weight markers (kDa) are missing from the immunoblot figures in the current version of the manuscript. These should be clearly indicated for each blot.

Referee #3 (Comments on Novelty/Model System for Author):

There are concerns regarding the assessment of mitochondrial function, usage of the mouse model (hyperglycemia vs diabetes), mouse power calculations, and brain histology.

Referee #3 (Remarks for Author):

This is an interesting study presenting cell, mouse, and human data to show that lactylation of LRPPRC is linked to cognitive function in diabetes.

Major:
1. The analysis of mitochondrial function is very superfluous. It is imperative to use Seahorse analysis to verify an impact on mitochondrial function beyond ROS and MMP.
2. It will be important to include indirect calorimetry to supplement the behavioral data with energy metabolism data in the context of diabetes.
3. The db/db and STZ models are type 2 and type 1 models, but neither are accepted to be close to the human situation (beyond hyperglycemia). For that, DIO and NOD models for T2D and T1D should have been used. Please comment and perhaps change the major conclusions based on that.
4. K223 could also be ubiquitinylated. Please verify if there is a change in that as the usage of the mutant might interfere with this PTM and subsequent regulation.
5. I am not a brain expert but it will be important to use in situ hybridization or similar to show that it is hippocampal LRPPRC. I fail to see the proof in vivo in brains here. Same applies to the usage of the AAVs and their appropriate targeting to these regions, which should be demonstrated by imaging of histology.
6. Please elaborate on the power calculation of the mouse experiments and if they were independently reproduced. The mouse methods part also lacks more information on breeding, housing, weaning. Altogether, it is imperative to revise all methods part to be much more detailed.

Minor:
7. Please include all uncropped Western blots with legends indicating what is what.
8. The legends in the figures are suboptimal and make it difficult to follow the stream of thought and understand the data.

As a service to authors, EMBO provides authors with the possibility to transfer a manuscript that one journal cannot offer to publish to another EMBO publication. The full manuscript and if applicable, reviewers reports are automatically sent to the receiving journal to allow for fast handling and a prompt decision on your manuscript. For more details of this service, and to transfer your manuscript to another EMBO title please click on Link Not Available

Please do not share this URL as it will give anyone who clicks it access to your account.

Referee #1:

In this study, the authors and their colleagues elucidate a novel mechanism whereby high glucose induces mitochondrial dysfunction and cognitive impairment through lactylation of LRPPRC at K223. They further develop a targeted intervention peptide that competitively inhibits LRPPRC-K223 lactylation, significantly ameliorating cognitive dysfunction in diabetic mice. The study design is comprehensive and the data are well documented, giving the work both scientific value and translational potential. However, the methodologies employed and the results presented are insufficient to fully support the conclusions, and the description of several experimental findings lacks clarity, raising serious concerns. These issues are reflected in the following points:

Response: We sincerely thank our reviewer for the outstanding and visionary review of our manuscript. Your expert comments reflect exceptional professional rigor and have substantially improved the quality of our work. Below, a detailed point-by-point response to each comment is provided. All revisions in the manuscript are highlighted in yellow. Please note that some figures have reduced resolution in the PDF format; the original high-resolution versions are available in the submission system.

Major concerns:

(1) On page 8, lines 8-11 (Figure 5C), the authors investigated the therapeutic potential of K223-pe in diabetic mice. They report that K223-pe, but not K223R-pe, significantly reversed high glucose-induced lactylation of LRPPRC at K223 in hippocampal CA1 neurons from both type 1 and type 2 diabetic mice, thereby restoring the LRPPRC-SLIRP interaction. This result appears contradictory to their previous findings. Earlier, the authors demonstrated that exogenous AAV-mediated expression of LRPPRC-K223R markedly enhanced LRPPRC-SLIRP interaction, ameliorating neuronal apoptosis and cognitive impairment. However, when K223-pe was administered to LRPPRC-K223R mice, the treatment competitively inhibited lactylation at the K223 site in vivo. This dual inhibition of LRPPRC-K223 lactylation not only failed to show therapeutic benefit in diabetic mice but also appeared to further suppress LRPPRC-SLIRP binding (Figure 5C), seemingly exacerbating pathology compared with vehicle treatment. This inconsistency raises serious concerns regarding the accuracy and interpretation of the results.

Response: We sincerely appreciate the reviewer's careful reading and apologize for the confusion caused by unclear notation in our figure legend.

1) In our previous experiments (Figure 2, Figure 3 and Figure S3), AAV-mediated expression of full-length LRPPRC-K223R was employed. The K223R mutation prevents lactylation at this site, thereby preserving LRPPRC-SLIRP interaction, ameliorating neuronal apoptosis and cognitive impairment.

2) Differently, the experiments in Figure 5 employed short synthetic peptides (K223-pe and K223R-pe), not full-length LRPPRC sequences. These short peptides cannot bind SLIRP or recapitulate full protein function. Instead, K223-pe functions as a competitive "decoy peptide" that preferentially captures lactylation modifications, thereby protecting endogenous LRPPRC from lactylation at K223. By reducing lactylation of endogenous LRPPRC, K223-pe treatment enhances LRPPRC-SLIRP interaction and improves cognitive function. This peptide-based strategy has been successfully employed with similar therapeutic rationale and efficacy in previous studies (*Cell, PMID: 38128537; PNAS, PMID: 36943877; eBioMedicine, PMID: 35339896*).

3) We would like to respectfully clarify that all experimental groups (db/db, db/m, STZ, and Ctr mice) in Figure 5 received direct injections of either K223-pe or K223R-pe peptide (scrambled peptide). None of these animals had genetic mutations at the K223 site. The figure legend has been revised to clearly describe the peptide treatment protocol.

(2) In the animal experiments, the authors employed AAV-LRPPRC-WT viral injections and used anti-LRPPRC antibody (Proteintech, Cat# 21175-1-AP) together with anti-Pan Lactic Acid-Lysine antibody (ABclonal, Cat# A23004). These approaches can only demonstrate the overall lactylation level of LRPPRC, not specifically that of K223. Even with the AAV-LRPPRC-K223R variant, the results merely suggest suppression of lactylation at K223. Throughout the manuscript, however, the authors consistently describe changes in the "lactylation level of LRPPRC-K223." Unless a custom site-specific antibody targeting LRPPRC-K223 was used, these claims are not substantiated. To more rigorously validate the findings, the authors might also consider generating and testing LRPPRC-K223T (a lactylation-mimic variant) as an additional control.

Response: We appreciate the reviewer for this constructive suggestion. We have addressed this concern through three approaches:

(1) Generation and validation of site-specific antibody:

A site-specific antibody targeting lactylated LRPPRC at K223 has been generated. The specificity of this antibody was validated using dot blot analysis, a well-established method for antibody specificity

verification (*Science Advances, PMID: 37267363; Theranostics, PMID: 39113793; Journal of Experimental & Clinical Cancer Research, PMID: 40849487*).

Methods Section, page 24, lines 14-21:

To prepare a site-specific antibody targeting mouse LRPPRC lactylated at K223, a synthetic polypeptide containing a lactyl group on lysine 223 (ILGFMK(lac)TKDLP) was utilized. Polyclonal antibodies were generated by immunizing New Zealand White rabbits with 0.5 mL of peptide (1 mg/mL) mixed with an equal volume of complete adjuvant, followed by three weekly booster injections, each containing 0.5 ml of the polypeptide (1 mg/ml) plus incomplete adjuvant. Antiserum was harvested via carotid artery seven days post final immunization and affinity-purified using a Protein A/G column. The human LRPPRC K224 is homologous to mouse LRPPRC K223, and the antibody prepared by the above method was also used to detect human LRPPRC lactylated at K224.

Results Section, page 5, lines 20-22:

To investigate whether high glucose induces elevated LRPPRC K223 lactylation in vivo, we employed a specific LRPPRC K223la antibody (Figure S1B) to detect LRPPRC K223 lactylation levels in the hippocampal CA1 region of diabetic mice.

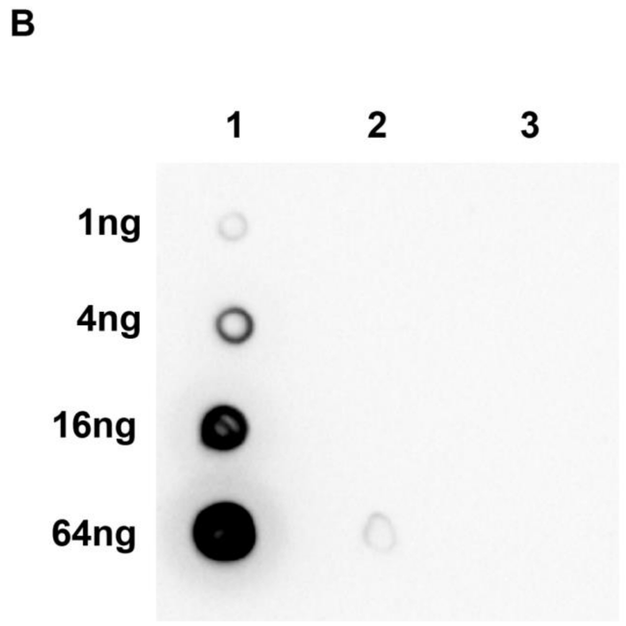

Figure S1 (B): Verification of the specificity of the LRPPRC K223la antibody by dot blots experiment. 1: modified peptide 1 (ILGFMK(lac)TKDLP); 2: modified peptide 2 (ILGFMKTK(lac)DLP); and 3: non-modified peptide (ILGFMKTKDLP).

(2) Replacement of overall lactylation level of LRPPRC with LRPPRC K223 site-specific lactylation

To provide more precise mechanistic insights, we have replaced the detection of overall lactylation level of LRPPRC with LRPPRC K223 site-specific lactylation detection throughout the manuscript. The relevant data are now presented in Figures 3B, 4A, 5C, S3E, S3F, S6C, S6H, S6K, and S10A.

(3) Generation and testing of lactylation-mimic mutant

The LRPPRC-K223T mutant has been generated and tested as a lactylation-mimic control.

Results Section, page 8, lines 10-15:

Similarly, in vitro experiments demonstrated that high glucose (HG) treatment significantly reduced the binding between LRPPRC and SLIRP compared to the normal glucose (NG) group (Figure S3F-H). Under high glucose conditions, the K223R group showed restored binding between LRPPRC and SLIRP compared with the WT group, by contrast, the K223T group exhibited even lower binding between LRPPRC and SLIRP than the WT group (Figure S3F-H). These results confirm that LRPPRC K223 lactylation inhibits the binding of LRPPRC and SLIRP.

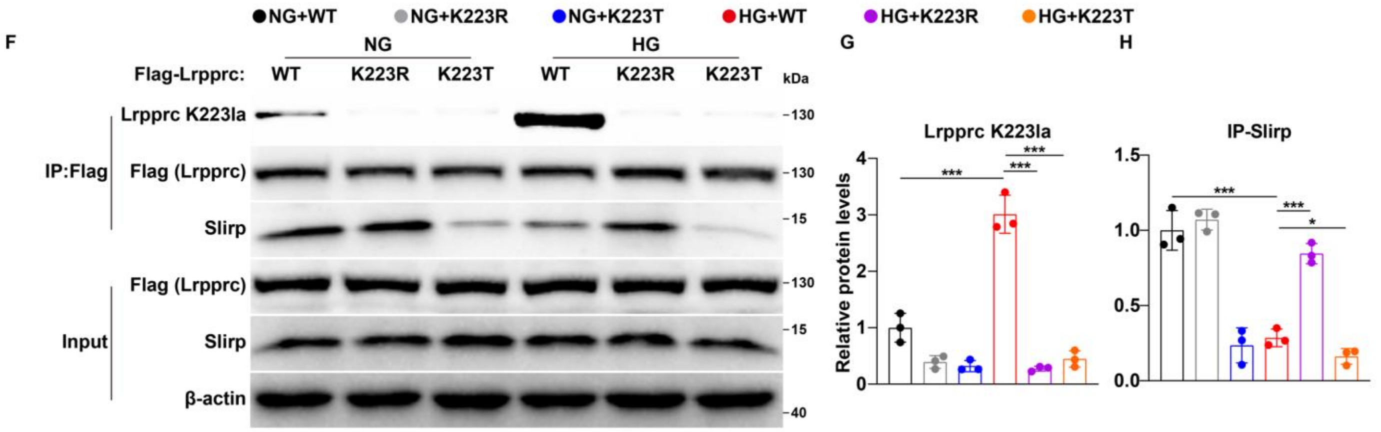

Figure S3 (F-H): Primary hippocampal neurons, which were stably silenced endogenous LRPPRC and expressed Flag-tagged WT LRPPRC, K223R LRPPRC or K223T LRPPRC, were treated with normal glucose (NG, 5.5 mmol/L D-glucose) or high glucose (HG, 25 mmol/L D-glucose). Cell lysates were immunoprecipitated with anti-Flag antibodies and western blotted with the indicated antibodies (F). Quantification analysis of Lrpprc K223la (G) and IP-Slirp (H) levels in the indicated groups (n = 3 biological replicates).

Results Section, page 9, lines 13-16:

Additionally, in vitro experimental results demonstrated that under high glucose conditions, the K223T group showed even lower mitochondrial mRNA stability (Figure S4O-R and W-Z) and more severe mitochondrial dysfunction compared with the WT group (as indicated by elevated mitochondrial ROS levels, decreased mitochondrial membrane potential and OCR) (Figure S5E-J).

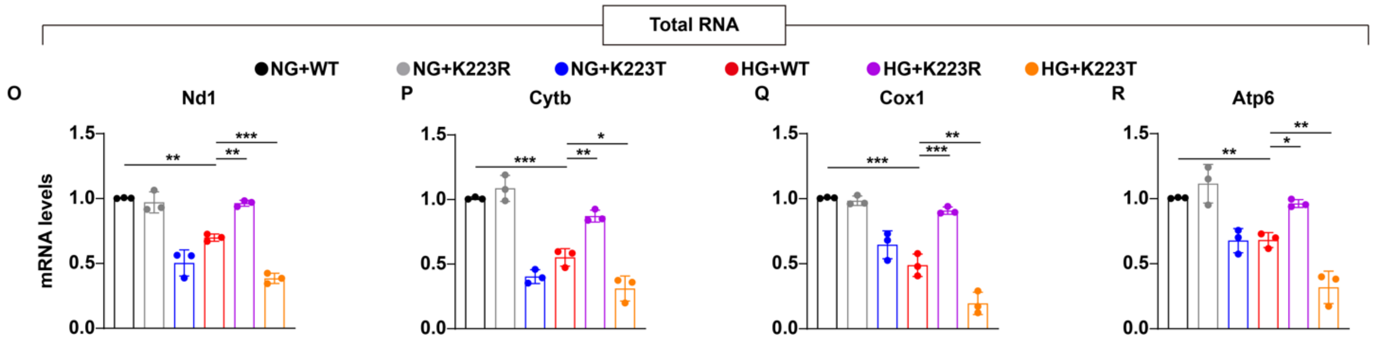

Figure S4 (O-R): Primary hippocampal neurons, which were stably silenced endogenous LRPPRC and expressed WT LRPPRC, K223R LRPPRC or K223T LRPPRC, were treated with normal glucose (NG, 5.5 mmol/L D-glucose) or high glucose (HG, 25 mmol/L D-glucose). Total RNA was isolated, and the levels of each mtRNA species were determined by PCR (n = 3 biological replicates).

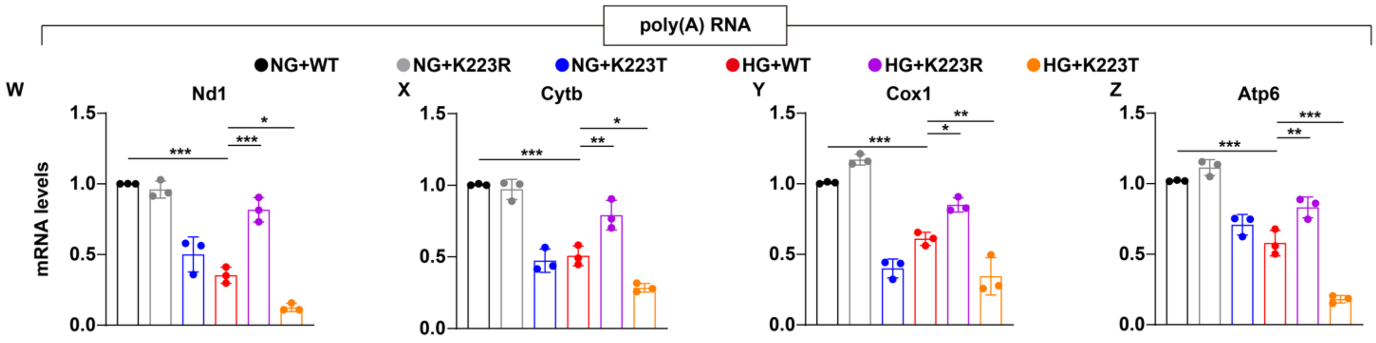

Figure S4 (W-Z): Primary hippocampal neurons, which were stably silenced endogenous LRPPRC and expressed WT LRPPRC, K223R LRPPRC or K223T LRPPRC, were treated with normal glucose (NG, 5.5 mmol/L D-glucose) or high glucose (HG, 25 mmol/L D-glucose). Poly(A)-tailed RNA was isolated, and the levels of each mtRNA species were determined by PCR (n = 3 biological replicates).

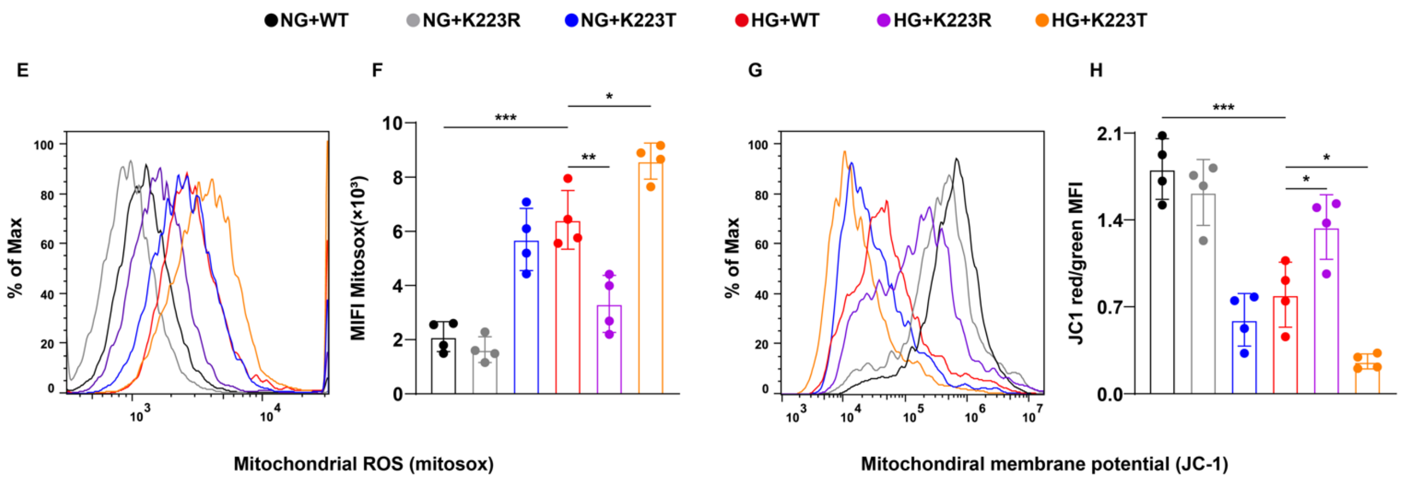

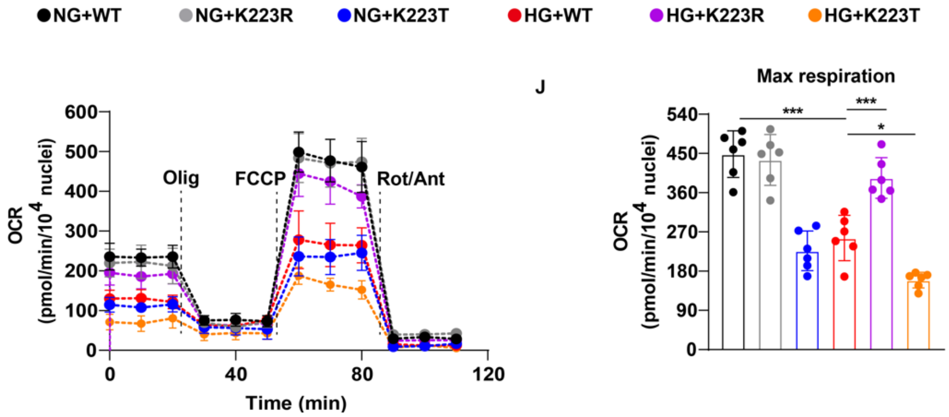

Figure S5 (E-F): Flow cytometry (E) and quantification analysis (F) of mtROS levels in primary hippocampal neurons with endogenous LRPPRC stably silenced and expressing either wild-type (WT) LRPPRC, K223R LRPPRC or K223T LRPPRC, treated with normal glucose (NG, 5.5 mmol/L D-glucose) or high glucose (HG, 25 mmol/L D-glucose) (n = 4 biological replicates).

Figure S5 (G-H): Flow cytometry (G) and quantification analysis (H) of MMP levels in primary hippocampal neurons with endogenous LRPPRC stably silenced and expressing either wild-type (WT) LRPPRC, K223R LRPPRC or K223T LRPPRC, treated with normal glucose (NG, 5.5 mmol/L D-glucose) or high glucose (HG, 25 mmol/L D-glucose) (n = 4 biological replicates).

Figure S5 (I-J): OCR measurements of primary hippocampal neurons with endogenous LRPPRC stably silenced and expressing either wild-type (WT) LRPPRC, K223R LRPPRC or K223T LRPPRC, treated with normal glucose (NG, 5.5 mmol/L D-glucose) or high glucose (HG, 25 mmol/L D-glucose) under basal conditions and in response to oligomycin (Olig), *p*-trifluoromethoxy carbonyl cyanide phenylhydrazone

(FCCP), antimycin A/rotenone (Rot/Ant) (I); the quantification of the maximal OCR (J) (n= 6 biological replicates).

Results Section, page 6, lines 12-17:

As illustrated in Figure S2C-F, high glucose (HG) treatment significantly increased apoptosis in primary hippocampal neurons compared to the normal glucose (NG) group. Under high glucose conditions, the K223R group showed markedly reduced apoptosis compared with the WT group, by contrast, the K223T group exhibited even higher apoptosis levels than the WT group, demonstrating that high glucose-induced LRPPRC K223 lactylation triggers hippocampal neuronal apoptosis.

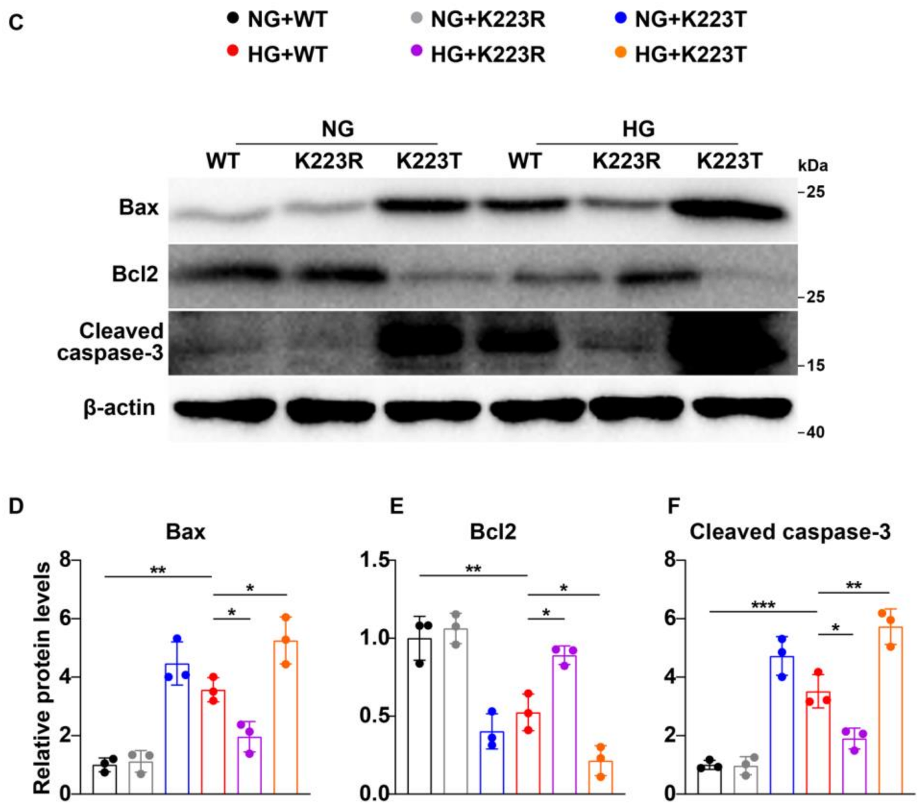

Figure S2 (C-F): Immunoblots (C) and quantification (D-F) analysis of Bax, Bcl-2 and Cleaved-caspase3 expression levels in the primary hippocampal neurons with endogenous LRPPRC stably silenced and expressing either wild-type (WT) LRPPRC, K223R LRPPRC or K223T LRPPRC, treated with normal glucose (NG, 5.5 mmol/L D-glucose) or high glucose (HG, 25 mmol/L D-glucose) (n = 3 biological replicates).

(3) The authors used exogenous AAV-LRPPRC-WT/K223R injections to study effects on neuronal apoptosis and cognitive impairment in diabetic mice. Given the elevated endogenous lactylation of LRPPRC in diabetic mice, the potential confounding influence of endogenous LRPPRC on SLIRP interaction cannot be excluded. To eliminate this interference, exogenous labeling (e.g., with tagged constructs) combined with antibody-based immunoprecipitation should be incorporated to strengthen the validity of the conclusions.

Response: We thank the reviewer for this insightful suggestion. We would like to politely clarify that, after consulting with the AAV manufacturer, it was confirmed that the AAV constructs used in our original experiments (AAV-LRPPRC-WT and AAV-LRPPRC-K223R) were already Flag-tagged. However, in our initial analysis, we performed immunoprecipitation using anti-LRPPRC antibody rather than anti-Flag antibody. In the revised manuscript, we have now performed anti-Flag antibody-based immunoprecipitation in db/db, db/m, STZ, and Ctr mice injected with AAV-Flag-LRPPRC-WT or AAV-Flag-LRPPRC-K223R. Using this approach, the exogenous Flag-tagged LRPPRC was specifically pulled down, and its interaction with SLIRP was examined independently of endogenous LRPPRC.

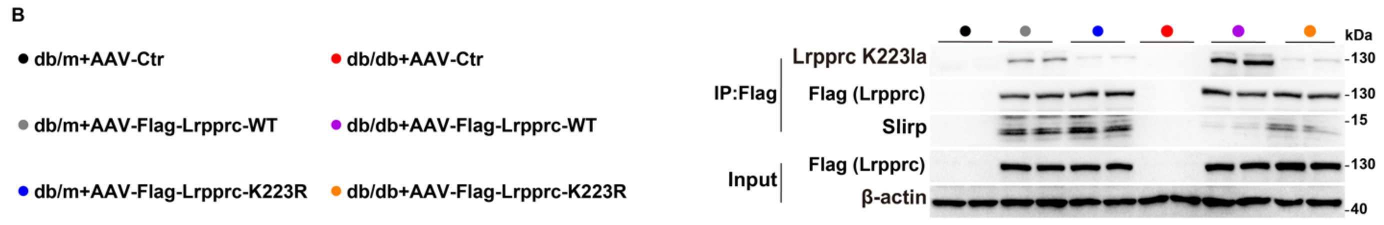

Figure 3 (B): Hippocampal CA1 neurons were isolated from db/m, db/db mice received stereotactic injections of AAV-Ctr, AAV-Flag-LRPPRC-WT or AAV-Flag-LRPPRC-K223R to specifically express either wild-type LRPPRC or the K223R mutant LRPPRC, and cell lysates were immunoprecipitated with anti-Flag antibodies and western blotted with the indicated antibodies (n = 6 mice per group).

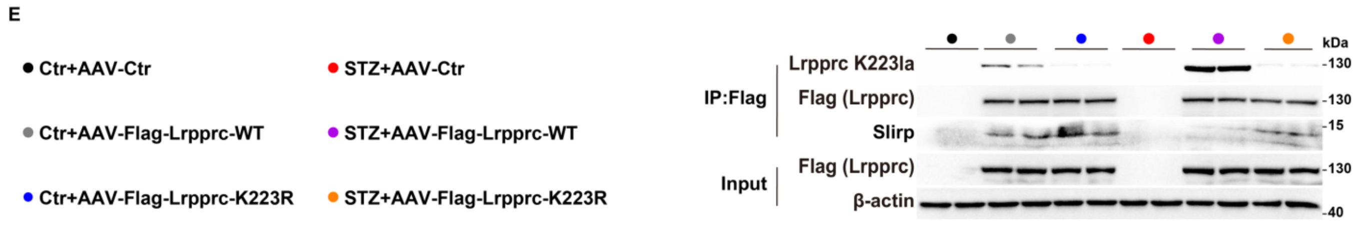

Figure S3 (E): Hippocampal CA1 neurons were isolated from Ctr or STZ mice received stereotactic injections of AAV-Ctr, AAV-Flag-LRPPRC-WT or AAV-Flag-LRPPRC-K223R to specifically express either

wild-type LRPPRC or the K223R mutant LRPPRC, and cell lysates were immunoprecipitated with anti-Flag antibodies and western blotted with the indicated antibodies (n = 6 mice per group).

(4) Neuronal apoptosis could be more clearly demonstrated by TUNEL staining. Moreover, swimming speed is an important confounding factor in the Morris water maze test, as it can affect learning and memory performance. Therefore, Figures 2C-D, 4G, and 6B should include the swimming speed of mice. Additionally, the small sample size in behavioral experiments resulted in large intra-group variability and scattered data. Increasing the number of animals per group would improve the robustness and stability of the behavioral findings.

Response: We thank the reviewer for these valuable suggestions to improve the rigor of our study.

1) Neuronal apoptosis: Following this suggestion, TUNEL staining has been performed to clearly demonstrate neuronal apoptosis in vivo.

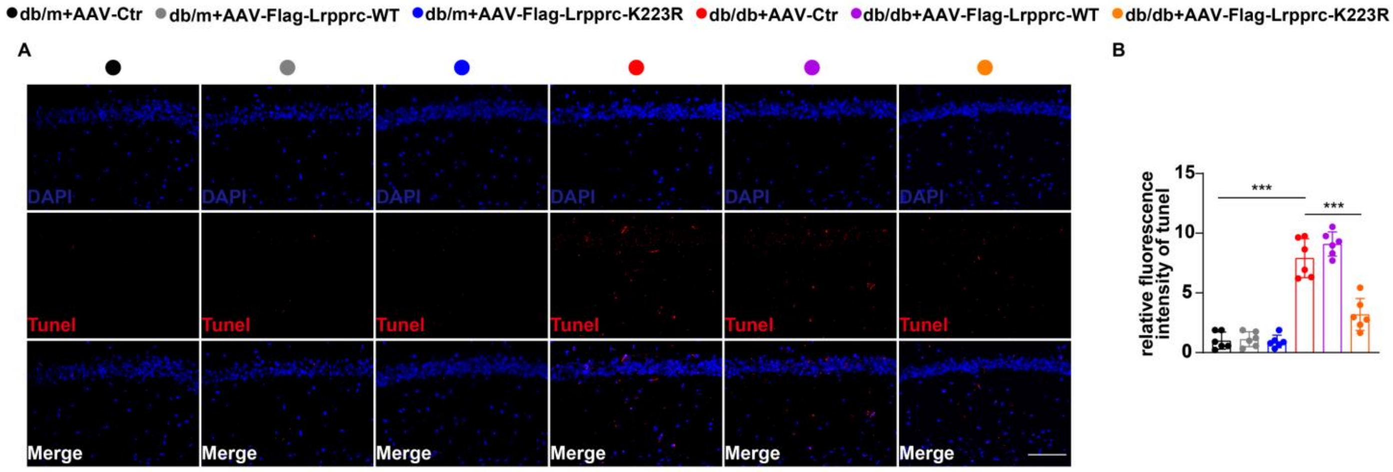

Figure 2 (A-B): Representative images (A) and quantifications (B) showing IF staining of Tunel (red) in the hippocampal CA1 neurons of db/m or db/db mice treated with AAV-Ctr, AAV-Flag-LRPPRC-WT or AAV-Flag-LRPPRC-K223R (n = 6 mice per group). Scale bar: 100 μm.

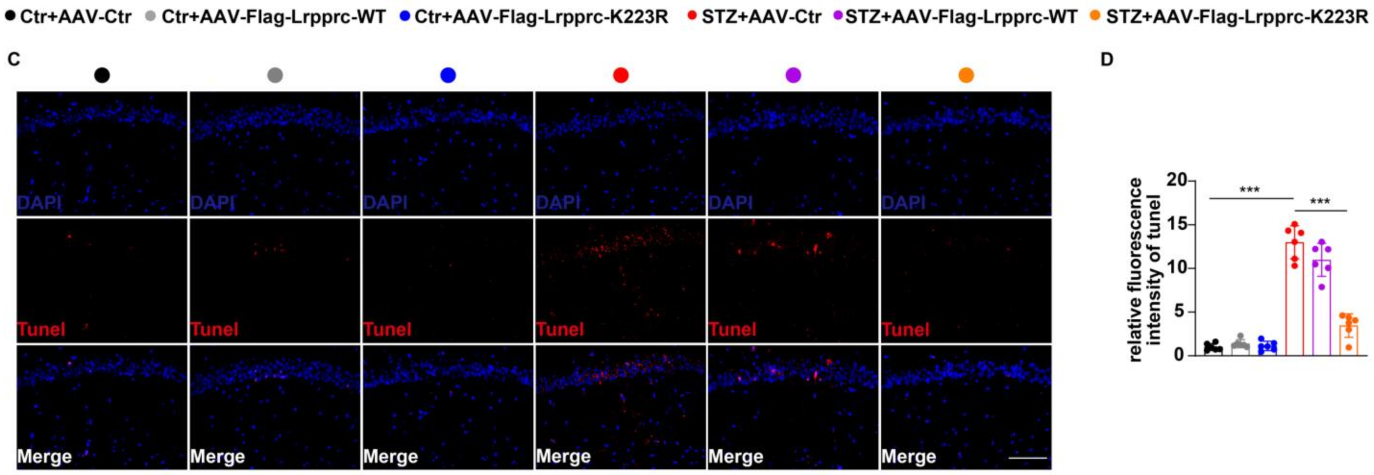

Figure 2 (C-D): Representative images (C) and quantifications (D) showing IF staining of Tunel (red) in the hippocampal CA1 neurons of Ctr or STZ mice treated with AAV-Ctr, AAV-Flag-LRPPRC-WT or AAV-Flag-LRPPRC-K223R (n = 6 mice per group). Scale bar: 100 μm.

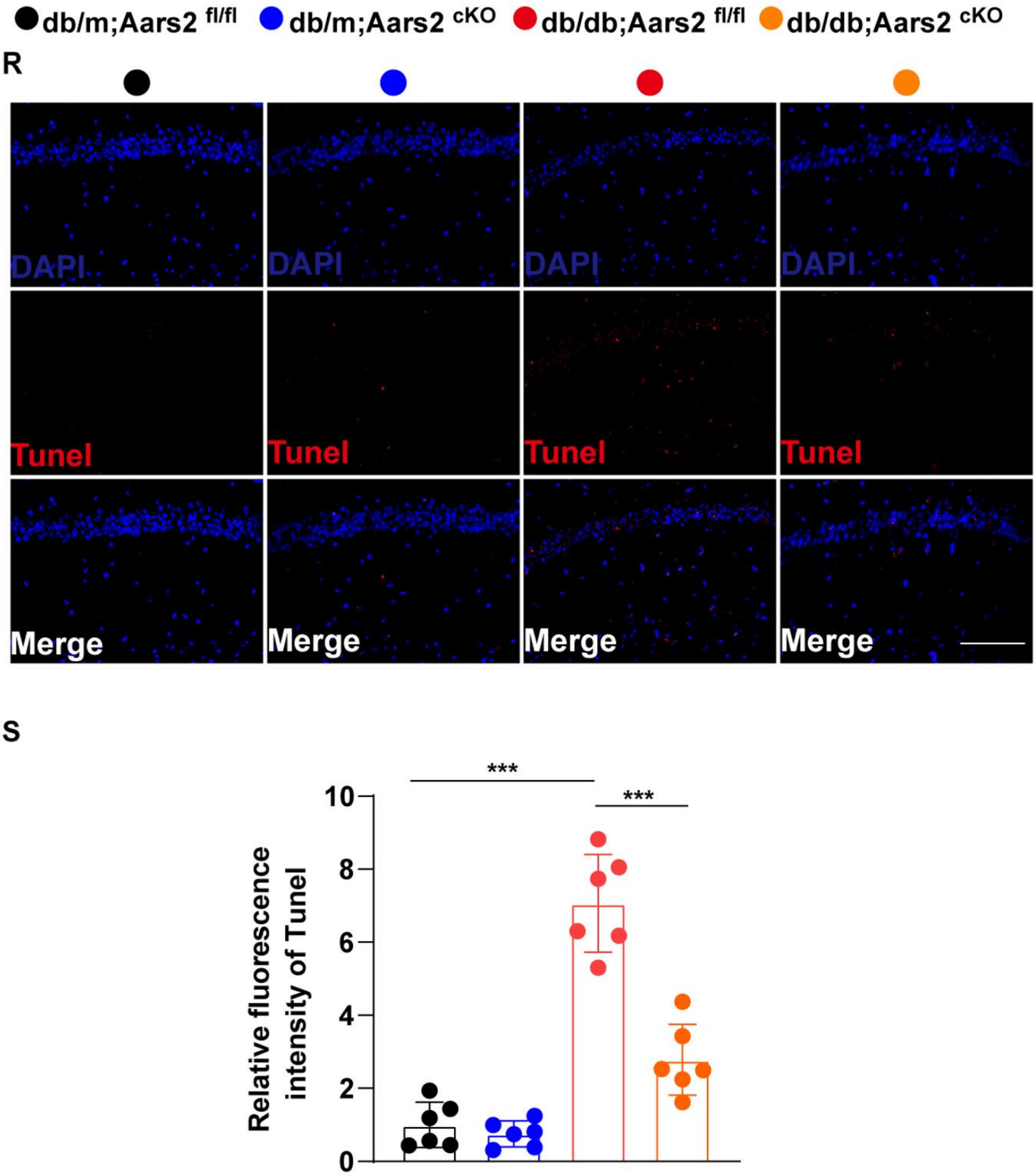

Figure 4 (R-S): Representative images (R) and quantifications (S) showing IF staining of Tunel (red) in the hippocampal CA1 neurons of db/m Aars2 $^{fl/fl}$, db/db Aars2 $^{fl/fl}$ mice injected with AAV-CAMKII or AAV-CAMKII-Cre (n = 6 mice per group). Scale bar: 100 μm.

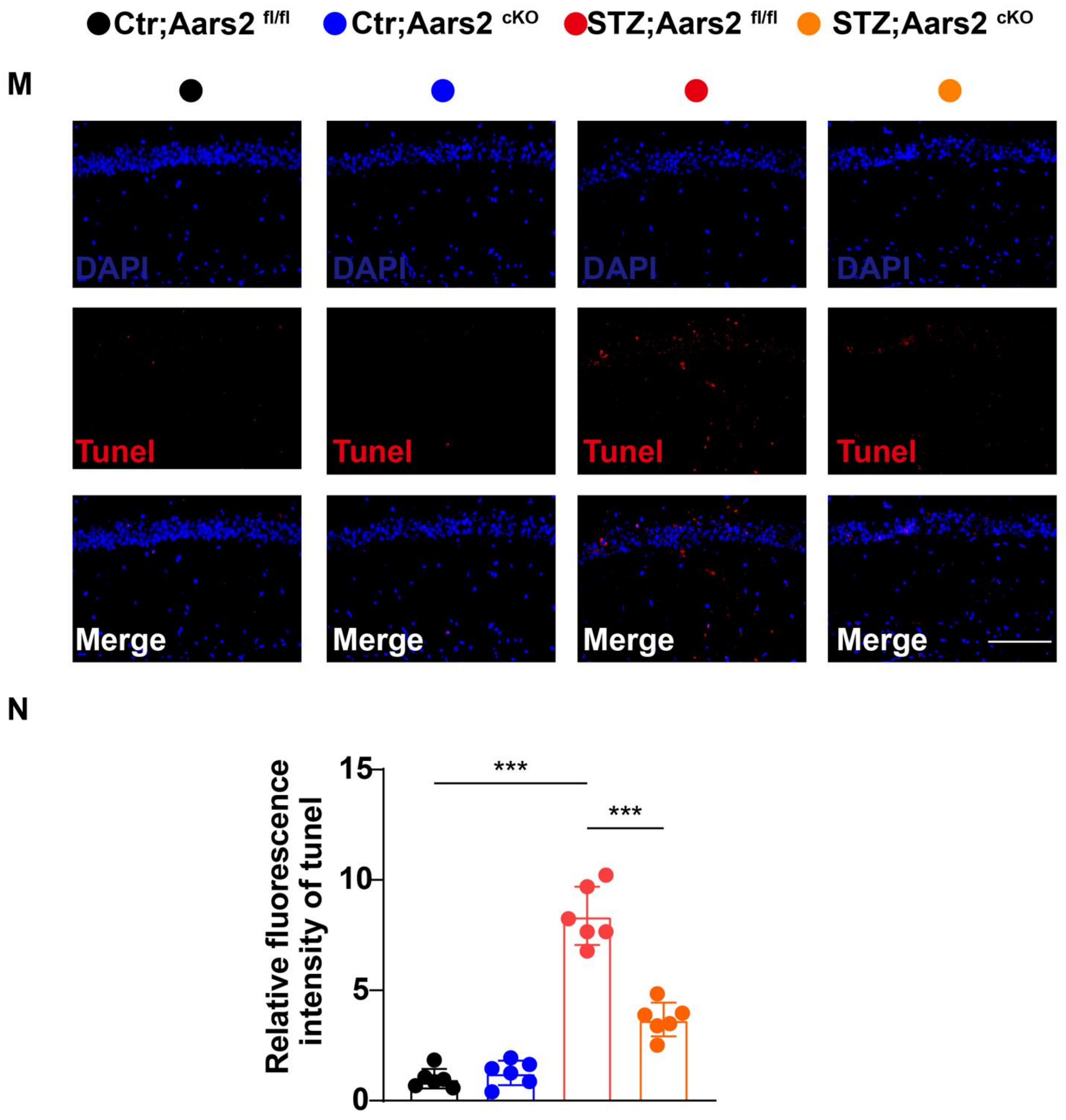

Figure S7 (M-N): Representative images (M) and quantifications (N) showing IF staining of Tunel (red) in the hippocampal CA1 neurons of Ctr Aars2 $^{fl/fl}$, STZ Aars2 $^{fl/fl}$ mice injected with AAV-CAMKII or AAV-CAMKII-Cre (n = 6 mice per group). Scale bar: 100 μm.

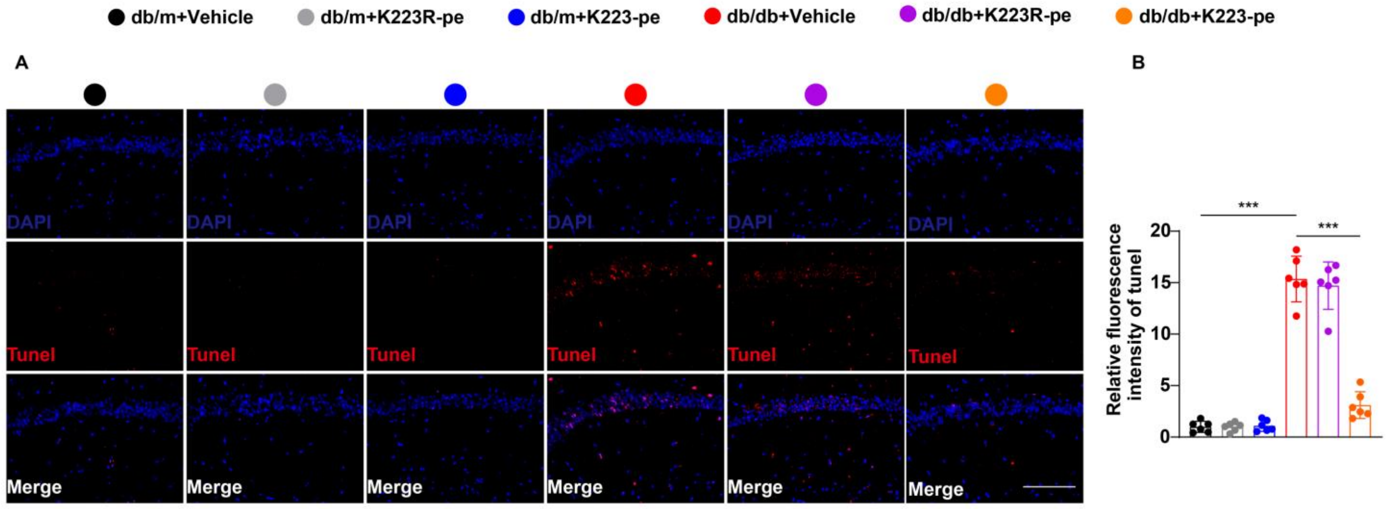

Figure 6 (A-B): Representative images (A) and quantifications (B) showing IF staining of Tunel (red) in the hippocampal CA1 neurons of db/m, db/db mice treated with vehicle, K223R-pe (scrambled peptide, 5 mg/kg) or K223-pe (5 mg/kg) (n = 6 mice per group). Scale bar: 100 µm.

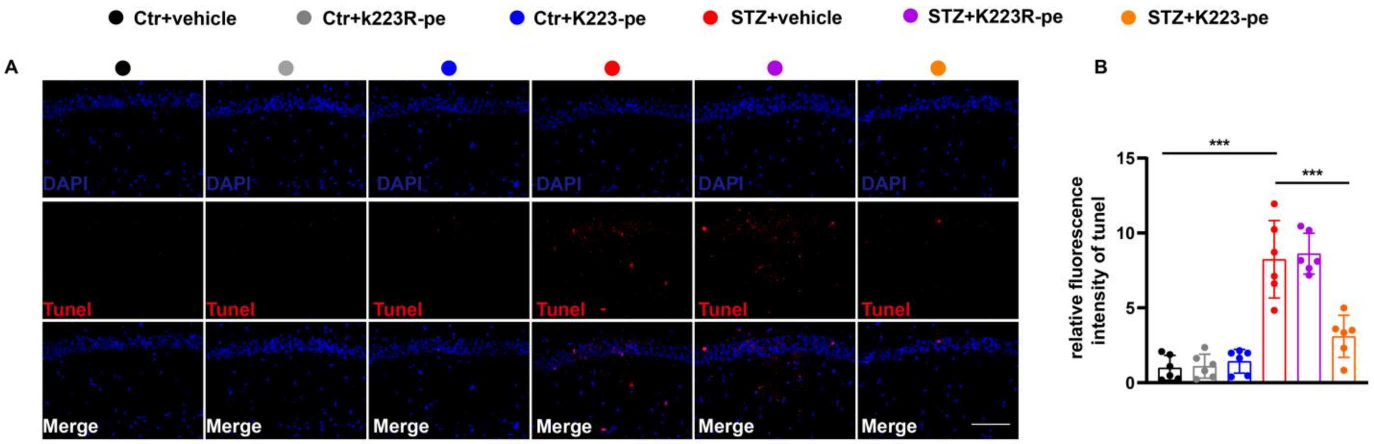

Figure S11 (A-B): Representative images (A) and quantifications (B) showing IF staining of Tunel (red) in the hippocampal CA1 neurons of Ctr, STZ mice treated with vehicle, K223R-pe (scrambled peptide, 5 mg/kg) or K223-pe (5 mg/kg) (n = 6 mice per group). Scale bar: 100 µm.

2) Swimming speed: Swimming speed data have now been included in relevant figures: Figure 2C-D (now Figure 2E-N), Figure 4G (now Figure 4T-X), and Figure 6B (now Figure 6C-G).

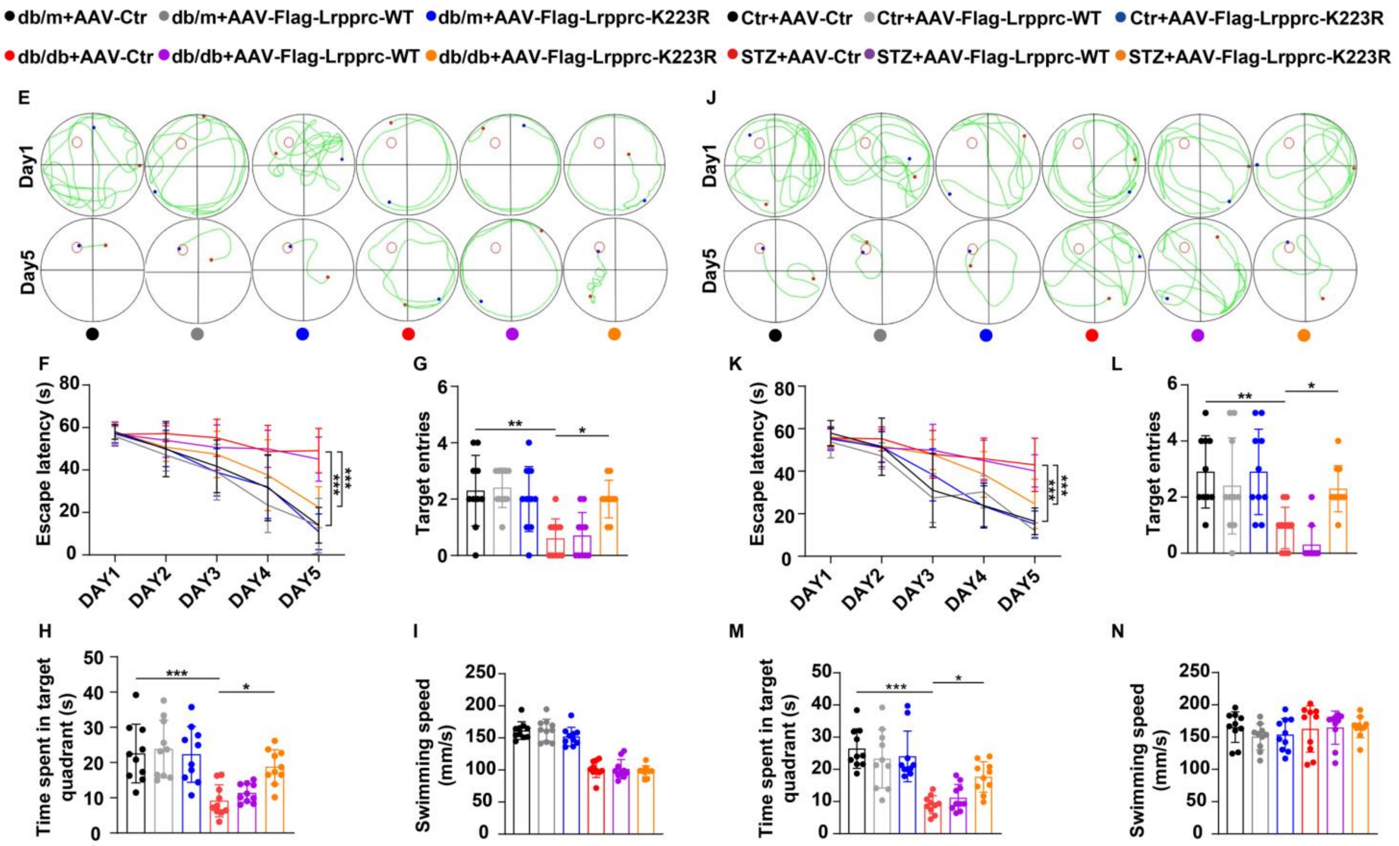

Figure 2 (E-I): Representative track images (E), escape latency to the platform (F) and swimming speed (I) during the training trials, target entries (G) and time spent in target quadrant (H) in the probe trial of Morris water maze of db/m or db/db mice injected with AAV-Ctr, AAV-Flag-LRPPRC-WT or AAV-Flag-LRPPRC-K223R (n = 10 mice per group).

Figure 2 (J-N): Representative track images (J), escape latency to the platform (K) and swimming speed (N) during the training trials, target entries (L) and time spent in target quadrant (M) in the probe trial of Morris water maze of Ctr or STZ mice injected with AAV-Ctr, AAV-Flag-LRPPRC-WT or AAV-Flag-LRPPRC-K223R (n = 10 mice per group).

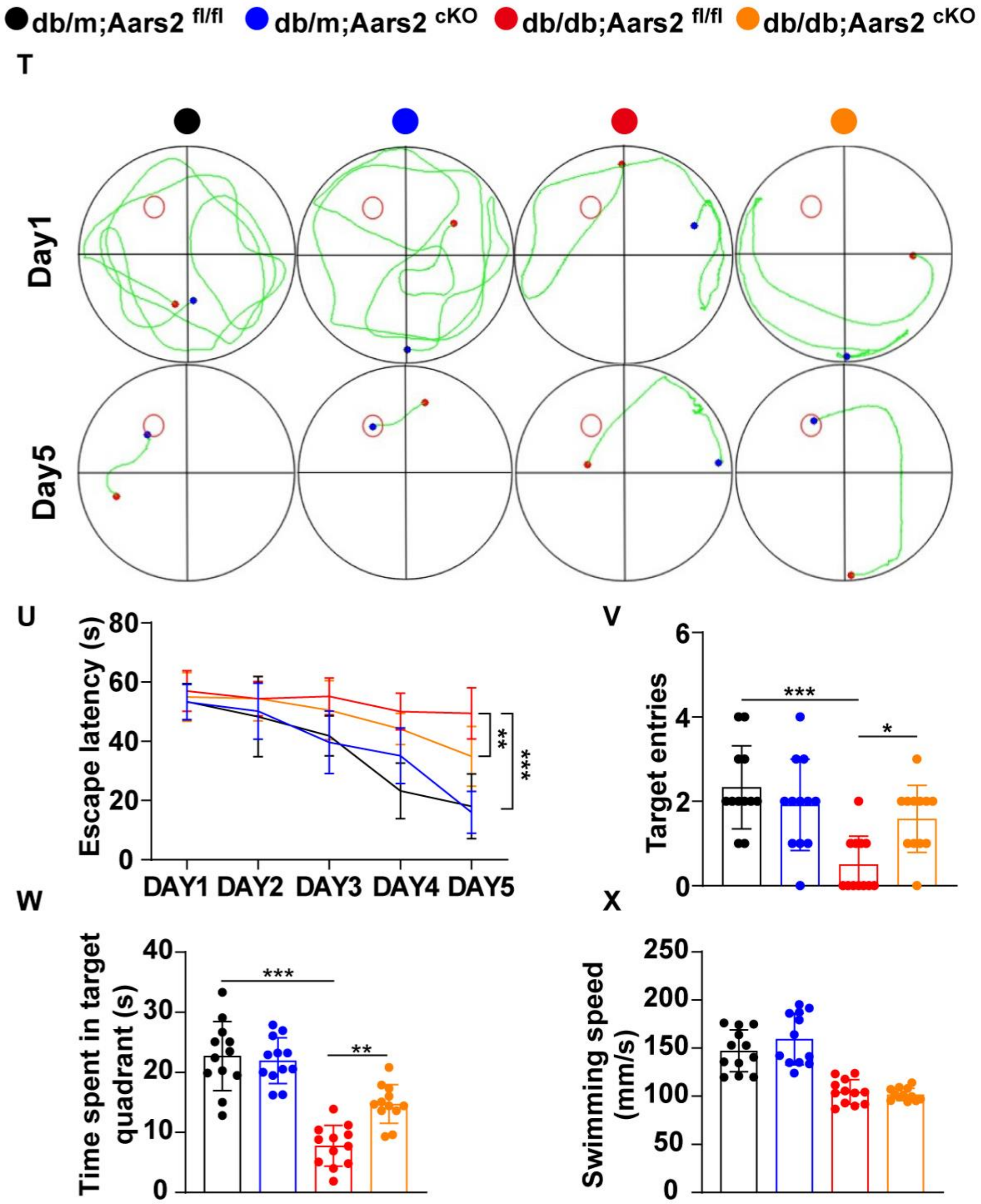

Figure 4 (T-X): Representative track images (T), escape latency to the platform (U) and swimming speed (X) during the training trials, target entries (V) and time spent in target quadrant (W) in the probe trial of Morris water maze of db/m Aars2 $^{fl/fl}$, db/db Aars2 $^{fl/fl}$ mice injected with AAV-CAMKII or AAV-CAMKII-Cre (n = 12 mice per group).

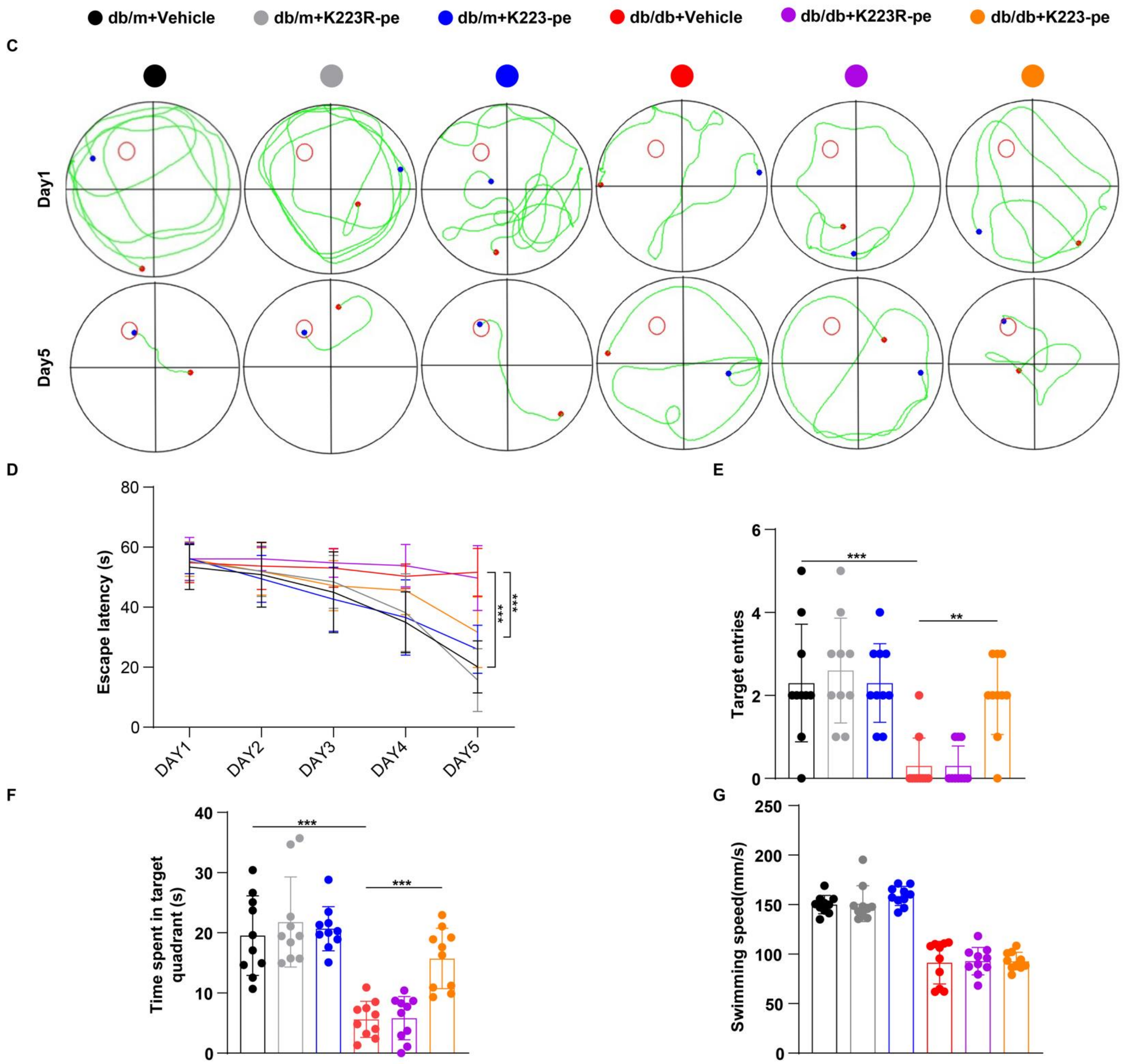

Figure 6 (C-G): Representative track images (C), escape latency to the platform (D) and swimming speed (G) during the training trials, target entries (E) and time spent in target quadrant (F) in the probe trial of Morris water maze of db/m, db/db mice treated with vehicle, K223R-pe (scrambled peptide, 5 mg/kg) or K223-pe (5 mg/kg) (n =10 mice per group).

3) Sample size in behavioral experiments: To address this concern, a sample size calculation has been performed using G*Power software (v3.1.9.4) based on previous study (*Translational Psychiatry PMID: 26731444; Nature Communications, PMID: 36959217; Nature Communications, PMID: 40593621*) with partial $\eta^2 = 0.15$, statistical power = 80%, and $\alpha = 0.05$. Based on this analysis, the number of animals per group has been increased accordingly.

Minor concerns:

(5) In Figure 3A, the authors mapped the LRPPRC-SLIRP binding sites. It would be logical to subsequently examine which binding site is functionally critical for the LRPPRC-SLIRP interaction and whether LRPPRC-K223R affects this binding. Unfortunately, this potentially informative question was not addressed in later experiments.

Response: We are grateful to the reviewer for raising this thought-provoking question. To address this issue, we first identified the functionally critical binding site for the LRPPRC-SLIRP interaction, and then examined whether LRPPRC-K223R affects this binding:

1) Our analysis identified Y440 as the functionally critical binding site for the LRPPRC–SLIRP interaction.

2) Under high-glucose conditions, the K223R mutation reduced lactylation at LRPPRC K223 and enhanced LRPPRC–SLIRP binding. Notably, in the Y440A mutation background, LRPPRC–SLIRP binding was abolished, and the K223R mutation did not restore binding under either normal- or high-glucose conditions.

Results Section, page 7, lines 19-22 -- page 8, lines 1-6:

To examine which binding site is functionally critical for the LRPPRC-SLIRP interaction, we transfected WT LRPPRC or LRPPRC mutants (R437A, H439A, or Y440A) into primary hippocampal neurons. As shown in Figure S3A-B, compared to WT LRPPRC, the Y440A mutant markedly reduced the binding between LRPPRC and SLIRP, whereas R437A and H439A had limited effects. These data identify Y440 as the functionally critical site within the LRPPRC-SLIRP binding interface. To investigate whether LRPPRC K223R affects this binding, we generated a double mutant (K223R/Y440A) LRPPRC. As shown in Figure S3C-D, under high glucose conditions, the K223R mutation significantly enhanced LRPPRC–SLIRP binding compared to the WT group. However, in the Y440A background, the K223R mutation failed to restore LRPPRC–SLIRP interaction, regardless of glucose conditions.

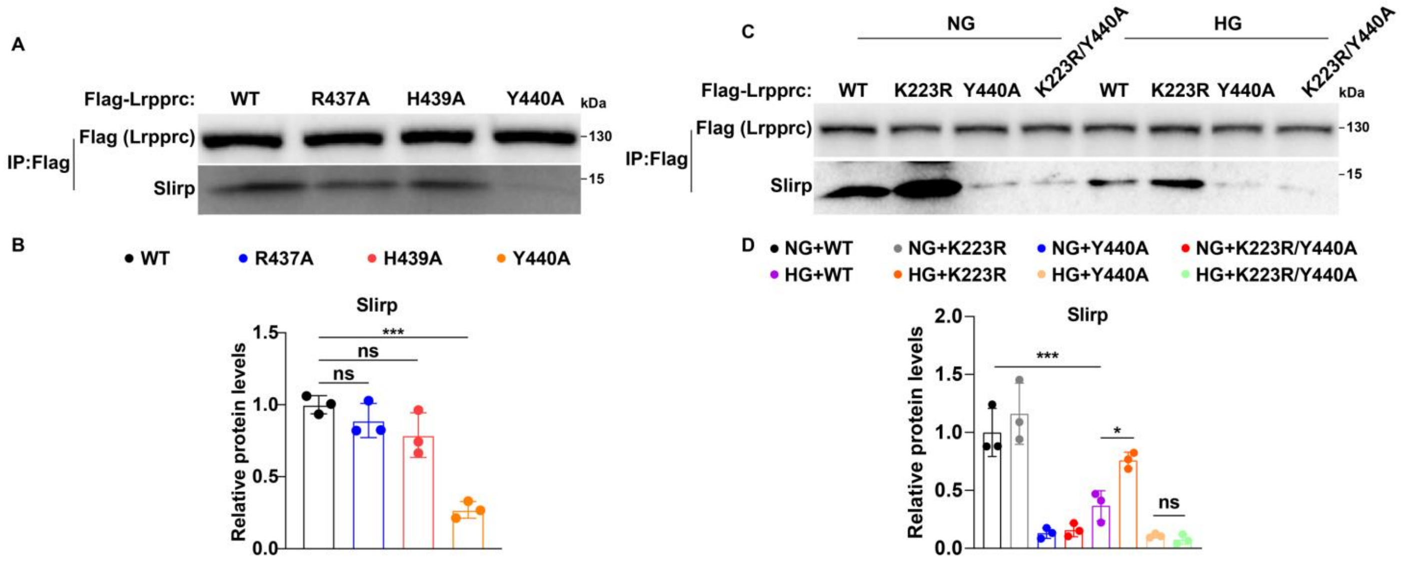

Figure S3 (A-B): Primary hippocampal neurons were transfected with the indicated plasmids, Slirp were analyzed by immunoprecipitation with anti-Flag antibodies and western blotted with the indicated antibodies (A). Quantification analysis of Slirp (B) in the indicated groups (n = 3 biological replicates).

Figure S3 (C-D): Primary hippocampal neurons transfected with the indicated plasmids were treated with normal glucose (NG, 5.5 mmol/L D-glucose) or high glucose (HG, 25 mmol/L D-glucose). Cell lysates were immunoprecipitated with anti-Flag antibodies and western blotted with the indicated antibodies (C). Quantification analysis of Slirp (D) levels in the indicated groups (n = 3 biological replicates).

(6) On page 5, lines 10-11 (Figure 3), the authors state: "LRPPRC K223 lactylation reduces its binding to SLIRP, weakens mitochondrial mRNA stability, and triggers mitochondrial dysfunction." These findings demonstrate that in diabetic mice (but not controls), LRPPRC-K223R promotes LRPPRC-SLIRP interaction, enhances mitochondrial mRNA stability, and ameliorates mitochondrial dysfunction in hippocampal neurons.

On page 5, line 22, the authors report that in hippocampal CA1 neurons from both type 1 and type 2 diabetic mice, lactylation of LRPPRC at K223 is elevated, while the LRPPRC-SLIRP interaction is reduced. However, the data only appear to demonstrate an overall increase in LRPPRC lactylation, rather than specifically at K223. The description should therefore be revised to ensure accuracy.

Response: We sincerely appreciate the reviewer's rigorous and meticulous evaluation of our data. To address this concern, a site-specific antibody against lactylated LRPPRC at K223 has been generated. Using this

antibody, the detection of overall LRPPRC lactylation has been replaced with LRPPRC K223 site-specific lactylation measurement throughout the revised manuscript. The relevant data are now presented in Figures 3B, 4A, 5C, S3E, S3F, S6C, S6H, S6K, and S10A.

(7) Please provide the statistical data plots for Figure S5A and indicate the sample size (n). Additionally, the manuscript states on page 6, lines 27-28 (Figure S5B): "thereby elevating LRPPRC K223 lactylation levels," and on page 7, lines 5-6 (Figure 4A): "resulting in elevated LRPPRC K223 lactylation." Since LRPPRC contains multiple lysine lactylation sites, and AARS2-mediated lactylation is unlikely to be specific for K223, the descriptions should instead refer to "overall lactylation of LRPPRC" rather than specifically to K223.

Response: We thank the reviewer for these insightful suggestions to improve the clarity of our presentation. Statistical data plots and sample sizes (n) have been added to Figure S5A (now Figure S6A-B) as requested. The site-specific antibody targeting lactylated LRPPRC at K223 was employed to examine LRPPRC K223 lactylation levels in Figures S5B (now Figure S6C-G) and 4A (now Figure 4A-E).

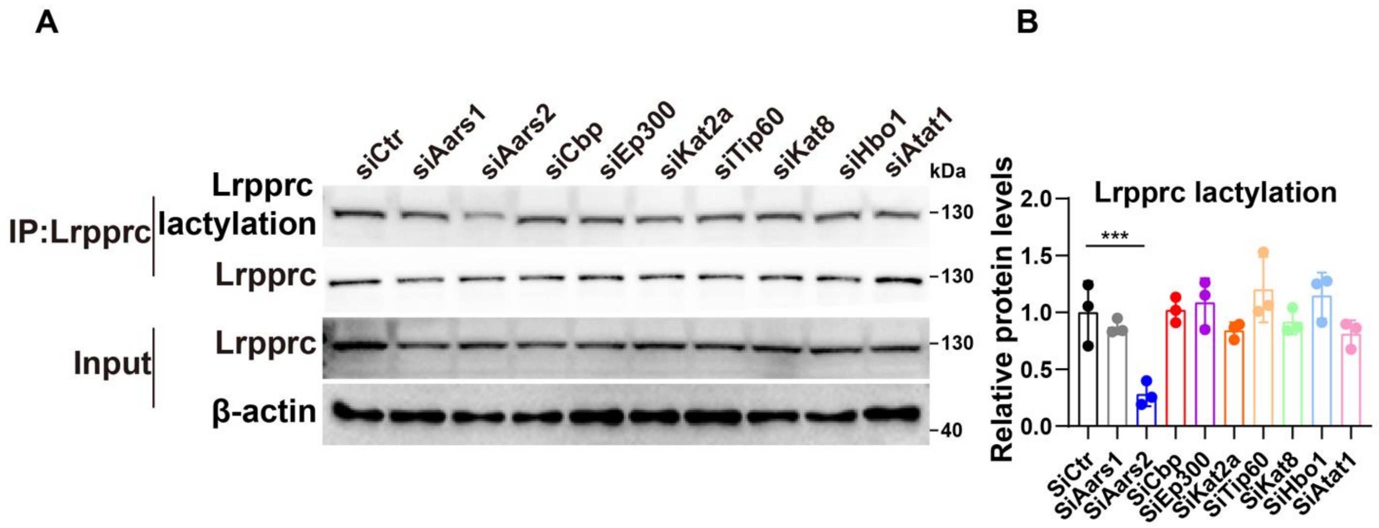

Figure S6 (A-B): Screening of LRPPRC lactylation writer. Lactyltransferases (Aars1, Aars2, Cbp, Ep300, Kat2a, Tip60, Kat8, Hbo1 and Atat1) were knocked down in primary hippocampal neurons, and the lactylation level of LRPPRC was detected by immunoprecipitation and Western blot (A). Quantification analysis of LRPPRC lactylation (B) levels in the indicated groups (n = 3 biological replicates).

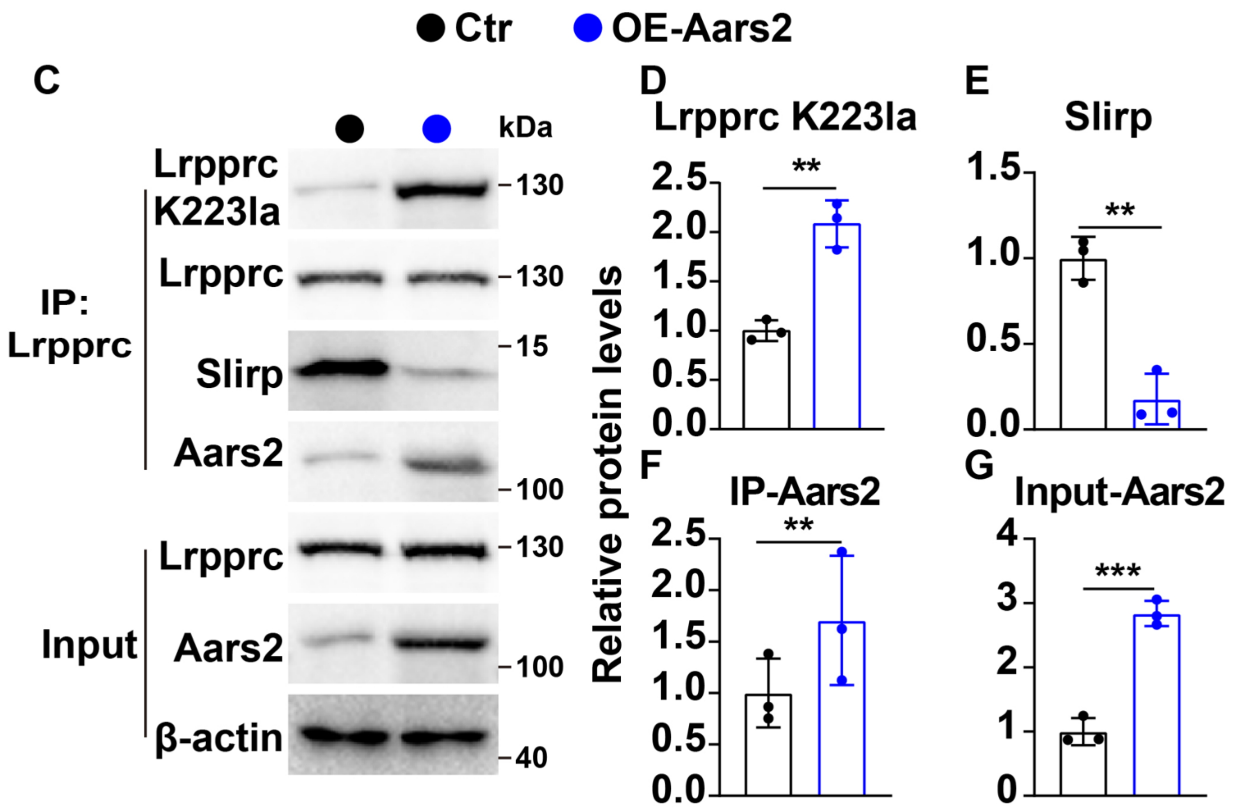

Figure S6 (C-G): Primary hippocampal neurons were transfected with Aars2 overexpression plasmid or control plasmid, and LRPPRC K223 lactylation was determined by immunoprecipitation and western blotted using the indicated antibodies (C). Quantification analysis of LRPPRC K223la (D), Slirp (E), IP-Aars2 (F) and Input-Aars2 (G) levels in the indicated groups (n = 3 biological replicates).

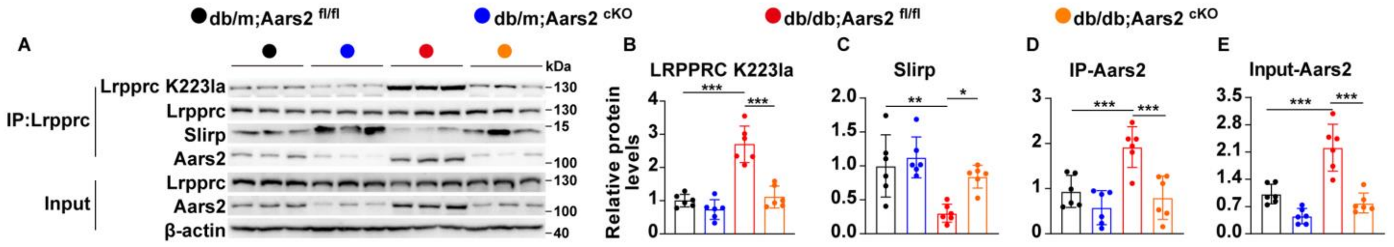

Figure 4 (A-E): Hippocampal CA1 neurons were isolated from db/m Aars2 $^{fl/fl}$, db/db Aars2 $^{fl/fl}$ mice injected with AAV-CAMKII (as a control vector) or AAV-CAMKII-Cre, and cell lysates were immunoprecipitated with LRPPRC antibodies and western blotted with the indicated antibodies (A). Quantification analysis of Lrpprc K223la (B), Slirp (C), IP-Aars2 (D) and Input-Aars2 (E) levels in the indicated groups (n = 6 mice per group).

(8) The pharmacokinetic properties of the short peptide, including half-life and blood-brain barrier penetration efficiency, have not been described and should be elaborated upon.

Response: We thank the reviewer for raising this important consideration. To address this issue, the half-life and blood-brain barrier penetration efficiency of the short peptide K223-pe have been characterized.

Methods Section, page 27, lines 23-26 -- page 28, lines 1-5:

Half-Life Measurement of K223-pe

C57BL/6 mice (n = 6) were intravenously injected with 5 mg/kg of K223-pe. Plasma levels of K223-pe were quantified using ELISA following injection. The plasma elimination half-life ($t_1/_2$) of K223-pe was determined using GraphPad Prism 9.5.1 software.

Evaluation of Blood–Brain Barrier Penetration of K223-pe

To assess the blood–brain barrier permeability of K223-pe, C57BL/6 mice were administered a single intraperitoneal injection of FITC-labeled K223-pe (5 mg/kg). Brains were collected 4 h after injection, and brain tissues were processed for fluorescence imaging to evaluate the distribution of K223-pe.

Results Section, page 11, lines 21-22-- page 12, line 1:

Next, we assessed the blood–brain barrier (BBB) permeability of K223-pe. As shown in Figure S9A, at 4 h after injection, FITC-labeled K223-pe crossed the BBB and was detected in the cytoplasm of neurons in the hippocampal CA1 region. The half-life of K223-pe was 59.27 minutes (Figure S9B).

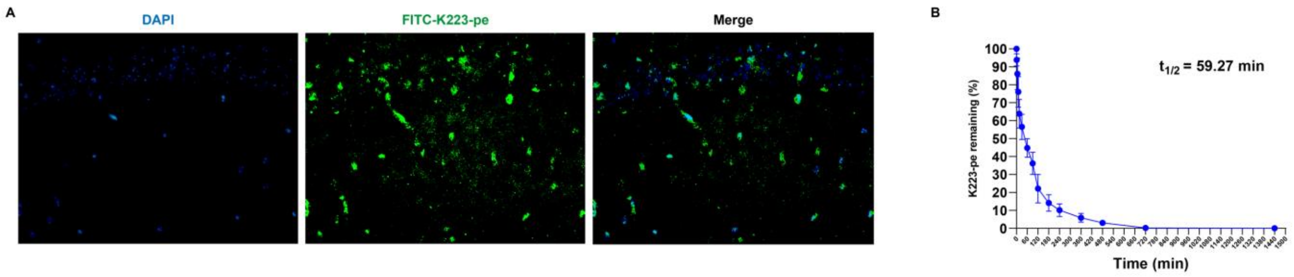

Figure S9 (A): DAPI staining, FITC fluorescence signal, and merged images of hippocampal CA1 tissue sections at 4 h after FITC-K223-pe injection.

Figure S9 (B): Half-life ($t_{1/2}$) of K223-pe (5mg/kg) (n = 6 mice per group).

Referee #2:

In this manuscript, the authors demonstrate that high glucose-induced LRPPRC K223 lactylation impairs mitochondrial functions, leading to neuronal apoptosis and cognitive decline in diabetic model mice. The underlying mechanisms involve reduced LRPPR-SLIRP interaction and decreased mitochondrial mRNA stability. The authors further show that a short peptide targeting LRPPRC K223 lactylation can reverse these defects. Additionally, they show that T2D patients have an elevated plasma LRPPRC K224 lactylation levels. While these findings are intriguing and clinically relevant, several critical issues need to be addressed:

Response: We are deeply grateful to our reviewer for providing exceptional and profound evaluation. Your scholarly critique demonstrates remarkable depth of expertise and has been pivotal in strengthening the scientific rigor and clarity of our study. Below, a detailed point-by-point response to each comment is provided. All revisions in the manuscript are highlighted in yellow. Please note that some figures have reduced resolution in the PDF format; the original high-resolution versions are available in the submission system.

1. The conclusion that LRPPRC K223 lactylation induces neuronal apoptosis relies heavily on flow cytometry data. Additional supportive evidence-such as TUNEL staining in brain sections-would strengthen this claim.

Response: We thank the reviewer for this constructive suggestion. To address this issue, TUNEL staining has been performed to directly demonstrate neuronal apoptosis in vivo.

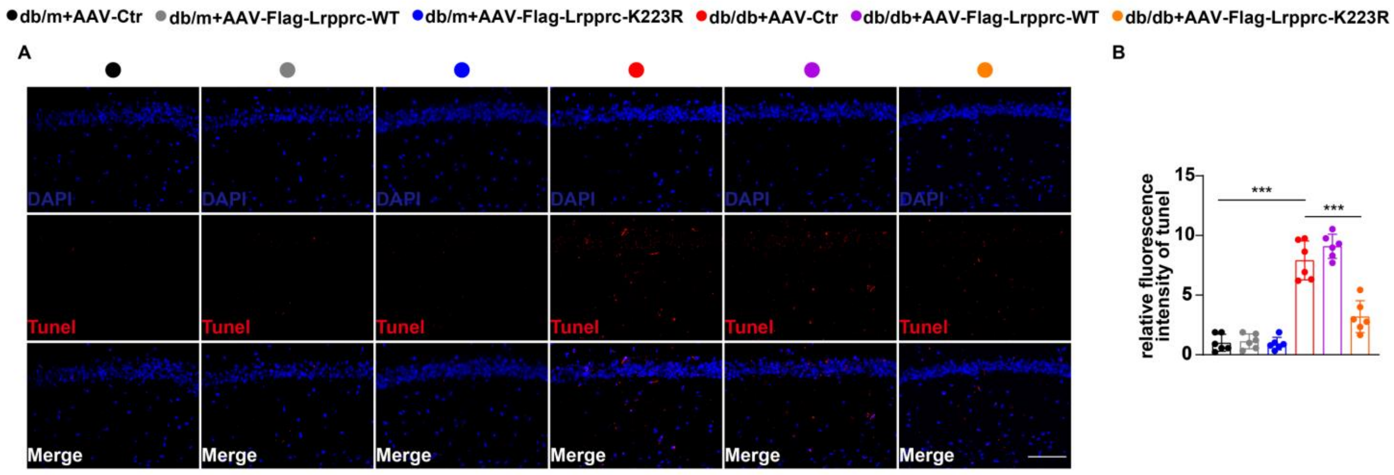

Figure 2 (A-B): Representative images (A) and quantifications (B) showing IF staining of Tunel (red) in the hippocampal CA1 neurons of db/m or db/db mice treated with AAV-Ctr, AAV-Flag-LRPPRC-WT or AAV-Flag-LRPPRC-K223R (n = 6 mice per group). Scale bar: 100 μm.

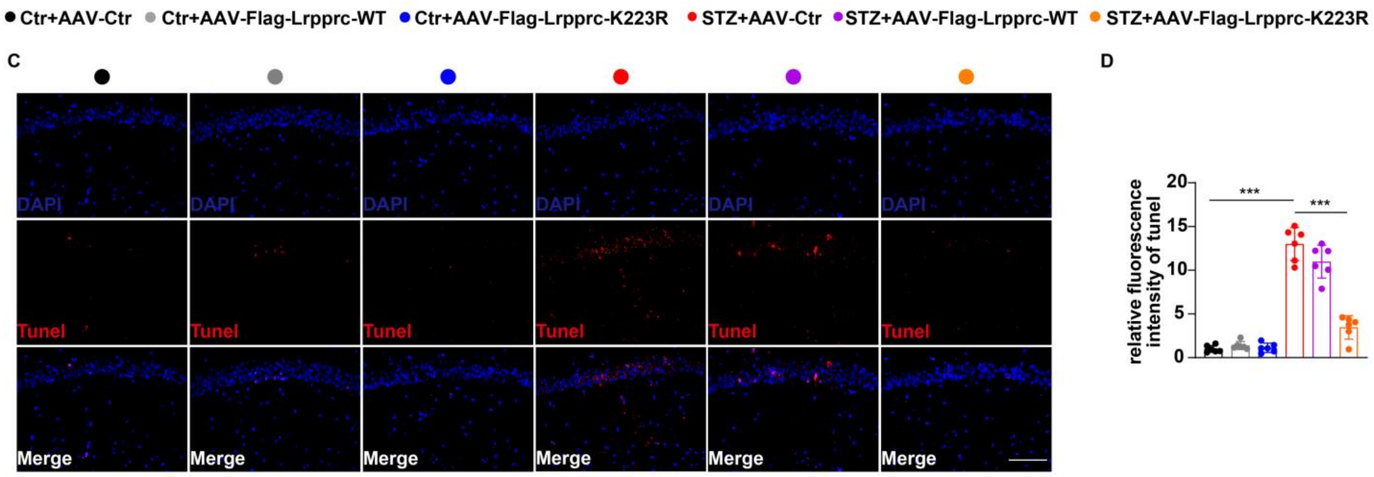

Figure 2 (C-D): Representative images (C) and quantifications (D) showing IF staining of Tunel (red) in the hippocampal CA1 neurons of Ctr or STZ mice treated with AAV-Ctr, AAV-Flag-LRPPRC-WT or AAV-Flag-LRPPRC-K223R (n = 6 mice per group). Scale bar: 100 μm.

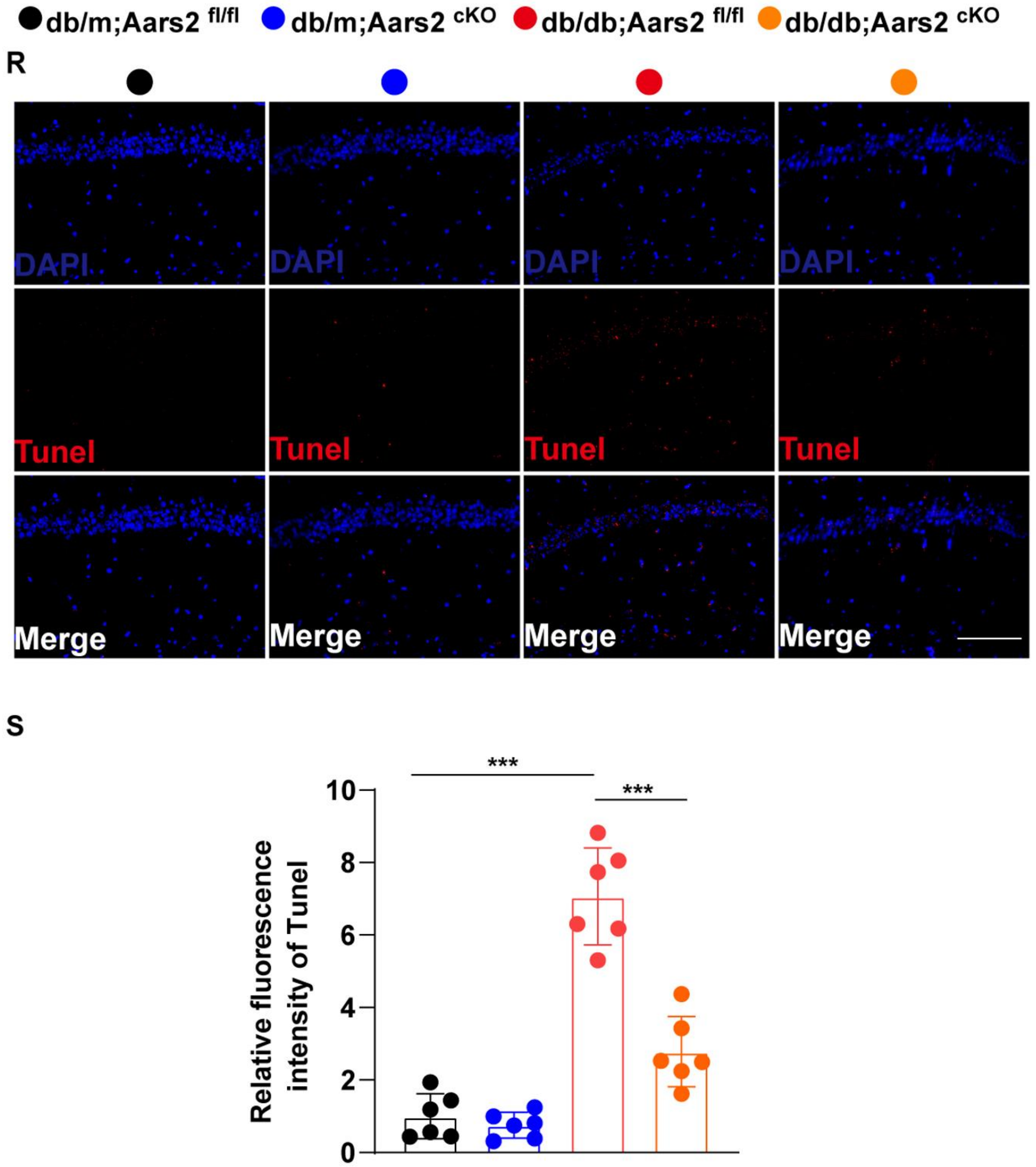

Figure 4 (R-S): Representative images (R) and quantifications (S) showing IF staining of Tunel (red) in the hippocampal CA1 neurons of db/m Aars2 $^{fl/fl}$, db/db Aars2 $^{fl/fl}$ mice injected with AAV-CAMKII or AAV-CAMKII-Cre (n = 6 mice per group). Scale bar: 100 μm.

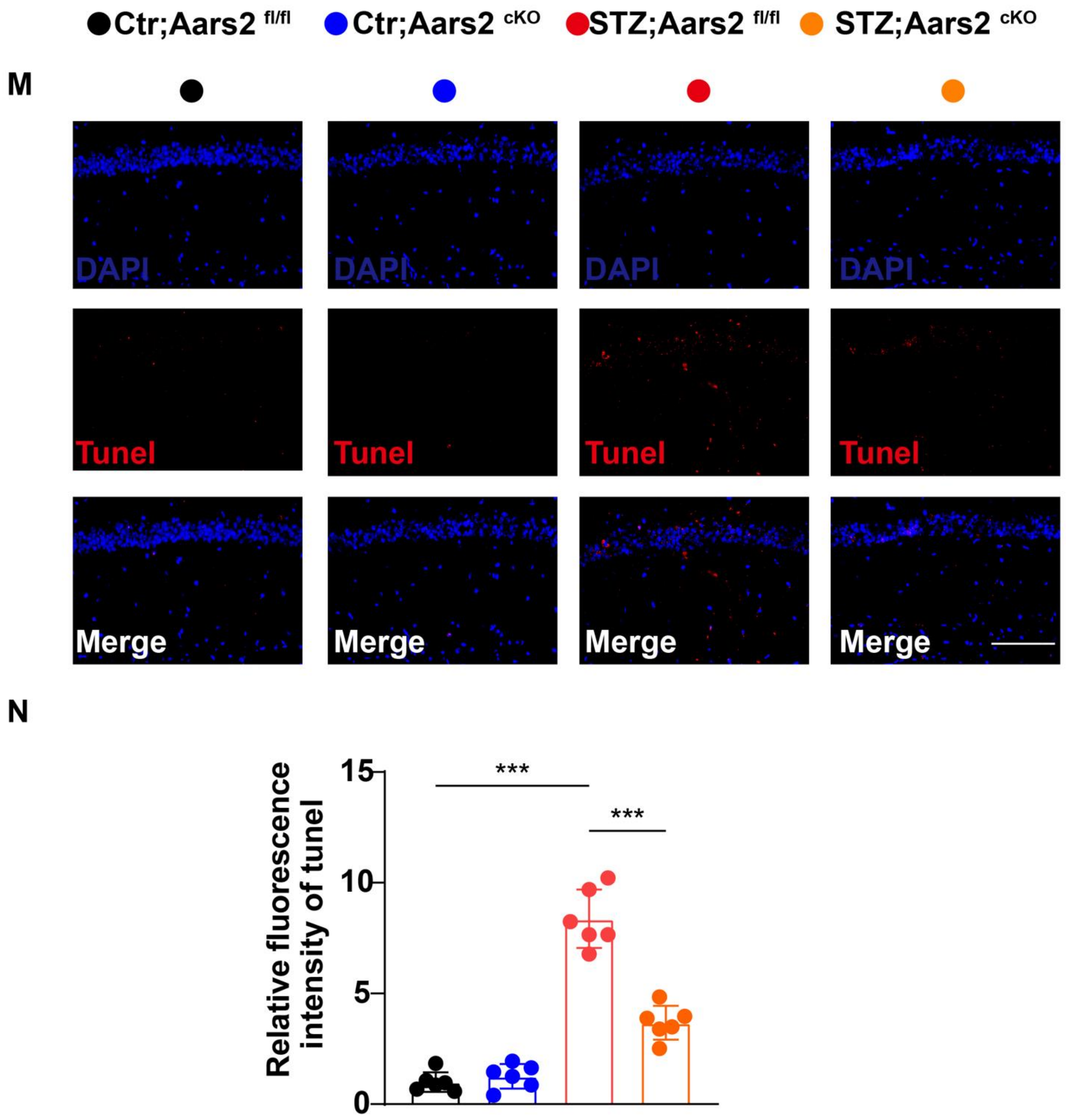

Figure S7 (M-N): Representative images (M) and quantifications (N) showing IF staining of Tunel (red) in the hippocampal CA1 neurons of Ctr Aars2 $^{fl/fl}$, STZ Aars2 $^{fl/fl}$ mice injected with AAV-CAMKII or AAV-CAMKII-Cre (n = 6 mice per group). Scale bar: 100 μm.

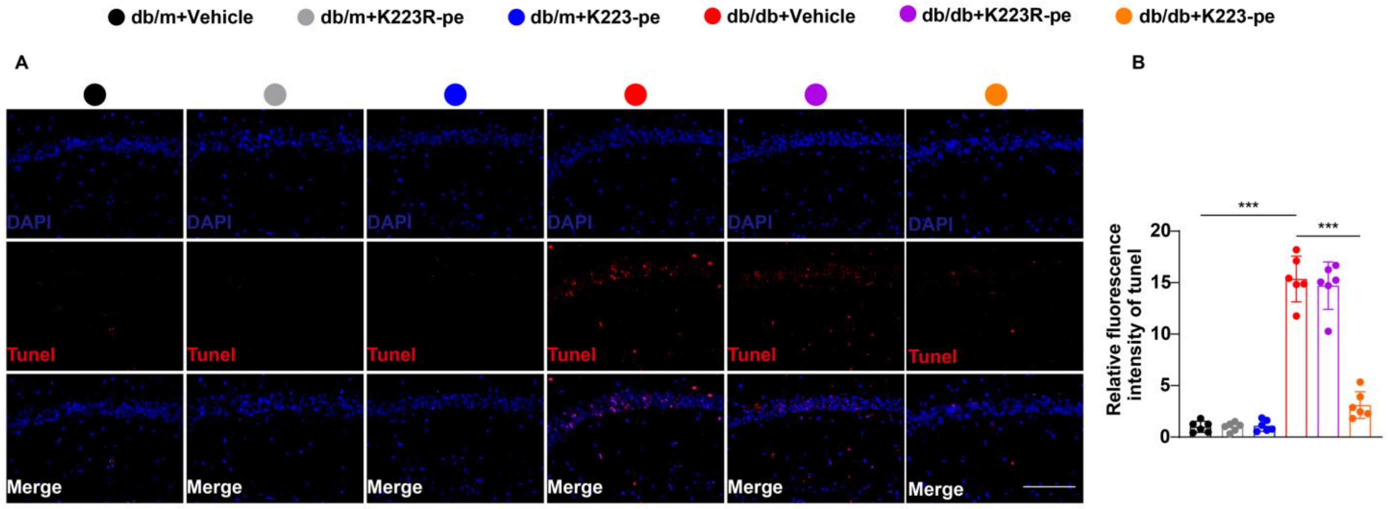

Figure 6 (A-B): Representative images (A) and quantifications (B) showing IF staining of Tunel (red) in the hippocampal CA1 neurons of db/m, db/db mice treated with vehicle, K223R-pe (scrambled peptide, 5 mg/kg) or K223-pe (5 mg/kg) (n = 6 mice per group). Scale bar: 100 μm.

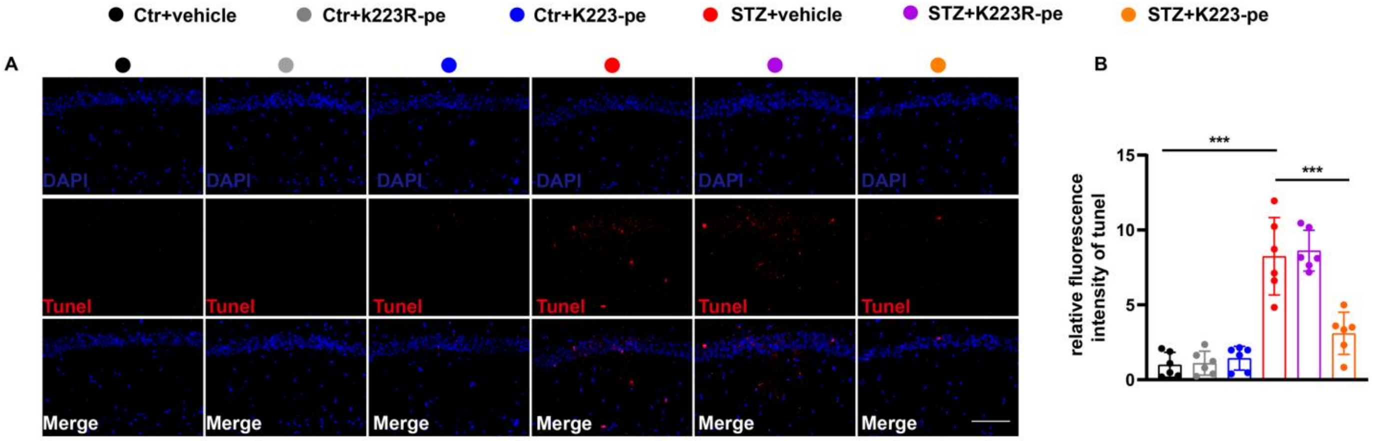

Figure S11 (A-B): Representative images (A) and quantifications (B) showing IF staining of Tunel (red) in the hippocampal CA1 neurons of Ctr, STZ mice treated with vehicle, K223R-pe (scrambled peptide, 5 mg/kg) or K223-pe (5 mg/kg) (n = 6 mice per group). Scale bar: 100 μm.

2. It is unclear whether the authors generated a antibody against LRPPRC K223/k224 lactylation or not, if not, how they specifically measured the plasma LRPPRC K223/k224 lactylation levels in mice and human patients; if yes, the specificity of the antibody needs to be validate.

Response: We are grateful to the reviewer for this excellent suggestion. We have addressed this concern through two approaches:

(1) Generation and validation of site-specific antibody:

A site-specific antibody targeting lactylated LRPPRC at K223 has been generated. The specificity of this antibody was validated using dot blot analysis, a well-established method for antibody specificity verification (*Science Advances, PMID: 37267363; Theranostics, PMID: 39113793; Journal of Experimental & Clinical Cancer Research, PMID: 40849487*).

Methods Section, page 24, lines 14-21:

To prepare a site-specific antibody targeting mouse LRPPRC lactylated at K223, a synthetic polypeptide containing a lactyl group on lysine 223 (ILGFMK(lac)TKDLP) was utilized. Polyclonal antibodies were generated by immunizing New Zealand White rabbits with 0.5 mL of peptide (1 mg/mL) mixed with an equal volume of complete adjuvant, followed by three weekly booster injections, each containing 0.5 ml of the polypeptide (1 mg/ml) plus incomplete adjuvant. Antiserum was harvested via carotid artery seven days post final immunization and affinity-purified using a Protein A/G column. The human LRPPRC K224 is homologous to mouse LRPPRC K223, and the antibody prepared by the above method was also used to detect human LRPPRC lactylated at K224.

Results Section, page 5, lines 20-22:

To investigate whether high glucose induces elevated LRPPRC K223 lactylation in vivo, we employed a specific LRPPRC K223la antibody (Figure S1B) to detect LRPPRC K223 lactylation levels in the hippocampal CA1 region of diabetic mice.

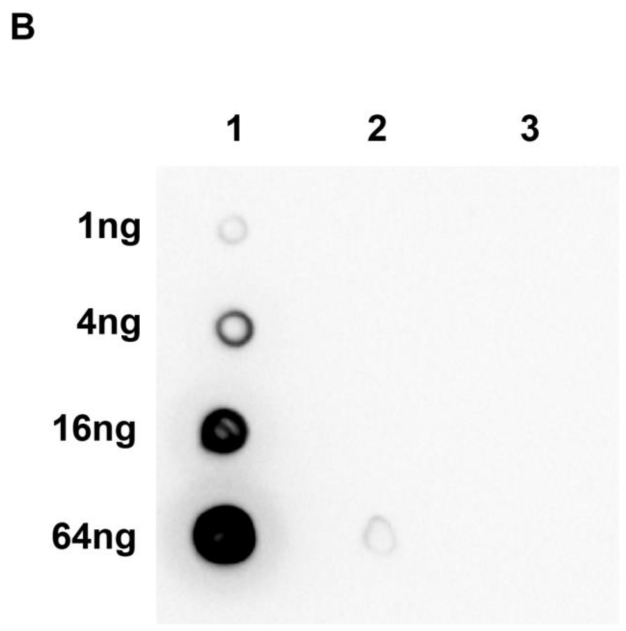

Figure S1 (B): Verification of the specificity of the LRPPRC K223la antibody by dot blots experiment. 1:

modified peptide 1 (ILGFMK(lac)TKDLP); 2: modified peptide 2 (ILGFMKTK(lac)DLP); and 3: non-modified peptide (ILGFMKTKDLP).

(2) Replacement of overall lactylation level of LRPPRC with LRPPRC K223 site-specific lactylation

To provide more precise mechanistic insights, we have replaced the detection of overall lactylation level of LRPPRC with LRPPRC K223 site-specific lactylation detection throughout the manuscript. The relevant data are now presented in Figures 3B, 4A, 5C, S3E, S3F, S6C, S6H, S6K, and S10A.

3. The authors show a significant decrease in mitochondrial RNA levels, but corresponding protein levels were not examined. Since proteins are the functional effectors of mitochondrial activity, assessing their expression is essential.

Response: Thank you for raising this important issue. To resolve this issue, western blot analysis has been performed to examine the corresponding mitochondrial protein levels.

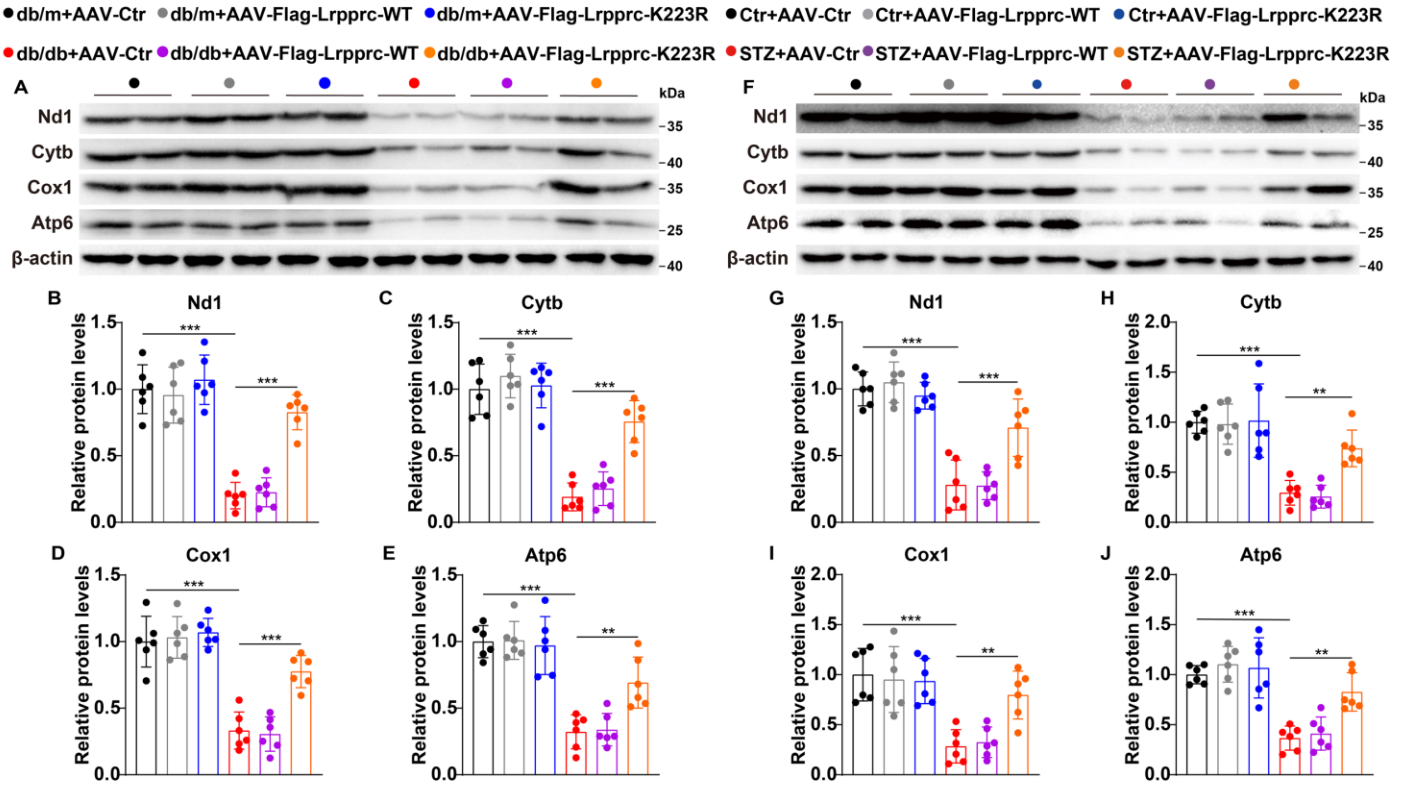

Figure S4 (A-E): Immunoblots (A) and quantification analysis of Nd1 (B), Cytb (C), Cox1 (D), and Atp6 (E) expression levels in the hippocampal CA1 neurons of db/m, db/db mice received stereotactic injections of AAV-Ctr, AAV-Flag-LRPPRC-WT or AAV-Flag-LRPPRC-K223R to specifically express either wild-type LRPPRC or the K223R mutant LRPPRC (n = 6 mice per group).

Figure S4 (F-J): Immunoblots (F) and quantification analysis of Nd1 (G), Cytb (H), Cox1 (I), and Atp6 (J) expression levels in the hippocampal CA1 neurons of Ctr, STZ mice received stereotactic injections of AAV-Ctr, AAV-Flag-LRPPRC-WT or AAV-Flag-LRPPRC-K223R to specifically express either wild-type LRPPRC or the K223R mutant LRPPRC (n = 6 mice per group).

4. The source of elevated plasma LRPPRC K224 lactylation in T2D patients remains unclear. Is it derived from brain neurons or peripheral tissues?

Response: Thank you for raising this thought-provoking question. We have comprehensively discussed this question and added it to the **Limitation of the Study** section.

Limitation of the Study Section, page 18, lines 12-18:

First, LRPPRC is expressed across multiple organs (Lei *et al*, 2016; Ruzzenente *et al.*, 2012; Sasarman *et al*, 2015), indicating that LRPPRC K223la detected in plasma may derive from diverse tissue sources. Notably, in mice experiments, we found a significant positive correlation between LRPPRC K223la in plasma and hippocampal CA1 neurons, suggesting that plasma LRPPRC K223la might be partially derived from hippocampal CA1 neurons. However, the inability to procure tissue specimens in patients precludes direct experimental verification of human plasma LRPPRC K224la sources, representing a limitation of this study that warrants future investigation.

5. Figure S10B, LRPPRC K224 lactylation is elevated in metformin users despite the authors' suggestion that lower lactylation is beneficial in T2D. This apparent contradiction requires clarification.

Response: We thank the reviewer for this insightful observation, which demonstrates exceptional attention to scientific rigor. A comprehensive discussion of this phenomenon and its potential mechanisms have been added to the revised manuscript.

Discussion Section, page 17, lines 21-22-- page 18, lines 1-3:

Notably, metformin users exhibited elevated plasma LRPPRC K224 lactylation. While metformin is highly effective in glycemic control and remains a first-line treatment for type 2 diabetes, several studies have reported cognitive side effects associated with metformin use (Moore *et al*, 2013; Porter *et al*, 2019). Our finding that metformin users exhibit elevated plasma LRPPRC K224 lactylation suggests a potential mechanism underlying these cognitive side effects.

6. The sample size used for behavioral tests appears insufficient. Increasing the number of animals would improve the statistical robustness of the conclusions.

Response: Thank you for raising this important issue. To address this concern, a sample size calculation has been performed using G*Power software (v3.1.9.4) based on previous study (*Translational Psychiatry PMID: 26731444; Nature Communications, PMID: 36959217; Nature Communications, PMID: 40593621*) with partial $\eta^2 = 0.15$, statistical power = 80%, and $\alpha = 0.05$. Based on this analysis, the number of animals per group has been increased accordingly.

7. Molecular weight markers (kDa) are missing from the immunoblot figures in the current version of the manuscript. These should be clearly indicated for each blot.

Response: Thank you for pointing this out. Molecular weight markers (kDa) have been added to all immunoblot figures as requested.

Referee #3 (Remarks for Author):

This is an interesting study presenting cell, mouse, and human data to show that lactylation of LRPPRC is linked to cognitive function in diabetes.

Response: We express our profound appreciation to our reviewer for the brilliant and penetrating assessment. Your comments exemplify distinguished scientific acumen and have greatly enhanced the overall quality of our research. Below, a detailed point-by-point response to each comment is provided. All revisions in the manuscript are highlighted in yellow. Please note that some figures have reduced resolution in the PDF format; the original high-resolution versions are available in the submission system.

Major:

1. The analysis of mitochondrial function is very superfluous. It is imperative to use Seahorse analysis to verify an impact on mitochondrial function beyond ROS and MMP.

Response: We sincerely thank the reviewer for this valuable suggestion. In response to this comment, we have now performed Seahorse analysis to measure the oxygen consumption rate (OCR) and comprehensively assess mitochondrial function.

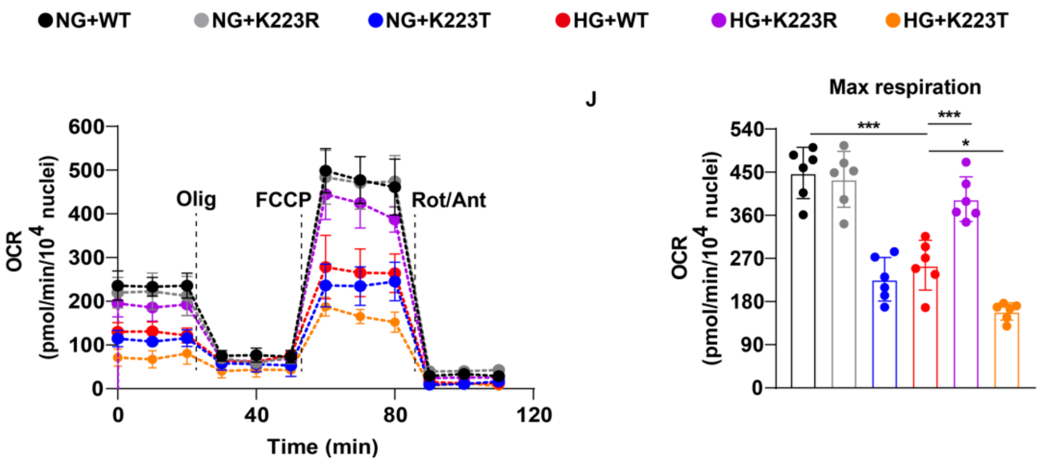

Figure S5 (I-J): OCR measurements of primary hippocampal neurons with endogenous LRPPRC stably silenced and expressing either wild-type (WT) LRPPRC, K223R LRPPRC or K223T LRPPRC, treated with normal glucose (NG, 5.5 mmol/L D-glucose) or high glucose (HG, 25 mmol/L D-glucose) under basal conditions and in response to oligomycin (Olig), *p*-trifluoromethoxy carbonyl cyanide phenylhydrazone (FCCP), antimycin A/rotenone (Rot/Ant) (I); the quantification of the maximal OCR (J) (n= 6 biological replicates).

2. It will be important to include indirect calorimetry to supplement the behavioral data with energy metabolism data in the context of diabetes.

Response: Thank you for the important and insightful suggestion that clearly reflects your deep expertise and profound understanding of energy metabolism in the context of diabetes.

1) We sincerely apologize that our laboratory does not currently have access to an indirect calorimetry system, so we are unable to perform these experiments at this stage. However, to address this important point, we have carefully reviewed the relevant literature and incorporated discussion of previously reported indirect calorimetry findings from db/db and STZ-induced diabetic mouse models into the revised **Discussion** section.

2) Given your extensive expertise and deep knowledge in this field, we would be most grateful if you could provide guidance on alternative experimental approaches you deem scientifically rigorous and acceptable for assessing energy metabolism. If you consider additional experiments necessary for the revision, we are fully committed to conducting them according to your recommendations and will include these results in our next submission.

3) Furthermore, your guidance on whether the selected references appropriately represent current knowledge in this field would be highly valued.

Discussion Section, page 16, lines 3-7:

Consistent with these findings, previous studies using indirect calorimetry have reported impaired energy metabolism in both db/db and STZ diabetic mice (Lafferty *et al*, 2022; López-Soldado *et al*, 2022). These observations suggest that mitochondrial dysfunction may lead to cognitive impairment through disruption of energy metabolism. Future studies should employ indirect calorimetry to directly validate this mechanism.

3. The db/db and STZ models are type 2 and type 1 models, but neither are accepted to be close to the human situation (beyond hyperglycemia). For that, DIO and NOD models for T2D and T1D should have been used. Please comment and perhaps change the major conclusions based on that.

Response: We sincerely thank the reviewer for this important and insightful comment. We chose db/db and STZ-induced diabetic mice for this study primarily because these models exhibit more severe and consistent hyperglycemia, as well as higher incidence of cognitive dysfunction, which are critical for investigating the mechanistic link between diabetes and cognitive impairment. DIO and NOD mice typically display milder hyperglycemia and lower incidence of cognitive deficits (*Cardiovascular Diabetology, PMID: 24460834; The Journal of Nutritional Biochemistry, PMID: 36372312; PLoS One, PMID: 34587222; Physiology & Behavior, PMID: 25542778; Journal of Clinical Investigation, PMID: 10930440; Behavioural Brain Research, PMID: 16191444*). Indeed, several studies have failed to detect cognitive impairment in these models (*Behavioural Brain Research, PMID: 16191444; PLoS One, PMID: 34587222; Physiology & Behavior, PMID: 25542778*). Consistent with these reports, our preliminary experiments using DIO and NOD models showed insufficient hyperglycemia and barely detectable cognitive dysfunction, making it challenging for us to conduct experiments.

The necessity for further validation in DIO and NOD models to confirm the translational potential of our findings has been clearly stated in **the limitation of the study** section.

Additionally, in response to your suggestion, the conclusions have been revised to specify "type 2 and type 1 diabetic mice" rather than using the broader term "diabetes" to more accurately reflect the scope of the experimental evidence.

Limitation of the Study Section, page 18, lines 18-20:

Second, because diet-induced obese (DIO) and non-obese diabetic (NOD) mice more closely recapitulate the human diabetic situation, the underlying mechanisms should be validated in DIO and NOD mouse models in future studies to improve translational relevance.

4. K223 could also be ubiquitinylated. Please verify if there is a change in that as the usage of the mutant might interfere with this PTM and subsequent regulation.

Response: Thank you for raising this important point. In the revised manuscript, we have assessed whether the K223R and K223T mutants affect LRPPRC ubiquitination levels and comprehensively discussed the results.

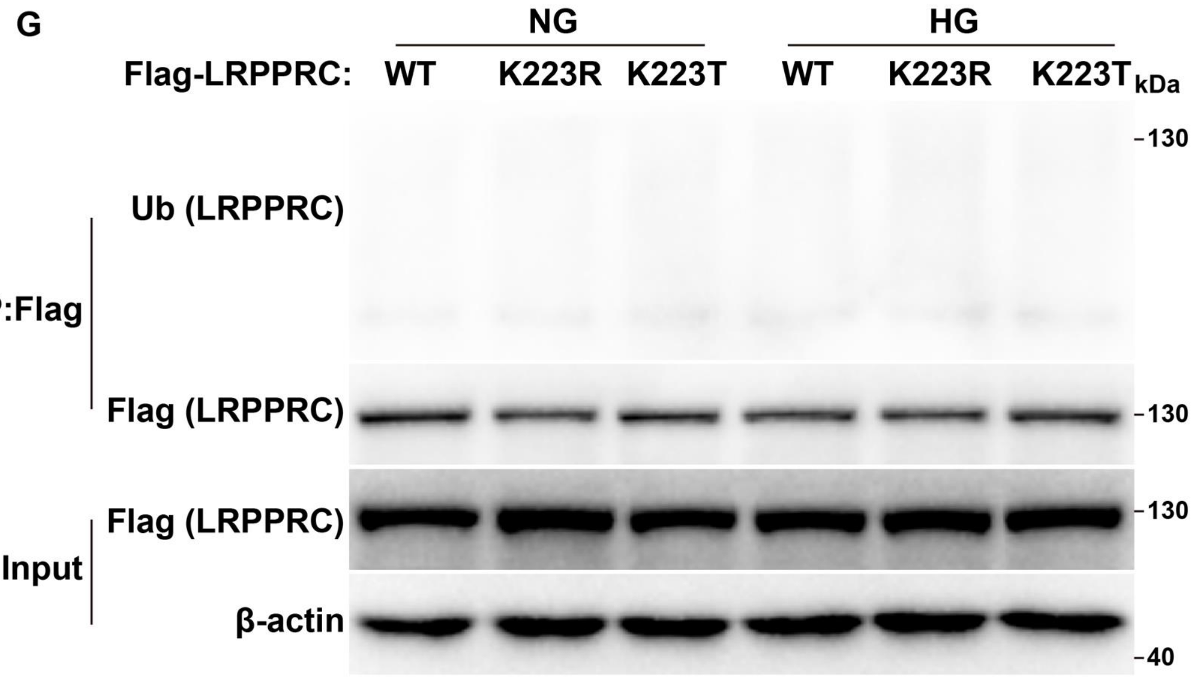

Figure S2 (G): Primary hippocampal neurons, which were stably silenced endogenous LRPPRC and expressed Flag-tagged wild-type (WT) LRPPRC, K223R LRPPRC or K223T LRPPRC, were treated with normal glucose (NG, 5.5 mmol/L D-glucose) or high glucose (HG, 25 mmol/L D-glucose). Cell lysates were immunoprecipitated with anti-Flag antibodies and western blotted with the indicated antibodies (n = 3 biological replicates).

Results Section, page 6, lines 17-21:

Previous studies have reported that high glucose may affect ubiquitination at specific sites, but these alterations appear to be relatively infrequent (Liang *et al*, 2024; Zhou *et al*, 2007). Consistent with this, our data indicate that LRPPRC exhibits minimal ubiquitination under both high- and normal-glucose conditions; the LRPPRC K223R and K223T mutations do not affect this PTM and subsequent regulation (Figure S2G).

5. I am not a brain expert but it will be important to use in situ hybridization or similar to show that it is hippocampal LRPPRC. I fail to see the proof in vivo in brains here. Same applies to the usage of the AAVs and their appropriate targeting to these regions, which should be demonstrated by imaging of histology.

Response: Thank you for this insightful suggestion. We have performed immunofluorescence staining to confirm both localization of LRPPRC K223 lactylation and AAV targeting specificity in the hippocampal CA1 region.

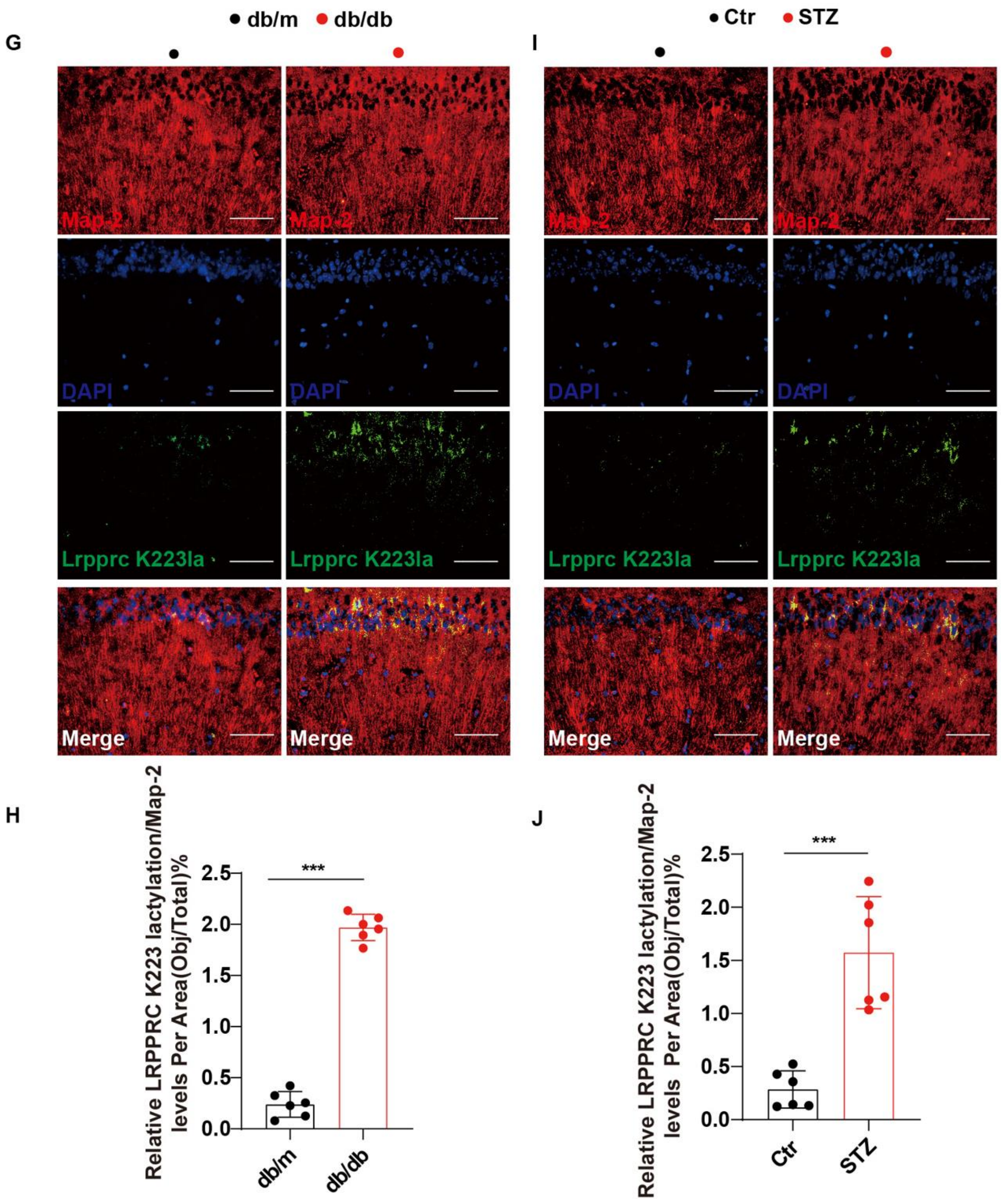

Figure 1 (G-H): Representative images (G) and quantifications (H) showing IF staining of Lrpprc K223la (green) and MAP-2 (red) in the hippocampal CA1 neurons of db/m or db/db mice (n = 6 mice per group). Scale bar: 100 μm.

Figure 1 (I-J): Representative images (I) and quantifications (J) showing IF staining of Lrpprc K223la (green) and MAP-2 (red) in the hippocampal CA1 neurons of Ctr or STZ mice (n = 6 mice per group). Scale bar: 100 μm.

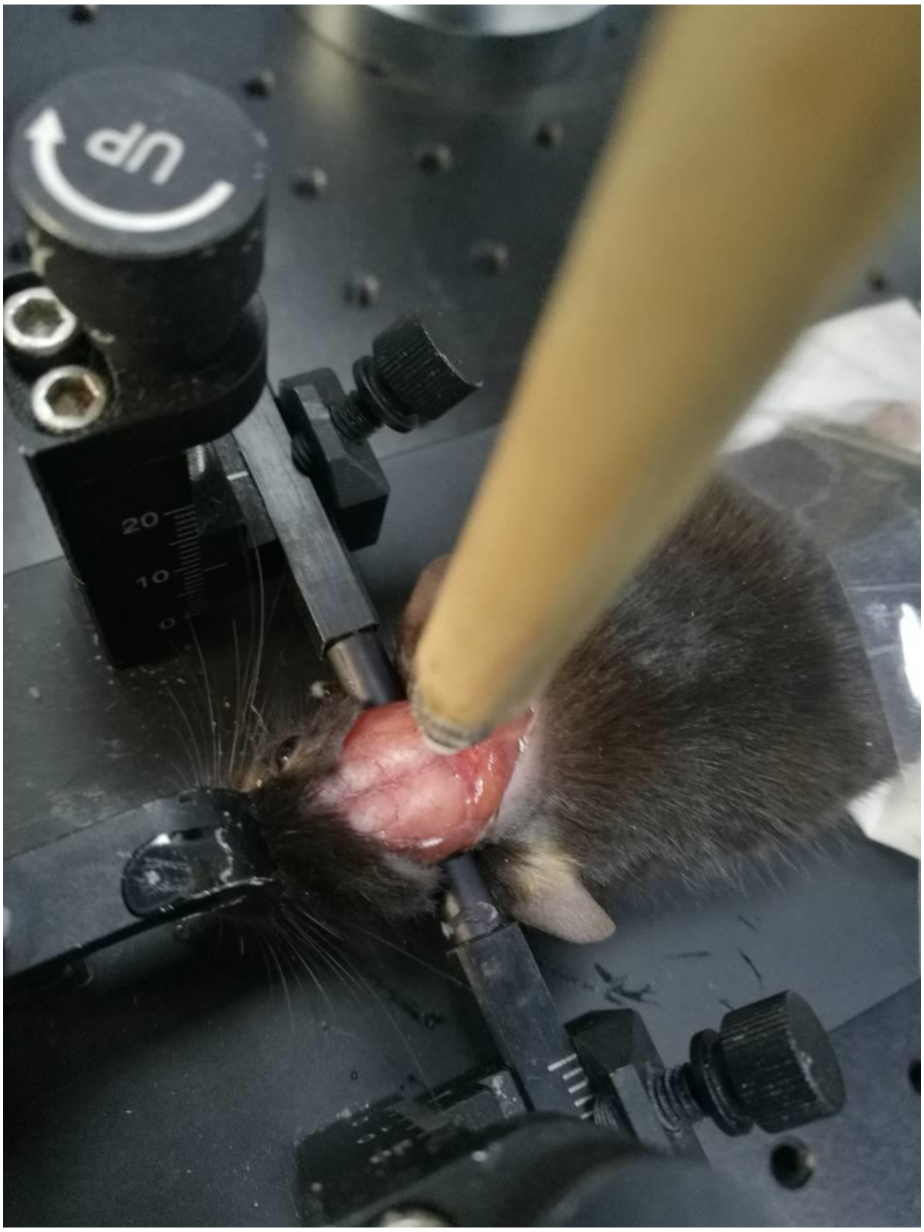

Mouse mounted on stereotaxic apparatus with exposed bregma

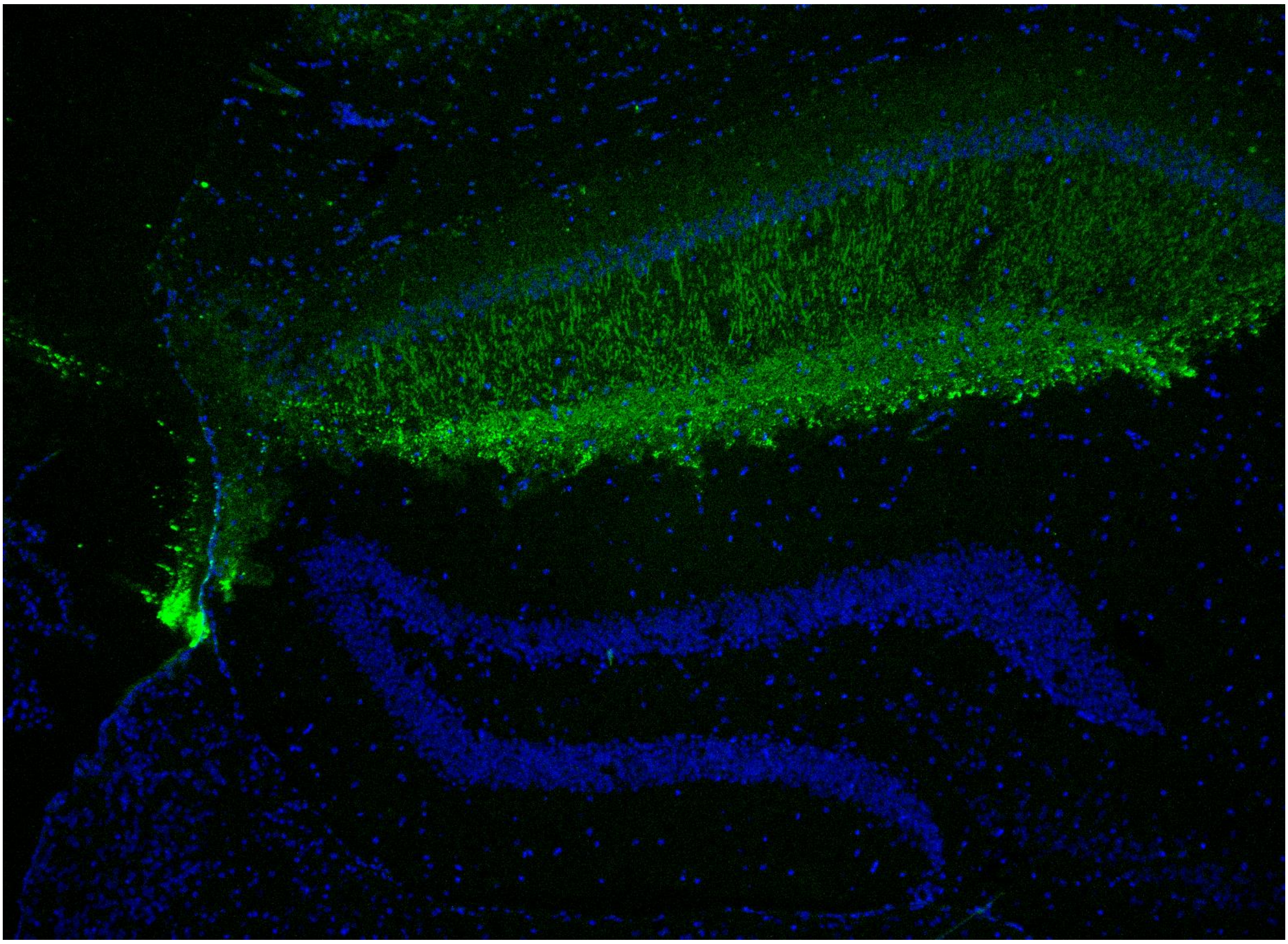

AAV targeting specificity in the hippocampal CA1 region.

6. Please elaborate on the power calculation of the mouse experiments and if they were independently reproduced. The mouse methods part also lacks more information on breeding, housing, weaning. Altogether, it is imperative to revise all methods part to be much more detailed.

Response: We sincerely thank the reviewer for these comprehensive suggestions, which reflect deep expertise in animal experimental methodology. To address this concern, a sample size calculation have been performed using G*Power software (v3.1.9.4) based on previous study (*Translational Psychiatry PMID: 26731444; Nature Communications, PMID: 36959217; Nature Communications, PMID: 40593621*) with partial $\eta^2 = 0.15$, statistical power = 80%, and $\alpha = 0.05$. Based on this analysis, the number of animals per group has been increased accordingly.

Moreover, information regarding mouse breeding, housing, and weaning procedures has been added to the Methods section. The Methods section has been substantially revised to provide more detailed experimental information.

Methods Section, page 19, lines 21-23 – page 20, lines 1-9:

BKS.Cg-Dock7^{m} +/+ Lepr ^{db}/J (db/m) mice were obtained from Jackson Laboratory, Aars2 $^{fl/fl}$ mice were sourced from GemPharmatech Co. Ltd, and C57BL/6 mice were supplied by Changsha Hunan Silaike Jingda Laboratory Animal Co., Ltd. The db/db mice were generated by self-breeding db/m mice, as db/db mice are sterile. Aars2-floxed (Aars2 $^{fl/fl}$) mice were created by inserting two flox sequences (ATAACTTCGTATAGCATACATTATACGAAGTTAT) into the terminals of Aars2 exons 1-5. Genomic DNA extracted from tail tips was used for genotyping, with the following primers utilized for Aars2 $^{fl/fl}$ mice: F- AAGCAACAGGAGAAGAGGTGTTGG; R-TAACCATCTCAGCAGCCCAGCAT. Aars2 $^{fl/+}$ mice were intercrossed to obtain Aars2 $^{fl/fl}$ mice. Aars2 $^{fl/fl}$ mice and db/m mice were crossed to produce Aars2 $^{fl/+}$ db/m mice, and Aars2 $^{fl/fl}$ db/db mice were obtained by further breeding between Aars2 $^{fl/+}$ db/m heterozygous mice. All mice were weaned at postnatal day 21 and housed in individually ventilated cages under standard laboratory conditions. Animals were maintained on a 12-hour light/dark cycle (lights on at 07:00, off at 19:00) at a controlled temperature of 23–24°C with ad libitum access to standard laboratory chow and water.

Minor:

7. Please include all uncropped Western blots with legends indicating what is what.

Response: Thank you for pointing this out. Uncropped Western blot images with legends have been provided as supplementary materials

8. The legends in the figures are suboptimal and make it difficult to follow the stream of thought and understand the data.

Response: Thank you for this valuable feedback. All figure legends have been revised to make the data easier to follow and interpret.

28th Feb 2026

Dear Prof. Zheng,

Thank you for the submission of your revised manuscript to EMBO Molecular Medicine. We have now received the enclosed report from the referees who were asked to re-assess it. As you will see, the referees are now supportive, and I am pleased to inform you that we will be able to accept your manuscript pending the following amendments:

1. Please address the the remaining minor concerns from Reviewer #3.

On a more editorial level:

1. Please provide a 'Synopsis' to further enhance discoverability. Synopses are displayed on the journal webpage and are freely accessible to all readers. They include a short stand first (maximum of 300 characters, including space) as well as 2-5 one-sentences bullet points that summarizes the paper. Please write the bullet points to summarize the key NEW findings. They should be designed to be complementary to the abstract - i.e. not repeat the same text. We encourage inclusion of key acronyms and quantitative information (maximum of 30 words / bullet point). Please use the passive voice. Please attach these in a separate file or send them by email, we will incorporate them accordingly.

Please provide visual abstract to illustrate your article as a PNG file 550 px wide x 300-600 px high.

2. The paper explained: EMBO Molecular Medicine articles are accompanied by a summary of the articles to emphasize the major findings in the paper and their medical implications for the non-specialist reader. Please provide a draft summary of your article highlighting

- the medical issue you are addressing,

- the results obtained and

- their clinical impact.

This may be edited to ensure that readers understand the significance and context of the research. Please refer to any of our published articles for an example.

3. Please download and fill our Reagents and Tools Table template (.docx), which you can find in our author guidelines: https://link.springer.com/journal/44320/submission-guidelines#structuredmethods. When submitting your revised manuscript, please DO NOT include the Reagents and Tools Table in the Methods section of the manuscript but upload it as a separate file choosing the file type "Reagent Table".

4. A complete author checklist, which you can download from our author guidelines (https://media.springernature.com/original/springer-cms/rest/v1/content/27825796/data/v1). Please insert information in the checklist that is also reflected in the manuscript.

5. At EMBO Press we ask authors to provide source data for the main manuscript figures. You should have already received a separate email with instructions for providing source data with your revised manuscript, including how to upload and organize the files.

6. Microscopy cells are pixelated in the figure set. This reduction in resolution is commonly caused by converting original 16-bit TIFF files to RGB format for publication. While this is not inherently problematic, it can raise concerns about image integrity for critical readers.

To avoid any misunderstanding and to meet EMBO Press standards, we kindly ask that you:
* Submit the source data for all microscopy cells at the captured original data resolution.

7. Please add the authors' names and affiliations back to the manuscript file, directly below the title.

8. Please complete the author contribution information in the submission system. A seprate "authors' contributions" section should not be included in the manuscript file.

9. Please remove the following statement: "Due to the double-blind review process, all information that could potentially reveal the authors' identities will be concealed during the review period. The relevant information will be released following the publication of the article." Please ensure that all relevant information is included in the manuscript at this stage.

10. Please add a "Data availability" statement in the manuscript file. Please include the following single sentence in this section- "This study includes no data deposited in external repositories".

11. "Competing interests" should be renamed to "DISCLOSURE AND COMPETING INTERESTS STATEMENT" and should be added to the manuscript file.

12. The "Acknowledgements" section should be included in the manuscript file. Currently, GYYK2025010, S202410601098, and Diabetes_ZZ01 are listed in the Acknowledgements but have not been entered in the submission system. Please ensure that these are also added to the system.

13. Appendix: Please remove the line numbers and yellow highlights; add a table of contents with page numbers; update the nomenclature to "Appendix Figure S1," etc., and "Appendix Table S1"; and update the corresponding callouts in the main manuscript text accordingly.

14. Please address the following issues with figure legends:
- Please define the annotated p values ****/***/**/* as well as provide the exact p-values for the same in the legend of figure 7D as appropriate.
-Please note that the exact p values are not provided in the legends of figures 1H, J; 2B, D, F-I, K-N; 3C-F, G-J, L, N; 4B-E, F-I, J-M, O, Q, S, U, V, W, X; 5D-I, J-M, O, Q; 6B, E, F, G; 7B, C, E, F, G, H
-Please indicate the statistical test used for data analysis in the legends of figures 1D, 7A-C; 7D, G, H.
-Please note that the box plots need to be defined in terms of minima, maxima, centre, bounds of box and whiskers, and percentile in the legends of figure 7D
-Please note that information related to n is missing in the legends of figure 7D

I look forward to seeing a revised form of your manuscript as soon as possible.

Sincerely,
Jingyi Hou

Jingyi Hou
Senior Editor
EMBO Molecular Medicine

*** Instructions to submit your revised manuscript ***

*** PLEASE NOTE *** As part of the EMBO Publications transparent editorial process initiative (see our Editorial at https://www.embopress.org/doi/pdf/10.1002/emmm.201000094), EMBO Molecular Medicine will publish online a Review Process File to accompany accepted manuscripts.

In the event of acceptance, this file will be published in conjunction with your paper and will include the anonymous referee reports, your point-by-point response and all pertinent correspondence relating to the manuscript. If you do NOT want this file to be published, please inform the editorial office at contact@embomolmed.org.

***** Reviewer's comments *****

Referee #1 (Remarks for Author):

The authors have addressed all my concerns, and the manuscript has been greatly improved.

Referee #2 (Comments on Novelty/Model System for Author):

None.

Referee #2 (Remarks for Author):

The authors had addressed my concerns in the revised version.

Referee #3 (Comments on Novelty/Model System for Author):

The technical quality are high and the model system is appropriate - there is promise in the data that there could be a therapeutic strategy, but this is only a start.

Referee #3 (Remarks for Author):

Thank you for addressing my comments. One more thing:

1. In the end, now the title is "high glucose" - this is an ill-defined term and may mean many things. I was also critical of "diabetes" so I suggest "hyperglycemia" instead.

We are deeply grateful to all three reviewers for their generous time, remarkable expertise, and highly constructive feedback. The insightful comments provided throughout this process have not only substantially elevated the quality of this manuscript but have also illuminated important directions for future research. All remaining concerns have been carefully addressed, as detailed below.

Referee #1 (Remarks for Author):

The authors have addressed all my concerns, and the manuscript has been greatly improved.

Response: We sincerely thank our Reviewer for the exceptionally thorough and professional evaluation of this manuscript. It is genuinely encouraging to learn that all concerns have been satisfactorily addressed. The reviewer's keen eye for detail and deep expertise in the field has been truly remarkable, and the feedback provided has played a decisive role in elevating the quality and clarity of this work to its current form.

Referee #2 (Comments on Novelty/Model System for Author):

None.

Referee #2 (Remarks for Author):

The authors had addressed my concerns in the revised version.

Response: We are truly grateful to our Reviewer for the perceptive and remarkably constructive assessment of this work. We are genuinely delighted to learn that the revised version has been found satisfactory. The reviewer's exceptional breadth of knowledge and sharp scientific judgment have been evident throughout, and the feedback provided in the previous round has played a pivotal role in sharpening the scientific rigor and depth of the manuscript.

Referee #3 (Comments on Novelty/Model System for Author):

The technical quality are high and the model system is appropriate - there is promise in the data that there could be a therapeutic strategy, but this is only a start.

Referee #3 (Remarks for Author):

Thank you for addressing my comments. One more thing:

1. In the end, now the title is "high glucose" - this is an ill-defined term and may mean many things. I was also critical of "diabetes" so I suggest "hyperglycemia" instead.

Response: We are most grateful to our Reviewer for the continued and dedicated engagement with this work across the process of revision. The reviewer's exceptional expertise and unwavering commitment to

scientific rigor have been truly enlightening, and the thoughtful suggestions offered at every stage have been instrumental in refining both the scientific content and the overall presentation of the manuscript. The invaluable guidance provided throughout this process will undoubtedly continue to inform and inspire our future research endeavors.

The title has been accordingly revised to use "hyperglycemia" instead of "high glucose", as this terminology better reflects the established clinical and scientific convention.

20th Mar 2026

Dear Prof. Zheng,

We are pleased to inform you that your manuscript is accepted for publication and is now being sent to our publisher to be included in the next available issue of EMBO Molecular Medicine.

Your manuscript will be processed for publication by EMBO Press. It will be copy edited and you will receive page proofs prior to publication. Please note that you will be contacted by Springer Nature Author Services to complete licensing and payment information.

You may qualify for financial assistance for your publication charges - either via a Springer Nature fully open access agreement or an EMBO initiative. Check your eligibility: https://link.springer.com/journal/44321/how-to-publish-with-us

Should you be planning a Press Release on your article, please get in contact with embo_production@springernature.com as early as possible in order to coordinate publication and release dates.

If you have any questions, please do not hesitate to contact the Editorial Office. Thank you for your contribution to EMBO Molecular Medicine.

Kind regards,
Jingyi

Jingyi Hou
Senior Editor
EMBO Molecular Medicine

>>> Please note that it is EMBO Molecular Medicine policy for the transcript of the editorial process (containing referee reports and your response letter) to be published as an online supplement to each paper. If you do NOT want this, you will need to inform the Editorial Office via email immediately. More information is available here: https://link.springer.com/partners/embo-press/editorial-policies#Peer%20review

EMBO Press Author Checklist

Corresponding Author Name: Tianpeng Zheng	
Journal Submitted to: EMBO Molecular Medicine	
Manuscript Number: EMM-2025-22469-V3	

USEFUL LINKS FOR COMPLETING THIS FORM
The EMBO Journal - Author Guidelines
EMBO Reports - Author Guidelines
Molecular Systems Biology - Author Guidelines
EMBO Molecular Medicine - Author Guidelines

Reporting Checklist for Life Science Articles (updated January
This checklist is adapted from Materials Design Analysis Reporting (MDAR) Checklist for Authors. MDAR establishes a minimum set of requirements in transparent reporting in the life sciences (see Statement of Task: 10.31222/osf.io/9sm4x). Please follow the journal's guidelines in preparing your
Please note that a copy of this checklist will be published alongside your article.

Abridged guidelines for figures
1. Data
The data shown in figures should satisfy the following conditions:
- the data were obtained and processed according to the field's best practice and are presented to reflect the results of the experiments in an accurate and unbiased manner.
- ideally, figure panels should include only measurements that are directly comparable to each other and obtained with the same assay.
- plots include clearly labeled error bars for independent experiments and sample sizes. Unless justified, error bars should not be shown for technical
- if n<5, the individual data points from each experiment should be plotted. Any statistical test employed should be justified.
- Source Data should be included to report the data underlying figures according to the guidelines set out in the authorship guidelines on Data

2. Captions
Each figure caption should contain the following information, for each panel where they are relevant:
- a specification of the experimental system investigated (eg cell line, species name).
- the assay(s) and method(s) used to carry out the reported observations and measurements.
- an explicit mention of the biological and chemical entity(ies) that are being measured.
- an explicit mention of the biological and chemical entity(ies) that are altered/varied/perturbed in a controlled manner.
- the exact sample size (n) for each experimental group/condition, given as a number, not a range;
- a description of the sample collection allowing the reader to understand whether the samples represent technical or biological replicates (including how many animals, litters, cultures, etc.).
- a statement of how many times the experiment shown was independently replicated in the laboratory.
- definitions of statistical methods and measures:
  - common tests, such as t-test (please specify whether paired vs. unpaired), simple χ2 tests, Wilcoxon and Mann-Whitney tests, can be unambiguously identified by name only, but more complex techniques should be described in the methods section;
  - are tests one-sided or two-sided?
  - are there adjustments for multiple comparisons?
  - exact statistical test results, e.g., P values = x but not P values < x;
  - definition of 'center values' as median or average;
  - definition of error bars as s.d. or s.e.m.

Please complete ALL of the questions below.
Select "Not Applicable" only when the requested information is not relevant for your study.

Materials

Newly Created Materials	Information included in the manuscript?	In which section is the information available? (Reagents and Tools Table, Materials and Methods, Figures, Data Availability Section)
New materials and reagents need to be available; do any restrictions apply?	Not Applicable	

Antibodies	Information included in the manuscript?	In which section is the information available? (Reagents and Tools Table, Materials and Methods, Figures, Data Availability Section)
For **antibodies** provide the following information:<br>- Commercial antibodies: RRID (if possible) or supplier name, catalogue number and or/clone number<br>- Non-commercial: RRID or citation	Yes	Reagents and Tools Table, Materials and Methods

DNA and RNA sequences	Information included in the manuscript?	In which section is the information available? (Reagents and Tools Table, Materials and Methods, Figures, Data Availability Section)
**Short novel DNA or RNA including primers, probes**: provide the sequences.	Yes	Reagents and Tools Table, Materials and Methods

Cell materials	Information included in the manuscript?	In which section is the information available? (Reagents and Tools Table, Materials and Methods, Figures, Data Availability Section)
**Cell lines:** Provide species information, strain. Provide accession number in repository **OR** supplier name, catalog number, clone number, and/**OR** RRID.	Not Applicable	
**Primary cultures:** Provide species, strain, sex of origin, genetic modification status.	Yes	Reagents and Tools Table, Materials and Methods
Report if the cell lines were recently **authenticated** (e.g., by STR profiling) and tested for mycoplasma contamination.	Not Applicable	

Experimental animals	Information included in the manuscript?	In which section is the information available? (Reagents and Tools Table, Materials and Methods, Figures, Data Availability Section)
**Laboratory animals or Model organisms:** Provide species, strain, sex, age, genetic modification status. Provide accession number in repository **OR** supplier name, catalog number, clone number, **OR** RRID.	Yes	Reagents and Tools Table, Materials and Methods
**Animal observed in or captured from the field:** Provide species, sex, and age where possible.	Not Applicable	
Please detail **housing and husbandry conditions**.	Yes	Materials and Methods

Plants and microbes	Information included in the manuscript?	In which section is the information available? (Reagents and Tools Table, Materials and Methods, Figures, Data Availability Section)
**Plants:** provide species and strain, ecotype and cultivar where relevant, unique accession number if available, and source (including location for collected wild specimens).	Not Applicable	
**Microbes:** provide species and strain, unique accession number if available, and source.	Not Applicable	

Human research participants	Information included in the manuscript?	In which section is the information available? (Reagents and Tools Table, Materials and Methods, Figures, Data Availability Section)
If collected and within the bounds of privacy constraints report on age, sex and gender or ethnicity for all study participants.	Yes	Materials and Methods

Core facilities	Information included in the manuscript?	In which section is the information available? (Reagents and Tools Table, Materials and Methods, Figures, Data Availability Section)
If your work benefited from core facilities, was their service mentioned in the acknowledgments section?	Not Applicable	

Design

Study protocol	Information included in the manuscript?	In which section is the information available? (Reagents and Tools Table, Materials and Methods, Figures, Data Availability Section)
If study protocol has been **pre-registered, provide DOI in the manuscript**. For clinical trials, provide the trial registration number **OR** cite DOI.	Not Applicable	
Report the **clinical trial registration number** (at ClinicalTrials.gov or equivalent), where applicable.	Yes	Materials and Methods

Laboratory protocol	Information included in the manuscript?	In which section is the information available? (Reagents and Tools Table, Materials and Methods, Figures, Data Availability Section)
Provide DOI OR other citation details if **external detailed step-by-step protocols** are available.	Not Applicable	

Experimental study design and statistics	Information included in the manuscript?	In which section is the information available? (Reagents and Tools Table, Materials and Methods, Figures, Data Availability Section)
Include a statement about **sample size** estimate even if no statistical methods were used.	Yes	Materials and Methods
Were any steps taken to minimize the effects of subjective bias when allocating animals/samples to treatment (e.g. **randomization procedure**)? If yes, have they been described?	Yes	Materials and Methods
Include a statement about **blinding** even if no blinding was done.	Yes	Materials and Methods
Describe **inclusion/exclusion criteria** if samples or animals were excluded from the analysis. Were the criteria pre-established?<br><br>If sample or data points were omitted from analysis, report if this was due to attrition or intentional exclusion and provide justification.	Yes	Materials and Methods
For every figure, are **statistical tests** justified as appropriate? Do the data meet the assumptions of the tests (e.g., normal distribution)? Describe any methods used to assess it. Is there an estimate of variation within each group of data? Is the variance similar between the groups that are being statistically compared?	Yes	Materials and Methods, Figure Legends

Sample definition and in-laboratory replication	Information included in the manuscript?	In which section is the information available? (Reagents and Tools Table, Materials and Methods, Figures, Data Availability Section)
In the figure legends: state number of times the experiment was **replicated** in laboratory.	Yes	Figure Legends
In the figure legends: define whether data describe **technical or biological replicates**.	Yes	Figure Legends

Ethics

Ethics	Information included in the manuscript?	In which section is the information available? (Reagents and Tools Table, Materials and Methods, Figures, Data Availability Section)
Studies involving **human participants**: State details of **authority granting ethics approval** (IRB or equivalent committee(s), provide reference number for approval.	Yes	Materials and Methods
Studies involving **human participants**: Include a statement confirming that **informed consent** was obtained from all subjects and that the experiments conformed to the principles set out in the WMA Declaration of Helsinki and the Department of Health and Human Services Belmont Report.	Yes	Materials and Methods
Studies involving **human participants: For publication of patient photos**, include a statement confirming that consent to publish was obtained.	Not Applicable	
Studies involving experimental **animals**: State details of **authority granting ethics approval** (IRB or equivalent committee(s), provide reference number for approval. Include a statement of compliance with ethical regulations.	Yes	Materials and Methods
Studies involving **specimen and field samples:** State if relevant **permits** obtained, provide details of authority approving study; if none were required, explain why.	Not Applicable	

Dual Use Research of Concern (DURC)	Information included in the manuscript?	In which section is the information available? (Reagents and Tools Table, Materials and Methods, Figures, Data Availability Section)
Could your study fall under dual use research restrictions? Please check biosecurity documents and list of **select agents and toxins** (CDC): https://www.selectagents.gov/sat/list.htm	Not Applicable	
If you used a select agent, is the security level of the lab appropriate and reported in the manuscript?	Not Applicable	
If a study is subject to dual use research of concern regulations, is the name of the **authority granting approval and reference number** for the regulatory approval provided in the manuscript?	Not Applicable	

Reporting

The MDAR framework recommends adoption of discipline-specific guidelines, established and endorsed through community initiatives. Journals have their own policy about requiring specific guidelines and recommendations to complement MDAR.

Adherence to community standards	Information included in the manuscript?	In which section is the information available? (Reagents and Tools Table, Materials and Methods, Figures, Data Availability Section)
State if relevant guidelines or checklists (e.g., **ICMJE, MIBBI, ARRIVE, PRISMA**) have been followed or provided.	Not Applicable	
For **tumor marker prognostic studies,** we recommend that you follow the **REMARK** reporting guidelines (see link list at top right). See author guidelines, under 'Reporting Guidelines'. Please confirm you have followed these guidelines.	Not Applicable	
For **phase II and III randomized controlled trials**, please refer to the **CONSORT** flow diagram (see link list at top right) and submit the CONSORT checklist (see link list at top right) with your submission. See author guidelines, under 'Reporting Guidelines'. Please confirm you have submitted this list.	Not Applicable	

Data Availability

Data availability	Information included in the manuscript?	In which section is the information available? (Reagents and Tools Table, Materials and Methods, Figures, Data Availability Section)
Have **primary datasets** been deposited according to the journal's guidelines (see 'Data Deposition' section) and the respective accession numbers provided in the Data Availability Section?	Not Applicable	
Were **human clinical and genomic datasets** deposited in a public access-controlled repository in accordance to ethical obligations to the patients and to the applicable consent agreement?	Not Applicable	
Are **computational models** that are central and integral to a study available without restrictions in a machine-readable form? Were the relevant accession numbers or links provided?	Not Applicable	
If publicly available data were reused, provide the respective **data citations in the reference list**.	Not Applicable	
